# Phenylpyrazoles as Inhibitors of the m^6^A RNA-Binding Protein YTHDF2

**DOI:** 10.1021/jacsau.4c00754

**Published:** 2025-02-10

**Authors:** Xiaqiu Qiu, Claus Kemker, Georg L. Goebel, Philipp Lampe, Nadav Wallis, Damian Schiller, Katrin Bigler, Mao Jiang, Sonja Sievers, Gene W. Yeo, Peng Wu

**Affiliations:** † Chemical Genomics Centre, Max Planck Institute of Molecular Physiology, Dortmund 44227, Germany; ‡ Department of Chemical Biology, Max Planck Institute of Molecular Physiology, Dortmund 44227, Germany; § Faculty of Chemistry and Chemical Biology, TU Dortmund University, Dortmund 44227, Germany; ∥ Compound Management and Screening Center, Dortmund 44227, Germany; ⊥ Department of Cellular and Molecular Medicine, University of California San Diego, La Jolla, California 92037, United States; # Sanford Stem Cell Institute and Sanford Consortium for Regenerative Medicine, University of California San Diego, La Jolla, California 92037, United States; ∇ Institute for Genomic Medicine, University of California San Diego, La Jolla, California 92037, United States; ○ Sanford Laboratories for Innovative Medicines, La Jolla, California 92037, United States; ◆ Center for RNA Technologies and Therapeutics, University of California San Diego, La Jolla, California 92037, United States

**Keywords:** RNA m^6^A modification, RNA-binding protein, post-transcriptional regulation, protein−RNA interaction, pyrazole inhibitor

## Abstract

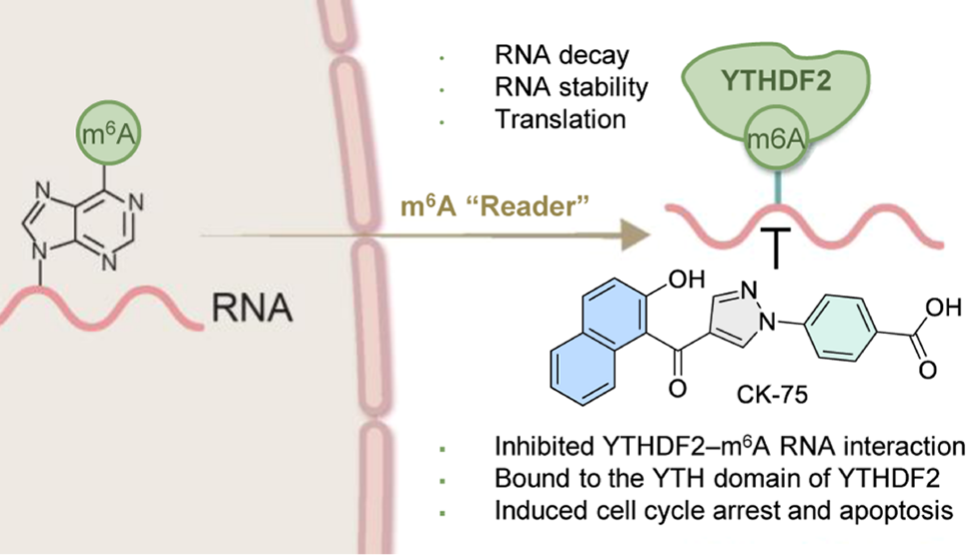

The *N*6-methyladenosine (m^6^A) modification, which is the most common RNA modification in eukaryotes, is regulated by the “writer” methyltransferases, the “reader” m^6^A binding proteins, and the “eraser” demethylases. m^6^A plays a multifunctional role in physiological and pathological processes, regulating all aspects of RNA metabolism and function, including RNA splicing, translation, transportation, and degradation. Accumulating evidence suggests that the YT521-B homology domain family 2 (YTHDF2), one of the m^6^A “readers,” is associated with various biological processes in cancers and noncancerous disorders, impacting migration, invasion, metastasis, proliferation, apoptosis, and cell cycle. Here, we describe our work in the identification of a series of functionalized pyrazoles, such as CK-75, as new YTHDF2 inhibitors, which potentially bind to a small hydrophobic pocket on the YTH domain. Cellular evaluations revealed that the small-molecule YTHDF2 inhibitors induced cell cycle arrest, induced apoptosis, and significantly inhibited the cell viability of cancer cells. Furthermore, we evaluated the transcriptome-wide change in the global RNA-binding protein and RNA-binding patterns of CK-75 via an enhanced cross-linking and immunoprecipitation assay. Our work demonstrated the feasibility of targeting the YTH domain of YTHDF2 with small molecules. The phenylpyrazoles studied in this work provided a lead structure for the further development of small molecules targeting YTHDF2 for both biological and therapeutic applications.

## Introduction

**Figure 1:**
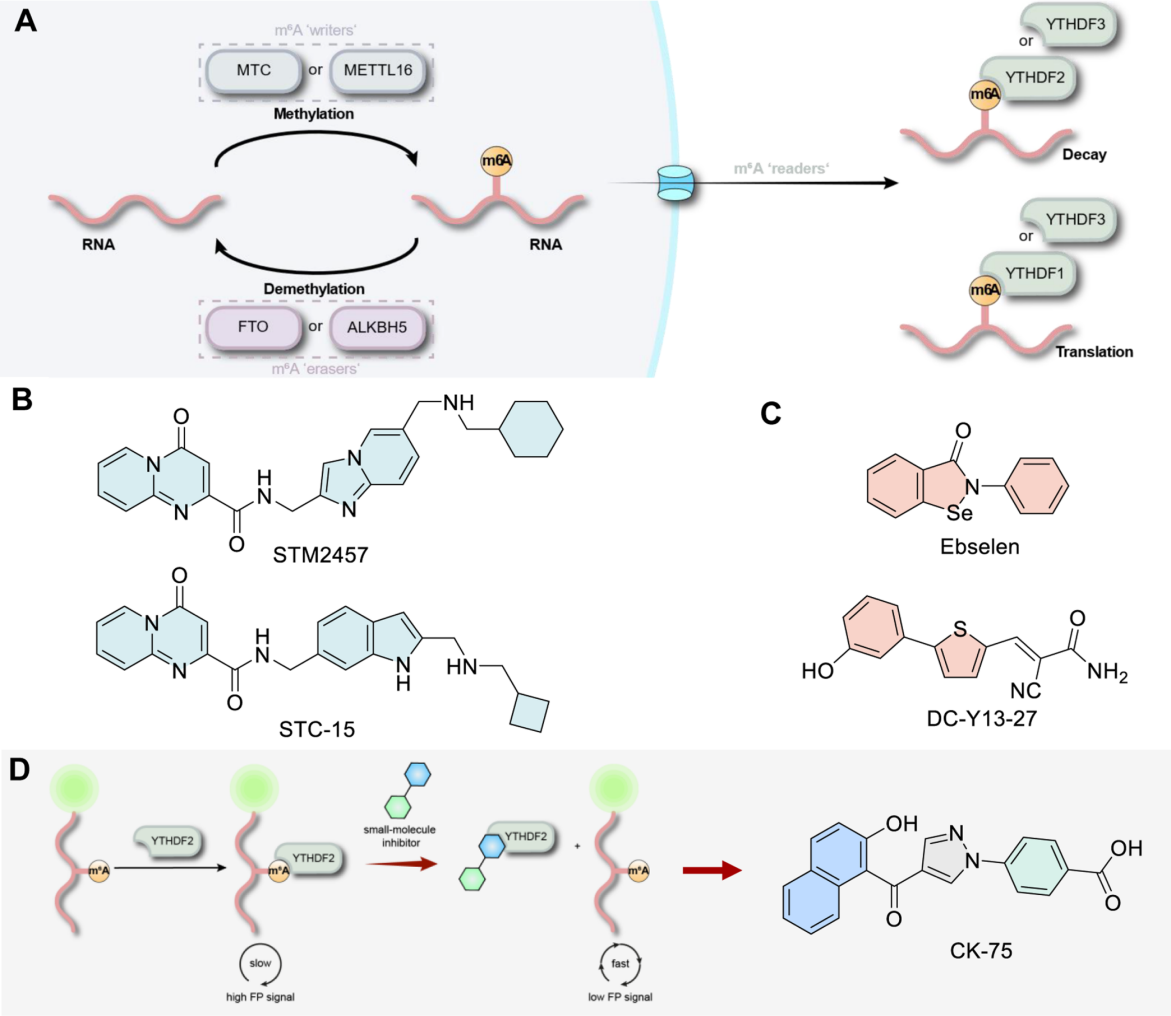
Small-molecule inhibitors targeting m^6^A-binding and modifying proteins. (A) m^6^A is a multifaceted regulator of gene expression via the interplay among methylation installation by writers, demethylation by erasers, and recognition by readers, including YTHDF1–3. (B) STM2457 and STC-15 are among the most advanced small-molecule inhibitors targeting m^6^A RNA-binding and modifying proteins. Both are potent anticancer agents with nanomolar inhibitory activity against METTL3. (C) Reported small molecules inhibiting YTHDF proteins. Ebselen is a covalent nonselective YTHDF inhibitor (YTHDF1, EC_50_: 1.6 μM, YTHDF2, EC_50_: 1.7 μM, tryptophan quenching assay; YTHDF1, IC_50_: 3.6 μM, AlphaScreen). DC-Y13–27 is a synthetic YTHDF2 inhibitor (IC_50_: 21.8 μM, AlphaScreen). (D) A new series of phenylpyrazoles, represented by the most active compound CK-75, were identified in our discovery pipeline as new YTHDF2 inhibitors based on a screening campaign using a fluorescence polarization assay (FP).

RNA epigenetics and post-transcriptional modifications are emerging fields for biomedical research and drug discovery.^1,2^ Among the more than 150 chemical modifications identified on RNAs,^3^ the methylation on the *N*6 position of adenosine (m^6^A) is the most abundant RNA modification in mRNAs and long noncoding RNAs in eukaryotic cells. The m^6^A modification regulates the stability and translation efficiency of mRNA^4^ and is dynamically regulated by the interplay among the m^6^A methyltransferases (writers, such as METTL3 and METTL16),^5^ the demethylases (erasers, such as FTO and ALKBH5),^6^ and the recognition by the m^6^A-binding proteins (readers, such as YTH domain-family proteins including YTHDF1–3, YTHDC1, and YTHDC2).^7^ The reader proteins mediate multiple effects on gene expression via selective binding to m^6^A directly or indirectly, although the exact binding mechanism is unclear. YTHDF2 is one of the first identified m^6^A reader proteins and has been mainly studied for its function in affecting mRNA stability (Figure 1A).^8^ The C-terminal YTH domain of YTHDF2 recognizes and binds to m^6^A-modified mRNA substrates. The N-terminal domain accelerates the degradation of target mRNAs via the recruitment of other protein components.^9^ YTHDF1 is often associated with the promotion of translation of m^6^A-modified mRNAs in the cytoplasm. YTHDF1 and YTHDF3 are described to synergistically accelerate the translation and decay of m^6^A-containing mRNAs together with YTHDF2.^8,10^ The reported crystal structures of YTHDF1 and YTHDF2 bound with m^6^A-containing RNA indicated the shared structural basis for YTHDF proteins’ binding to m^6^A.^11^ Studies have shown that YTHDF2 recruits the complex of CCR4-NOT deadenylase to initiate deadenylation and decay of m^6^A-containing RNAs by interacting with the superfamily homology domain of CNOT1, the scaffold protein of the CCR4-NOT deadenylase complex (Figure 1A).^9^ Accumulating evidence indicated the therapeutic potential of targeting m^6^A RNA-binding and -modifying proteins in diseases,^12^ with initial work mainly focused on the m^6^A demethylase FTO.^13^ Recent progress in the field includes the identification of inhibitors targeting the writer METTL3,^14−16^ such as STM2457 with nanomolar inhibitory activity and STC-15, which has been evaluated in a Phase I clinical trial (Figure 1B),^14^ as well as the first-in-class aminothiazolone inhibitors for the writer METTL16.^17^

YTHDF2 overexpression has been detected in many tumor types, and its expression is correlated with tumor mutation burden, microsatellite instability, and mismatch repair.^18^ Two potential functional mechanisms that affect tumor progression are YTHDF2’s involvement in “spliceosome” and “RNA degradation.” Recently, YTHDF2 inhibition has been reported to trigger apoptosis in triple-negative breast cancer cells and tumors.^19^ Despite the established association with cancers, targeting YTHDF2 remains a challenging task with few reported chemotypes. Except for a few YTHDF2-binding small fragments,^20^ the only available small-molecule examples inhibiting YTHDF2 are the covalent nonselective inhibitor ebselen against YTHDF1–3, DC-Y13–27 that disrupted the m^6^A–YTHDF2 binding with moderate micromolar inhibitory potency, and a series of adenosine analogues that inhibited the five YTH-domain containing proteins (Figure 1C).^21−23^ Small molecules with varied biological performance and new chemotypes are thus highly desired to elucidate the involved m^6^A recognizing mechanism and validate the anticancer strategy of targeting YTHDF2. Herein, we report our discovery pipeline of assay establishment, in-house screening, and the downstream hit-derived structural modifications, which lead to the identification of a series of new noncovalent phenylpyrazoles (Figure 1D) that bound to the YTH domain of YTHDF2, disrupted the interaction between YTHDF2 and m^6^A RNA, affected cellular RNA methylation levels, induced cell cycle arrest and apoptosis, and inhibited the proliferation of K562 cells that expressed YTHDF2.

## Results and Discussion

### Pyrazole CK-23 as the Initial YTHDF2 Hit

**Figure 2:**
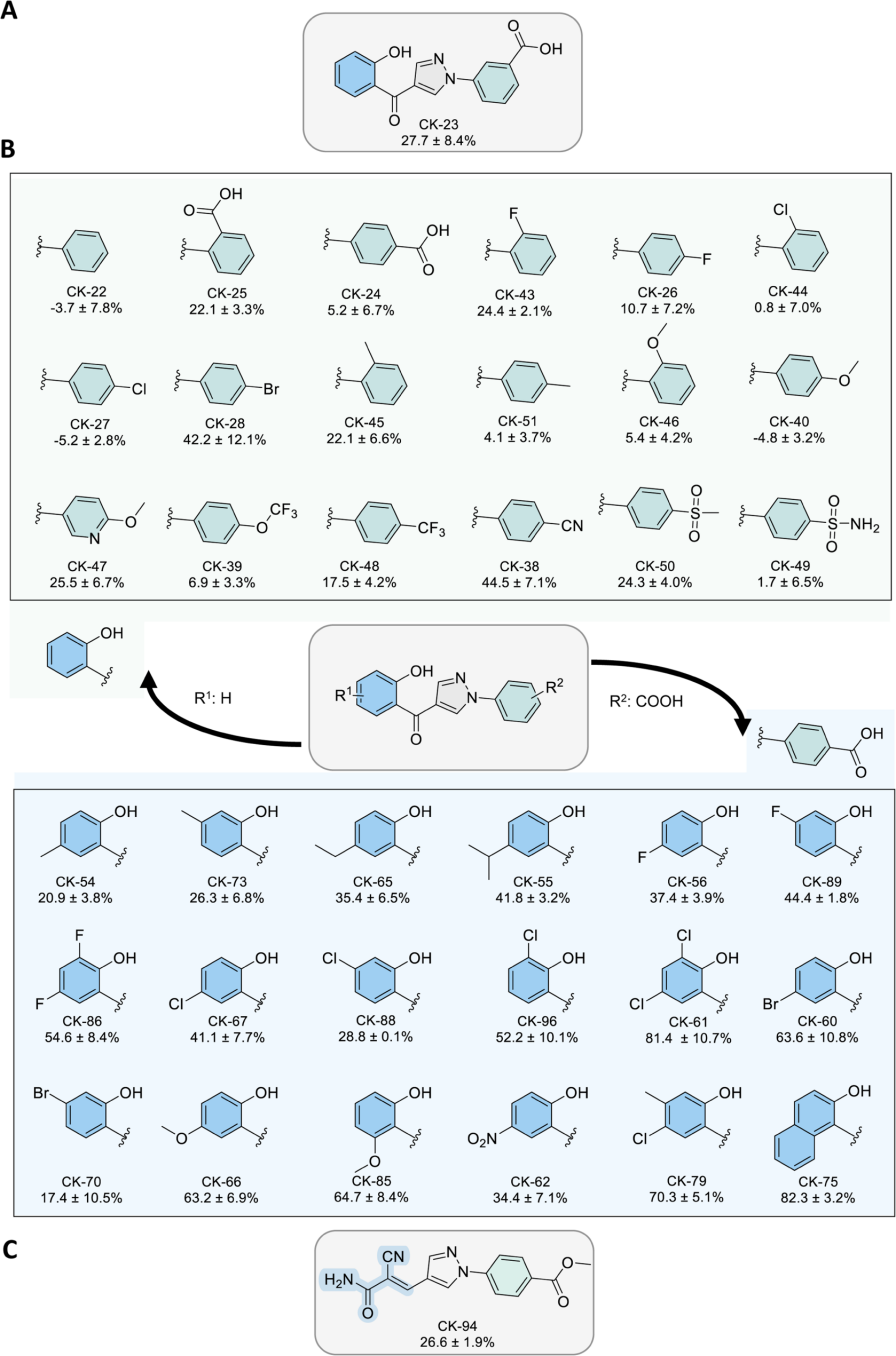
Phenylpyrazoles evaluated as YTHDF2 inhibitors. (A) Structure of the initial hit CK-23 identified from the initial FP-based screening and (B) the 36 in-housed synthesized phenylpyrazoles featuring a 2-hydroxybenzoyl moiety R^1^ (blue) and a 4-carboxyphenyl moiety R^2^ (green) tested using fluorescence polarization (FP); (C) structure of the methylpyrazolylbenzoate CK-94 with a 3-amino-2-cyano-3-oxoprop-1-en-1yl moiety at the 4-pyrazole position. The percentage value indicates the inhibition at 100 μM of the compounds tested in the FP assay using YTHDF2 (residues 383–553, 20 nM) and an FAM-labeled m^6^A RNA probe (UUCUUCUGUGG(m6)ACUGUG, 5 nM). Data represent average values ± SD.

To initiate the discovery of small-molecule modulators targeting YTHDF2, we first established a robust fluorescence polarization assay (FP) using an FAM-labeled 17mer-RNA probe (Figure 1D). The FP showed a high signal-to-noise ratio, with the FAM-unlabeled m^6^A RNA probe showing an IC_50_ of ∼115 nM, while another RNA probe without m^6^A modification showed no inhibitory activity (Figure S1). The established FP enabled in-house screening of ∼15,000 synthetic small molecules. Considering the limited chemotypes that have been associated with YTHDF2-targeting and to ensure that we will not overlook potentially weak inhibitors with new chemotypes, we performed an initial screening at a single concentration of 30 μM, followed by concentration-dependent validation and exclusion of compounds that showed significant autofluorescence. The resulting list of promising hits of different scaffolds, among which was 3-(4-(2-hydroxybenzoyl)-1*H*-pyrazol-1-yl)benzoic acid (CK-23, Figure 2A Table S1), was subject to further evaluations. A collection of 36 structural analogues featuring the phenylpyrazole scaffold of CK-23 was synthesized to explore the structure–activity relationship and identify more potent inhibitors. We were delighted to see that a few phenylpyrazoles of the 36 analogues showed over 50% inhibition against the YTHDF2–m^6^A RNA interaction in the FP assay (Figure 2B), especially for compounds CK-61, CK-75, and CK-79 (all showed >70% inhibition at 100 μM and low double-digit micromolar inhibitory IC_50_, Figure S2). In comparison, the reported inhibitor DC-Y13–27 showed 14.2% inhibition against YTHDF2 at 100 μM in the FP assay. It is noteworthy that a hybrid compound CK-94, which incorporated the phenylpyrazole core scaffold of our in-house hit and the 3-amino-2-cyano-3-oxoprop-1-en-1-yl moiety from DC-Y13–27, only showed moderate inhibition (<30% inhibition at 100 μM) in comparison with that of CK-75 in FP (Figure 2C). In addition to FP, we tested the binding affinity of the inhibitors in a bead-based amplified luminescent proximity homogeneous assay (AlphaScreen), which showed an overall matching result with that of FP. Particularly, compounds CK-55, CK-61, CK-66, CK-67, and CK-75 showed >80% inhibition at 100 μM and low double-digit micromolar inhibitory IC_50_ in AlphaScreen (Figures S3 and S4).

### Identification of CK-75 as the YTHDF2 Inhibitor

**Figure 3:**
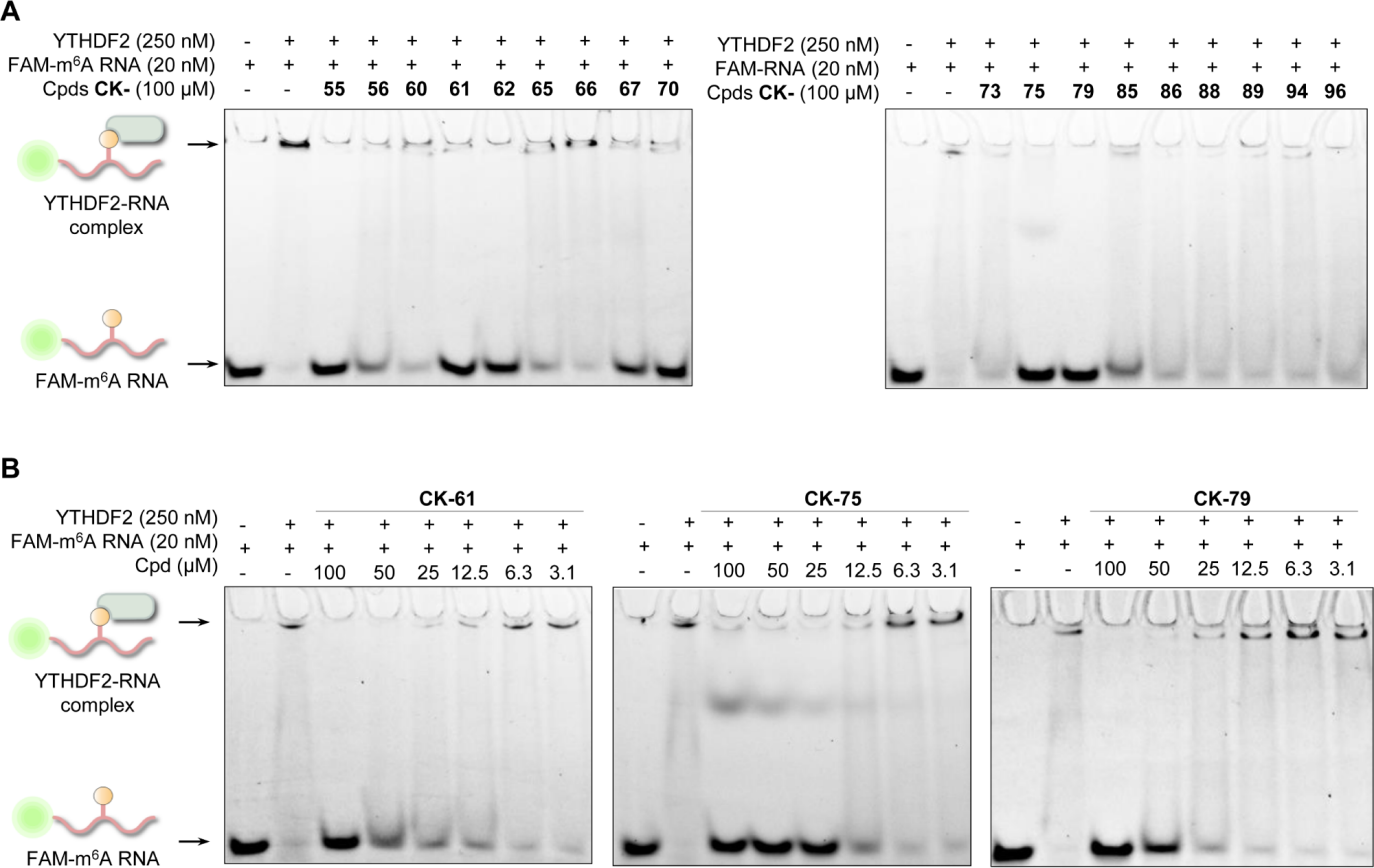
Phenylpyrazoles inhibited the YTHDF2–m^6^A RNA interaction in EMSA. (A) Selected compounds (CK-55 to CK-96) tested in the EMSA at 100 μM, in which compounds CK-55, CK-61, CK-62, CK-67, CK-70, CK-75, and CK-79 potently inhibited the protein–RNA interaction of YTHDF2. The EMSA was performed using an FAM-labeled m^6^A RNA with/without 250 nM YTHDF2 to test the formation of the protein–RNA complex. Electrophoresis of the FAM-labeled RNA probe is shown at the left column of each panel. (B) Compounds CK-61, CK-75, and CK-79 showed potent inhibition for the formation of the YTHDF2–m^6^A RNA complex concentration-dependently.

The phenylpyrazole compounds were validated in a pipeline of orthogonal assays. First, we used an electrophoretic mobility shift assay (EMSA) with an FAM-labeled m^6^A RNA probe. Sufficient sensitivity in detecting the binding was observed at concentrations of YTHDF2 starting from 250 nM (Figure S5). We tested the 37 phenylpyrazoles at 100 μM in EMSA, which showed consistent results with those of FP and AlphaScreen (Figures 3A and S6A). Compounds CK-55, CK-56, CK-61, CK-62, CK-67, CK-70, CK-75, and CK-79 exhibited potent inhibition toward the YTHDF2–m^6^A RNA interaction. The subsequent concentration-dependent inhibition in EMSA confirmed the inhibitory activity of the active compounds (Figures 3B and S6B), especially for CK-75, which showed 100% inhibition at 25 μM, echoing the FP and AlphaScreen results.

Given the EMSA, FP, and AlphaScreen results, seven compounds were selected for further evaluation for their direct binding with YTHDF2 in a nanodifferential scanning fluorimetry thermal shift assay (nanoDSF). Overall, the tested compounds showed a stabilizing effect at either 25 or 10 μM (Figure S7 and Table 1), especially as CK-75 induced a thermal shift of ∼2 °C at 10 μM. The result can probably be explained by the increase in the intramolecular interactions caused by the binding of the small molecules to YTHDF2.

**Table 1:** Thermal Shift (Δ*T*m) of YTHDF2 Upon Addition of the Phenylpyrazoles Determined by nanoDSF

	Δ*T*m/°C		
Cpd.	25 μM	10 μM	5 μM
**CK-55**	1.48 ± 0.52	0.43 ± 0.11	0.15 ± 0.14
**CK-61**	3.21 ± 0.00	1.56 ± 0.41	0.94 ± 0.58
**CK-62**	0.48 ± 0.30	0.50 ± 0.14	0.30 ± 0.05
**CK-67**	2.27 ± 0.26	1.35 ± 0.66	0.47 ± 0.11
**CK-70**	2.42 ± 0.24	1.42 ± 0.36	0.63 ± 0.08
**CK-75**	ND^a^	2.08 ± 0.08	1.37 ± 0.44
**CK-79**	ND^a^	1.39 ± 0.37	0.96 ± 0.08

a ND: not detected.

We then investigated the selectivity of selected inhibitors CK-61, CK-75, and CK-79 against the other YTH domain-family proteins. We expressed and purified the YTH domains of YTHDF1, YTHDF3, YTHDC1, and YTHDC2^23^ and first performed the FP assay to confirm the binding affinities between the YTH domains and the m^6^A RNA probe were consistent with the reported data (Figures S8–S11).^24^ The inhibitory activities of CK-61, CK-75, and CK-79 were subsequently evaluated in FP using the different YTH domains, which revealed that CK-75 and CK-79 were inactive against the YTH domains of YTHDF1, YTHDF3, YTHDC1, and YTHDC2. Interestingly, CK-61 showed weak activities against YTHDF1 (IC_50_: 67.5 μM), YTHDF3 (12.4% inhibition at 100 μM), and YTHDC2 (36.1% inhibition at 100 μM). A further evaluation in EMSA testing the inhibitory activities of CK-61, CK-75, and CK-79 echoed the FP results (Figures S12–S15), which showed that CK-75 and CK-79 did not show detectable inhibition against the binding between m^6^A RNA and the YTH domain of YTHDF1, YTHDF3, YTHDC1, or YTHDC2. CK-61 showed minimal inhibition against YTHDF1, but no activity against the other three YTH domains. Given that the YTH domains share a highly conserved sequence, particularly in the regions involved in RNA binding,^25^ the observed selectivity over YTHDF2 among the tested YTH domains guarantees further investigation.

### Cellular Thermal Shift Assay and Dot Blot Assay

**Figure 4:**
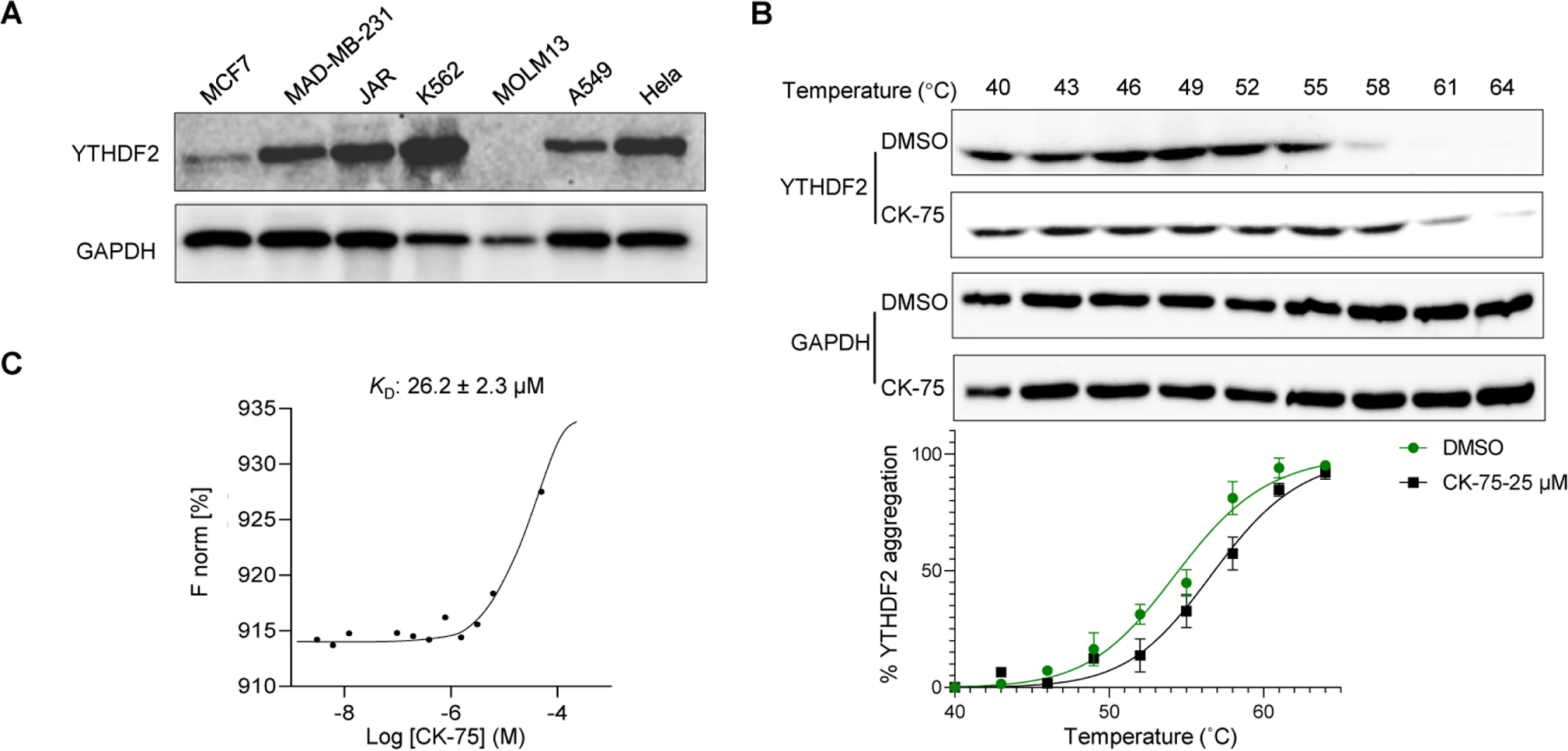
Evaluation of CK-75 in cellular assays. (A) The expression amounts of YTHDF2 in different cancer cell lines. (B) *T*_agg_ curves and the cellular thermal shift assay (CETSA). Western blots were performed for YTHDF2 in K562 cells in the presence of CK-75 (25 μM) and DMSO. CK-75 caused a shift of 2.4 °C in the *T*_agg_ of YTHDF2 in K562 cells. The CETSA data are expressed as mean ± SD (*n* = 2 independent tests), normalized to DMSO-treated samples; relative band intensities were fitted via a sigmoidal curve fit (variable slope). *T*_agg_ was determined where 50% of YTHDF2 aggregated. (C) The binding affinity of CK-75 with YTHDF2 was measured in the microscale thermophoresis (MST).

To investigate the inhibitory potency of the phenylpyrazoles toward the cellular protein–RNA complex involving the YTH domain, we tested the YTHDF2 binding in a cellular thermal shift assay.^26^ The temperature of protein unfolding (aggregation temperature, *T*_agg_) can be modified by binding a small molecule, leading to a thermal shift. In the YTHDF2-expressing K562 cells, CK-75 significantly increased the *T*_agg_ of YTHDF2 by 2.4 °C when treated at 25 μM (Figure 4A,B). Furthermore, we measured the binding affinity of CK-75 for YTHDF2 in the microscale thermophoresis (MST) assay, which showed a *K*_D_ of 26.2 ± 2.3 μM (Figure 4C).

Additionally, we evaluated the cellular RNA m^6^A methylation level in K562 and JAR cells by treating them with selected compounds at different concentrations in the dot blot assay (Figure S16). CK-75 and CK-79 did not significantly impact the global m^6^A RNA abundance, while CK-61 led to a slight increase of m^6^A RNA abundance at the highest tested concentration of 25 μM. The results were unexpected, given that the YTHDF2 knockdown was reported to lead to an increase of the global m^6^A abundance in acute myeloid leukemia cells.^27^ Therefore, it could be that either the current phenylpyrazole inhibitors were not sufficiently potent in cells to induce the cellular m^6^A methylation level or unclarified pathways were involved in the dynamic regulation between YTHDF2 inhibition and m^6^A RNA methylation.

### CK-75 Induced Cell Cycle Arrest and Apoptosis and Inhibited Cell Proliferation

**Figure 5:**
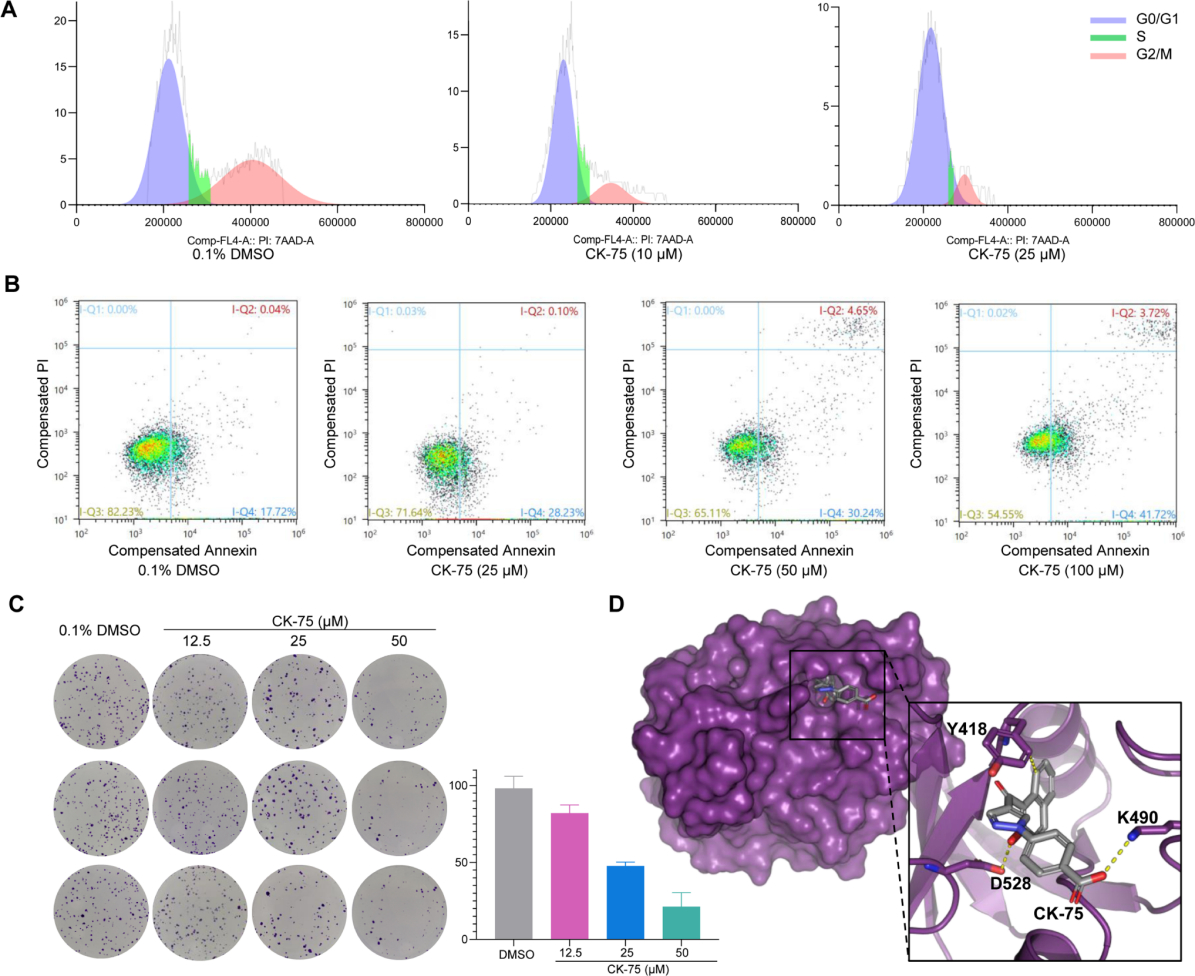
CK-75 induced cell cycle arrest and apoptosis in K562 cells. (A) CK-75 induced cell cycle arrest at the G0/G1 phase in K562 cells measured by flow cytometry. K562 cells were treated with CK-75 for 24 h under the indicated concentrations. The accumulation of cells in the G0/G1 phase indicated the formation of apoptotic cells. Blue: G0/G1 phase; green: S phase; magenta: G2/M phase. (B) CK-75 induced apoptosis in K562 cells in a concentration-dependent manner (total apoptosis: 28.2%, 25 μM; 34.9%, 50 μM; 45.4%, 100 μM) in comparison with that of the DMSO control (total apoptosis: 17.7%). Q1: necrotic; Q2: late apoptotic; Q3: viable; Q4: early apoptosis. Total apoptosis: Q2 + Q4. (C) CK-75 inhibited colony formation in JAR cells (mean ± SD, *n* = 3 biological replicates). (D) The proposed binding mode of CK-75 on the YTH domain of YTHDF2 involving the formation of a π-stacking interaction with Y418, a salt bridge with K490, and a hydrogen bond interaction with D528 (PDB: 4rdn).

Overexpression of YTHDF2 has been reported to inhibit apoptosis in ovarian cancer cells.^28^ Subsequently, we proceeded with the evaluation of CK-75 for its ability to impact cell cycle change and apoptosis in K562 cells (Figure 5A,B). In the cell cycle test, CK-75 induced cell cycle arrest in the G0/G1 phase concentration-dependently (Figure 5A), indicating the formation of apoptotic cells. The majority of cells (>70%) were observed in the G0/G1 phase when treated with 25 μM of CK-75. Furthermore, it was shown that compound CK-75 induced apoptosis in a concentration-dependent manner in K562 cells (Figure 5B). Apoptosis was observed in 28.3% upon the treatment with 25 μM CK-75, 34.9% at 50 μM, and 45.4% at 100 μM. In addition to inducing cell cycle arrest and apoptosis, CK-75 showed micromolar cytotoxicity against the human cancer cells (K562, IC_50_: 29.7 μM, HCT116, IC_50_: 33.3 μM; JAR, IC_50_: 47.5 μM, Table S2) and moderate micromolar inhibitory activity against the proliferation of human cancer cells (K562, IC_50_: 38.5 μM; HCT116, IC_50_: 36.8 μM; JAR, IC_50_: 43.4 μM, Table S3). The results could be associated with the modulation of the YTHDF2-mediated apoptosis. However, given the complex apoptotic-inducing cellular network, the exact apoptotic mechanism needs to be further studied. To further evaluate the cellular activity, CK-75 was tested in a colony-forming assay in JAR cells, in which CK-75 concentration-dependently inhibited the colony formation (Figure 5C).

### CK-75 May Bind to the RNA-Binding Site of YTH Domain

To probe the potential binding mode for the phenylpyrazoles, we conducted a molecular docking analysis of CK-75, the best-performed YTHDF2 inhibitor, based on a complex structure between the YTH domain and an m^6^A substrate.^29^ The optimal binding configuration revealed key interactions that were consistent with the observed structure–activity relationship in in vitro and cellular assays (Figure 5D). The 1-naphthoyl moiety of CK-75 fits into the m^6^A-binding site, forming a stabilizing π-stacking interaction with Y418, one of the amino acids forming the aromatic cage. The carboxylic acid residue of CK-75 could form a stable salt bridge with K490, explaining the association between YTHDF2 inhibitory potency and the presence of a carboxylic acid in this series of phenylpyrazole inhibitors. Additionally, a hydrogen bond interaction between the hydroxyl group of the naphthoyl and D528 potentially contributed to the binding affinity. Overall, the potential binding of CK-75 at the RNA-binding site of the YTH domain involving the aromatic cage residue can be the mechanism of inhibition for the phenylpyrazole inhibitors.

### Transcriptome-Wide Evaluation of CK-75 in eCLIP

Given that YTHDF2 has been reported to function in stabilizing mRNA and mediating mRNA degradation, we proceeded with a transcriptome-wide evaluation of whether the global RBP- and RNA-binding patterns would be impacted by CK-75 in an enhanced cross-linking and immunoprecipitation (CLIP) assay.^30^ K562 cells were UV irradiated to cross-link proteins and associated RNAs. RNA immunoprecipitation (RIP) was performed to pull down YTHDF2 with an anti-YTHDF2 antibody. In the following deep sequencing analysis, the associated RNAs were analyzed, and the resulting windows (peaks) were mapped to their corresponding RNAs.

**Figure 6:**
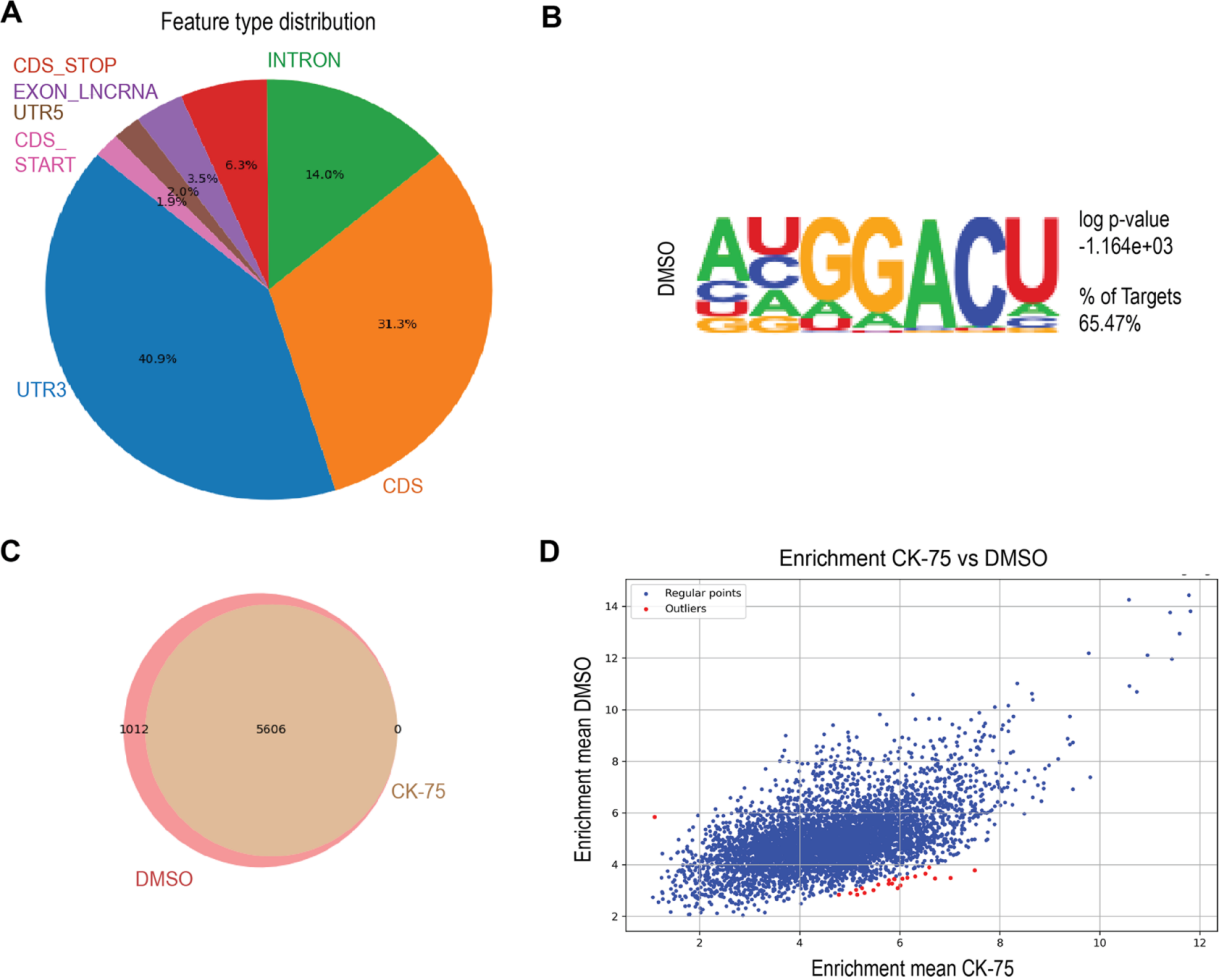
Transcriptome-wide evaluation via eCLIP in K562 cells. (A) The distribution of the windows in DMSO-treated cells in pie charts based on the region in the RNA to which they map. (B) HOMER analysis of enriched motifs in eCLIP peaks from DMSO-treated K562 cells showing the DRACH motif. (C) Venn diagram showing the overlap of significant windows in the CK-75-treated eCLIP cells in comparison with that of the DMSO-treated cells. The large majority of DMSO eCLIP windows (5606/6618 = 84.71%) were significantly enriched in the CK75 with a log2 fold change of >1 and a *p* value < 0.05. (D) The enrichment mean IP/IN of the eCLIP DMSO (*Y*-axis) with the enrichment mean of CK-75 (*X*-axis) was plotted. Windows with Z score above and below 3 were highlighted in red. The K562 cells were treated for 4 h with either DMSO or 20 μM CK-75. Tested in duplicates.

The analysis revealed that 6618 windows were reproducible in untreated cells (Table S4). Only reproducible windows between two replicates were used, and windows were filtered (FDR < 0.2). Most of the windows were in the 3′UTR and at the CDS, consistent with data from previous YTHDF2 eCLIP^19^ and with m^6^A locations detected with m^6^A-seq^31^ (Figure 6A). The eCLIP data allowed us to identify motifs enriched with YTHDF2-bound RNAs. HOMER analysis was performed to identify and compare enriched motifs, which revealed that the most common motif (65.47% of windows) in the eCLIP was the same m^6^A: DRACH (D = A, G, or U; *R* = A or G; H = A, C, or U) sequence motif (Figure 6B).

To measure the global effect of CK-75 on RNA binding, we performed eCLIP in K562 cells treated with 20 μM of CK-75 for 4 h to minimize secondary transcription effects. The CK-75 eCLIP showed the same RNA-binding feature pattern as the DMSO eCLIP (Figure S17A), and the same DRACH motif as the most abounded and significant motif (Figure S17B and Table S5). Enrichment (IP/IN) was calculated on the reproducible windows from the nontreated eCLIP. Treated enrichment was defined for windows that were reproducible in both replicates, with a log2 fold change greater than 1 and a *p* value less than 0.05. Out of 6618 windows in the DMSO eCLIP, the vast majority (5606 windows) was also significantly enriched in the treated eCLIP (Figure 6C). We sought to assess the differential enrichment windows between CK-75-treated and untreated samples, which reflected the binding strength of YTHDF2 to RNA. A Z-score was calculated for each window, and windows with a Z-score greater than 3 or lower than −3 were considered differentially bound. Out of all windows, only 1 window was differentially enriched in DMSO, while 23 windows were enriched in the CK-75-treated cells (Figure 6D) (Table S6). Since the windows were similar between the treated and untreated eCLIPs, this may indicate that a higher potency is needed to observe significant inhibition of global RNA binding in cells. Another possibility is that the additional windows observed in the CK-75 eCLIP suggested a gain of function of CK-75 on YTHDF2; However, further experiments are needed to determine whether this is due to the impact of CK-75 treatment or the inherent variability in RNA binding and eCLIP.

## Conclusion

The m^6^A RNA-binding protein YTHDF2 is one of the m^6^A effector proteins that plays important roles in epitranscriptomic regulations. Whereas targeting YTHDF2 with small molecules holds great therapeutic potential, few reports and very limited chemotypes are currently available. In this study, we identified a series of phenylpyrazoles as new YTHDF2-targeting inhibitors binding with the YTH domain via a discovery pipeline that started with an in-house screening. Based on the initial phenylpyrazole hit CK-23, we evaluated a collection of 37 synthesized structural analogues, among which we identified CK-75 that concentration-dependently inhibited the formation of the YTHDF2–m^6^A RNA complex in vitro and may bind to YTHDF2 in K562 cells, as shown in a cellular thermal shift assay. In addition to YTHDF2 inhibition, CK-75 induced cell cycle changes in K562 with increased arrest at the G0/G1 phase, induced apoptosis and inhibited colony formation, and showed moderate inhibitory activity against cell proliferation. One limitation in the current study lies in the lack of specificity assessment in YTHDF2-deficient cells, which would be important to directly attribute that the observed cellular effects are consequences of targeting YTHDF2. Such YTHDF-depleted control would facilitate a clear interpretation of the inhibitors’ mechanism action. In an eCLIP assay evaluating the RBP and RNA-binding patterns, CK-75 did not lead to significant changes in the global RBP-binding pattern in K562 cells, which indicated that YTHDF2 inhibitors with more potent inhibitory activity are needed to validate whether YTHDF2 inhibition would lead to changes at the transcriptomic level. Although it is not yet clear whether YTHDF2 inhibition alone would be a sufficient anticancer strategy, the phenylpyrazole inhibitors discovered from this study can be useful to evaluate further application in combination with other anticancer options^22^ and for the elucidation of the regulatory mechanisms involved in m^6^A modulation.

## Experimental Section

### Protein Expression and Purification

A YTHDF2 YTH domain construct (residues 383–553) was subcloned to a pOPIN plasmid containing subsequent N-terminal His6 and GST tags. Plasmids of the YTH domain of YTHDF1 (residues 375–552), YTHDF3 (residues 391–585), YTHDC1 (residues 345–509), and YTHDC2 (residues 1285–1424) were obtained from the Zhou’s lab (Boston College). The plasmids were transformed into chemically competent *Escherichia coli* BL21(DE3) and cultured in LB medium until reaching an OD600 of 0.6. Expression of the fusion protein was then induced by the addition of 300 μM IPTG at 18 °C for 18 h. The cells were collected and lysed by sonication in buffer (20 mM HEPES, pH 7.4, 200 mM NaCl, 5% glycerol, 0.1 mM PMSF, 10 μg/mL DNase I, 10 μg/mL lysozyme, and SIGMAFAST Protease inhibitor cocktail). After centrifugation of the lysate at 60000 × g and 4 °C for 1 h, the supernatant was purified via immobilized metal affinity chromatography using a 5 mL HisTrap column (Cytiva) equilibrated with buffer A, which contained 20 mM HEPES (pH 7.4), 200 mM NaCl, and 5% glycerol. The protein was eluted with a gradient of buffer A containing up to 0.5 M imidazole. Fractions containing YTHDF2 were pooled. The protein solution was again passed through the HisTrap column, concentrated, and further purified using a High Load Superdex 75pg 16/600 column (Cytiva) equilibrated with buffer A. The purified and concentrated protein was stored at −80 °C until further use.

### Cell Culture

JAR, K562, HCT116, MCF7, MOLM13, A549, and MDA-MB-231 cells were purchased from the Leibniz German Collection of Microorganisms and Cell Cultures GmbH (DSMZ, Braunschweig, Germany). JAR, K562, MOLM13, and HeLa cells were grown in the RPMI 1640 medium (Gibco). HCT116, MCF7, A549, and MDA-MB-231 cells were grown in DMEM with GlutaMAX (Gibco). All of the cells were supplemented with 10% FBS (Gibco) in a 37 °C humidified incubator with 5% CO_2_.

### Compound Screening

The screening was performed against a chemical library of 15500 compounds at the Compound Management and Screening Center (COMAS, MPI Dortmund). An FP assay, described in detail below, with truncated YTHDF2 protein (YTH-domain) and FAM-17mer-m6A RNA (UUCUUCUGUGG(m6)ACUGUG, IDT) was performed in 1536-well plates (Corning no. 3728) with a 30 μM compound concentration in a final volume of 8 μL. Multidrop dispensers (Thermo Fisher) were used for the addition of protein and substrate solution. The compounds were added to the protein solution with an ECHO520/550 acoustic liquid handler (Labcyte), followed by incubation at room temperature for 30 min. Upon addition of the substrate and incubation for an additional 30 min at room temperature, the fluorescence polarization was detected with an EnVision XCite multimode plate reader (PerkinElmer). Primary hits were tested in serial dilutions in 384-well plates (Corning no. 4514) in a final volume of 15 μL to determine IC_50_ values. CK-23 was one of the initial hits identified through primary screening.

### Fluorescence Polarization (FP) Assay

The FP assay was performed in 384-well black plates (Corning no. 4514) with a total reaction volume of 15 μL. The final concentrations of protein (YTHDF2) and RNA were 20 and 5 nM, respectively, in a buffer containing 20 mM HEPES/KOH pH 7.5, 50 mM NaCl, 0.005% Tween 20, and 1 mM GSH (DTT). Compounds were incubated with protein, added FAM-m^6^A RNA probe (UUCUUCUGUGG(m6)ACUGUG) (purchased from IDT), and incubated at 4 °C for 30 min. The fluorescence polarization was measured using a plate reader (TECAN Spark) under an excitation wavelength of 485 nm and an emission wavelength of 535 nm with a bandwidth of 20 nm. The inhibition rate was calculated based on the following equation: inhibition rate = 100% (Control – X)/(Control – Blank); control: DMSO with protein and FAM-RNA; blank: DMSO with FAM-RNA; X: compound with protein and FAM-RNA. The data were determined using GraphPad Prism 9.

### Electrophoretic Mobility Shift Assay (EMSA)

Compounds with different concentrations (3.1–100 μM) were incubated with 250 nM YTH-domain and 20 nM FAM-17mer-RNA in a buffer containing 20 mM HEPES, pH 8.2, 50 mM NaCl, 0.05% Triton X-100, and 5% v/v glycerol for 20 min at 4 °C. The samples were subjected to 12% native polyacrylamide gel and run at 120 V for 90 min at 4 °C using 0.5× TBE buffer. Gel imaging was performed in ChemiDoc MP (Bio-Rad), and Cy2/Alexa488 fluorescence was detected with a 1-min exposure time. Quantification of the gel image was performed by ImageJ.

### AlphaScreen Assay

Initially, the YTHDF2 YTH domain at different concentrations (0–2500 nM) was incubated with varied concentrations of RNA probes (5’-Bi-CGAm6ACUGUC–3′, IDT company) (5, 25, 50, and 100 nM) in 25 mM Hepes (pH 7.5), 100 mM NaCl, and 0.1% BSA to probe the optimal ratio between protein and RNA before the detection signal reached saturation. The AlphaScreen Histidine Detection Kit (Nickel Chelate) (PerkinElmer) 6760619 M and white 384-well Optiplates (PerkinElmer) were used. Anti-His receptor beads (PerkinElmer) (final concentration: 20 μg/mL) and streptavidin donor beads (final concentration: 20 μg/mL) were first added. The reaction mixture was then incubated at room temperature in a dark environment for 1 h to reach equilibrium. The optical signal was detected using a Tecan plate reader.

### Nano Differential Scanning Fluorimetry

NanoDSF was measured on a NanoTemper instrument (Prometheus NT.48). YTHDF2 protein was used at a concentration of 5 μM and incubated with 50, 25, 10, and 5 μM compound in buffer (100 mM KPi, pH 7.0, 250 mM NaCl, 10% Glycerol) for 50 min, in which 0.1% DMSO was used as the reference. The heat rate was set to 1 °C per minute, measured in a range between 20 and 90 °C.

### Cellular Thermal Stability Assay

JAR cells were seeded in cell culture plates (100 mm) and incubated overnight. Cells were treated with 50 μM compound or 0.1% DMSO and incubated for 24 h. Cells were collected and washed in PBS, and then, the lysis buffer (RIPA: 1 mM PMSF = 99:1) (SERVA) was added. Lysates were then divided into 9 aliquots of 50 μL in 0.2 mL microcentrifuge tubes and incubated at a temperature gradient (40–64 °C) on a PCR machine for 3 min. Lysates were then frozen and thawed three times on dry ice and at room temperature for 3 min each. All tubes were centrifuged at 4 °C for 20 min at 12,000 rpm, followed by the collection of the supernatants. The protein concentration was determined by a BCA kit (SERVA), and then, the supernatants were transferred to clean tubes with a loading buffer 5×, which were heated for 5 min at 95 °C. Primary antibody YTHDF2 (Proteintech, 24744–1-AP, 1:15000) and secondary antibody Goat Anti-Rabbit IgG Antibody (H+L), HRP Conjugated (bs-0295G-HRP, 1:5000) were used. Chemiluminescent detection was performed using a ChemiDoc MP instrument (Bio-Rad).

### Western Blot

JAR, K562, MCF7, MOLM13, A549, HeLa, and MDA-MB-231 cells were seeded in 6-well plates (1.0 × 10^6^ cells/well) and incubated overnight. The cells were detached with trypsin or collected directly and then washed twice with PBS. Following the addition of the lysis buffer (RIPA: 1 mM PMSF = 99:1) (SERVA), all samples were kept on ice for 30 min and then centrifuged at 4 °C for 20 min at 12,000 rpm. Supernatants were then collected. The protein concentration was determined by a BCA kit (SERVA), and then, the supernatants were transferred to clean tubes with a loading buffer 5×, which were heated for 5 min at 95 °C. Primary antibody YTHDF2 (Proteintech, 24744–1-AP, 1:15000) and secondary antibody Goat Anti-Rabbit IgG Antibody (H+L), HRP Conjugated (bs-0295G-HRP, 1:5000) were used. Chemiluminescent detection was performed using a ChemiDoc MP (Bio-Rad).

### Dot Blot

JAR and K562 cells were seeded in six-well plates (1.0 × 10^6^ cells/well) and incubated overnight. The cells were scraped off directly and then washed with PBS. Total RNA was then extracted with the Aurum Total RNA Mini Kit. The RNA concentration was determined by NanoDrop 2000c (Thermo) and then heated for 5 min at 95 °C. Primary antibody Rb pAb to N^6^-methyladenosine (m^6^A) (Abcam, ab286164, 1:1000) and secondary antibody HRP-conjugated AffiniPure Goat Anti-Rabbit IgG(H+L) (SA00001–2, 1:8000) were used. Chemiluminescent detection was performed using a ChemiDoc MP (Bio-Rad).

### Apoptosis Assay

K562 cells were seeded in six-well plates (1.0 × 10^6^ cells/well) and incubated overnight. Compounds of different concentrations were added to the 6-well plates. After incubation for 24 h, the cells were detached with trypsin, washed twice with PBS and cold BioLegend’s cell staining buffer, and then, 5 μL of FITC Annexin V and 10 μL of propidium iodide solution were added. The cells were gently vortexed and incubated in a dark environment for 15 min at room temperature (25 °C). After adding 400 μL of Annexin V binding buffer to each tube, each sample was analyzed by flow cytometry (Sony Biotechnology Cell Sorter SH800S) with proper machine settings, and the data were analyzed by cell sorter software (Sony Biotechnology).

### Cell Cycle Analysis

K562 cells were seeded in 6-well plates (1.0 × 10^6^ cells/well) and incubated overnight. Compounds of different concentrations were added to the 6-well plates. The cell cycle analysis kit (SIGMA-Aldrich, MAK344) was used after incubation for 24 h. The cells were detached with trypsin and washed with 2 mL of ice-cold cell cycle assay buffer for each sample. The supernatant was removed after centrifugation. The cells were fixed by adding 2 mL of ice-cold 70% ethanol (added dropwise while vortexing) to the cell pellet and then placed on ice for 30 min. The supernatant was removed after centrifuging at 400 × g for 5 min. Cells were washed with a cell cycle assay buffer (2 mL). Cells were resuspended completely in 500 μL of staining solution and were protected from light exposure. After being incubated at room temperature for 30 min, each sample was analyzed by flow cytometry (Sony Biotechnology Cell Sorter SH800S) with proper machine settings. The data were analyzed by cell sorter software (Sony Biotechnology).

### MTT Assay

JAR, K562, and HCT116 cells were seeded in 96-well plates (3500 cells/well) and incubated for 8 h. The medium with different concentrations of compounds (100, 33.3, 11.1, 3.7, 1.2, and 0.4 μM) was added to the 96-well plates. Data were normalized to the medium with 0.1% DMSO. After incubation for 72 h, MTT solution (5 mg/mL, 20 μL) was added per well in a dark environment and incubated for 4 h. After the medium was discarded, DMSO (150 μL) was added per well, and the absorbance was measured at 492 nm by a plate reader (TECAN Spark).

### Cell Proliferation and Cytotoxicity Assay CCK8

The JAR, K562, HCT116, and MOLM13 cells were seeded in 96-well plates (3000 cells/well). After being cultured overnight, compounds were added together with the DMSO treatment (0.1%) as a control. After 72 h, CCK-8 solution (Vazyme, A311) was added, and the cells were incubated at 37 °C for 4 h. The absorbance was measured at 450 nm using a plate reader (TECAN Spark).

### Colony Formation Assay

JAR cells in the growth phase are prepared into cell suspensions by traditional digestion. A cell suspension (2 mL) with 200 cells per dish was seeded into a 6-well plate and incubated for 7 days. When visible colonies are present in the 6-well plate, the medium is discarded. The colonies are carefully soaked twice in PBS and dried in air. Cells were fixed with a 4% paraformaldehyde solution for 15 min. The paraformaldehyde solution was discarded, and Giemsa was used to stain the cells for 10 min. The dye solution was slowly washed away with Milli-Q water and then dried in air. The colony number and type were assessed manually under a microscope.

### MST Binding Affinity Assay

Monolith His-Tag Labeling Kit RED-tris-NTA Second Generation (catalog no. MO-L018) was used. The affinity of the dye to the target protein YTHDF2 was tested. The *K*_D_ value for YTHDF2 is 623 nm. Then, 10 times the protein *K*_D_ value was used to label YTHDF2 protein at 6.23 μM. The labeled protein was used to test the binding affinity of compound CK-75 binding to YTHDF2. NanoTemper MST Monolith Nt.115 was used to obtain the direct *K*_D_ value. The test was repeated three times.

### Docking Analysis

To analyze a potential binding mode of pyrazoles, docking analysis was conducted by docking the compounds to the RNA binding site of YTHDF2 (PDB code: 4RDN). Schrödinger Maestro 12.3 was used for the analysis. First, 3D structures of compounds were prepared after performing MM2 energy minimization (PerkinElmer Chem3D 22.2). The ligand preparation module and YTHDF2 conformation with the protein preparation module were used to generate the chemical states. Protein preparation included the addition of hydrogen atoms, the removal of water molecules, and energy minimization. Crucial interactions of m^6^A with YTHDF2 were identified based on the resolved structure of the protein-m^6^A complex and then used to identify potential binding sites for the pyrazoles. The grid generation module was used to generate the binding site of YTHDF2 for docking. Finally, the glide dock module was used, and the results were evaluated according to interactions between small molecules and YTHDF2, small molecule orientations, docking scores, and solvent exposure patterns. The interactions of pyrazoles and YTHDF2 were visualized by using PyMOL.

### Enhanced UV Cross-Linking and Immunoprecipitation (CLIP) Followed by Sequencing

#### Library Preparation

A total of 40 mL with 80 × 10^6^ cells were divided into 10 cm plates (2 plates per condition). 20 μM CK75 (20 μM) or 0.01% DMSO was then added for 4 h. After incubation, cells were spun down (300*g*, 3 min), and the pellet was resuspended in 3 mL of cold DPBS to a concentration of ∼6 million cells/mL. YTHDF2–RNA interactions were stabilized with UV cross-linking (254 nm, 400 mJ cm^–2^) in cold DPBS. The cells were collected and frozen, and the thawed pellets were then lysed in iCLIP lysis buffer (1 mL) and digested with RNase I (Ambion). Immunoprecipitation of YTHDF2–RNA complexes with YTHDF2 primary antibody (Aviva ARP67917_P050, 10 μg per mL of lysate) was performed using magnetic beads with precoupled secondary antibody (*M*-280 sheep antirabbit IgG Dynabeads, Thermo Fisher Scientific, 11204D). The beads were stringently washed. A barcoded RNA adapter was ligated to the 3′ end (T4 RNA Ligase, NEB) after dephosphorylation with FastAP (Thermo Fisher) and T4 PNK (NEB). To allow washing away of the unincorporated adapter, on-bead ligations were performed in a high concentration of PEG8000. Samples were then run on standard protein gels and transferred to nitrocellulose membranes. Isolation of a region 75 kDa (∼220 nucleotides of RNA) above the protein size and treatment with proteinase K (NEB) was performed to isolate RNA, which was then reverse transcribed with Superscript III (Invitrogen) and treated with ExoSAP-IT (Affymetrix) to remove excess oligonucleotides. A second DNA adapter (containing a random-mer of 10 (N10) random bases at the 5′ end) was then ligated to the cDNA fragment 3′ end (T4 RNA Ligase, NEB), which was performed at a high concentration of PEG8000 to improve ligation efficiency and DMSO to decrease the inhibition of ligation due to the secondary structure. Following cleanup (Dynabeads MyOne Silane, Thermo Fisher), an aliquot of each sample was first subjected to quantitative PCR to identify the proper number of PCR cycles. The remainder was PCR amplified (Q5, NEB) and size-selected via agarose gel electrophoresis. Samples were sequenced on an Illumina NovaSeqXPlus platform.

#### Data Analysis

The eCLIP libraries were processed with Skipper (https://github.com/YeoLab/skipper/).^32^ In short, reads were adapter trimmed with Skewer. Reads less than 20 bp were discarded. UMI was extracted using fastp 0.11.5 (https://github.com/OpenGene/fastp). Mapping was performed against the full human genome (hg38), which included a database of splice junctions with STAR (v 2.7.6) that permitted up to 100 multimapped regions. Reads are randomly assigned to any of the matching positions when multimapping occurred. UMICollapse was used for the PCR deduplication of the reads (https://github.com/Daniel-Liu-c0deb0t/UMICollapse). A GC-bias aware beta-binomial model was used to call enriched “windows” (IP versus SM-Input) on deduplicated reads. Each window (∼100 bp) was partitioned from Gencode v38 and associated with a specific type of genomic region (CDS, UTR, proximal introns near splice site). Filtration of the windows using FDR < 0.2 was performed, and only reproducible windows between two replicates were used.

For motif calling, the creation of 75 nt control windows was performed by selecting a random window in the partitioned transcriptome of the same feature group within 500 nt of the exon-intron boundary (e.g., noncoding exons, mRNA exons, proximal introns), or by randomly selecting a center for distal introns. For fine-mapped windows, the findMotifsGenome.pl script from HOMER was run with the following options: -preparsedDir -size given -rna -nofacts -S 20 -len 5,6,7,8,9 -nlen 1 -bg. In the gene ontology analysis, windows lying in genes belonging to each term were tallied and compared to the representation of each term across all ENCODE 3 CLIPs to control for expression, library preparation bias, and mappability. *p* Values were calculated by a binomial test with the rate of success equal to the representation of the term in the ENCODE 3 CLIPs and Bonferroni corrected for multiple hypotheses.

## Supporting Information Available

Figures S1–S17, Tables S1–S6, general chemistry information, synthetic procedures, compound characterizations, and ^1^H NMR, ^13^C NMR, and UPLC-MS spectra (PDF)
