# Supplementary material for: Phenylpyrazoles as Inhibitors of the m6A RNA-Binding Protein YTHDF2
Source: JACS Au. 2025 Feb 10;5(2):618–30. doi: 10.1021/jacsau.4c00754 (PMC11862924; doi:10.1021/jacsau.4c00754)
Supplement: Supplementary file 1 — au4c00754_si_001.pdf [file au4c00754_si_001.pdf]

Supporting information

Phenylpyrazoles as inhibitors of the m^{6}A RNA-binding protein YTHDF2

Xiaqiu Qiu,[1-3,#] Claus Kemker,[1-3,#] Georg L. Goebel,[1-3] Philipp Lampe,[2,4] Nadav Wallis,[5] Damian Schiller,[3] Katrin Bigler,[3] Mao Jiang,[1-3] Sonja Sievers,[2,4] Gene W. Yeo,[5-9] and Peng Wu[1-3,*]

[1]Chemical Genomics Centre, Max Planck Institute of Molecular Physiology, Dortmund 44227, Germany

[2]Department of Chemical Biology, Max Planck Institute of Molecular Physiology, Dortmund 44227, Germany

[3]Faculty of Chemistry and Chemical Biology, TU Dortmund University, Dortmund 44227, Germany

[4]Compound Management and Screening Center, Dortmund 44227, Germany

[5]Department of Cellular and Molecular Medicine, University of California San Diego, La Jolla, California 92037, United States

[6]Sanford Stem Cell Institute and Sanford Consortium for Regenerative Medicine, University of California San Diego, La Jolla, California 92037, United States

[7]Institute for Genomic Medicine, University of California San Diego, La Jolla, California 92037, United States

[8]Sanford Laboratories for Innovative Medicines, La Jolla, California 92037, United States

[9]Center for RNA Technologies and Therapeutics, University of California San Diego, La Jolla, California 92037, United States

[#]Equally contributing authors

*Corresponding author: P.Wu, email: peng.wu@mpi-dortmund.mpg.de

CONTENTS

SUPPLEMENTARY FIGURE S1–S17…….………………………….……….S3

SUPPLEMENTARY TABLE S1 –S3..………..…...…………………..…S22

GENERAL CHEMISTRY INFORMATION ……………………………….S26

SYNTHETIC PROCEDURE………………………………………………… S28

NMR SPECTRA ………………………………………………………....S51

LC-MS Spectra……………..……………………………………………S139

REFERENCES……………..…………………………………………...…S154

SUPPLEMENTARY FIGURES

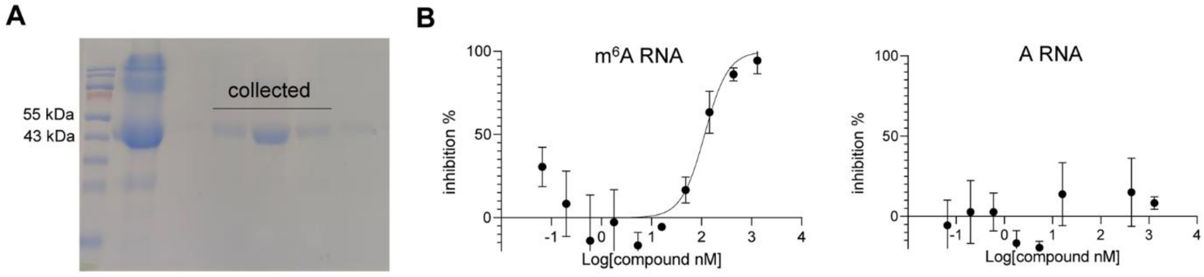

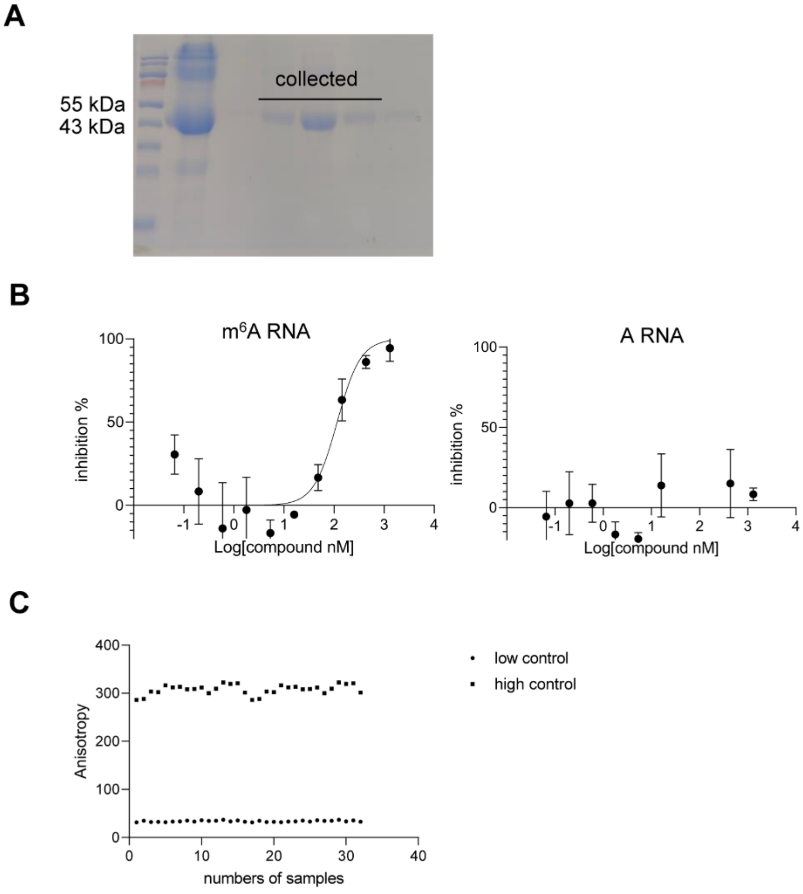

Figure S1. The fluorescence polarization (FP) assay of YTHDF2 binding to the m^{6}A RNA substrate. (A)The purification of YTHDF2 (residues 383-553), molecular weight: 47.7 kDa. (B) The m^{6}A RNA showed an IC_{50} of 115.2 nM in FP (left). In comparison, the unmethylated RNA substrate (A RNA) was inactive (right). m^{6}A RNA: 2 nM; YTHDF2: 100 nM (C) Z-factor of the FP assay: 0.87.

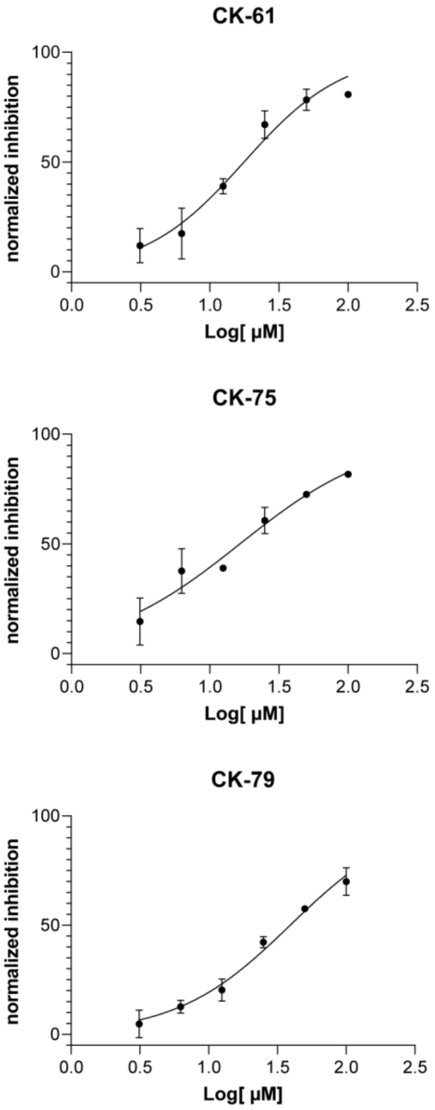

Figure S2. The inhibitory activities of the most active compounds CK-61, CK-75, and CK-79 tested in the FP.

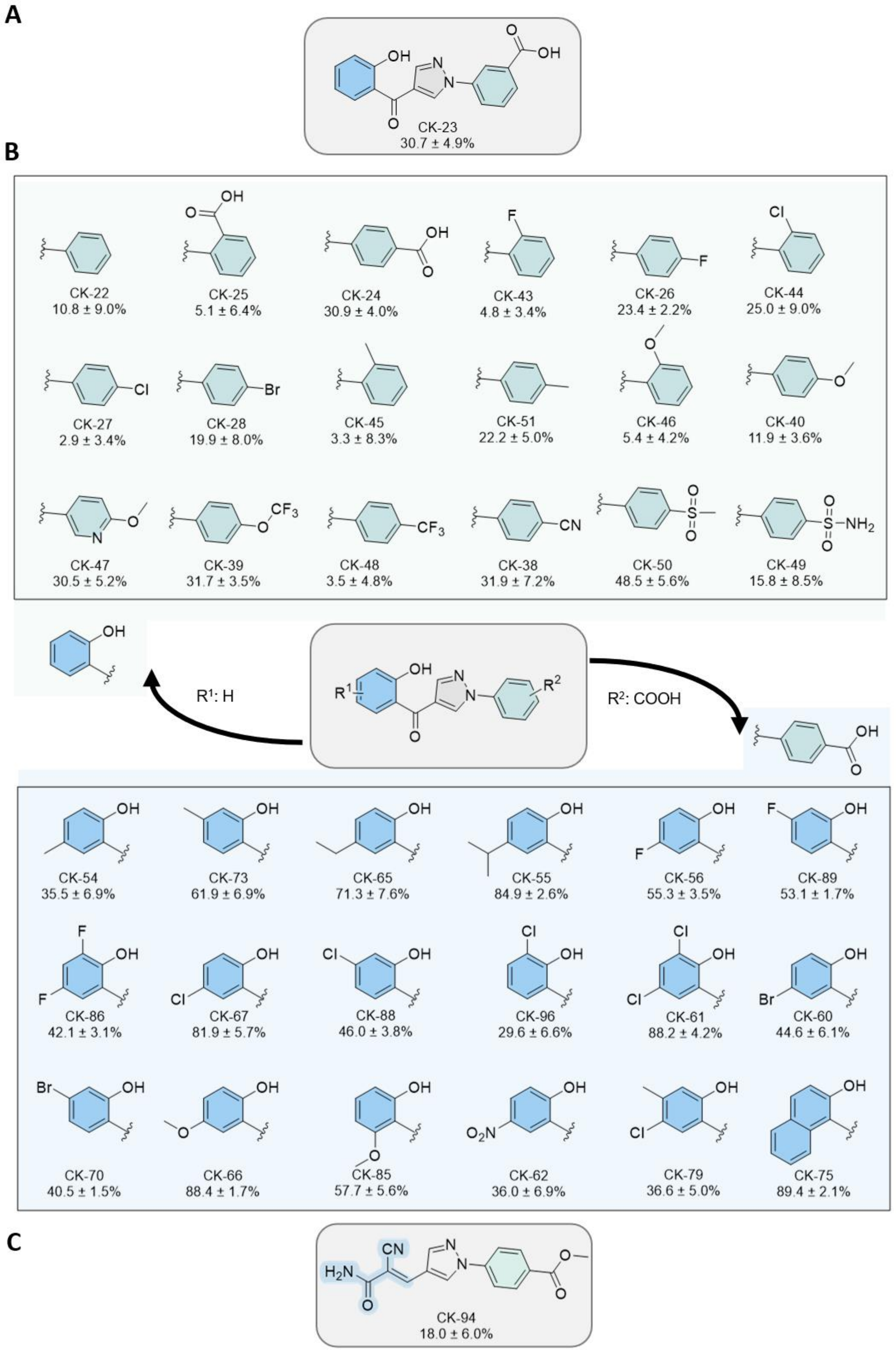
A
CK-23
30.7 ± 4.9%
B
CK-22
10.8 ± 9.0%
CK-25
5.1 ± 6.4%
CK-24
30.9 ± 4.0%
CK-43
4.8 ± 3.4%
CK-26
23.4 ± 2.2%
CK-44
25.0 ± 9.0%
CK-27
2.9 ± 3.4%
CK-28
19.9 ± 8.0%
CK-45
3.3 ± 8.3%
CK-51
22.2 ± 5.0%
CK-46
5.4 ± 4.2%
CK-40
11.9 ± 3.6%
CK-47
30.5 ± 5.2%
CK-39
31.7 ± 3.5%
CK-48
3.5 ± 4.8%
CK-38
31.9 ± 7.2%
CK-50
48.5 ± 5.6%
CK-49
15.8 ± 8.5%
R1: H
R2: COOH
CK-54
35.5 ± 6.9%
CK-73
61.9 ± 6.9%
CK-65
71.3 ± 7.6%
CK-55
84.9 ± 2.6%
CK-56
55.3 ± 3.5%
CK-89
53.1 ± 1.7%
CK-86
42.1 ± 3.1%
CK-67
81.9 ± 5.7%
CK-88
46.0 ± 3.8%
CK-96
29.6 ± 6.6%
CK-61
88.2 ± 4.2%
CK-60
44.6 ± 6.1%
CK-70
40.5 ± 1.5%
CK-66
88.4 ± 1.7%
CK-85
57.7 ± 5.6%
CK-62
36.0 ± 6.9%
CK-79
36.6 ± 5.0%
CK-75
89.4 ± 2.1%
C
CK-94
18.0 ± 6.0%

Figure S3. The inhibitory activities of the phenylpyrazoles (at 100 μM) against YTHDF2 binders in the amplified luminescent proximity homogeneous assay (AlphaScreen). (A) The inhibition against YTHDF2 by CK-23. (B) The inhibition against YTHDF2 by the 36 in-housed synthesized phenylpyrazoles featuring a 2-hydroxybenzoyl moiety R1 (blue) and a 4-carboxyphenyl moiety R2 (green). (C) The inhibition against YTHDF2 by the methylpyrazolylbenzoate CK-94. Data represent average values ± s.d.

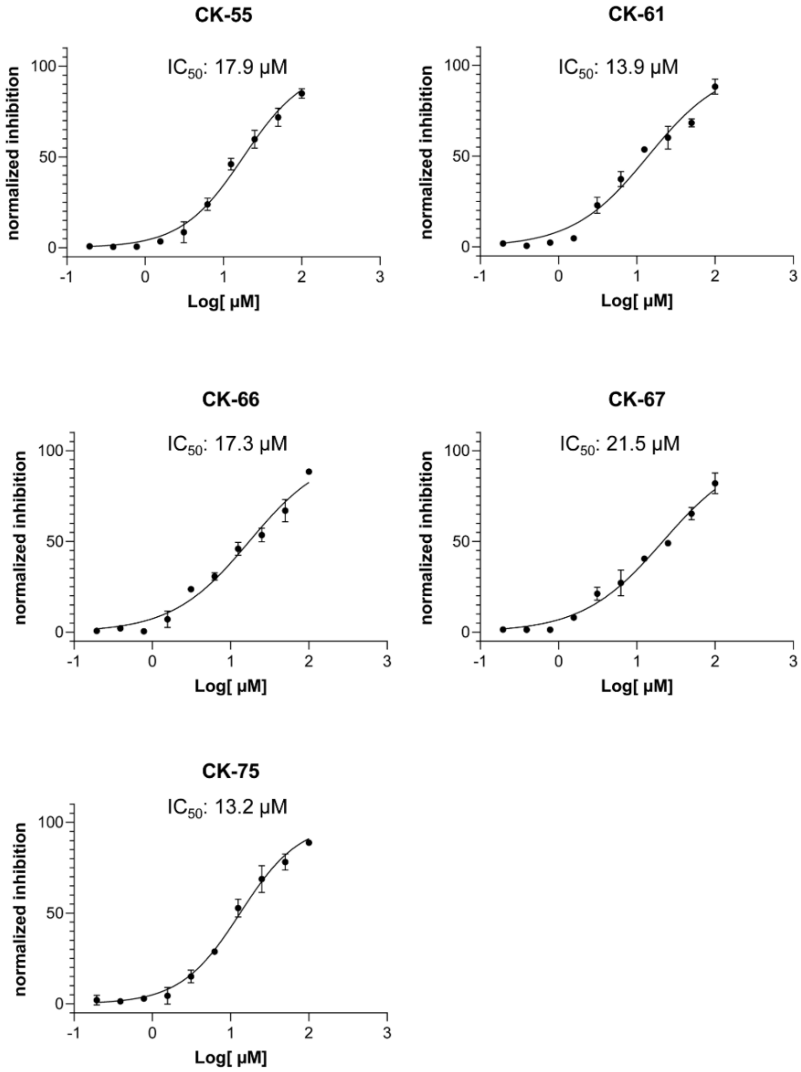

Figure S4. The inhibitory activities of the most active compounds CK-55, CK-61, CK-66, CK-67, and CK-75 tested in the AlphaScreen.

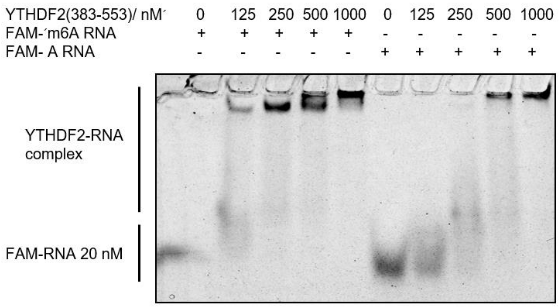

Figure S5. The EMSA using (m6)A RNA or unmethylated RNA substrate (A RNA) and YTHDF2 (residues 383-553) of varied concentrations.

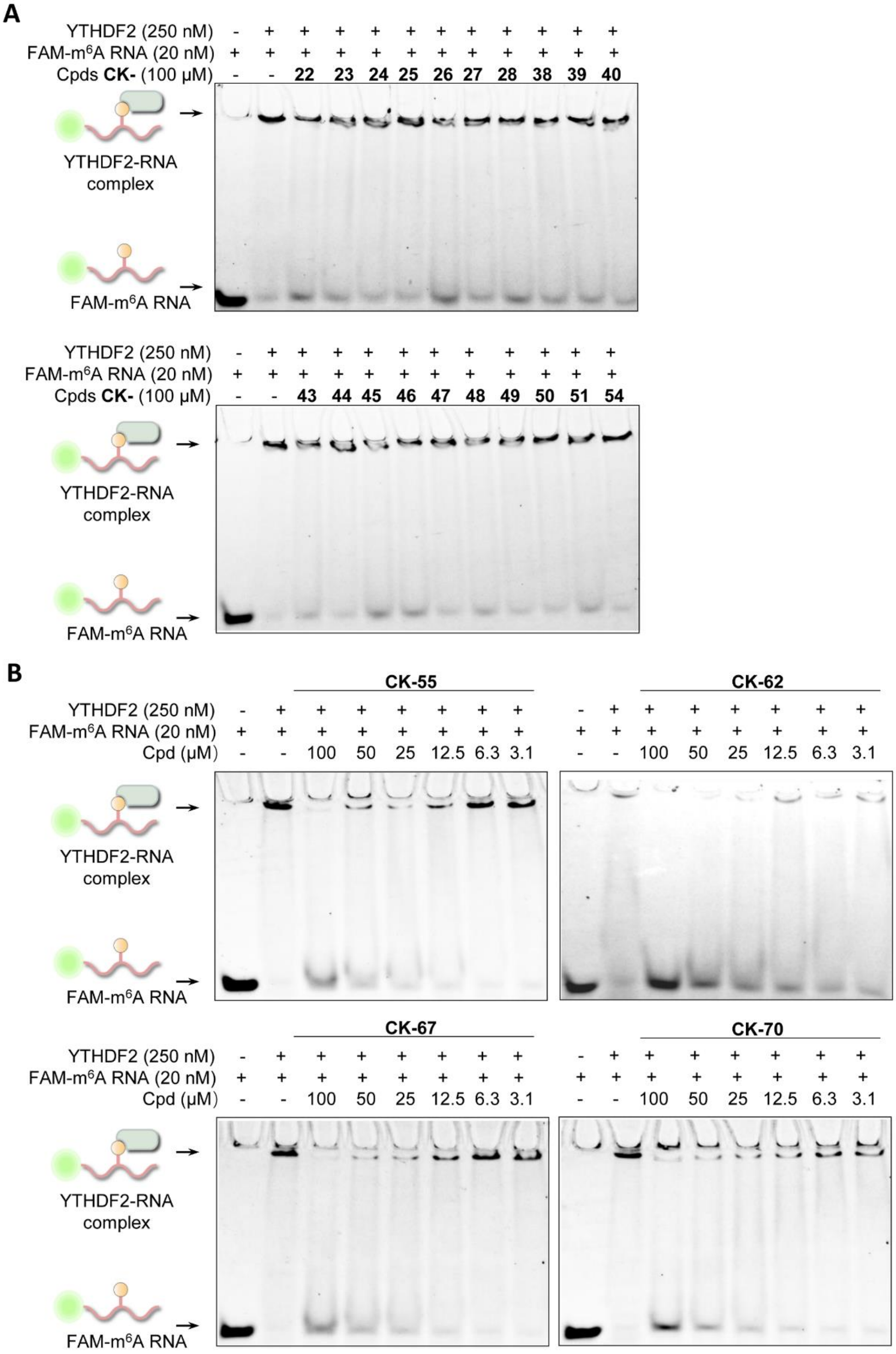
A
YTHDF2 (250 nM) - + + + + + + + + + + +
FAM-m6A RNA (20 nM) + + + + + + + + + + + +
Cpds CK- (100 µM) - - 22 23 24 25 26 27 28 38 39 40
YTHDF2-RNA complex
FAM-m6A RNA
YTHDF2 (250 nM) - + + + + + + + + + + +
FAM-m6A RNA (20 nM) + + + + + + + + + + + +
Cpds CK- (100 µM) - - 43 44 45 46 47 48 49 50 51 54
YTHDF2-RNA complex
FAM-m6A RNA
B
CK-55
CK-62
YTHDF2 (250 nM) - + + + + + + +
FAM-m6A RNA (20 nM) + + + + + + + +
Cpd (µM) - - 100 50 25 12.5 6.3 3.1
YTHDF2-RNA complex
FAM-m6A RNA
CK-67
CK-70
YTHDF2 (250 nM) - + + + + + + +
FAM-m6A RNA (20 nM) + + + + + + + +
Cpd (µM) - - 100 50 25 12.5 6.3 3.1
YTHDF2-RNA complex
FAM-m6A RNA

Figure S6. Testing of the phenylpyrazoles in EMSA. (A) 20 compounds (CK-022 to CK-054) were tested at 100 µM. Few compounds inhibit FAM-labelled (m6)A RNA binding to YTHDF2. (B) Compounds CK-55, CK-62, CK-67 and CK-70 dose-dependently inhibited the formation of the YTHDF2–mRNA complex. The EMSA was performed using 20 nM FAM-labelled m^6A mRNA with or without 250 nM YTHDF2. Electrophoresis of the FAM-labelled fragment (probe) alone is shown at the first left column of each panel.

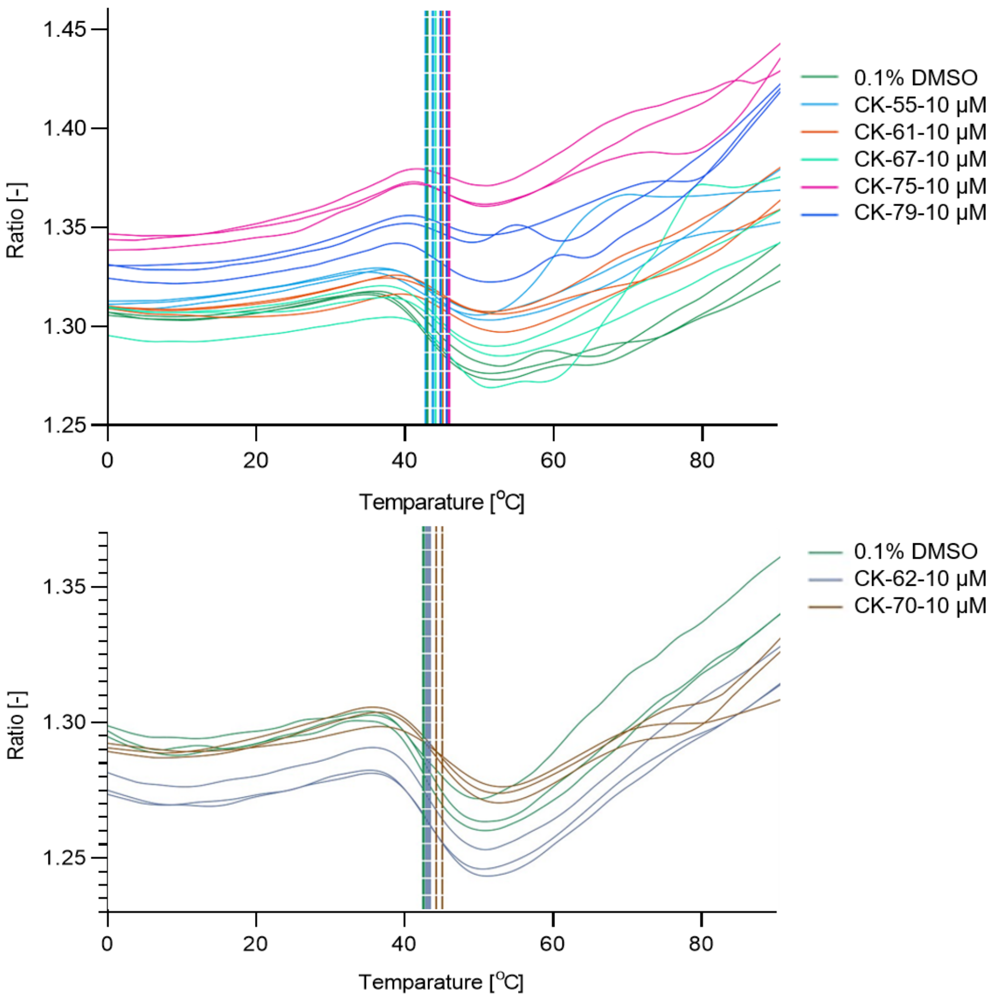

Figure S7. NanoDSF unfolding curves for YTHDF2. The corresponding first derivative of tryptophan fluorescence at 330 nm is plotted. Inflection points (equivalent to the *T*m) are shown as vertical lines.

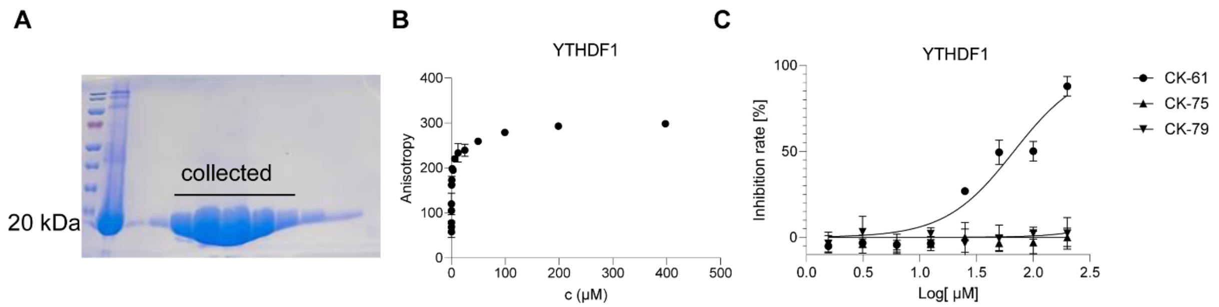

Figure S8. The FP assay of YTHDF1 binding to the m^{6}A RNA substrate. (A)The purification of YTHDF1 (residues 375-552), molecular weight: 21.9 kDa. (B) The anisotropy values using different concentrations of YTHDF1 with the m^{6}A RNA probe (4 nM), which was used to decide the appropriate YTHDF1 concentration for the FP (0.5 μM of YTHDF1, Z-factor: 0.65). (C) The inhibitory activities of CK-61, CK-75 and CK-79 against YTHDF1 in FP (CK-61, IC_{50}: 67.5 μM; CK-75: inactive; CK-79: inactive).

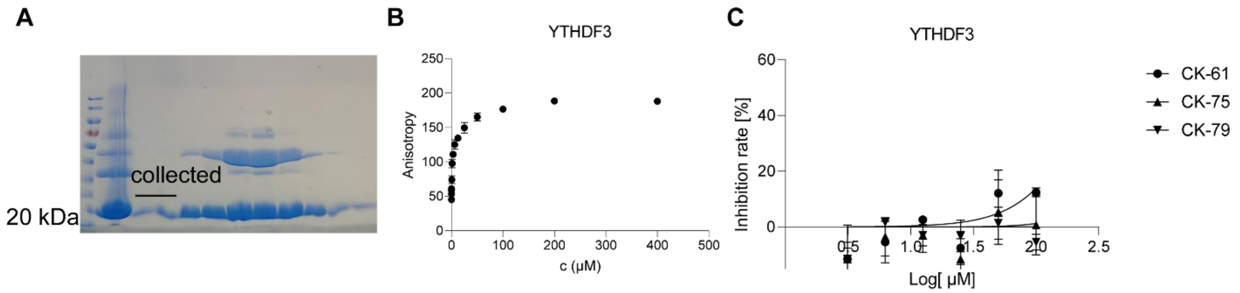

Figure S9. The FP assay of YTHDF3 binding to the m^{6}A RNA substrate. (A) The purification of YTHDF3 (residues 391-585), molecular weight: 24.1 kDa. (B) The anisotropy values using different concentrations of YTHDF3 with the m^{6}A RNA probe (4 nM), which was used to decide the appropriate YTHDF1 concentration for the FP (2.0 μM of YTHDF3, Z-factor: 0.55). (C) The inhibitory activities of CK-61, CK-75 and CK-79 against YTHDF3 in FP (CK-61, 12.4% inhibition under 100 μM; CK-75: inactive; CK-79: inactive).

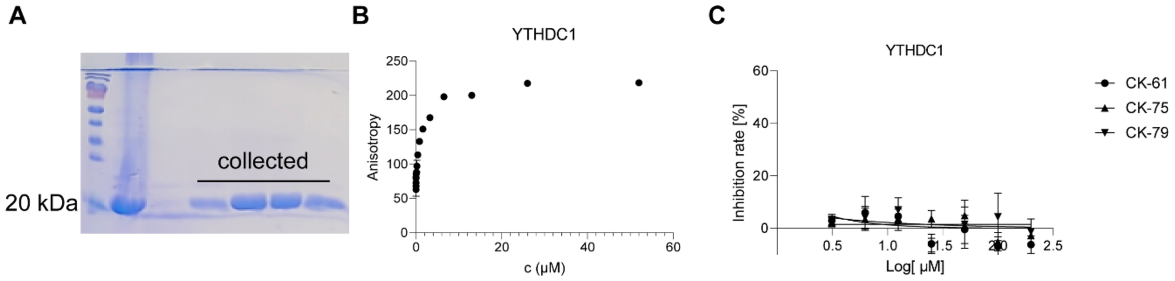

Figure S10. The FP assay of YTHDC1 binding to the m^{6}A RNA substrate. (A) The purification of YTHDC1 (residues 345-509), molecular weight: 19.9 kDa. (B) The anisotropy values using different concentrations of YTHDC1 with the m^{6}A RNA probe (4 nM), which was used to decide the appropriate YTHDF1 concentration for the FP (2.0 μM of YTHDC1, Z-factor: 0.54). (C) CK-61, CK-75 and CK-79 did not show detectable inhibitory activity against YTHDC1 in FP.

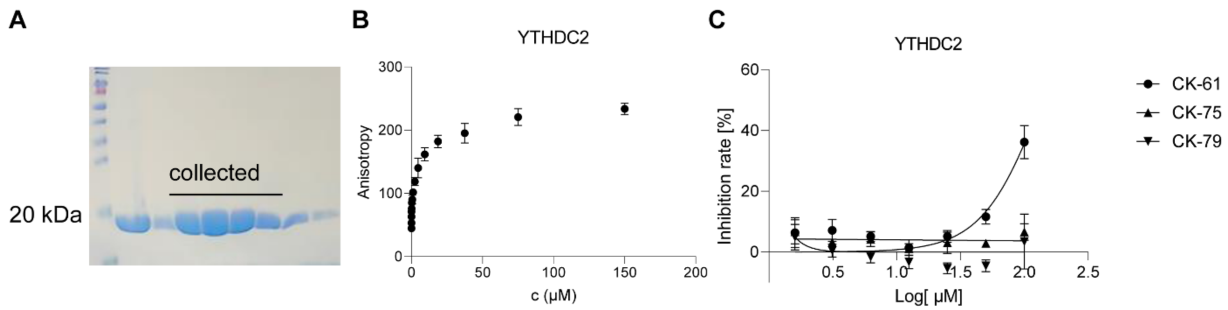

Figure S11. The FP assay of YTHDC2 binding to the m^{6}A RNA substrate. (A)The purification of YTHDC2 (residues 1285-1424), molecular weight: 17.4 kDa. (B) The anisotropy values using different concentrations of YTHDC2 with the m^{6}A RNA probe (4 nM), which was used to decide the appropriate YTHDC2 concentration for the FP (5.0 μM of YTHDC2, Z-factor: 0.83). (C) The inhibitory activities of CK-61, CK-75 and CK-79 against YTHDC2 in FP (CK-61, 36.1% inhibition under 100 μM; CK-75: inactive; CK-79: inactive).

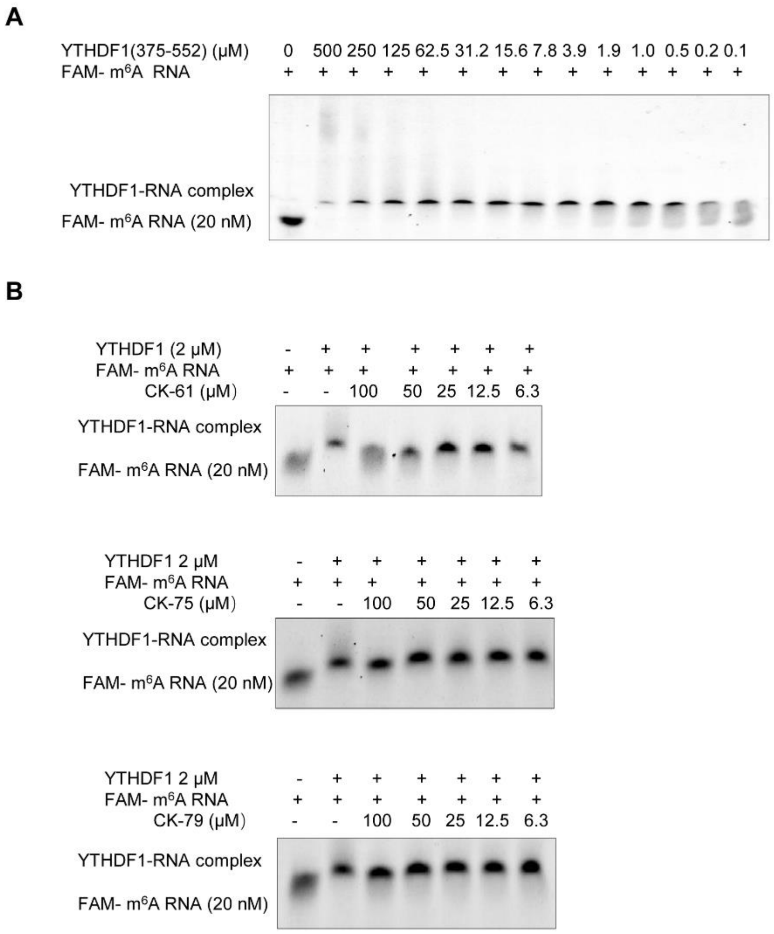

Figure S12. The EMSA of YTHDF1 binding to the m^{6}A RNA substrate. (A) The YTHDF1–RNA complex formation in EMSA using m^{6}A RNA (20 nM) and different concentrations of YTHDF1. (B) The inhibitory activities of CK-61, CK-75 and CK-79 against YTHDF1 in EMSA, which showed consistent results with that of FP.

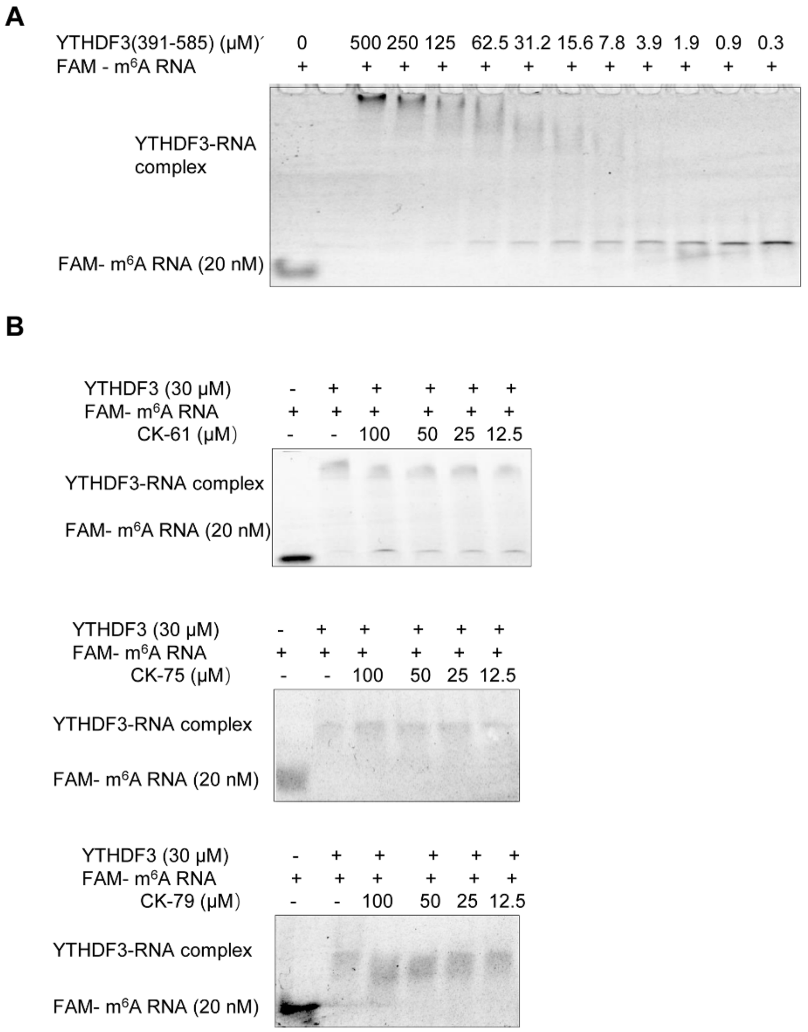

Figure S13. The EMSA of YTHDF3 binding to the m^{6}A RNA substrate. (A) The YTHDF3–RNA complex formation in EMSA using m^{6}A RNA (20 nM) and different concentrations of YTHDF1. (B) CK-61, CK-75 and CK-79 did not show detectable inhibitory activity against YTHDF3 in EMSA.

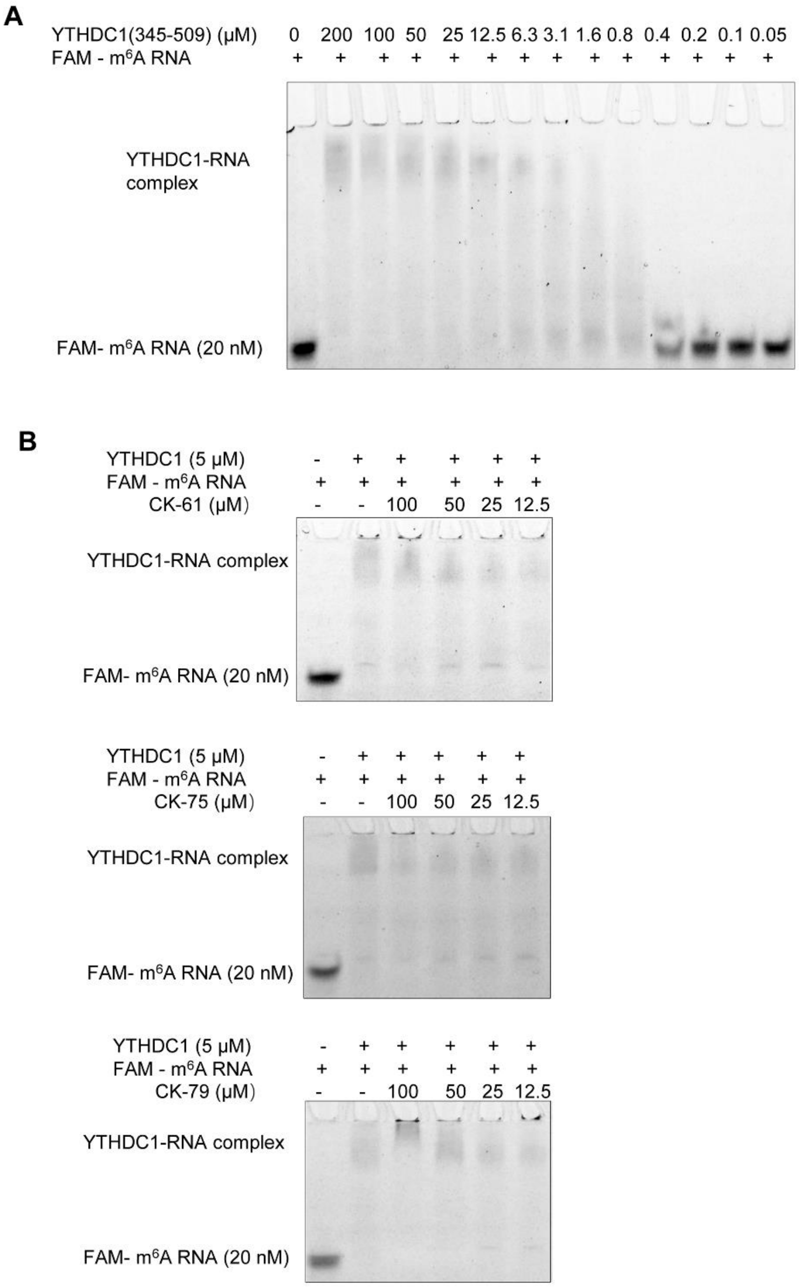

Figure S14. The EMSA of YTHDC1 binding to the m^{6}A RNA substrate. (A) The YTHDC1–RNA complex formation in EMSA using m^{6}A RNA (20 nM) and different concentrations of YTHDC1. (B) CK-61, CK-75 and CK-79 did not show detectable inhibitory activity against YTHDC1 in EMSA.

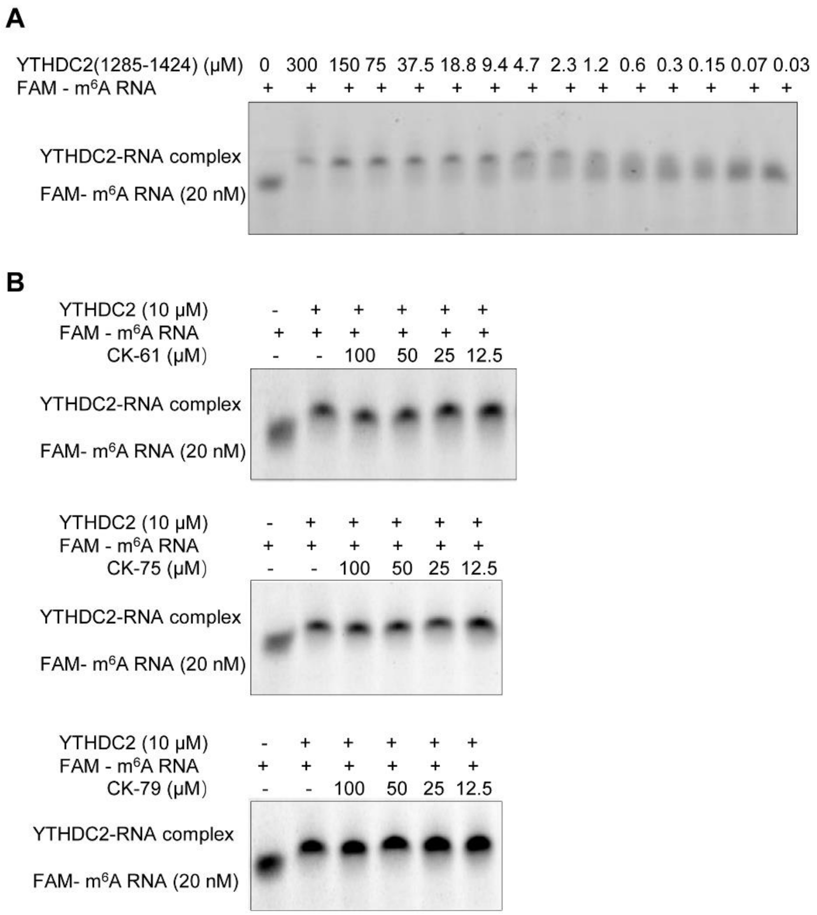

Figure S15. EMSA using m^{6}A RNA and YTHDC2 protein of varied concentrations as indicated. (A) 20 nM of m^{6}A RNA was used and 10 µM of YTHDC2 was chosen for EMSA. (B) CK-61, CK-75 and CK-79 did not show detectable inhibitory activity against YTHDC2 in EMSA.

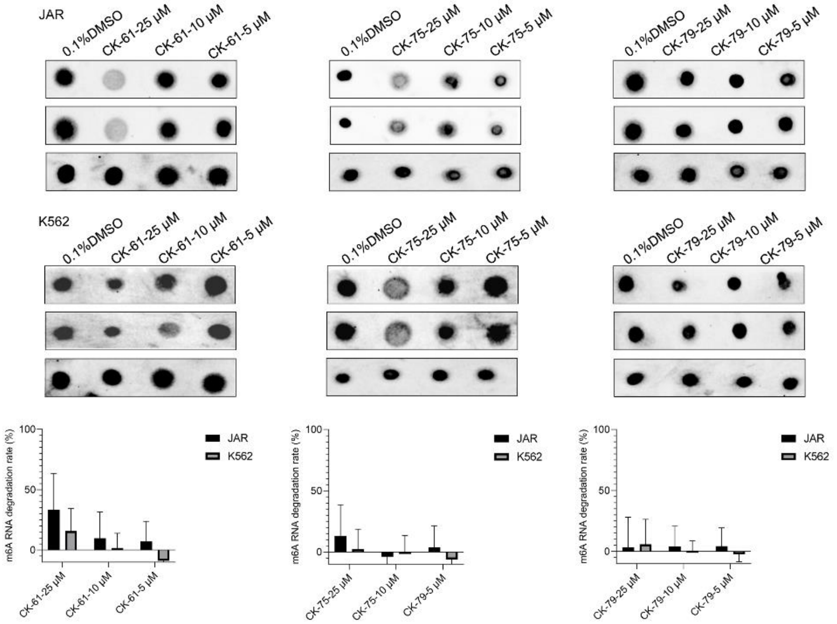

Figure S16. Dot blot assay. Histogram of m^{6}A RNA level upon CK-75 incubation in K562 (mean ± SD, n = 3 biological replicates) and JAR (mean ± SD, n = 3 biological replicates) cells, normalized to DMSO-treated samples for each cell line.

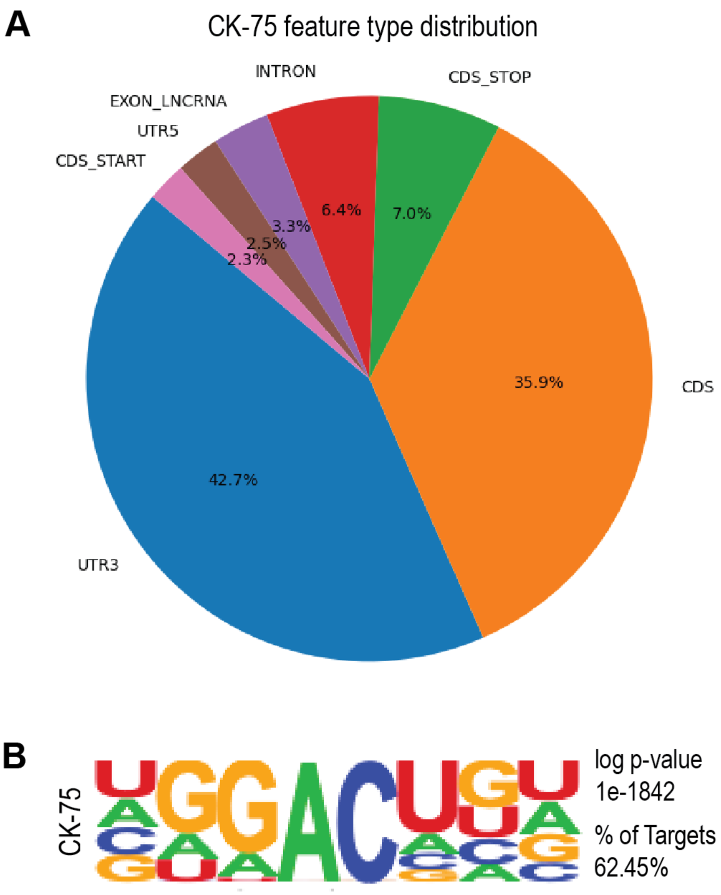

Figure S17. Transcriptome-wide evaluation using eCLIP. (A) Pie charts showing the distribution of the windows in the CK-75-treated cells according to the region in the RNA to which they map. (B) HOMER analysis of enriched motifs in eCLIP peaks in the K562 cells treated with CK-75 showing the DRACH motif.

Table S1. The IC_{50} values for the top-listed hits from the screening of an in-house small-molecule library using the fluorescence polarization assay (FP).

Hit Nr.	IC_{50}, n1	IC_{50}, n2	IC_{50}, n3	IC_{50}, n4	AVERAGE IC_{50}±SD (µM)
1	15.94	20.66	20.81	21.00	19.60± 2.45
2	22.56	25.09	26.01	25.78	24.86± 1.58
3	6.59	9.11	10.10	10.66	9.12± 1.80
4	11.53	14.22	14.94	15.24	13.98± 1.69
5	15.58	19.87	19.46	19.89	18.70± 2.09
6	20.65	23.70	24.30	23.82	23.12± 1.67
7	5.06	5.55	5.89	5.64	5.54± 0.35
8	11.10	13.57	14.57	14.65	13.47± 1.66
9	4.88	6.20	7.51	6.10	6.17± 1.07
10	4.71	5.60	5.75	5.48	5.39± 0.46
11	7.48	9.30	10.49	9.71	9.24± 1.28
12	7.78	9.30	9.75	9.10	8.98± 0.85
13	20.81	27.47	25.45	24.85	24.65± 2.79
14	6.68	7.66	7.06	9.58	7.75± 1.29
15	10.53	13.69	14.33	13.78	13.08± 1.73
16	7.88	8.99	8.54	8.63	8.51± 0.46
17	6.17	7.78	7.71	6.81	7.12± 0.77
18	12.70	16.74	14.07	12.36	13.97± 1.99
19	20.67	23.74	22.89	19.37	21.67± 2.01
20	8.57	12.76	12.62	10.27	11.05± 2.01

21	26.66	> 30 µM	27.22	26.08	26.65± 0.57
22 (CK-23)	14.36	14.93	13.52	11.76	13.64± 1.38
23	10.45	12.03	12.45	12.85	11.95± 1.05
24	16.78	18.34	19.52	21.56	19.05± 2.01
25	27.72	> 30 µM	> 30 µM	> 30 µM	-
26	8.71	10.29	10.55	10.71	10.06± 0.92
27	24.78	28.58	29.75	> 30 µM	27.70± 2.60
28	9.55	11.36	10.83	11.63	10.84± 0.92
29	7.72	8.75	9.33	9.85	8.91± 0.91
30	17.04	21.14	20.50	22.77	20.36± 2.41
31	14.05	17.13	16.05	17.52	16.19± 1.55
32	9.63	11.34	12.48	14.01	11.87± 1.85
33	21.69	25.49	26.28	27.39	25.21± 2.47
34	11.40	14.04	14.17	14.29	13.48± 1.39
35	14.88	21.23	23.24	16.69	19.01± 3.88
36	8.47	10.92	11.20	10.12	10.18± 1.23
37	26.73	> 30 µM	> 30 µM	26.31	26.52± 0.30
38	2.99	3.94	6.25	5.31	4.62± 1.44
39	4.51	5.39	5.42	4.49	4.95± 0.52
40	13.83	14.85	18.88	17.11	16.17± 2.27
41	13.42	16.09	14.86	13.03	14.35± 1.40
42	19.41	22.35	23.85	18.92	21.13± 2.36
43	> 30 µM	> 30 µM	> 30 µM	28.26	-
44	21.93	21.89	17.83	17.42	19.77± 2.48

Table S2. The cytotoxicity of the phenylpyrazole inhibitors against human cancer cells (tested in the MTT assay)

Compounds	Cytotoxicity against cancer cells (IC_{50}, μM)[a]		
	JAR	K562	HCT116
CK-22	48.1± 10.2	32.1± 12.2	43.5± 12.3
CK-23	45.0± 4.6	21.5± 8.2	12.9± 2.5
CK-24	>50	43.2± 15.7	>50
CK-25	>50	44.3± 13.1	>50
CK-26	47.2± 15.5	23.4± 9.4	29.4± 10.4
CK-27	>50	>50	>50
CK-28	>50	>50	>50
CK-38	>50	43.2± 11.2	>50
CK-39	46.2± 20.8	19.8± 14.3	26.4± 10.3
CK-40	44.6± 12.3	33.2± 5.8	25.5± 1.8
CK-43	>50	46.2± 10.1	>50
CK-44	44.7± 14.4	32.3± 8.8	18.5± 14.1
CK-45	43.2± 15.5	21.9± 7.6	26.0± 16.8
CK-46	>50	23.8± 4.7	49.4± 22.1
CK-47	>50	>50	>50
CK-48	37.0± 12.7	22.8± 2.5	43.1± 8.6
CK-49	34.6± 5.9	20.1± 12.3	29.0± 11.4
CK-50	>50	48.2± 11.1	>50
CK-51	32.9± 14.2	20.2± 15.4	18.5± 9.4
CK-54	>50	>50	>50
CK-55	44.4± 13.5	32.4± 22.1	49.5± 8.5
CK-56	41.2± 14.1	23.9± 7.5	19.3± 2.5
CK-60	37.1± 11.4	16.4± 8.2	12.5± 6.4
CK-61	>50	>50	>50
CK-62	42.6± 18.5	>50	>50
CK-65	>50	>50	>50
CK-66	>50	>50	>50
CK-67	>50	34.9± 13.4	48.5± 4.9
CK-70	>50	42.1± 14.6	36.4± 2.8
CK-73	>50	>50	>50
CK-75	47.5± 16.4	29.7± 5.5	33.3± 9.8
CK-79	31.6	29.4± 16.7	33.6± 14.7
CK-85	>50	>50	>50
CK-86	>50	>50	>50
CK-88	25.0± 3.6	43.2± 10.4	>50
CK-89	>50	43.8± 22.7	32.8± 7.5
CK-95	7.8± 8.1	14.4± 8.1	1.7± 5.0
CK-96	40.23± 12.7	25.4± 7.8	20.6± 8.1

[a] Tested in triplicates, with an incubation time of 72 h.

Table S3. Antiproliferative activity of the phenylpyrazole inhibitors against human cancer cells (tested in the CCK-8 kit assay)

Compounds	Anti-proliferation activity against cancer cells (IC_{50}, μM)[a]		
	JAR	K562	HCT116
CK-22	27.6± 2.8	28.3± 5.4	>50
CK-23	>50	>50	>50
CK-24	>50	>50	>50
CK-25	>50	>50	>50
CK-26	31.2± 6.4	21.5± 1.9	43.2± 5.3
CK-27	>50	44.8± 8.7	45.6± 2.5
CK-28	>50	>50	>50
CK-38	>50	>50	>50
CK-39	31.2± 3.5	25.6± 2.3	38.9± 12.1
CK-40	>50	39.2± 6.1	>50
CK-43	>50	>50	>50
CK-44	41.2± 8.5	34.9± 3.5	>50
CK-45	42.8± 1.2	33.1± 5.7	>50
CK-46	>50	42.9± 6.2	>50
CK-47	>50	>50	>50
CK-48	>50	>50	>50
CK-49	>50	>50	>50
CK-50	>50	>50	>50
CK-51	>50	30.1± 6.3	>50
CK-54	>50	>50	>50
CK-55	>50	31.2± 8.9	>50
CK-56	>50	>50	>50
CK-60	>50	>50	>50
CK-61	>50	>50	>50
CK-62	>50	>50	>50
CK-65	45.6± 3.9	43.9± 12.0	>50
CK-66	47.3± 6.3	38.8± 3.7	47.2± 8.7
CK-67	38.5± 9.2	33.4± 3.3	41.6± 8.2
CK-70	48.5± 3.9	45.5± 6.2	>50
CK-73	>50	>50	>50
CK-75	43.4± 3.4	38.5± 3.5	36.8± 1.8
CK-79	32.1± 6.3	28.1± 1.6	44.0± 6.8
CK-85	46.2± 2.6	>50	>50
CK-86	>50	>50	>50
CK-88	39.0± 3.2	48.2± 6.3	>50
CK-89	>50	>50	>50
CK-95	8.5± 1.3	9.3± 3.2	20.3± 3.6
CK-96	>50	>50	`>50
DC-Y13-27	33.3± 5.6	24.5± 3.7	5.8± 0.9

[a] Tested in triplicates, with an incubation time of 72 h.

General Chemistry Information

The commercially available reagents were used without further purification unless noted otherwise. The solvents were obtained from VWR, Fischer Scientific and Acros and used without further treatment. Reagents that are sensitive to oxygen or moisture sensitive and dry solvents were transferred using cannulas and syringes under inert gas.

Thin layer chromatography (TLC) was performed using silica coated aluminum plates (Merck 60 F_{254}). The reaction controls were visualized under UV radiation (254 nm) or using potassium permanganate stain solution (1.5 g $KMnO_4$, 10 g K_2CO_3, 1.25 mL NaOH solution (10% in H_2O) and 200 mL H_2O).

Analytical UHPLC-MS and LC-MS were performed on the system “Agilent 1260 II Infinity” and the mass detectors “Zorbax Eclipse C18 Rapid Resolution” (UHPLC, 2.1x50 mm 1.8 µm) and “InfinityLab Poroshell 120 EC-C18” (LC-MS, 2.1x150, 2.7 µm). Different gradients were applied using different mixtures of Acetonitrile (+0.1% TFA) and water (+0.1% TFA).

High-resolution mass spectrometry (HRMS) was performed on an LTQ Orbitrap mass spectrometer coupled to an Accela HPLC-System (HPLC column: Hypersyl GOLD, 50 mm x 1 mm, particle size 1.9 µm, ionization method: electron spray ionization (ESI)).

NMR spectra were recorded on Bruker AV Avance III HD (NanoBay), Aglient Technologies DD2, Bruker AV 500 Avance III HD (Prodigy), Bruker AV 600 Avance III HD (CryoProbe) or Bruker AV 700 Avance III HD (CryoProbe) spectrometers. The data is displayed in parts per million (ppm) including a reference for the used deuterated solvent ($CDCl_3$: 7.26 ppm, 77.16 ppm; DMSO-d_6: 2.50 ppm, 39.52 ppm). The integration value,

coupling constants and the multiplicity (s = singlet, d = doublet, t = triplet, dd = double doublet, and m = multiplet) is reported in Hz.

Synthetic Procedures and Compound Characterization

General Procedure A

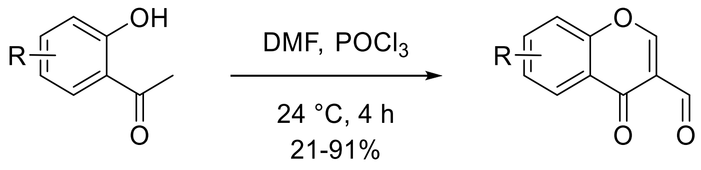

Scheme S1: Synthetic route for the synthesis of substituted 3-formylchromones.[1]

Substituted 2-hydroxyacetophenone (1 eq.) was dissolved in anhydrous DMF (6 eq.) under nitrogen atmosphere. Phosphorus(V) oxychloride (2 eq.) was added dropwise over 10 min. The reaction mixture was stirred at ambient temperature for 4 h, followed by the addition of ice-cold water (10 mL). The precipitate was filtered off, washed with ice cold water and dried. The residue was either used directly or purified using silica column chromatography.[1]

General procedure B

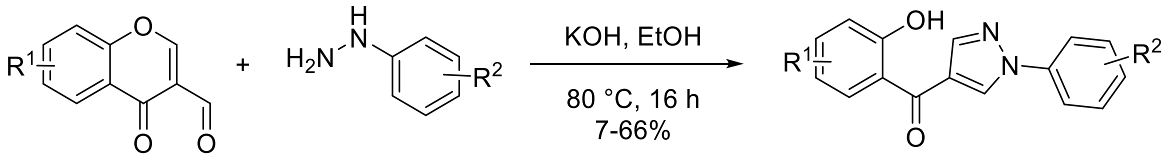

Scheme S2: Synthetic route for the synthesis of substituted pyrazoles.[2]

The corresponding formylchromone (1 eq.) and arylhydrazine (1 eq.) were dissolved in ethanol (1.5 mL) and KOH (0.5 mL, 4 M, 4 eq.) was added. The reaction mixture was refluxed overnight. After cooling to ambient temperature, HCl (1 M) was added until pH <

1. The precipitate was filtered off, washed with a small amount of water and ethanol and dried. The precipitate was used directly or purified by silica column chromatography.[2]

7-Methyl-4-oxo-4H-chromene-3-carbaldehyde (CK-53) synthesized according to the general procedure A, obtained as a red solid (94.30 mg, yield 70%); no further purification; ^{1}H NMR (400 MHz, DMSO-d_6) δ 10.10 (s, 1H), 8.86 (s, 1H), 8.01 (d, *J* = 8.1 Hz, 1H), 7.56 (s, 1H), 7.39 (dd, *J* = 8.2, 1.6 Hz, 1H), 2.47 (s, 4H); ^{13}C NMR (176 MHz, Chloroform-*d*) δ 187.7, 174.8, 159.4, 155.3, 145.5, 127.0, 124.9, 121.9, 119.2, 117.3, 20.8. (LR)MS (ESI) for $C_{11}H_9O_3$ m/z 189, found m/z 189, 99% [M+H]$^+$.

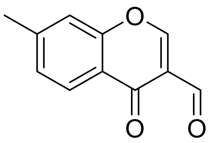

1-Oxo-1H-benzo[*f*]chromene-2-carbaldehyde (CK-57) synthesized according to the general procedure A, obtained as a pale yellow solid (104.20 mg, yield 87%); no further purification; ^{1}H NMR (400 MHz, Chloroform-*d*) δ 10.52 (s, 1H), 10.00 (ddq, *J* = 8.7, 1.3, 0.6 Hz, 1H), 8.56 (s, 1H), 8.17 (dd, *J* = 9.0, 0.6 Hz, 1H), 7.95 (ddd, *J* = 8.1, 1.4, 0.7 Hz, 1H), 7.82 (ddd, *J* = 8.6, 7.0, 1.5 Hz, 1H), 7.69 (dddd, *J* = 8.1, 6.9, 1.2, 0.5 Hz, 1H), 7.56 (d, *J* = 9.1 Hz, 1H); ^{13}C NMR (176 MHz, Chloroform-*d*) δ 188.3, 176.8, 157.0, 156.6, 135.6, 130.0, 129.3, 128.8, 127.4, 126.4, 126.0, 121.3, 118.0, 116.3. (LR)MS (ESI) for $C_{14}H_9O_3$ m/z 225, found m/z 225, 99% [M+H]$^+$.

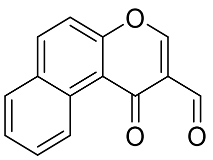

7-Chloro-4-oxo-4H-chromene-3-carbaldehyde (CK-71) synthesized according to the general procedure A, obtained as a light red solid (159.30 mg, yield 65%); no further purification; ^{1}H NMR (400 MHz, Chloroform-*d*) δ 10.30 (s, 1H), 8.44 (s, 1H), 8.17 (d, *J* = 8.5 Hz, 1H), 7.50 (d, *J* = 1.9 Hz, 1H), 7.41 (dd, *J* = 8.5, 1.9 Hz, 1H); ^{13}C NMR (176 MHz, Chloroform-*d*) δ 187.1, 174.1, 159.5, 155.2, 140.0, 126.4 (2C), 122.8, 119.5, 117.7; (LR)MS (ESI) for $C_{10}H_6ClO_3$ m/z 209, found m/z 209, 99% $[M+H]^+$.

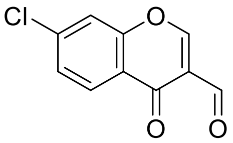

7-Fluoro-4-oxo-4H-chromene-3-carbaldehyde (CK-72) synthesized according to the general procedure A, obtained as an orange solid (110.80 mg, yield 44%); no further purification; ^{1}H NMR (400 MHz, Chloroform-*d*) δ 10.31 (s, 1H), 8.45 (s, 1H), 8.29 – 8.23 (m, 1H), 7.19 – 7.14 (m, 2H); ^{13}C NMR (176 MHz, Chloroform-*d*) δ 187.27, 174.01, 165.13 (d, $^{1}J_{C,F}$ = 257.8 Hz), 159.64, 156.17 (d, $^{3}J_{C,F}$ = 13.2 Hz), 127.77 (d, $^{3}J_{C,F}$ = 10.6 Hz), 121.10 (d, $^{4}J_{C,F}$ = 2.7 Hz), 119.40, 114.38 (d, $^{2}J_{C,F}$ = 22.7 Hz), 104.60 (d, $^{2}J_{C,F}$ = 25.8 Hz). ^{13}C NMR (176 MHz, Chloroform-*d*) δ 187.2, 174.0, 165.8, 164.3, 159.6, 127.7 (d, ^{3}J = 10.6 Hz), 121.1 (d, ^{4}J = 2.7 Hz), 119.4, 114.3 (d, ^{2}J = 22.7 Hz), 104.6 (d, ^{2}J = 25.8 Hz); (LR)MS (ESI) for $C_{10}H_6FO_3$ m/z 193, found m/z 193, 99% $[M+H]^+$.

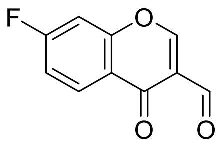

5-Methoxy-4-oxo-4H-chromene-3-carbaldehyde (CK-77) synthesized according to the general procedure A, obtained as an orange solid (112.30 mg, yield 91%); no further purification; ^{1}H NMR (400 MHz, Chloroform-*d*) δ 10.28 (s, 1H), 8.32 (s, 1H), 7.55 (t, *J* = 8.4 Hz, 1H), 7.01 (dd, *J* = 8.4, 0.9 Hz, 1H), 6.83 (dd, *J* = 8.4, 0.9 Hz, 1H), 3.95 (s, 3H); ^{13}C NMR (176 MHz, Chloroform-*d*) δ 188.1, 174.8, 159.3, 157.7, 157.0, 133.9, 120.2, 114.5, 109.4, 106.9, 55.5; (LR)MS (ESI) for $C_{11}H_9O_4$ m/z 205, found m/z 205, 99% $[M+H]^+$.

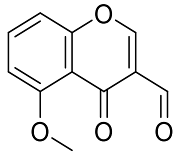

6,8-Difluoro-4-oxo-4H-chromene-3-carbaldehyde (CK-82) synthesized according to the general procedure A, obtained as a beige solid (86.00 mg, yield 70%); no further purification; ^{1}H NMR (400 MHz, Chloroform-*d*) δ 10.30 (s, 1H), 8.49 (s, 1H), 7.68 (ddd, *J* = 7.7, 3.0, 1.9 Hz, 1H), 7.26 (ddd, *J* = 9.8, 7.7, 3.0 Hz, 1H); ^{13}C NMR (176 MHz, Chloroform-*d*) δ 202.5 (t, $^{4}J_{C,F}$ = 2.7 Hz), 186.7, 173.1 (t, $^{4}J_{C,F}$ = 2.7 Hz), 159.1, 159.0 – 149.6 (m), 140.7 (dd, $^{3,4}J_{C,F}$ = 11.6, 3.1 Hz), 126.7 (d, $^{3}J_{C,F}$ = 8.4 Hz), 118.9, 109.3 (dd, ^{2}J = 28.6, 20.1 Hz), 105.6 (dd, ^{2}J = 23.8 Hz). (LR)MS (ESI) for $C_{10}H_5F_2O_3$ m/z 211, found m/z 211, 99% $[M+H]^+$.

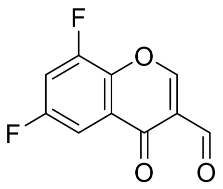

3-(4-(2-Hydroxybenzoyl)-1H-pyrazol-1-yl)benzoic acid (CK-23) synthesized according to the general procedure B, obtained as a yellow solid (29.50 mg, yield 33%); no further purification; ^{1}H NMR (500 MHz, DMSO-d_6) δ 13.32 (s, 1H), 11.07 (s, 1H), 9.21 (s, 1H), 8.49 (t, J = 2.0 Hz, 1H), 8.27 – 8.21 (m, 2H), 7.96 (dt, J = 7.7, 1.3 Hz, 1H), 7.78 (dd, J = 7.8, 1.7 Hz, 1H), 7.68 (t, J = 7.9 Hz, 1H), 7.52 (ddd, J = 8.7, 7.2, 1.7 Hz, 1H), 7.06 – 6.97 (m, 2H); ^{13}C NMR (126 MHz, DMSO-d^6) δ 190.6, 167.0, 159.0, 143.2, 139.5, 134.8, 132.7, 132.5, 131.2, 130.5, 128.5, 124.5, 123.9, 123.8, 120.3, 119.7, 117.7; (LR)MS (ESI) for $C_{17}H_{12}N_2O_4$ m/z 308, found m/z 308, 99% [M+H]$^+$.

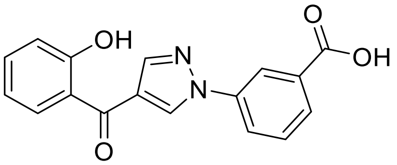

(2-Hydroxyphenyl)(1-phenyl-1H-pyrazol-4-yl)methanone (CK-22)[3] synthesized according to the general procedure B, obtained as a yellow solid (66.30 mg, yield 58%); no further purification; ^{1}H NMR (600 MHz, DMSO-d_6) δ 11.09 (s, 1H), 9.10 (s, 1H), 8.21 (s, 1H), 7.98 (d, J = 8.0 Hz, 2H), 7.77 (dd, J = 7.8, 1.7 Hz, 1H), 7.58 – 7.50 (m, 3H), 7.41 (t, J = 7.4 Hz, 1H), 7.05 – 6.97 (m, 2H); ^{13}C NMR (151 MHz, DMSO-d_6) δ 190.7, 159.0, 142.9, 139.3, 134.8, 132.1, 131.2, 130.0 (2C), 127.9, 124.3, 123.8, 119.8 (3C), 117.7; MS (HRMS-ESI): calculated for [M+H$^+$] $C_{16}H_{13}N_2O_2$ m/z 365.0972, found m/z 365.0966.

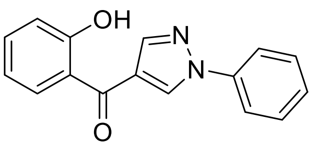

4-(4-(2-Hydroxybenzoyl)-1H-pyrazol-1-yl)benzoic acid (CK-24) synthesized according to the general procedure B, obtained as a yellow solid (29.60 mg, yield 33%); no further purification; ^{1}H NMR (600 MHz, DMSO-d_6) δ 13.11 (s, 1H), 11.02 (s, 1H), 9.21 (s, 1H), 8.26 (s, 1H), 8.15 – 8.06 (m, 4H), 7.75 (dd, J = 7.8, 1.8 Hz, 1H), 7.52 (ddd, J = 8.7, 7.3, 1.7 Hz, 1H), 7.05 – 6.97 (m, 2H); ^{13}C NMR (151 MHz, DMSO) δ 190.5, 167.0, 158.9, 143.5, 142.3, 134.8, 132.6, 131.3 (2C), 131.1, 124.8, 124.0, 119.8, 119.4 (2C), 117.7; MS (HRMS-ESI): calculated for [M+H$^+$] $C_{16}H_{13}N_2O_4$ m/z 309.0870, found m/z 309.0866.

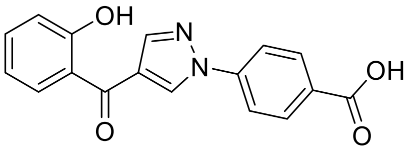

(1-(4-Fluorophenyl)-1H-pyrazol-4-yl)(2-hydroxyphenyl)methanone (CK-26)[4] synthesized according to the general procedure B, obtained as a yellow solid (51.90 mg, yield 64%); no further purification; ^{1}H NMR (400 MHz, Chloroform-d) δ 11.98 (s, 1H), 8.43 (d, J = 0.7 Hz, 1H), 8.17 (d, J = 0.7 Hz, 1H), 7.91 (dd, J = 8.0, 1.7 Hz, 1H), 7.77 – 7.69 (m, 2H), 7.53 (ddd, J = 8.7, 7.2, 1.7 Hz, 1H), 7.26 – 7.17 (m, 2H), 7.08 (dd, J = 8.4, 1.1 Hz, 1H), 6.98 (ddd, J = 8.2, 7.2, 1.2 Hz, 1H); ^{13}C NMR (176 MHz, DMSO-d_6) δ 190.6, 161.4 ($^{1}J_{C,F}$ = 244.4 Hz) , 159.0, 142.9, 135.9 (d, $^{4}J_{C,F}$ = 2.7 Hz), 134.8, 132.3, 131.2, 125.7 (d, $^{3}J_{C,F}$ = 8.7 Hz), 124.3, 123.8, 122.0 (d, $^{3}J_{C,F}$ = 8.6 Hz), 119.7, 117.7, 116.8 (2C)

(d, $^2J_{C,F}$ = 23.1 Hz).; MS (HRMS-ESI): calculated for [M+H$^+$] $C_{16}H_{12}N_2O_2$ m/z 283.0877, found m/z 283.0872.

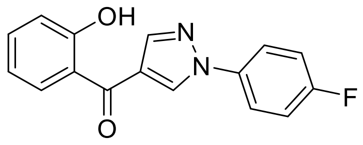

(1-(4-Chlorophenyl)-1H-pyrazol-4-yl)(2-hydroxyphenyl)methanone (CK-27) synthesized according to the general procedure B, obtained as a red solid (16.40 mg, yield 19%); no further purification; ^{1}H NMR (400 MHz, Chloroform-*d*) δ 11.97 (s, 1H), 8.46 (s, 0H), 8.18 (s, 0H), 7.90 (dd, *J* = 8.0, 1.7 Hz, 1H), 7.75 – 7.67 (m, 3H), 7.53 (ddd, *J* = 8.7, 7.2, 1.7 Hz, 1H), 7.49 (d, *J* = 8.9 Hz, 2H), 7.08 (dd, *J* = 8.4, 1.1 Hz, 1H), 6.98 (ddd, *J* = 8.3, 7.2, 1.2 Hz, 1H); MS (HRMS-ESI): calculated for [M+H$^+$] $C_{16}H_{12}ClN_2O_2$ m/z 299.0582, found m/z 299.0577.

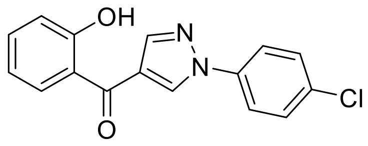

(1-(4-Bromophenyl)-1H-pyrazol-4-yl)(2-hydroxyphenyl)methanone (CK-28)[4] synthesized according to the general procedure B, obtained as a brown solid (6.60 mg, yield 13%); no further purification; ^{1}H NMR (700 MHz, DMSO-d_6) δ 11.04 (s, 1H), 9.13 (s, 1H), 8.22 (s, 1H), 7.98 – 7.94 (m, 2H), 7.77 – 7.73 (m, 3H), 7.51 (ddd, *J* = 8.6, 7.2, 1.8 Hz, 1H), 7.02 (dd, *J* = 8.3, 1.1 Hz, 1H), 6.99 (td, *J* = 7.5, 1.1 Hz, 1H). ^{13}C NMR (176 MHz, DMSO) δ 190.5, 158.9, 143.1, 138.6, 134.8, 132.9 (2C), 132.3, 131.1, 124.5, 123.9, 121.7 (2C), 120.4, 119.7, 117.7; MS (HRMS-ESI): calculated for [M+H$^+$] $C_{16}H_{12}{}^{79}BrN_2O_2$ m/z

343.0077, found m/z 343.0075. Calculated for [M+H+] $C_{16}H_{12}{}^{81}BrN_2O_2$, 345.0077, found m/z 343.0075.

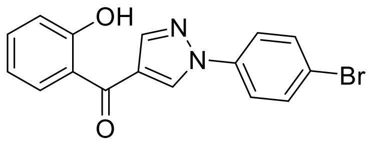

4-(4-(2-Hydroxybenzoyl)-1H-pyrazol-1-yl)benzonitrile (CK-38) synthesized according to the general procedure B with a reaction time of 2 h. After cooling the reaction mixture to room temperature, HCl (1 M) was added to the reaction mixture until pH reached 7. The precipitate was filtered off and washed with a small amount of water and ethanol. The crude product was purified by column chromatography to obtain the target compound as a pale yellow solid (7.20 mg, yield 4%); ^{1}H NMR (500 MHz, Chloroform-*d*) δ 11.91 (s, 1H), 8.58 – 8.53 (m, 1H), 8.22 (s, 1H), 7.97 – 7.91 (m, 2H), 7.88 (dd, *J* = 8.0, 1.7 Hz, 1H), 7.85 – 7.80 (m, 2H), 7.55 (ddd, *J* = 8.7, 7.2, 1.7 Hz, 1H), 7.09 (dd, *J* = 8.4, 1.1 Hz, 1H), 6.99 (ddd, *J* = 8.2, 7.2, 1.2 Hz, 1H); ^{13}C NMR (126 MHz, Chloroform-*d*) *δ* 192.0, 163.0, 143.4, 142.2, 136.6, 134.0, 131.2, 130.5, 124.6, 119.9, 119.4, 118.9, 118.1, 111.5; (LR)MS (ESI) for $C_{17}H_{12}N_3O_2$ m/z 290, found m/z 290, 99% $[M+H]^+$.

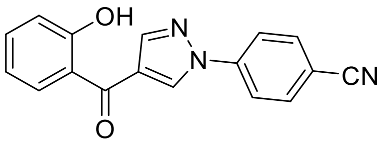

(2-Hydroxyphenyl)(1-(4-(trifluoromethoxy)phenyl)-1H-pyrazol-4-yl)methanone (CK-39) synthesized according to the general procedure B, obtained as a pale orange solid (24.10 mg, yield 48%); no further purification; ^{1}H NMR (700 MHz, DMSO-d_6) *δ* 11.04 (s,

1H), 9.15 (s, 1H), 8.23 (s, 1H), 8.13 – 8.09 (m, 2H), 7.77 – 7.74 (m, 1H), 7.59 – 7.55 (m, 2H), 7.51 (ddt, J = 8.6, 7.3, 2.0 Hz, 1H), 7.04 – 7.01 (m, 1H), 7.01 – 6.98 (m, 1H); ^{13}C NMR (176 MHz, DMSO) δ 190.6, 159.0, 147.4, 143.2, 138.2, 134.8, 132.6, 131.2, 125.2 ($^{1}J_{C,F}$ = 224.9 Hz), 124.6, 123.9, 122.8 (2C), 121.6 (2C), 119.7, 117.7; MS (HRMS-ESI): calculated for [M+H$^+$] $C_{17}H_{12}F_3N_2O_3$ m/z 349.0795, found m/z 349.0792.

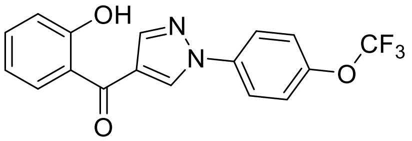

(2-Hydroxyphenyl)(1-(4-methoxyphenyl)-1H-pyrazol-4-yl)methanone (CK-40)[4] synthesized according to the general procedure B, obtained as a grey solid (20.60 mg, yield 49%); no further purification; ^{1}H NMR (600 MHz, DMSO-d_6) δ 11.12 (s, 1H), 8.99 (s, 1H), 8.16 (s, 1H), 7.90 – 7.86 (m, 2H), 7.77 (dd, J = 7.8, 1.8 Hz, 1H), 7.51 (ddd, J = 8.7, 7.3, 1.7 Hz, 1H), 7.11 – 7.07 (m, 2H), 7.02 (dd, J = 8.3, 1.1 Hz, 1H), 6.99 (td, J = 7.5, 1.1 Hz, 1H), 3.82 (s, 3H); ^{13}C NMR (151 MHz, DMSO) δ 190.7, 159.1, 158.9, 142.6, 134.7, 132.9, 131.8, 131.2, 131.1, 123.9, 123.8, 121.4 (2C), 119.7, 117.7, 115.1 (2C), 55.9; MS (HRMS-ESI): calculated for [M+H$^+$] $C_{17}H_{15}N_2O_3$ m/z 295.1077, found m/z 295.1072.

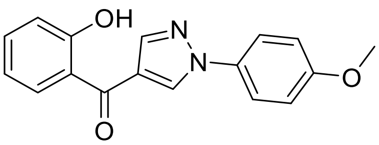

(2-Hydroxyphenyl)(1-(6-methoxypyridin-3-yl)-1H-pyrazol-4-yl)methanone (CK-47) synthesized according to the general procedure B, obtained as a shiny brow solid (15.90 mg, yield 38%); no further purification; ^{1}H NMR (500 MHz, DMSO-d_6) δ 11.07 (s,

1H), 9.07 (d, *J* = 0.6 Hz, 1H), 8.75 (dd, *J* = 2.9, 0.6 Hz, 1H), 8.27 (dd, *J* = 8.9, 2.8 Hz, 1H), 8.21 (s, 1H), 7.76 (dt, *J* = 7.8, 2.1 Hz, 1H), 7.51 (ddd, *J* = 8.7, 7.3, 1.7 Hz, 1H), 7.03 (dd, *J* = 3.9, 0.9 Hz, 1H), 7.02 – 7.00 (m, 1H), 6.99 (dd, *J* = 7.6, 1.1 Hz, 1H), 3.92 (s, 3H); ^{13}C NMR (126 MHz, DMSO) δ 190.5, 163.0, 159.0, 143.0, 138.6, 134.8, 132.5, 131.9, 131.1, 131.0, 124.2, 123.8, 119.7, 117.7, 111.5, 54.1; MS (HRMS-ESI): calculated for [M+H$^+$] $C_{16}H_{14}N_3O_3$ m/z 296.1030, found m/z 296.1024.

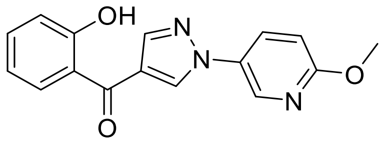

(2-Hydroxyphenyl)(1-(4-(trifluoromethyl)phenyl)-1H-pyrazol-4-yl)methanone (CK-48) synthesized according to the general procedure B, obtained as an orange solid (29.00 mg, yield 61%); no further purification; ^{1}H NMR (600 MHz, DMSO-*d*$_6$) δ 11.03 – 10.99 (m, 1H), 9.27 – 9.19 (m, 1H), 8.28 – 8.25 (m, 1H), 8.23 (d, *J* = 8.4 Hz, 1H), 8.17 – 8.07 (m, 2H), 7.93 (d, *J* = 8.6 Hz, 1H), 7.75 (dt, *J* = 7.8, 1.8 Hz, 1H), 7.54 – 7.49 (m, 1H), 7.05 – 7.02 (m, 1H), 7.00 (td, *J* = 7.5, 1.1 Hz, 1H); ^{13}C NMR (151 MHz, DMSO-*d*$_6$) δ 190.5, 158.9, 143.5 (d, $^3J_{C,F}$ = 5.4 Hz), 143.5, 134.8 (d, $^4J_{C,F}$ = 2.8 Hz), 132.8, 132.6 (d, $^3J_{C,F}$ = 5.6 Hz), 131.3, 131.1, 128.2 ($^1J_{C,F}$ = 119.6 Hz), 127.3 (q, $^3J_{C,F}$ = 3.6 Hz), 124.9 (d, $^2J_{C,F}$ = 15.0 Hz), 124.0 (d, $^4J_{C,F}$ = 2.0 Hz), 120.1, 119.8, 119.5 (d, $^2J_{C,F}$ = 12.6 Hz), 117.7. MS (HRMS-ESI): calculated for [M+H$^+$] $C_{17}H_{12}F_3N_2O_2$ m/z 333.0845, found m/z 333.0843.

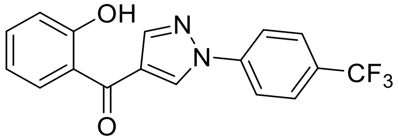

4-(4-(2-Hydroxybenzoyl)-1H-pyrazol-1-yl)benzenesulfonamide (CK-49) synthesized according to the general procedure B, obtained as a beige solid (27.50 mg, yield 56%); no further purification; ^{1}H NMR (600 MHz, DMSO-d_6) δ 11.00 (s, 1H), 9.21 (s, 1H), 8.26 (s, 1H), 8.21 – 8.17 (m, 2H), 8.00 – 7.94 (m, 2H), 7.75 (dd, J = 7.8, 1.7 Hz, 1H), 7.54 – 7.49 (m, 1H), 7.48 (s, 2H), 7.03 (dd, J = 8.3, 1.1 Hz, 1H), 7.00 (td, J = 7.5, 1.1 Hz, 1H); ^{13}C NMR (151 MHz, DMSO) δ 190.5, 158.8, 143.5, 143.0, 141.4, 134.8, 132.7, 131.1, 127.7 (2C), 124.9, 124.0, 119.8 (2C), 119.8, 117.7; (LR)MS (ESI) for $C_{16}H_{14}N_3O_4S$ m/z 344, found m/z 344, 97% $[M+H]^+$.

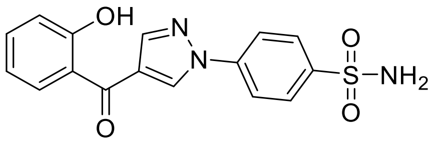

(2-Hydroxyphenyl)(1-(4-(methylsulfonyl)phenyl)-1H-pyrazol-4-yl)methanone (CK-50) synthesized according to the general procedure B, obtained as a beige solid (32.30 mg, yield 66%); no further purification; ^{1}H NMR (600 MHz, DMSO-d_6) δ 10.99 (s, 1H), 9.27 (s, 1H), 8.28 (d, J = 2.9 Hz, 2H), 8.27 (d, J = 2.0 Hz, 1H), 8.11 – 8.07 (m, 2H), 7.75 (dd, J = 7.8, 1.7 Hz, 1H), 7.52 (ddd, J = 8.3, 7.3, 1.7 Hz, 1H), 7.03 (dd, J = 8.3, 1.1 Hz, 1H), 7.01 (td, J = 7.5, 1.1 Hz, 1H), 3.29 (s, 3H); ^{13}C NMR (151 MHz, DMSO) δ 190.4, 158.8, 143.7, 142.7, 139.5, 134.8, 132.9, 131.1, 129.2 (2C), 125.1, 124.0, 120.1 (2C), 119.8, 117.7; (LR)MS (ESI) for $C_{17}H_{15}N_2O_4S$ m/z 343, found m/z 343, 99% $[M+H]^+$.

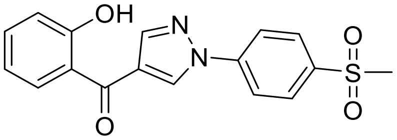

(2-Hydroxyphenyl)(1-(*p*-tolyl)-1H-pyrazol-4-yl)methanone (CK-51) synthesized according to the general procedure B, obtained as a brown solid (23.20 mg, yield 58%); no further purification; ^{1}H NMR (600 MHz, DMSO-d_6) δ 11.10 (s, 1H), 9.04 (s, 1H), 8.18 (s, 1H), 7.88 – 7.83 (m, 2H), 7.76 (dd, J = 7.8, 1.8 Hz, 1H), 7.51 (ddd, J = 8.7, 7.2, 1.7 Hz, 1H), 7.35 (d, J = 8.4 Hz, 2H), 7.02 (d, J = 8.4 Hz, 1H), 7.00 (t, J = 7.5 Hz, 1H), 2.37 (s, 3H); ^{13}C NMR (151 MHz, DMSO) δ 190.6, 159.0, 142.7, 137.4, 137.1, 134.7, 131.9, 131.1, 130.4 (2C), 124.1, 123.8, 119.7, 119.7 (2C), 117.7, 20.9; MS (HRMS-ESI): calculated for [M+H$^+$] $C_{17}H_{15}N_2O_2$ m/z 279.1128, found m/z 279.1123.

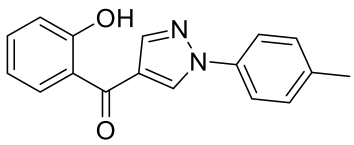

2-(4-(2-Hydroxybenzoyl)-1H-pyrazol-1-yl)benzoic acid (CK-25) synthesized according to the general procedure B, obtained as a pale yellow solid (26.50 mg, yield 60%); no further purification; ^{1}H NMR (700 MHz, DMSO-d_6) δ 13.11 (s, 1H), 11.21 (s, 1H), 8.78 (s, 1H), 8.16 (s, 1H), 7.85 (dd, J = 7.7, 1.3 Hz, 1H), 7.77 (dd, J = 7.8, 1.7 Hz, 1H), 7.74 – 7.69 (m, 2H), 7.61 (ddd, J = 7.6, 6.5, 2.1 Hz, 1H), 7.51 (ddd, J = 8.6, 7.2, 1.7 Hz, 1H), 7.02 (dd, J = 8.3, 1.1 Hz, 1H), 6.98 (td, J = 7.5, 1.1 Hz, 1H); ^{13}C NMR (176 MHz, DMSO) δ 190.8, 167.6, 159.3, 142.4, 138.2, 135.6, 134.9, 132.5, 131.2, 131.1, 130.5, 129.3, 126.4, 123.5, 123.4, 119.7, 117.8; MS (HRMS-ESI): calculated for [M+H$^+$] $C_{17}H_{13}N_2O_4$ m/z 309.0870, found m/z 309.0866.

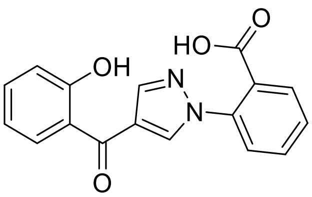

(1-(2-Chlorophenyl)-1H-pyrazol-4-yl)(2-hydroxyphenyl)methanone (CK-44) synthesized according to the general procedure B, obtained as a pale beige solid (25.80 mg, yield 60%); no further purification; ^{1}H NMR (500 MHz, DMSO-d_6) δ 11.08 (s, 1H), 8.78 (s, 1H), 8.22 (s, 1H), 7.75 – 7.69 (m, 3H), 7.60 – 7.54 (m, 2H), 7.50 (ddd, J = 8.7, 7.3, 1.7 Hz, 1H), 7.02 (dd, J = 8.4, 1.1 Hz, 1H), 6.98 (td, J = 7.5, 1.2 Hz, 1H); ^{13}C NMR (126 MHz, DMSO) δ 190.6, 159.0, 142.5, 137.4, 136.8, 134.8, 131.2, 131.0, 130.9, 128.8, 128.7, 123.8, 123.5, 119.7, 117.7; MS (HRMS-ESI): calculated for [M+H^{+}] $C_{16}H_{11}ClN_2O_2$ m/z 299.0582, found m/z 299.0577.

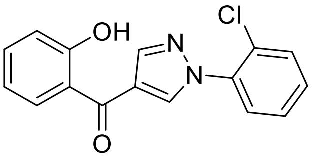

(2-Hydroxyphenyl)(1-(o-tolyl)-1H-pyrazol-4-yl)methanone (CK-45) synthesized according to the general procedure B, obtained as a brown solid (18.00 mg, yield 45%); no further purification; ^{1}H NMR (500 MHz, DMSO-d_6) δ 11.16 (s, 1H), 8.68 (d, J = 0.7 Hz, 1H), 8.19 (s, 1H), 7.78 (dd, J = 7.8, 1.7 Hz, 1H), 7.52 – 7.48 (m, 1H), 7.47 (dd, J = 7.9, 1.3 Hz, 1H), 7.46 – 7.43 (m, 2H), 7.38 (ddd, J = 7.7, 4.9, 3.6 Hz, 1H), 7.01 (dd, J = 8.3, 1.1 Hz, 1H), 6.98 (td, J = 7.5, 1.1 Hz, 1H), 2.24 (s, 3H); ^{13}C NMR (126 MHz, DMSO) δ 190.8, 159.2, 142.2, 139.2, 136.0, 134.8, 133.6, 131.7, 131.2, 129.5, 127.2, 126.5, 123.7,

123.1, 119.7, 117.7, 18.2; MS (HRMS-ESI): calculated for [M+H$^+$] $C_{17}H_{15}N_2O_2$ m/z 279.1128, found m/z 279.1123.

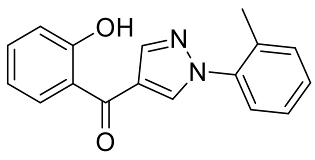

4-(4-(2-Hydroxy-5-methylbenzoyl)-1H-pyrazol-1-yl)benzoic acid (CK-54)[3] synthesized according to the general procedure B, obtained as a yellow solid (32.00 mg, yield 60%); no further purification; ^{1}H NMR (500 MHz, DMSO-d_6) δ 13.02 (s, 1H), 10.79 (s, 1H), 9.20 (s, 1H), 8.26 (s, 1H), 8.15 – 8.06 (m, 4H), 7.51 (d, J = 1.9 Hz, 1H), 7.32 (dd, J = 8.3, 2.2 Hz, 1H), 6.93 (d, J = 8.3 Hz, 1H), 2.30 (s, 3H); ^{13}C NMR (126 MHz, DMSO) δ 190.6, 167.0, 156.7, 143.5, 142.4, 135.5, 132.6, 131.3 (2C), 130.9, 129.7, 128.5, 124.9, 123.7, 119.4 (2C), 117.6; (LR)MS (ESI) for $C_{18}H_{14}N_2O_4$ m/z 322, found m/z 322, 95% [M+H]$^+$.

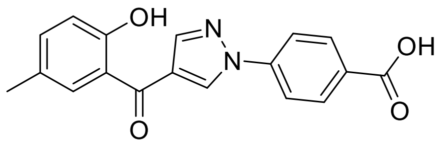

4-(4-(2-Hydroxy-5-isopropylbenzoyl)-1H-pyrazol-1-yl)benzoic acid (CK-55) synthesized according to the general procedure B, obtained as a yellow solid (31.00 mg, yield 54%); no further purification; ^{1}H NMR (500 MHz, DMSO-d_6) δ 13.09 (s, 1H), 10.71 (s, 1H), 9.18 (s, 1H), 8.23 (s, 1H), 8.15 – 8.06 (m, 4H), 7.49 (d, J = 2.3 Hz, 1H), 7.40 (dd, J = 8.5, 2.3 Hz, 1H), 6.96 (d, J = 8.5 Hz, 1H), 2.93 (hept, J = 6.9 Hz, 1H), 1.22 (s, 3H), 1.20 (s, 3H); ^{13}C NMR (126 MHz, DMSO) δ 190.5, 167.0, 156.7, 143.4, 142.4, 139.5,

132.7, 132.6, 131.3 (2C), 129.7, 128.3, 125.1, 124.0, 119.4 (2C), 117.7; MS (HRMS-ESI): calculated for [M+H$^+$] $C_{20}H_{19}N_2O_4$ m/z 351.1339, found m/z 351.1338.

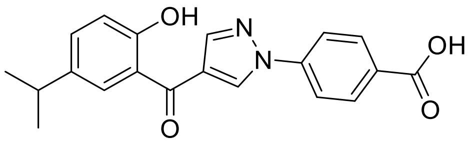

4-(4-(5-Fluoro-2-hydroxybenzoyl)-1H-pyrazol-1-yl)benzoic acid (CK-56) synthesized according to the general procedure B, obtained as a yellow solid (19.00 mg, yield 35%); purification by silica flash column chromatography (0-6% MeOH in DCM); ^{1}H NMR (700 MHz, DMSO-d_6) δ 13.13 (s, 1H), 10.49 (s, 1H), 9.19 (s, 1H), 8.24 (s, 1H), 8.13 – 8.07 (m, 4H), 7.40 (dd, J = 8.7, 3.2 Hz, 1H), 7.33 (td, J = 8.6, 3.2 Hz, 1H), 7.02 (dd, J = 9.0, 4.4 Hz, 1H); ^{13}C NMR (126 MHz, DMSO-d_6) δ 188.6, 167.0, 155.3 (d, $^1J_{C,F}$ = 235.9 Hz), 153.7 (d, $^4J_{C,F}$ = 1.4 Hz), 143.4, 142.3, 132.9, 131.3, 129.8, 125.9 (d, $^3J_{C,F}$ = 6.4 Hz), 125.0, 123.1, 120.6 (d, $^2J_{C,F}$ = 23.2 Hz), 119.4 (2C), 118.8 (d, $^3J_{C,F}$ = 7.6 Hz), 116.1 (d, $^2J_{C,F}$ = 23.9 Hz).; (LR)MS (ESI) for $C_{17}H_{12}FN_2O_4$ m/z 327, found m/z 327, 96% [M+H]$^+$.

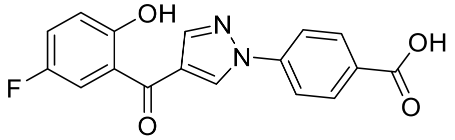

4-(4-(5-Bromo-2-hydroxybenzoyl)-1H-pyrazol-1-yl)benzoic acid (CK-60) synthesized according to the general procedure B, obtained as a yellow solid (7.10 mg, yield 11%); purification by silica flash column chromatography (0-6% MeOH in DCM (+0.1% AcOH)); ^{1}H NMR (600 MHz, DMSO-d_6) δ 13.07 (s, 1H), 10.60 (s, 1H), 9.17 (s, 1H), 8.20 (s, 1H), 8.09 (q, J = 8.6 Hz, 4H), 7.59 (dq, J = 5.5, 2.4 Hz, 2H), 6.98 (d, J = 9.5 Hz, 1H); ^{13}C NMR

(151 MHz, DMSO) δ 188.0, 167.0, 156.0, 143.3, 142.3, 135.7, 132.9, 132.0, 131.3 (2C), 129.8, 128.5, 125.6, 119.7, 119.4 (2C), 110.6; MS (ESI): calculated for $C_{17}H_{11}{}^{79}BrN_2O_4$ m/z 386.99, found m/z 384.80; calculated for $C_{17}H_{11}{}^{81}BrN_2O_4$ m/z 389, found m/z 389.

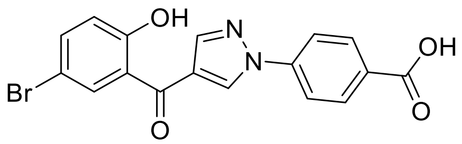

4-(4-(3,5-Dichloro-2-hydroxybenzoyl)-1H-pyrazol-1-yl)benzoic acid (CK-61) synthesized according to the general procedure B, obtained as a yellow solid (38.50 mg, yield 62%); no further purification; ^{1}H NMR (600 MHz, DMSO-d_6) δ 13.11 (s, 1H), 10.88 (s, 1H), 9.24 (s, 1H), 8.29 (s, 1H), 8.13 – 8.07 (m, 4H), 7.82 (d, J = 2.6 Hz, 1H), 7.61 (d, J = 2.6 Hz, 1H); ^{13}C NMR (151 MHz, DMSO) δ 187.9, 166.9, 152.0, 143.3, 142.2, 133.4, 132.8, 131.3 (2C), 129.9, 128.5, 128.5, 124.6, 123.8, 123.5, 119.5 (2C); (LR)MS (ESI) for $C_{17}H_{10}Cl_2N_2NaO_4$ m/z 399, found m/z 399, 83% $[M+Na]^+$.

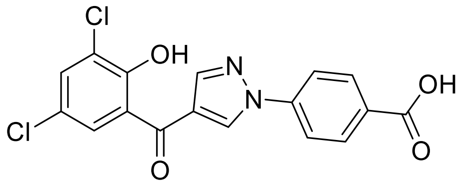

4-(4-(2-Hydroxy-5-nitrobenzoyl)-1H-pyrazol-1-yl)benzoic acid (CK-62) synthesized according to the general procedure B, obtained as a yellow solid (36.30 mg, yield 63%); no further purification; ^{1}H NMR (600 MHz, DMSO-d_6) δ 13.11 (s, 1H), 11.71 (s, 1H), 9.21 (s, 1H), 8.32 (dd, J = 9.1, 2.9 Hz, 1H), 8.28 (d, J = 2.9 Hz, 1H), 8.26 (s, 1H), 8.12 – 8.06 (m, 4H), 7.18 (d, J = 9.1 Hz, 1H); ^{13}C NMR (151 MHz, DMSO) δ 186.9, 166.9, 162.0,

143.2, 142.2, 139.8, 133.2, 131.3 (2C), 129.8, 128.3, 127.4, 126.1, 125.4, 119.3 (2C), 117.9; MS (HRMS-ESI): calculated for [M+H$^+$] $C_{17}H_{12}N_3O_6$ m/z 354.0721, found m/z 354.0719.

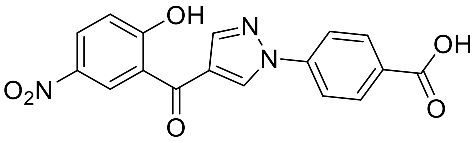

4-(4-(5-Ethyl-2-hydroxybenzoyl)-1H-pyrazol-1-yl)benzoic acid (CK-65) synthesized according to the general procedure B, obtained as a yellow solid (33.50 mg, yield 61%); no further purification; ^{1}H NMR (600 MHz, DMSO-d_6) δ 13.04 (s, 1H), 10.76 (s, 1H), 9.19 (s, 1H), 8.25 (d, J = 0.5 Hz, 1H), 8.14 – 8.06 (m, 4H), 7.50 (d, J = 2.3 Hz, 1H), 7.36 (dd, J = 8.4, 2.3 Hz, 1H), 6.95 (d, J = 8.4 Hz, 1H), 2.62 (q, J = 7.6 Hz, 2H), 1.19 (t, J = 7.6 Hz, 3H); ^{13}C NMR (151 MHz, DMSO) δ 190.6, 167.0, 156.8, 143.4, 142.4, 134.9, 134.3, 132.6, 131.3 (2C), 129.8, 129.8, 124.9, 123.8, 119.4 (2C), 117.7; MS (HRMS-ESI): calculated for [M+H$^+$] $C_{19}H_{17}N_2O_4$ m/z 337.1183, found m/z 337.1180.

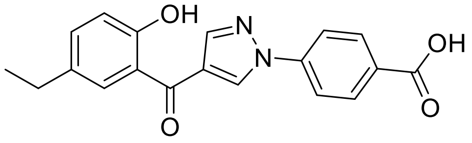

4-(4-(2-Hydroxy-5-methoxybenzoyl)-1H-pyrazol-1-yl)benzoic acid (CK-66) synthesized according to the general procedure B, obtained as a yellow solid (18.50 mg, yield 33%); no further purification; ^{1}H NMR (700 MHz, DMSO-d_6) δ 13.16 (s, 1H), 10.33 (s, 1H), 9.20 (s, 1H), 8.25 (s, 1H), 8.13 – 8.05 (m, 4H), 7.15 – 7.08 (m, 2H), 6.96 (d, J = 8.8 Hz, 1H), 3.76 (s, 3H); ^{13}C NMR (176 MHz, DMSO) δ 190.0, 167.0, 152.3, 152.2, 143.4,

142.2, 132.6, 131.3 (2C), 125.0, 124.6, 121.4, 119.3 (2C), 118.7, 114.0, 56.0; MS (HRMS-ESI): calculated for [M+H$^+$] $C_{18}H_{15}N_2O_5$ m/z 339.0976, found m/z 339.0973.

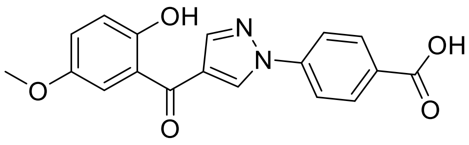

4-(4-(5-Chloro-2-hydroxybenzoyl)-1H-pyrazol-1-yl)benzoic acid (CK-67) synthesized according to the general procedure B, obtained as a yellow solid (26.70 mg, yield 47%); no further purification; ^{1}H NMR (500 MHz, DMSO-*d*$_6$) δ 12.97 (s, 1H), 10.60 (s, 1H), 9.17 (s, 1H), 8.21 (s, 1H), 8.13 – 8.06 (m, 4H), 7.51 – 7.46 (m, 2H), 7.03 (d, *J* = 8.6 Hz, 1H); ^{13}C NMR (126 MHz, DMSO) δ 188.2, 167.0, 155.6, 143.3, 142.3, 132.9, 132.9, 131.3 (2C), 129.8, 129.3, 127.8, 125.3, 123.2, 119.4 (2C), 119.3; (LR)MS (ESI) for $C_{17}H_{12}ClN_2O_4$ m/z 343, found m/z 343, 95% [M+H]$^+$.

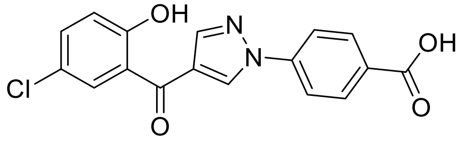

4-(4-(4-Bromo-2-hydroxybenzoyl)-1H-pyrazol-1-yl)benzoic acid (CK-70) synthesized according to the general procedure B, obtained as a yellow solid (24.10 mg, yield 63%); no further purification; ^{1}H NMR (600 MHz, DMSO-*d*$_6$) δ 13.09 (s, 1H), 11.00 (s, 1H), 9.18 (s, 1H), 8.24 (s, 1H), 8.14 – 8.07 (m, 4H), 7.55 (d, *J* = 8.3 Hz, 1H), 7.22 (d, *J* = 1.9 Hz, 1H), 7.16 (dd, *J* = 8.3, 1.9 Hz, 1H); ^{13}C NMR (151 MHz, DMSO) δ 189.0, 166.9, 158.5, 143.3, 142.3, 132.9, 132.3, 131.3 (2C), 129.8, 126.6, 125.1, 124.8, 122.6, 120.1, 119.4 (2C); (LR)MS (ESI) for $C_{17}H_{11}{}^{79}BrN_2O_4$ m/z 387, found m/z 387, [M+H]$^+$; for $C_{17}H_{11}{}^{81}BrN_2O_4$ m/z 389, found m/z 389, 84% [M+H]$^+$.

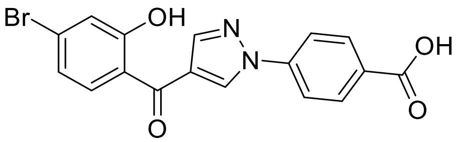

4-(4-(2-Hydroxy-4-methylbenzoyl)-1H-pyrazol-1-yl)benzoic acid (CK-73) synthesized according to the general procedure B, obtained as a brown solid (11.50 mg, yield 27%); no further purification; ^{1}H NMR (700 MHz, DMSO-d_6) δ 13.11 (s, 1H), 11.45 (s, 1H), 9.23 (s, 1H), 8.29 (s, 1H), 8.15 – 8.07 (m, 4H), 7.78 (d, J = 8.0 Hz, 1H), 6.86 (s, 1H), 6.84 (dd, J = 8.0, 1.6 Hz, 1H), 2.35 (s, 3H); ^{13}C NMR (176 MHz, DMSO) δ 190.7, 167.0, 160.4, 146.5, 143.4, 142.3, 132.5, 131.7, 131.3 (2C), 129.8, 124.4, 120.9, 120.1, 119.4 (2C), 118.0; MS (HRMS-ESI): calculated for [M+H$^+$] $C_{18}H_{15}N_2O_4$ m/z 323.1026, found m/z 323.1023.

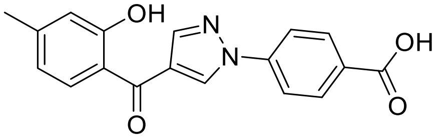

4-(4-(2-Hydroxy-1-naphthoyl)-1H-pyrazol-1-yl)benzoic acid (CK-75) synthesized according to the general procedure B, obtained as a orange solid (17.20 mg, yield 43%); no further purification; ^{1}H NMR (700 MHz, DMSO-d_6) δ 13.10 (s, 1H), 10.14 (s, 1H), 9.04 (s, 1H), 8.09 (s, 1H), 8.08 – 8.02 (m, 4H), 7.94 (d, J = 8.9 Hz, 1H), 7.89 (dd, J = 8.2, 1.4 Hz, 1H), 7.52 (dd, J = 8.5, 1.0 Hz, 1H), 7.41 (ddd, J = 8.4, 6.7, 1.4 Hz, 1H), 7.34 (ddd, J = 8.0, 6.7, 1.2 Hz, 1H), 7.28 (d, J = 8.9 Hz, 1H); ^{13}C NMR (176 MHz, DMSO) δ 190.5, 166.9, 152.7, 143.0, 142.3, 132.5, 131.8, 131.5, 131.2 (2C), 129.7, 128.6, 128.1, 127.5, 123.5, 120.7, 119.3 (2C), 118.9; MS (HRMS-ESI): calculated for $C_{21}H_{15}N_2O_4$ m/z 359.1038, found m/z 359.1024.

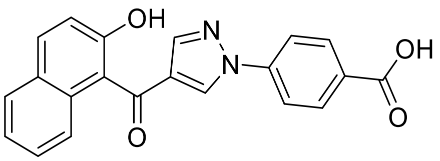

4-(4-(5-Chloro-2-hydroxy-4-methylbenzoyl)-1H-pyrazol-1-yl)benzoic acid (CK-79)[5] synthesized according to the general procedure B, obtained as a yellow solid (19.00 mg, yield 47%); purification by flash column chromatography (0-6% MeOH in DCM (+0.1% AcOH)); ^{1}H NMR (700 MHz, DMSO-d_6) δ 13.10 (s, 1H), 10.76 (s, 1H), 9.20 (s, 1H), 8.23 (s, 1H), 8.09 (m, 4H), 7.57 (s, 1H), 7.00 (s, 1H), 2.35 (s, 3H); ^{13}C NMR (151 MHz, DMSO) δ 188.4, 167.0, 156.5, 143.3, 142.3, 141.8, 132.8, 131.3 (2C), 130.2, 129.9, 125.0, 124.5, 123.8, 120.0, 119.4 (2C); (LR)MS (ESI) for $C_{18}H_{14}ClN_2O_4$ m/z 357, found m/z 357, 87% $[M+H]^+$.

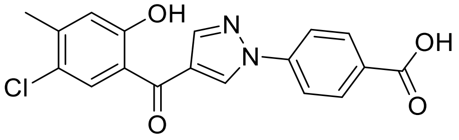

4-(4-(2-Hydroxy-6-methoxybenzoyl)-1H-pyrazol-1-yl)benzoic acid (CK-85) synthesized according to the general procedure B, obtained as a red-brown solid (3.40 mg, yield 8%); no further purification; ^{1}H NMR (600 MHz, DMSO-d_6) δ 13.10 (s, 1H), 9.73 (s, 1H), 8.97 (d, J = 0.6 Hz, 1H), 8.10 – 8.03 (m, 4H), 7.99 (s, 1H), 7.26 – 7.21 (m, 1H), 6.61 – 6.55 (m, 2H), 3.68 (s, 3H); ^{13}C NMR (151 MHz, DMSO) δ 206.9, 188.1, 166.9, 157.7, 155.5, 142.8, 142.4, 131.9, 131.3 (2C), 131.1, 127.4, 119.3 (2C), 117.6, 109.2, 102.8, 56.0; (LR)MS (ESI) for $C_{18}H_{15}N_2O_5$ m/z 339, found m/z 339, 99% $[MH^+]$.

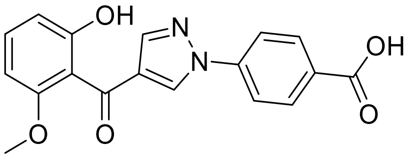

4-(4-(3,5-Difluoro-2-hydroxybenzoyl)-1H-pyrazol-1-yl)benzoic acid (CK-86) synthesized according to the general procedure B, obtained as a brown solid (14.90 mg, yield 36%); no further purification; ^{1}H NMR (700 MHz, DMSO-d_6) δ 13.12 (s, 1H), 10.31 (s, 1H), 9.18 (s, 1H), 8.25 (s, 1H), 8.12 – 8.06 (m, 4H), 7.52 (ddd, J = 11.3, 8.6, 3.1 Hz, 1H), 7.22 (ddd, J = 8.4, 3.1, 1.6 Hz, 1H); ^{13}C NMR (176 MHz, DMSO-d_6) δ 187.2, 166.9, 155.0 (d, $^{3}J_{C,F}$ = 10.1 Hz), 154. 4 ($^{1}J_{C,F}$ = 238.7 Hz) 153.6 (d, $^{3}J_{C,F}$ (CF) = 10.8 Hz), 152.4 (d, $^{4}J_{C,F}$ = 2.3 Hz), 143.2, 142.3, 133.3, 131.3 (2C), 129.8, 125.1, 119.4 (2C), 111.2 (d, $^{2}J_{C,F}$ = 23.3 Hz), 107.8 (dd, $^{2}J_{C,F}$ = 27.5, 22.7 Hz).; (LR)MS (ESI) for $C_{17}H_{11}F_2N_2O_4$ m/z 345, found m/z 345, 98% $[M+H]^+$.

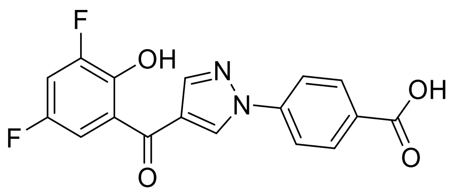

4-(4-(4-Chloro-2-hydroxybenzoyl)-1H-pyrazol-1-yl)benzoic acid (CK-88) synthesized according to the general procedure B, obtained as a orange solid (18.90 mg, yield 46%); no further purification; ^{1}H NMR (700 MHz, DMSO-d_6) δ 13.10 (s, 1H), 11.07 (s, 1H), 9.19 (s, 1H), 8.24 (s, 1H), 8.13 – 8.06 (m, 4H), 7.65 (d, J = 8.3 Hz, 1H), 7.08 (d, J = 2.0 Hz, 1H), 7.02 (dd, J = 8.3, 2.0 Hz, 1H); ^{13}C NMR (176 MHz, DMSO) δ 188.9, 166.9, 158.7, 143.3, 142.3, 137.9, 132.8, 132.3, 131.3 (2C), 129.8, 125.1, 124.4, 119.8, 119.4 (2C), 117.2; (LR)MS (ESI) for $C_{17}H_{12}ClN_2O_4$ m/z 343, found m/z 343, 97% $[M+H]^+$.

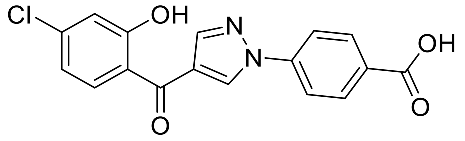

4-(4-(4-Fluoro-2-hydroxybenzoyl)-1H-pyrazol-1-yl)benzoic acid (CK-89) synthesized according to the general procedure B, obtained as a orange solid (19.80 mg, yield 47%); no further purification; ^{1}H NMR (700 MHz, DMSO-d_6) δ 13.12 (s, 1H), 11.49 (s, 1H), 9.22 (s, 1H), 8.27 (s, 1H), 8.13 – 8.07 (m, 4H), 7.84 (dd, J = 8.7, 6.7 Hz, 1H), 6.87 – 6.81 (m, 2H); ^{13}C NMR (176 MHz, DMSO-d_6) δ 189.3, 166.9, 165.8 (d, $^{1}J_{C,F}$ = 250.8 Hz), 161.2 (d, $^{3}J_{C,F}$ = 12.8 Hz), 143.4, 142.3, 133.7 (d, $^{3}J_{C,F}$ = 11.4 Hz), 132.7, 131.3 (2C), 129.8, 124.7, 121.1 (d, $^{4}J_{C,F}$ = 1.9 Hz), 119.4 (2C), 107.2 (d, $^{2}J_{C,F}$ = 22.1 Hz), 104.4 (d, $^{2}J_{C,F}$ = 23.8 Hz).; (LR)MS (ESI) for $C_{17}H_{12}FN_2O_4$ m/z 327, found m/z 327, 99% $[M+H]^+$.

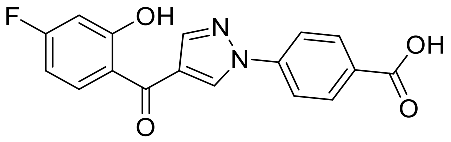

4-(4-(3-Chloro-2-hydroxybenzoyl)-1H-pyrazol-1-yl)benzoic acid (CK-96) synthesized according to the general procedure B, obtained as a orange solid (5.90 mg, yield 14%); no further purification; ^{1}H NMR (700 MHz, DMSO-d_6) δ 13.15 (s, 1H), 11.61 (s, 1H), 9.28 (s, 1H), 8.34 (s, 1H), 8.15 – 8.08 (m, 4H), 7.86 (dd, J = 7.8, 1.6 Hz, 1H), 7.74 (dd, J = 7.9, 1.5 Hz, 1H), 7.06 (td, J = 7.9, 5.0 Hz, 1H); ^{13}C NMR (176 MHz, DMSO) δ 190.4, 155.1, 143.5, 142.57, 142.3, 135.1, 135.1, 133.1, 131.3 (2C), 130.3, 124.6, 124.0, 122.0, 120.7, 119.6 (2C); (LR)MS (ESI) for $C_{17}H_{11}ClN_2NaO_4$ m/z 365, found m/z 365, 99% $[M+Na]^+$.

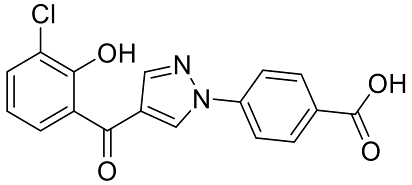

Methyl (*E*)-3-(3-(3-amino-2-cyano-3-oxoprop-1-en-1-yl)-1H-pyrazol-1-yl)benzoate (CK-94) 1*H*-pyrazole-3-carbaldehyde (1 eq.), 3-methoxycarbonylphenylboronic acid (2 eq.), pyridine (2 eq.) and Cu(OAc)2 (1.5 eq.) were dissolved in DCM (3 mL) and stirred overnight at ambient temperature. The reaction mixture was filtered over celite, concentrated and purified by silica flash column chromatography (MeOH in DCM, 0-0.5 %) to give the intermediate methyl 3-(3-formyl-1*H*-pyrazol-1-yl)benzoate as a white solid. This was dissolved in ethanol (1.5 mL) and 2-cyanoacetamide (0.9 eq.) and piperidine (0.9 eq.) were added. The reaction mixture was refluxed for 2 h, the resulting solid was filtered, washed with EtOH and dried to give methyl (*E*)-3-(3-(3-amino-2-cyano-3-oxoprop-1-en-1-yl)-1H-pyrazol-1-yl)benzoate (42.30 mg, yield 69%) as a white solid; ^{1}H NMR (600 MHz, DMSO-d_6) δ 8.89 (dd, J = 2.8, 0.6 Hz, 1H), 8.50 – 8.47 (m, 1H), 8.24 – 8.21 (m, 2H), 8.08 – 8.02 (m, 1H), 7.99 (ddd, J = 7.8, 1.6, 1.0 Hz, 1H), 7.81 – 7.77 (m, 1H), 7.74 (t, J = 8.0 Hz, 1H), 7.29 (d, J = 2.7 Hz, 1H), 3.92 (s, 3H); MS (ESI): calculated for $C_{15}H_{12}N_4NaO_3$ m/z 319, found m/z 319, 99% $[M+Na]^+$.

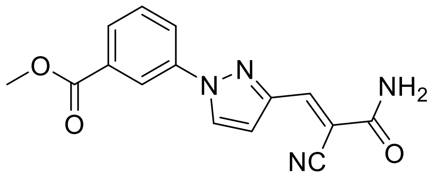

NMR Spectra

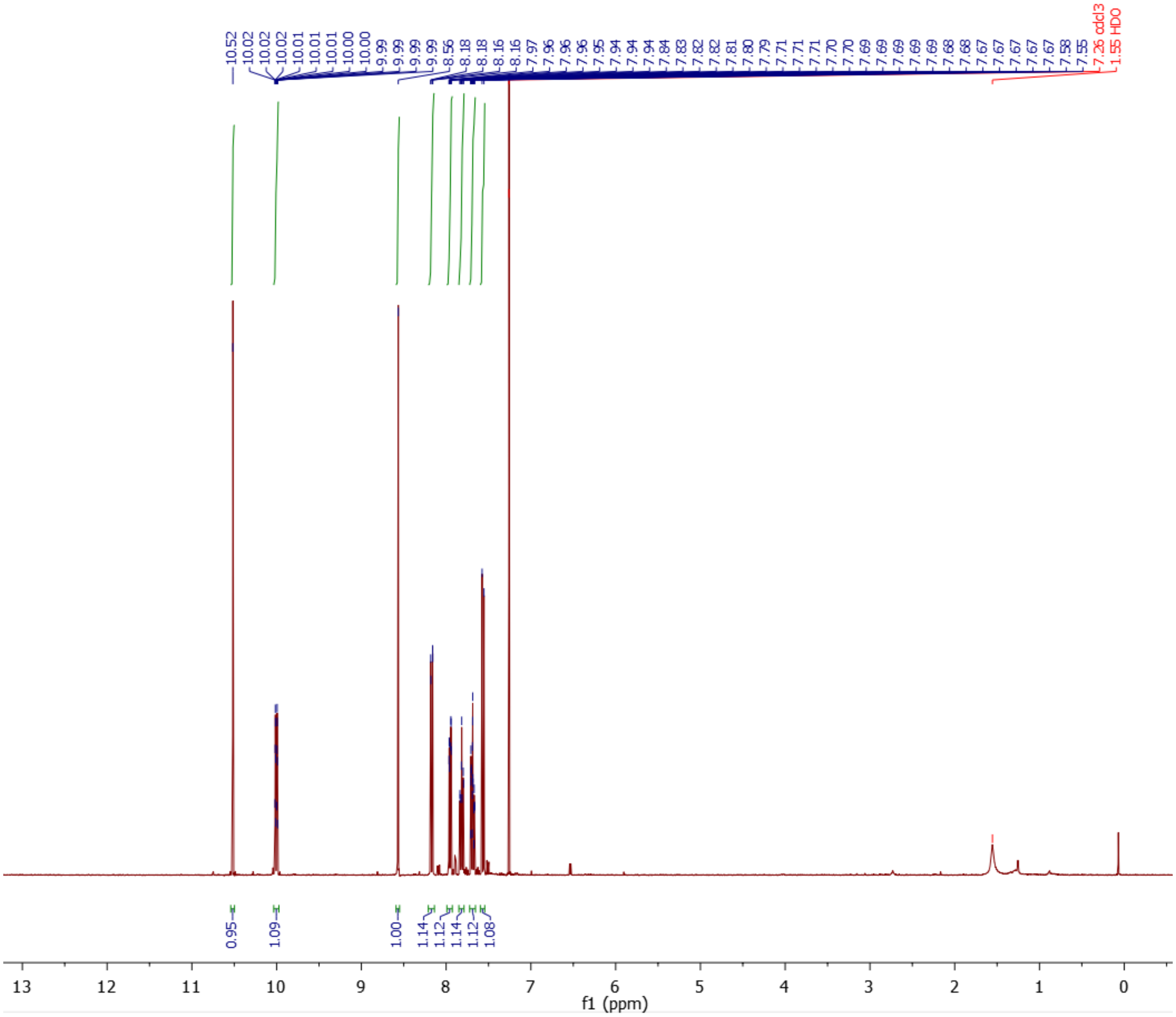

^{1}H NMR of compound **CK-57** (400 MHz, $CDCl_3$).

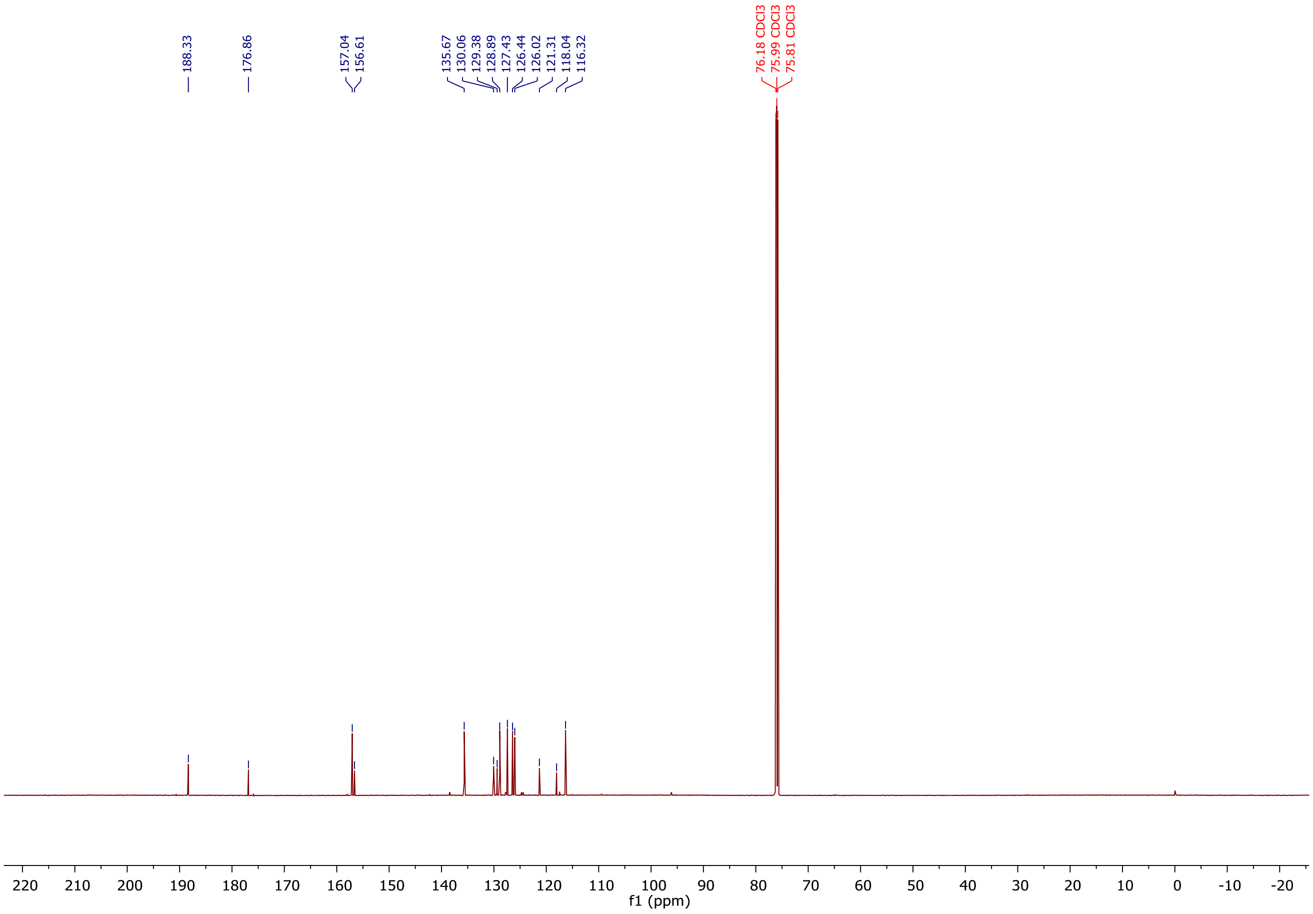

^{13}C NMR of compound **CK-57** (176 MHz, $CDCl_3$).

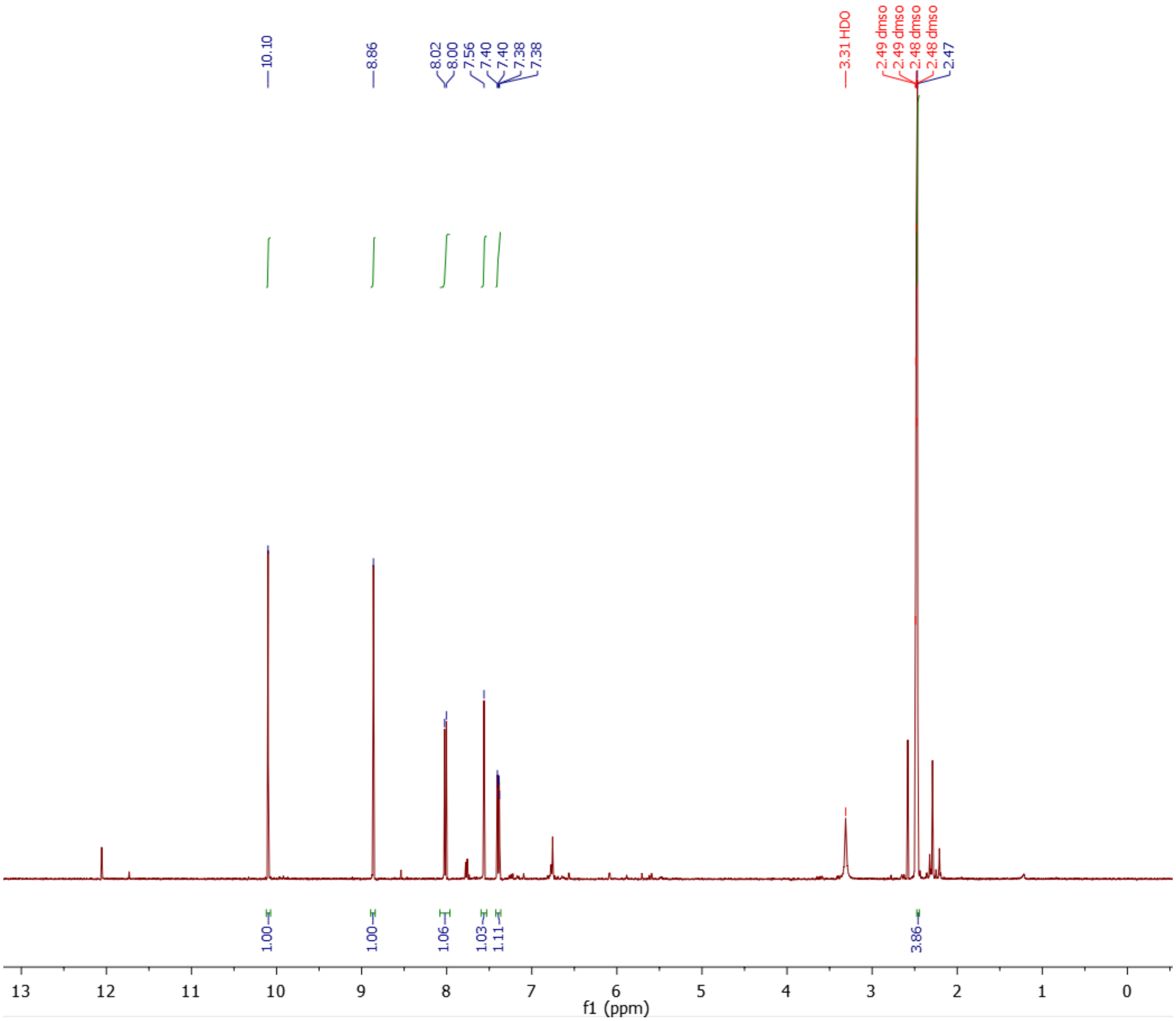

^{1}**H NMR** of compound **CK-53** (400 MHz, DMSO-d_6).

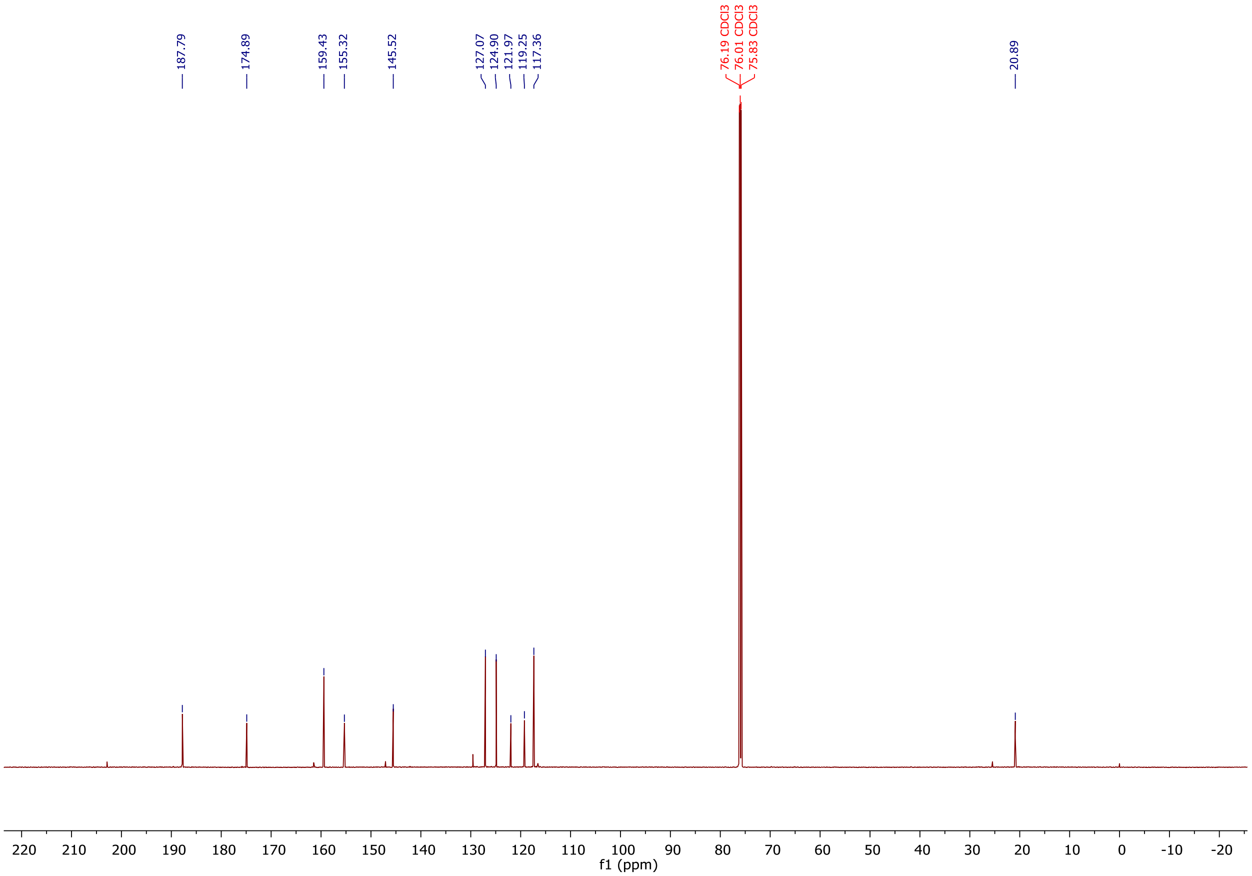

^{13}C NMR of compound **CK-53** (176 MHz, $CDCl_3$).

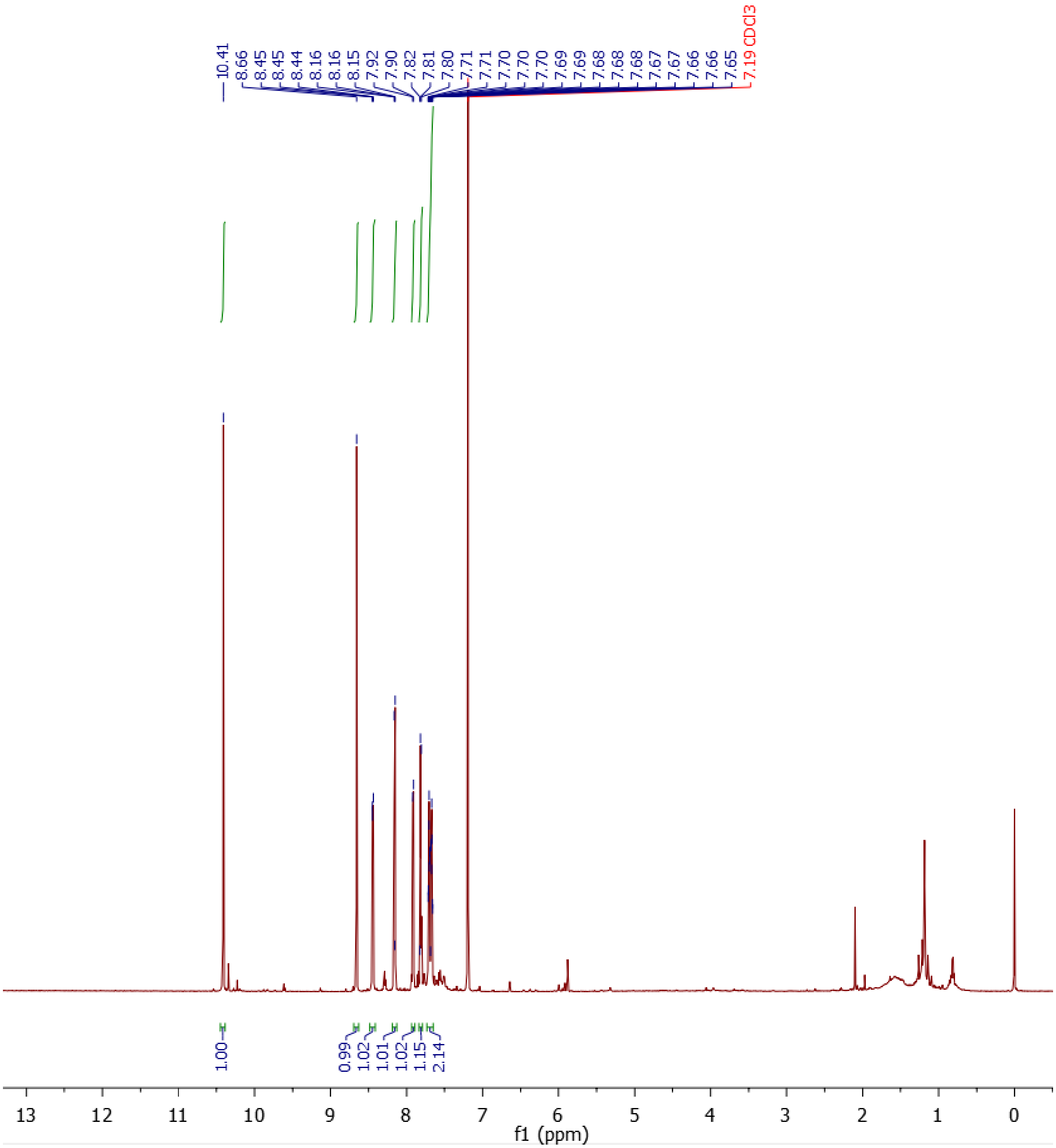

^{1}H NMR of compound **CK-58** (700 MHz, $CDCl_3$).

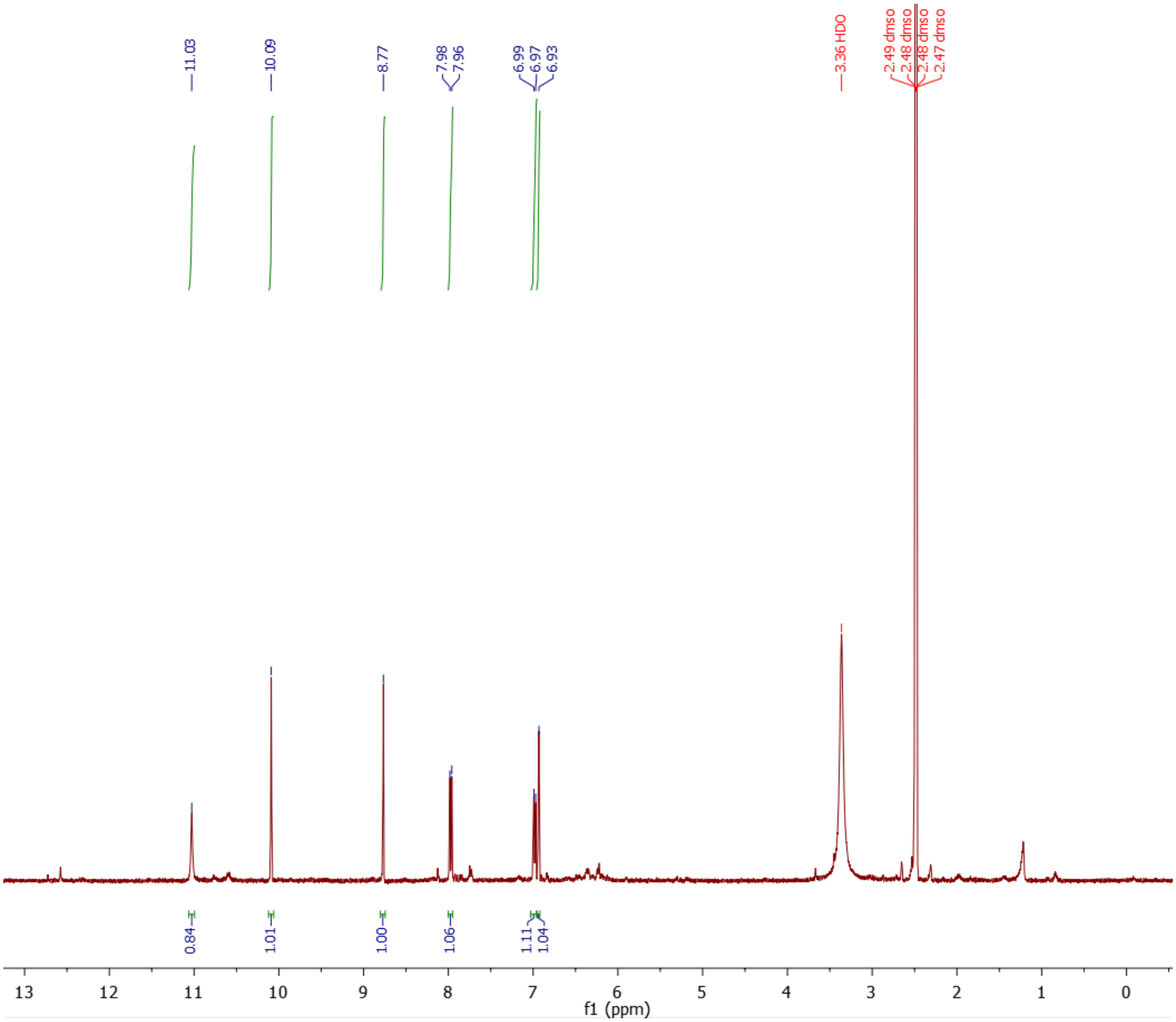

[1]**H NMR** of compound **CK-63** (400 MHz, DMSO-*d*6).

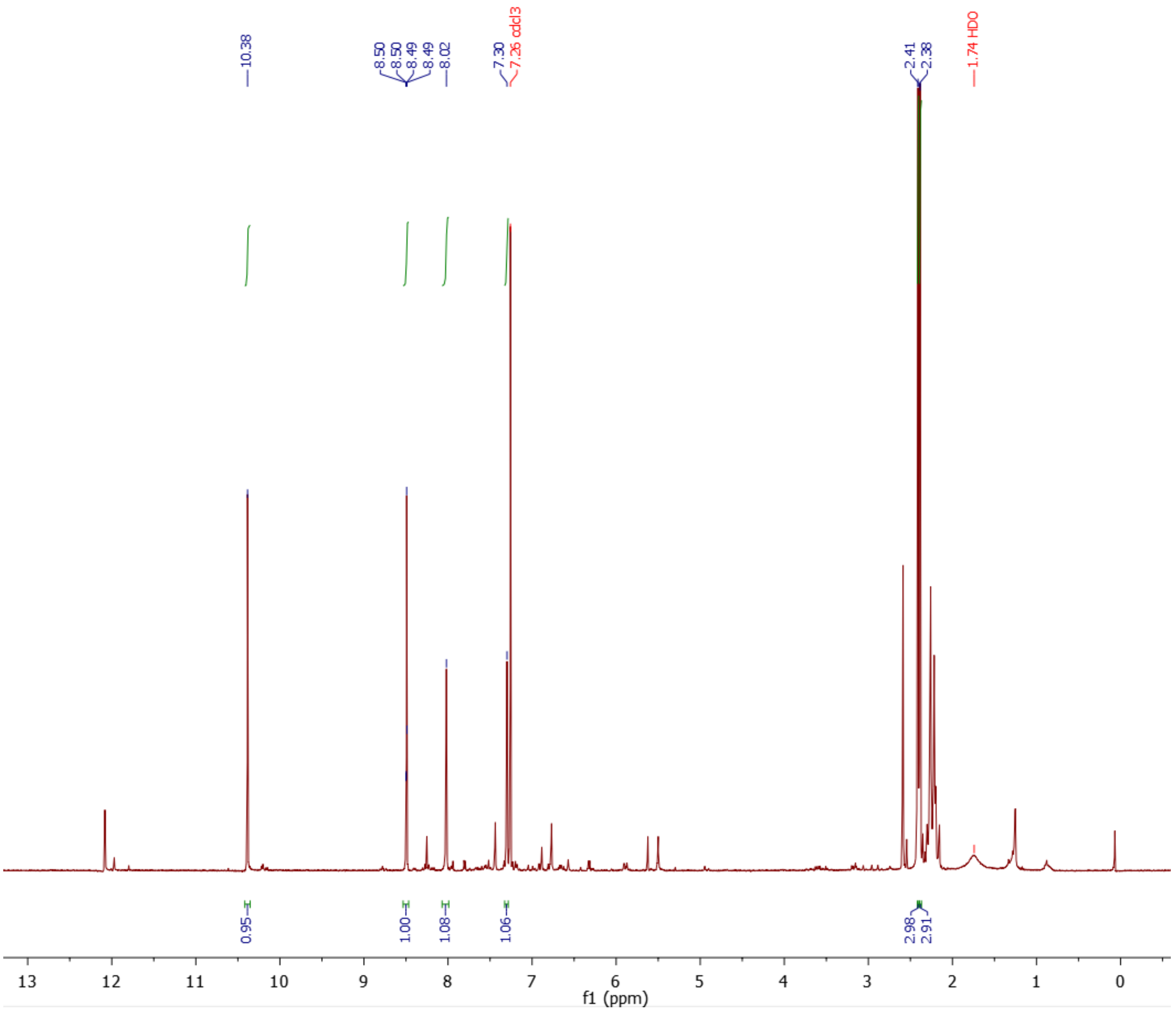

^{1}H NMR of compound **CK-64** (400 MHz, $CDCl_3$).

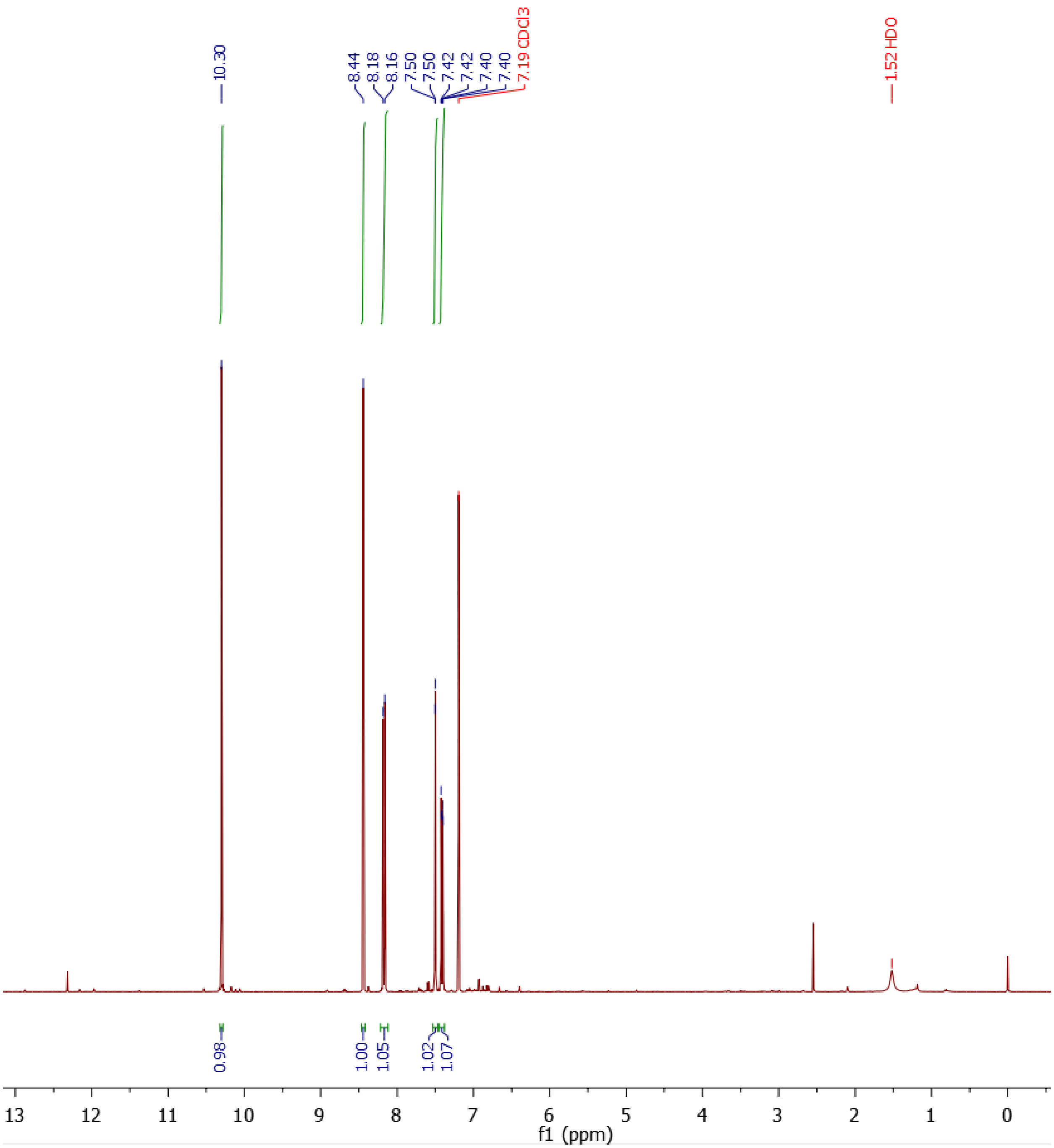

[1]**H NMR** of compound **CK-71** (400 MHz, $CDCl_3$).

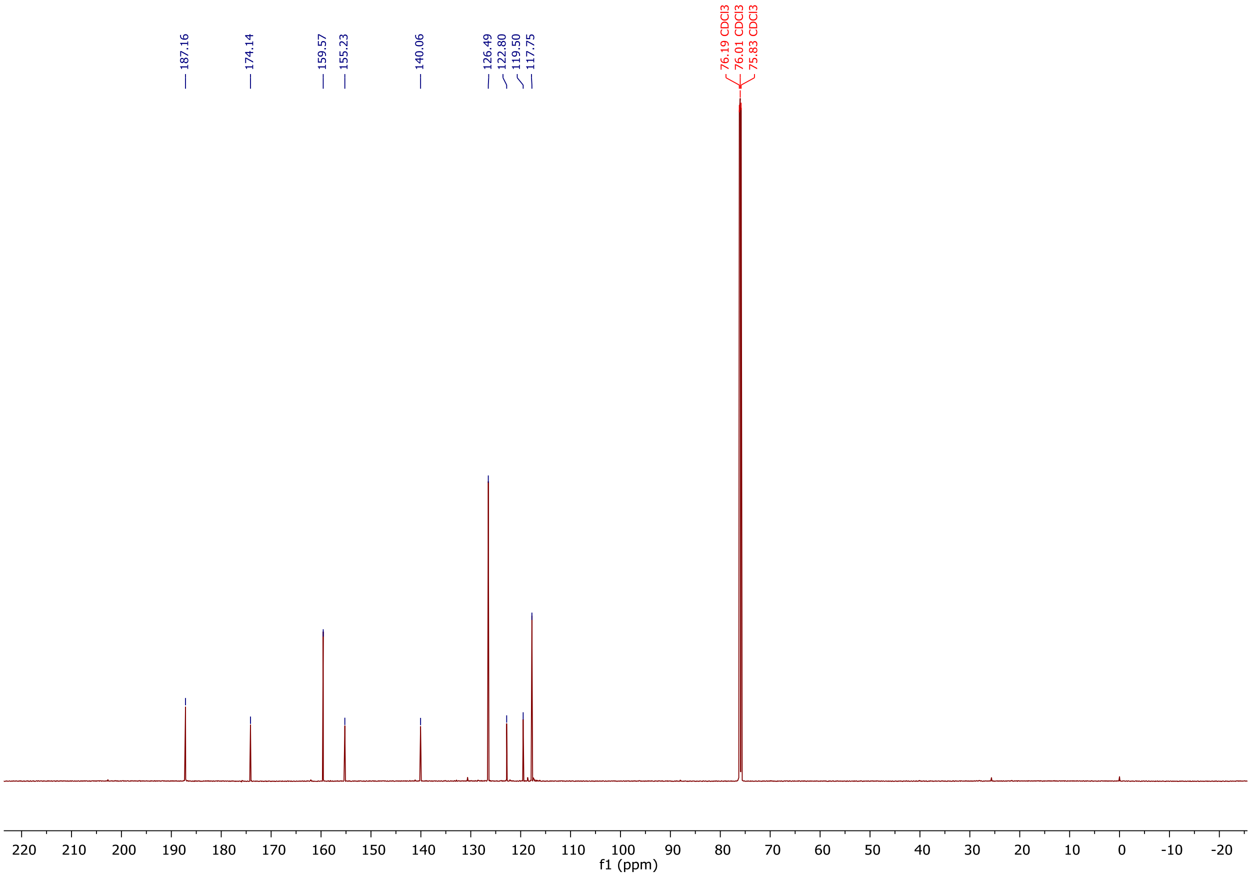

^{13}C NMR of compound **CK-71** (176 MHz, $CDCl_3$).

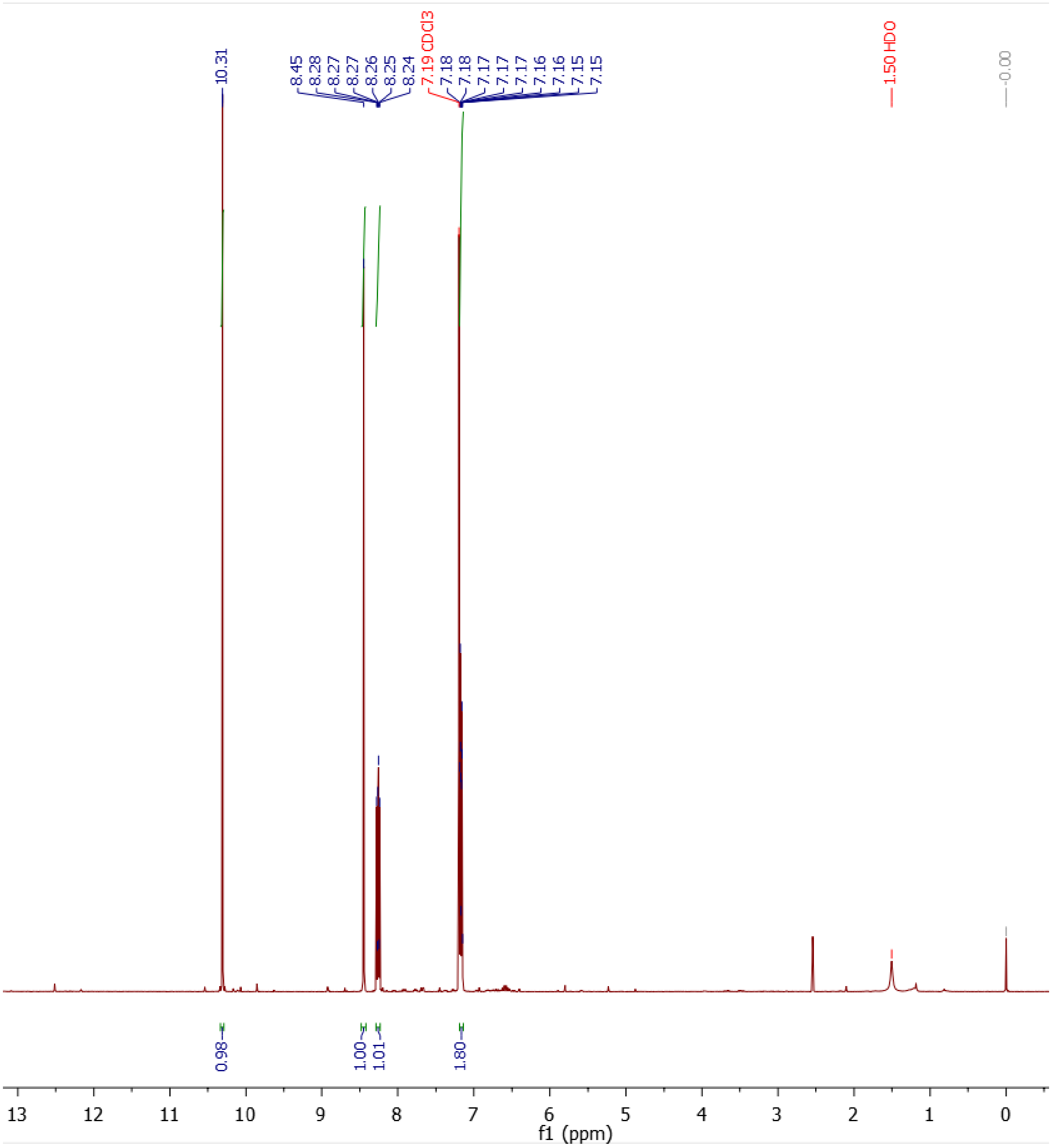

^{1}H NMR of compound **CK-72** (400 MHz, $CDCl_3$).

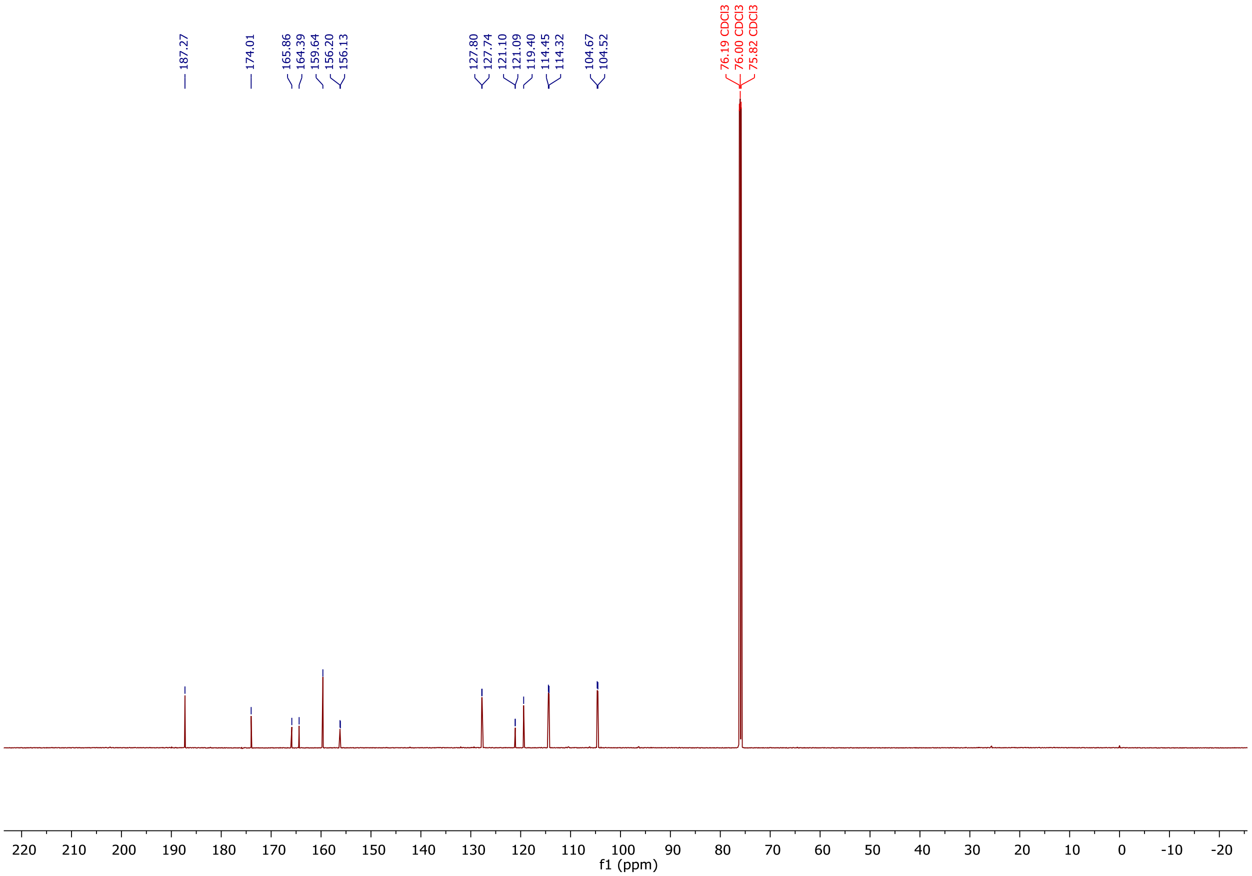

^{13}C NMR of compound **CK-72** (176 MHz, $CDCl_3$).

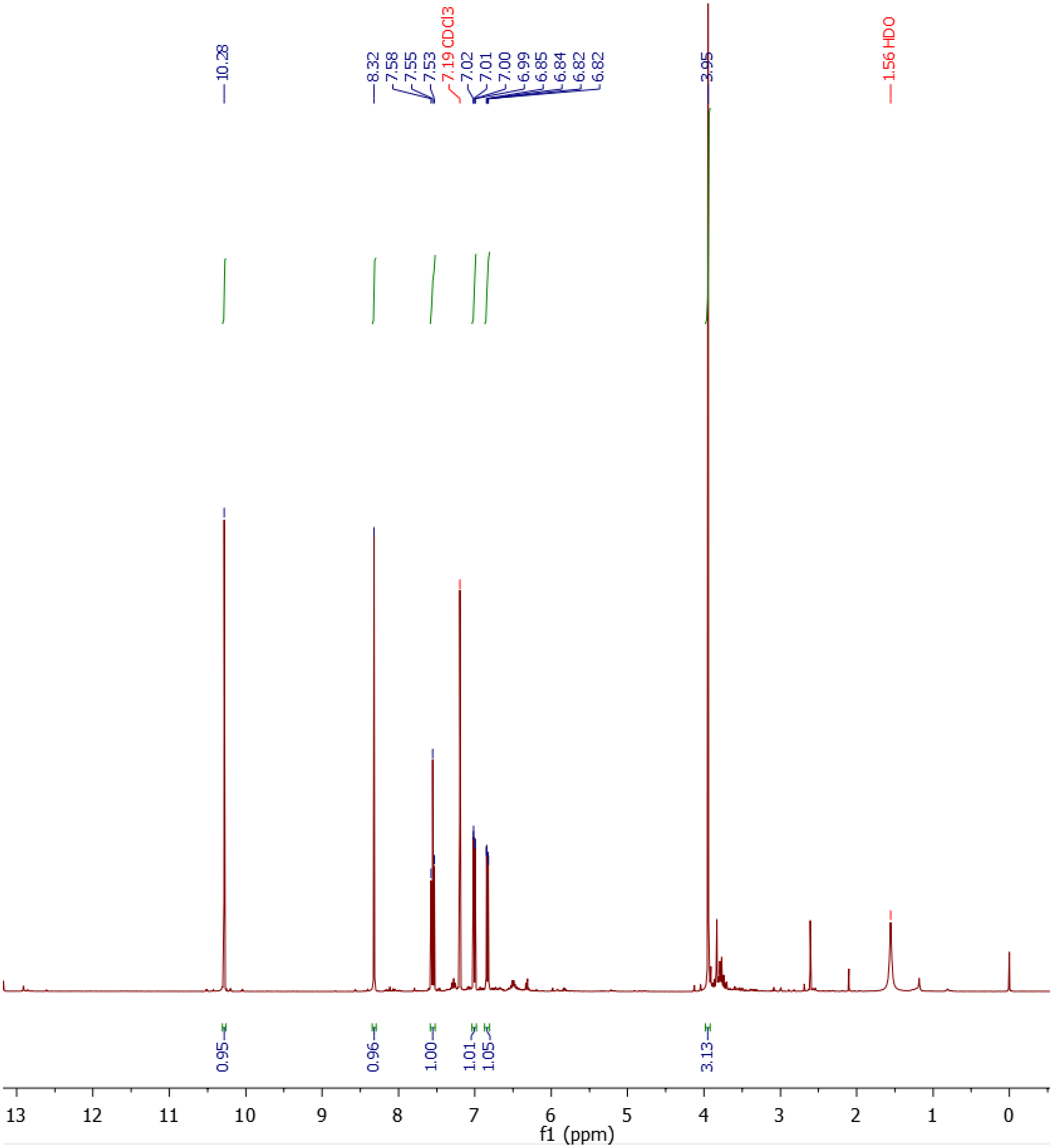

^{1}H NMR of compound **CK-77** (400 MHz, $CDCl_3$).

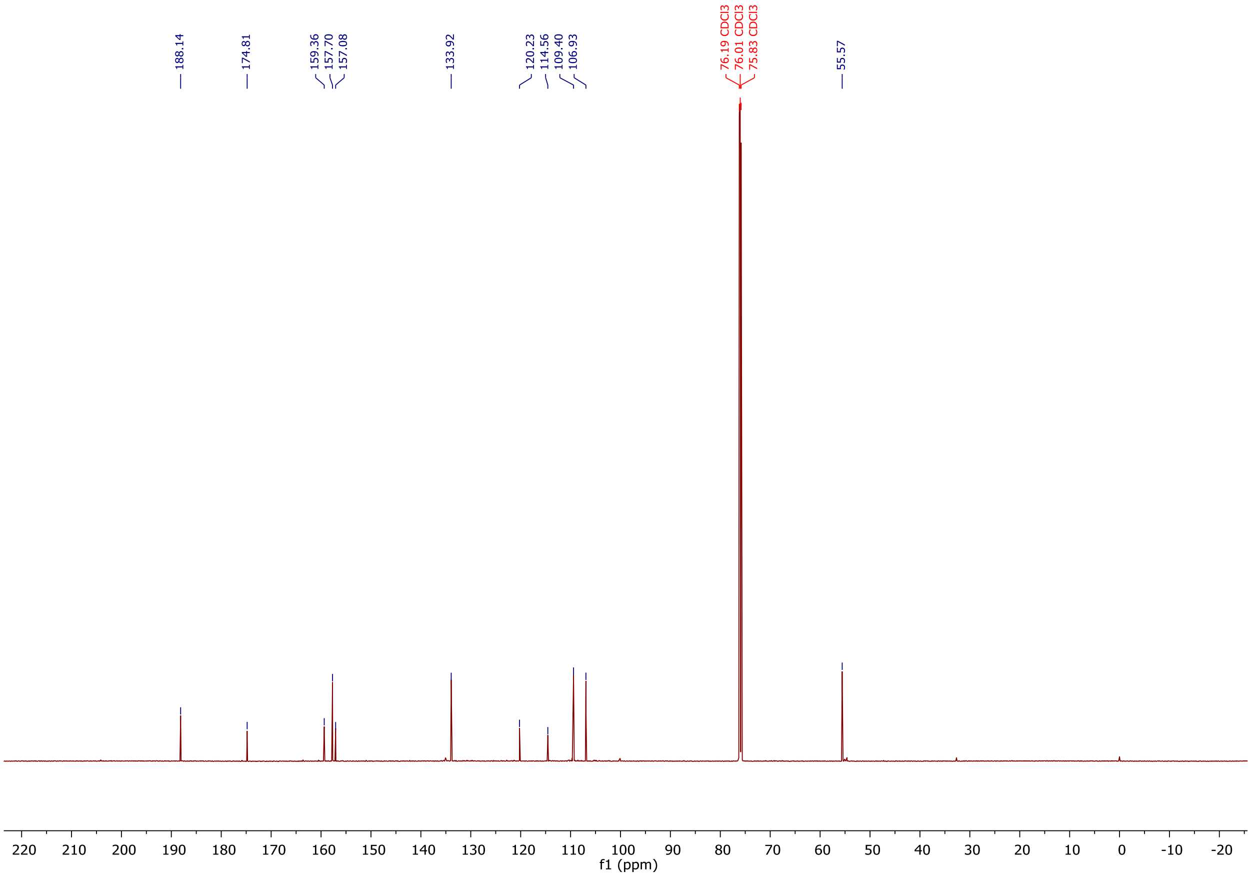

^{13}C NMR of compound **CK-77** (176 MHz, $CDCl_3$).

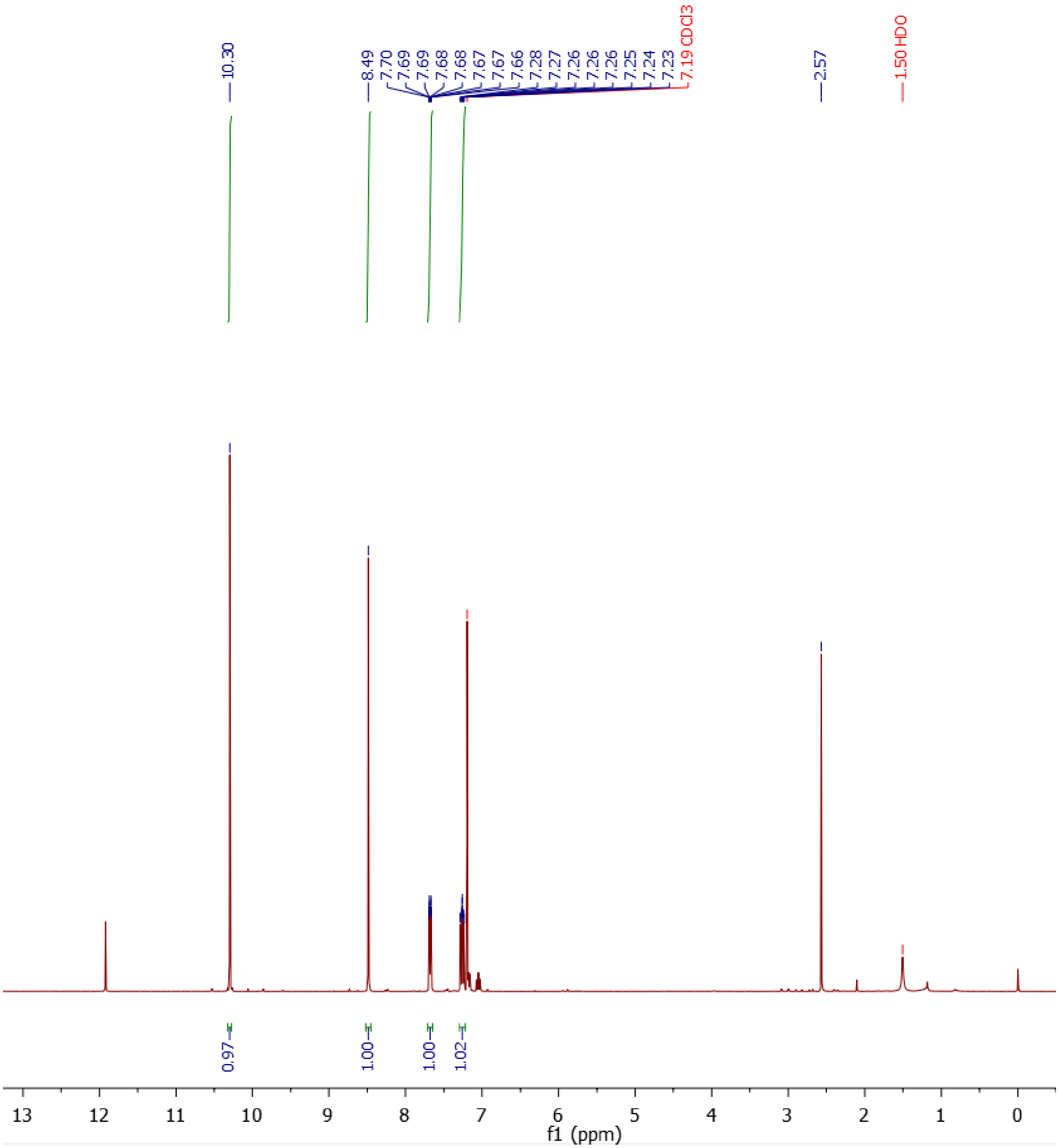

^{1}H NMR of compound **CK-82** (400 MHz, $CDCl_3$).

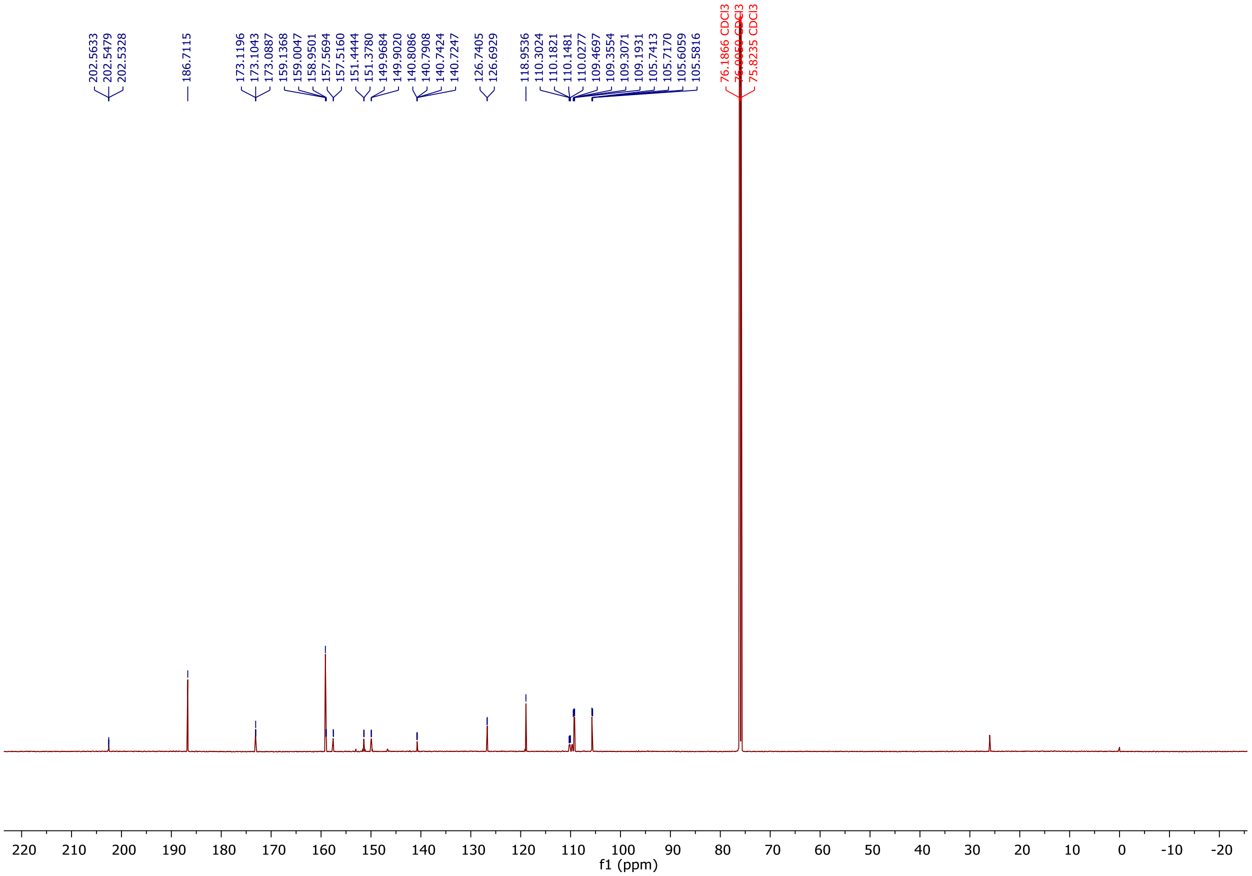

^{13}C NMR of compound **CK-82** (176 MHz, $CDCl_3$).

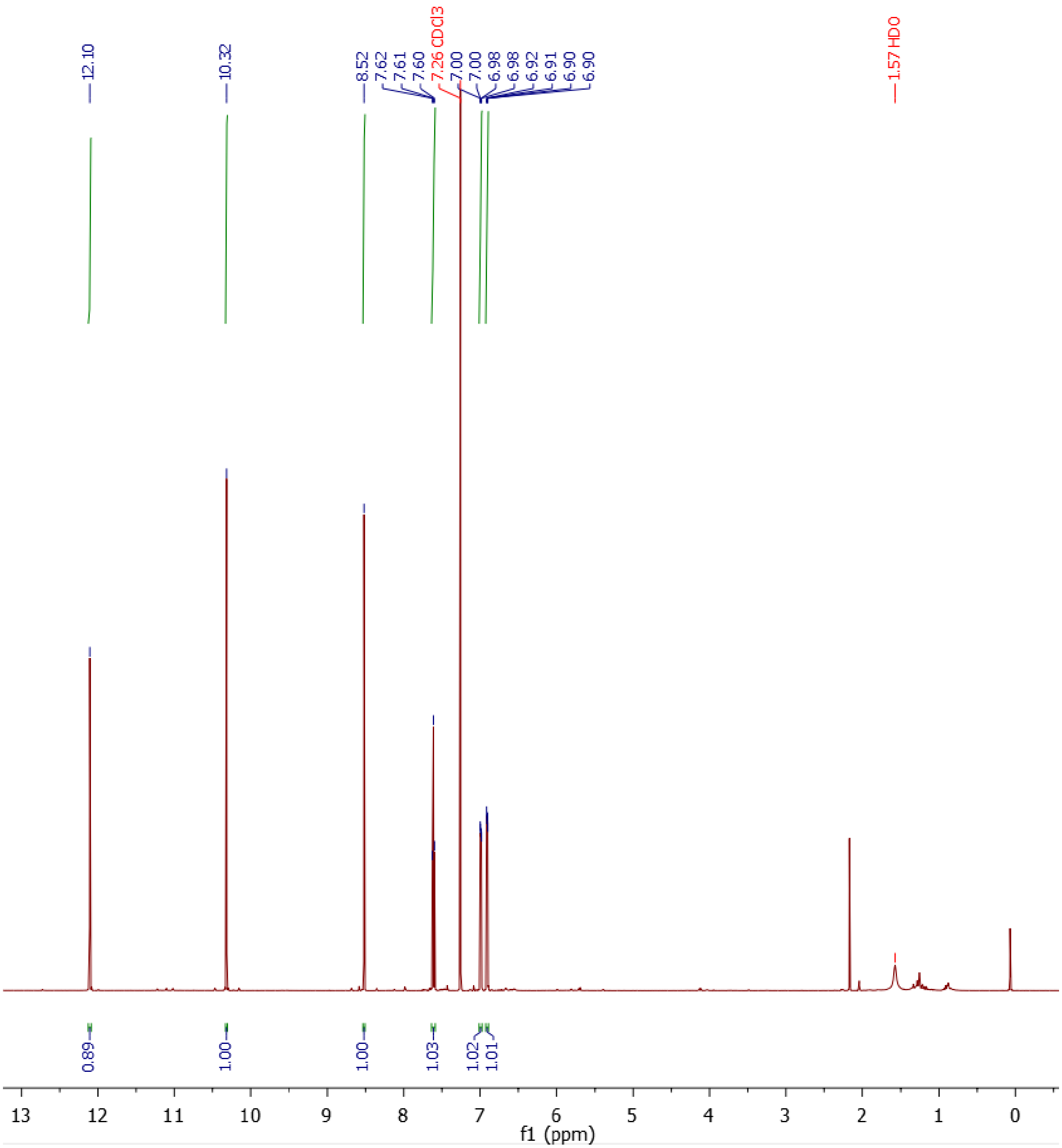

[1]**H NMR** of compound **CK-90** (600 MHz, $CDCl_3$).

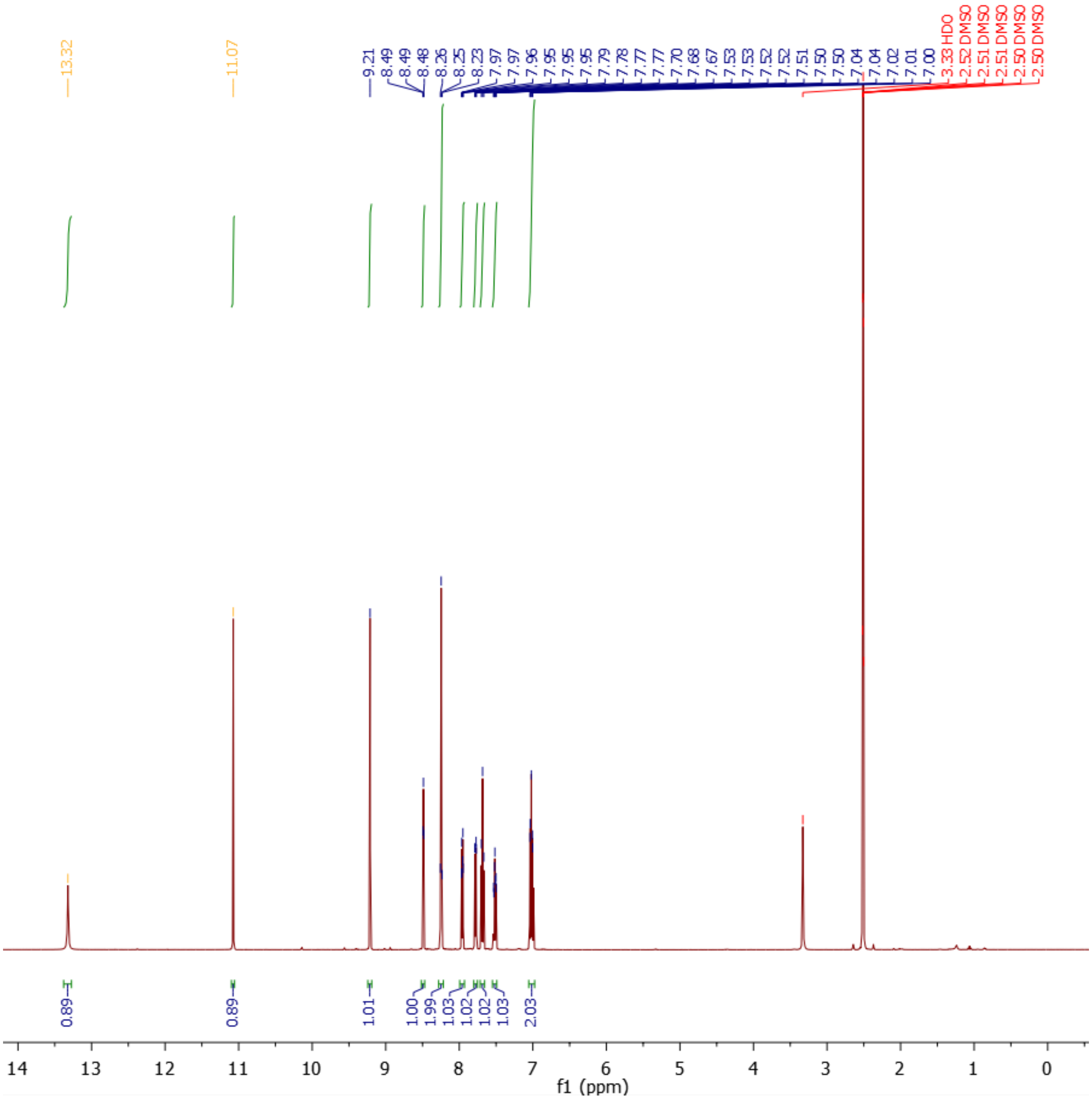

^{1}H NMR of compound **CK-23** (500 MHz, DMSO-d_6).

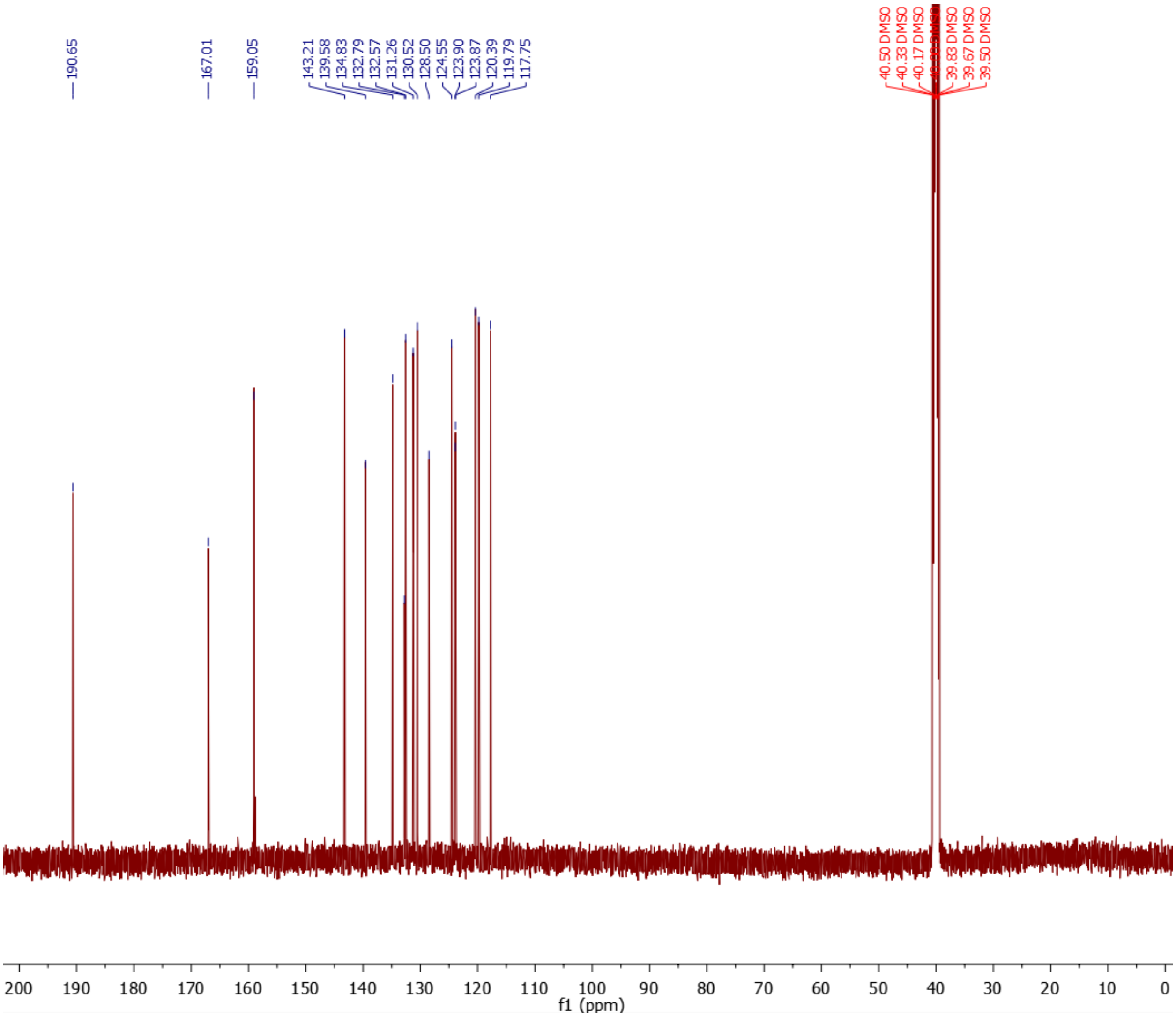

^{13}C NMR of compound **CK-23** (126 MHz, DMSO-d_6).

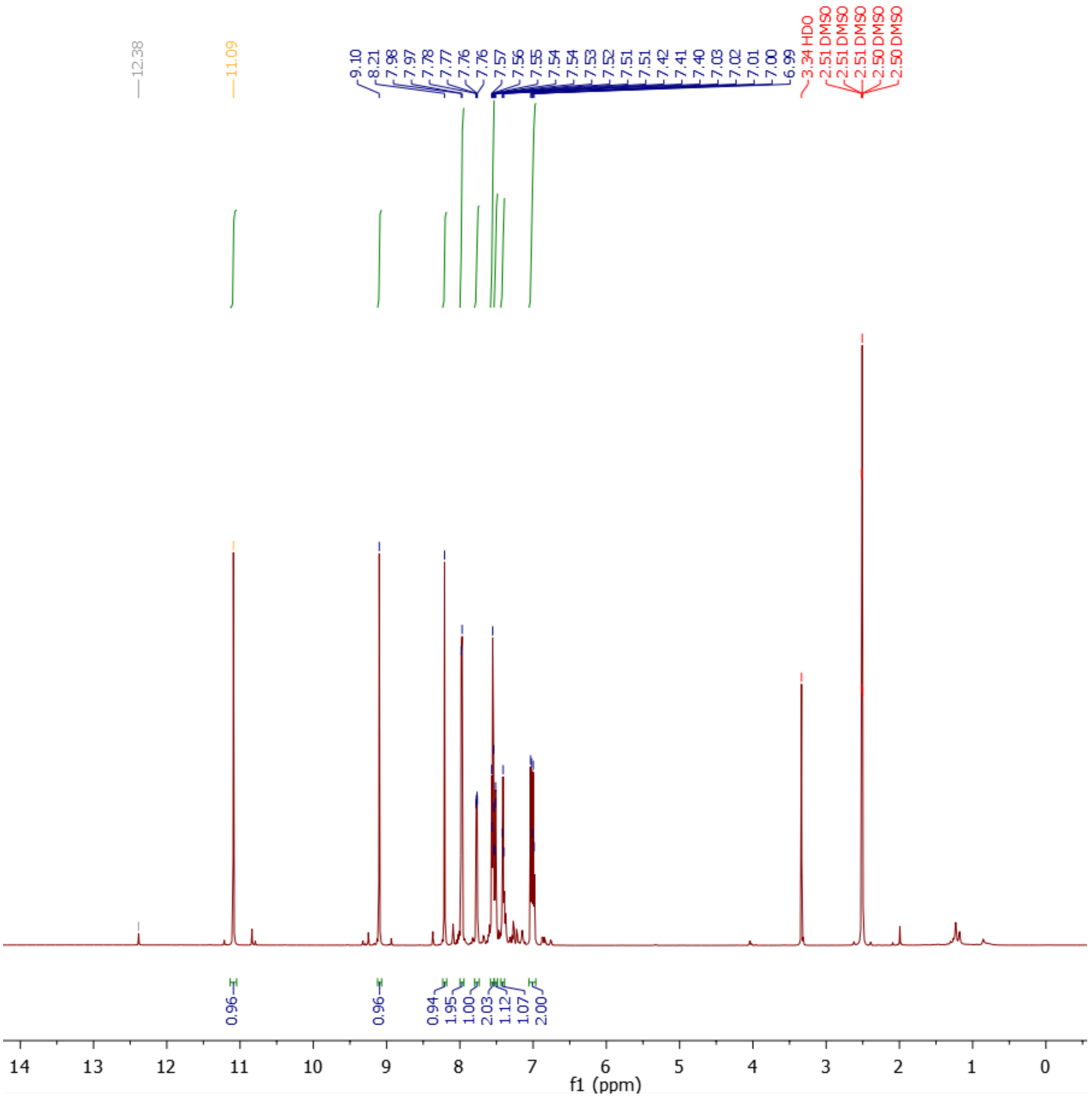

^{1}H NMR of compound **CK-22** (600 MHz, DMSO-*d*6).

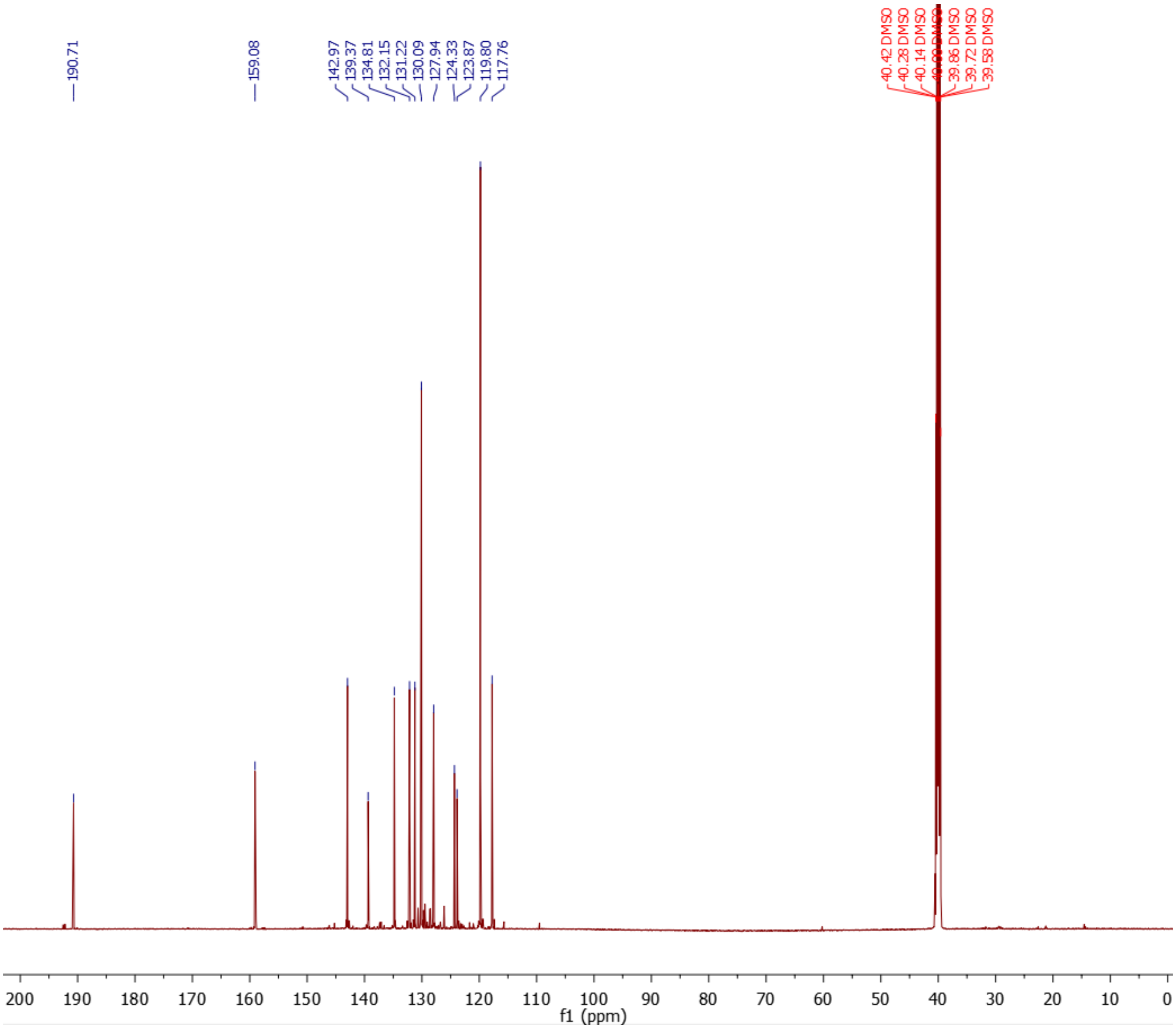

^{13}**C NMR** of compound **CK-22** (151 MHz, DMSO-*d*$_6$).

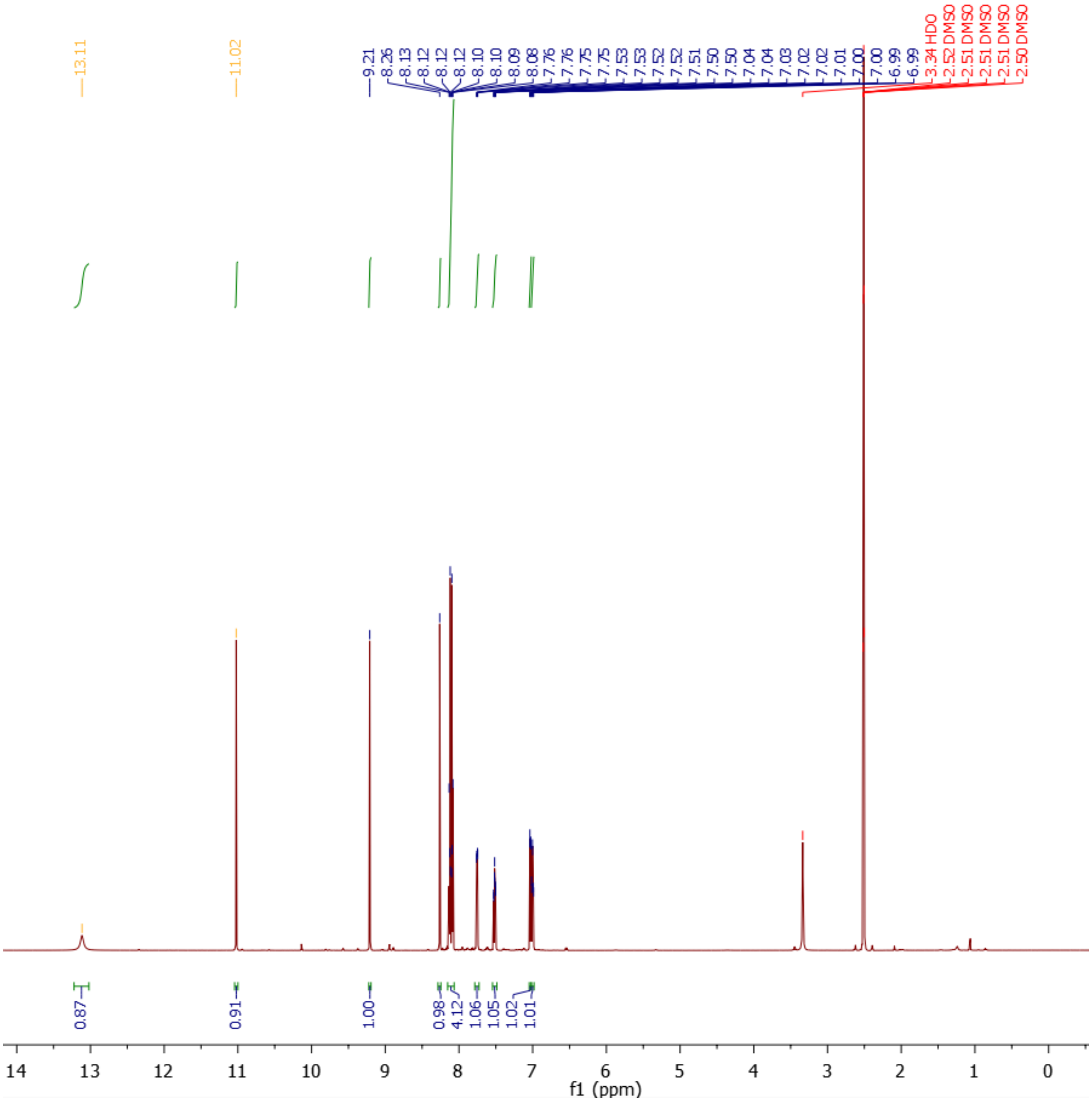

^{1}H NMR of compound **CK-24** (600 MHz, DMSO-*d*$_6$).

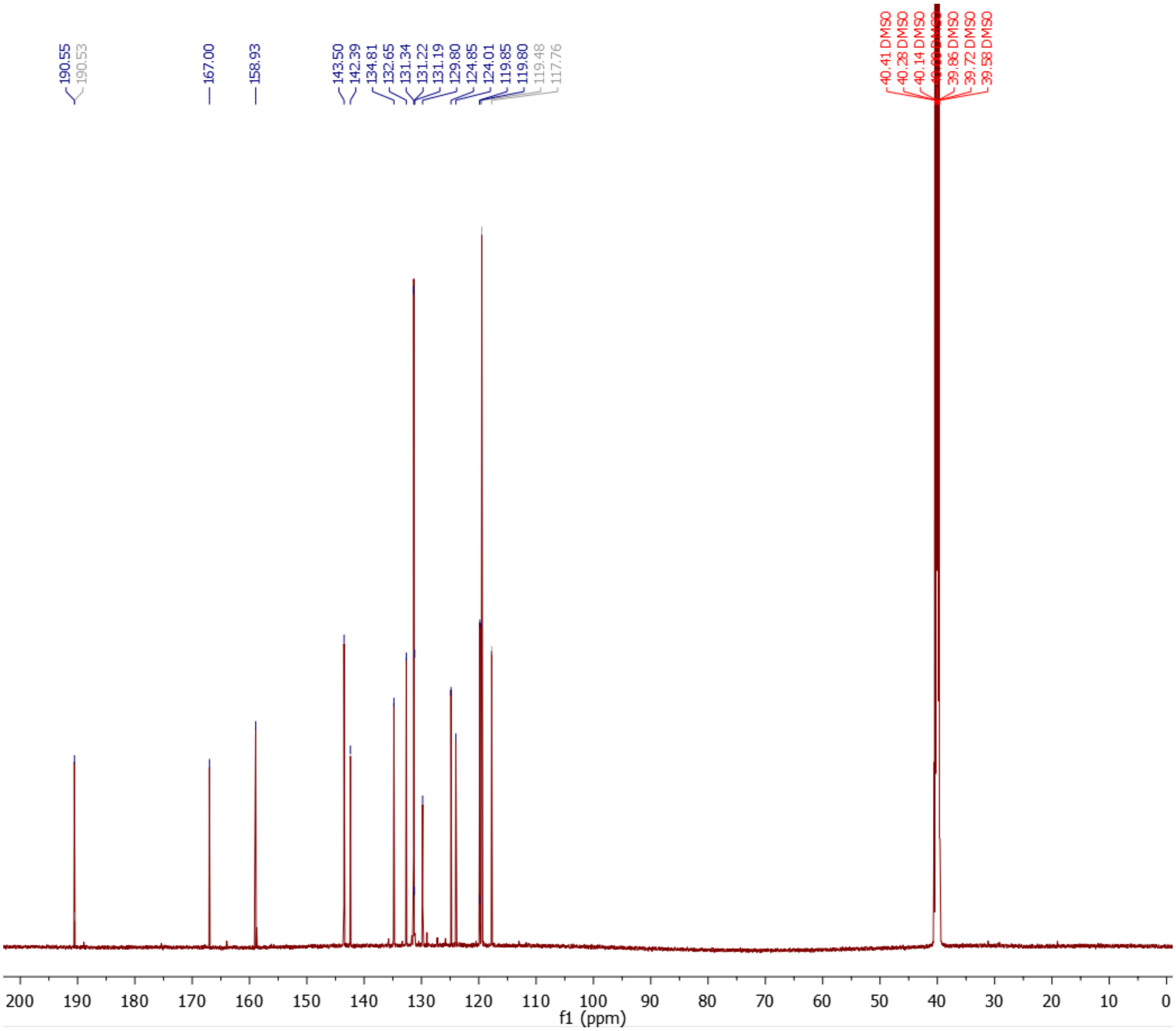

^{13}C NMR of compound **CK-24** (151 MHz, DMSO-*d*$_6$).

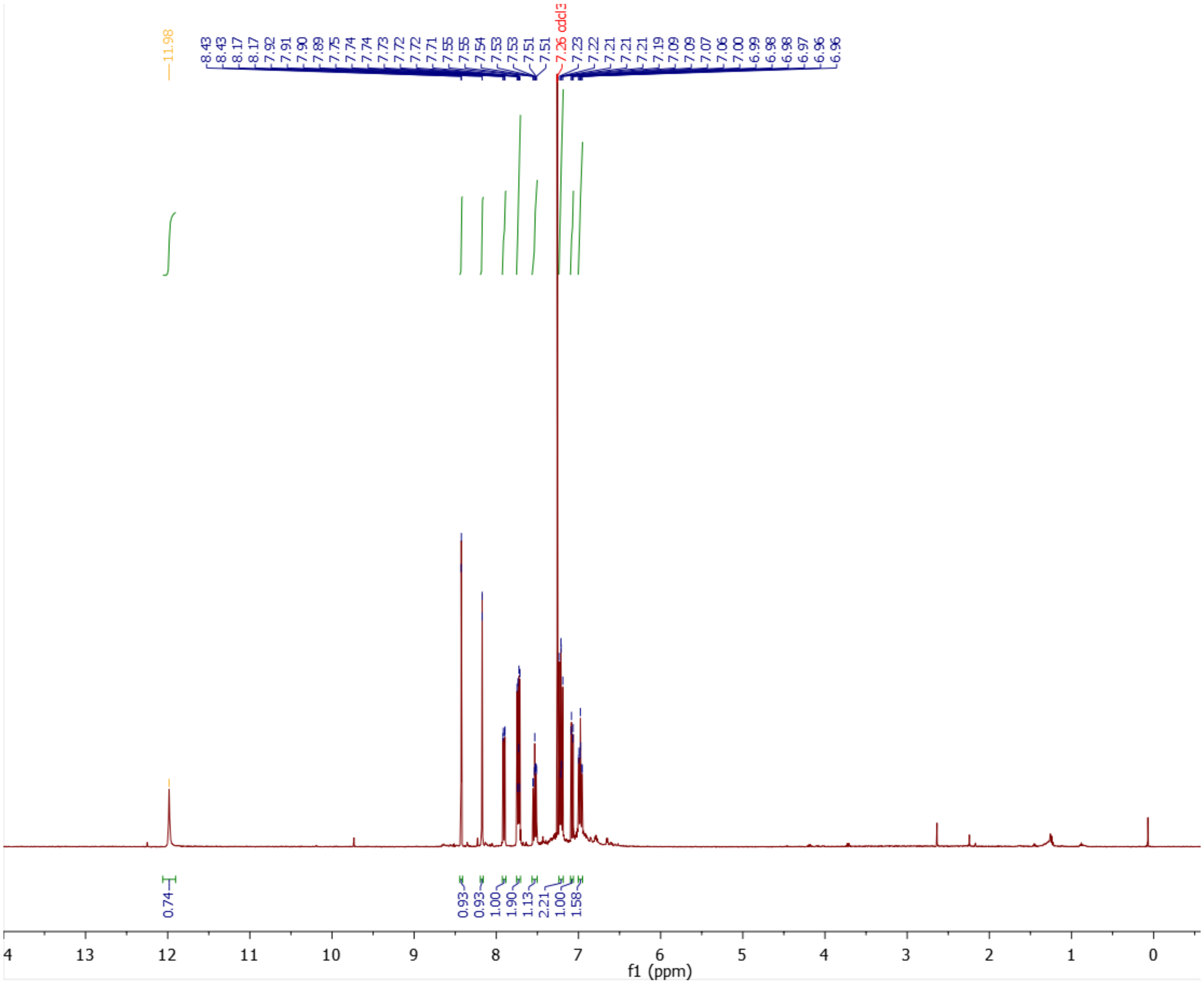

^{1}H NMR of compound **CK-26** (400 MHz, $CDCl_3$).

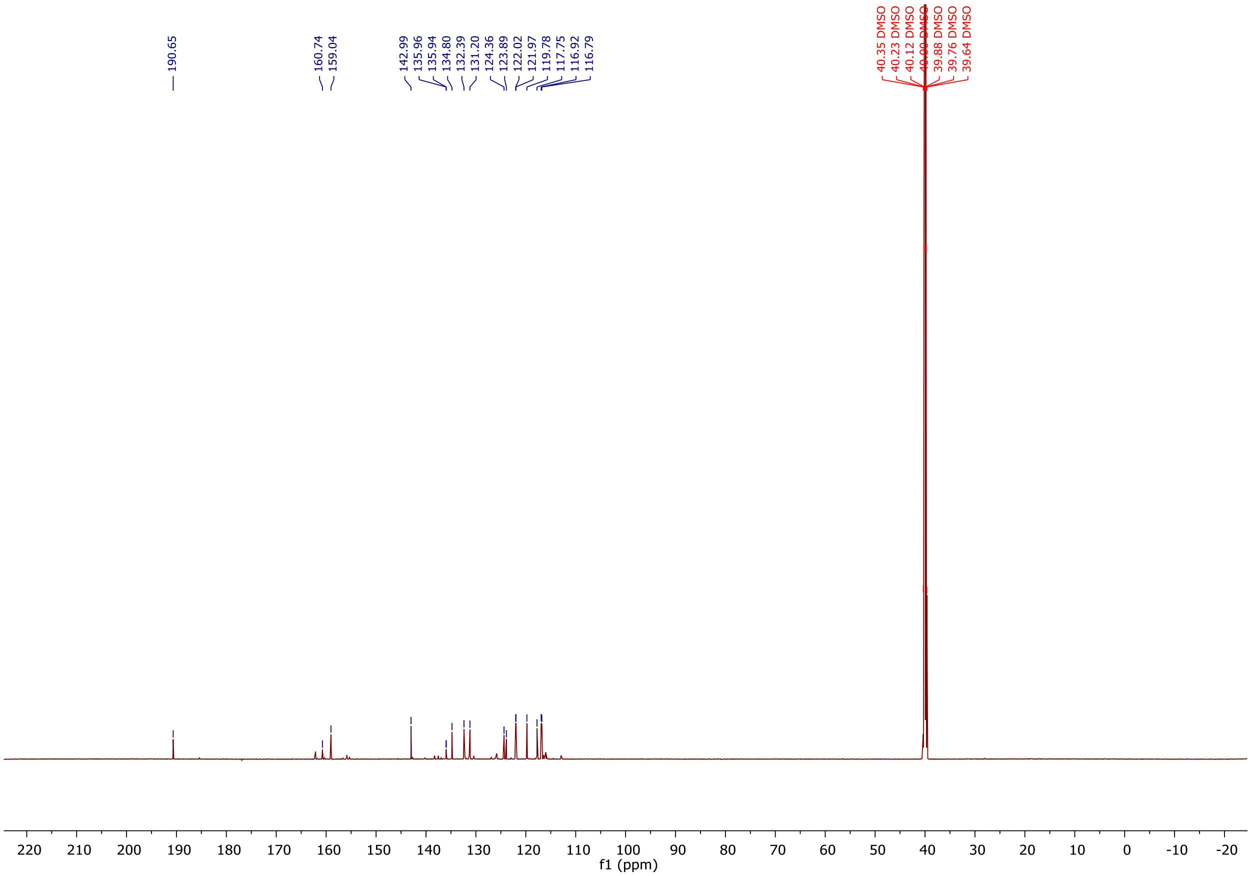

^{13}C NMR of compound **CK-26** (176 MHz, DMSO-d_6).

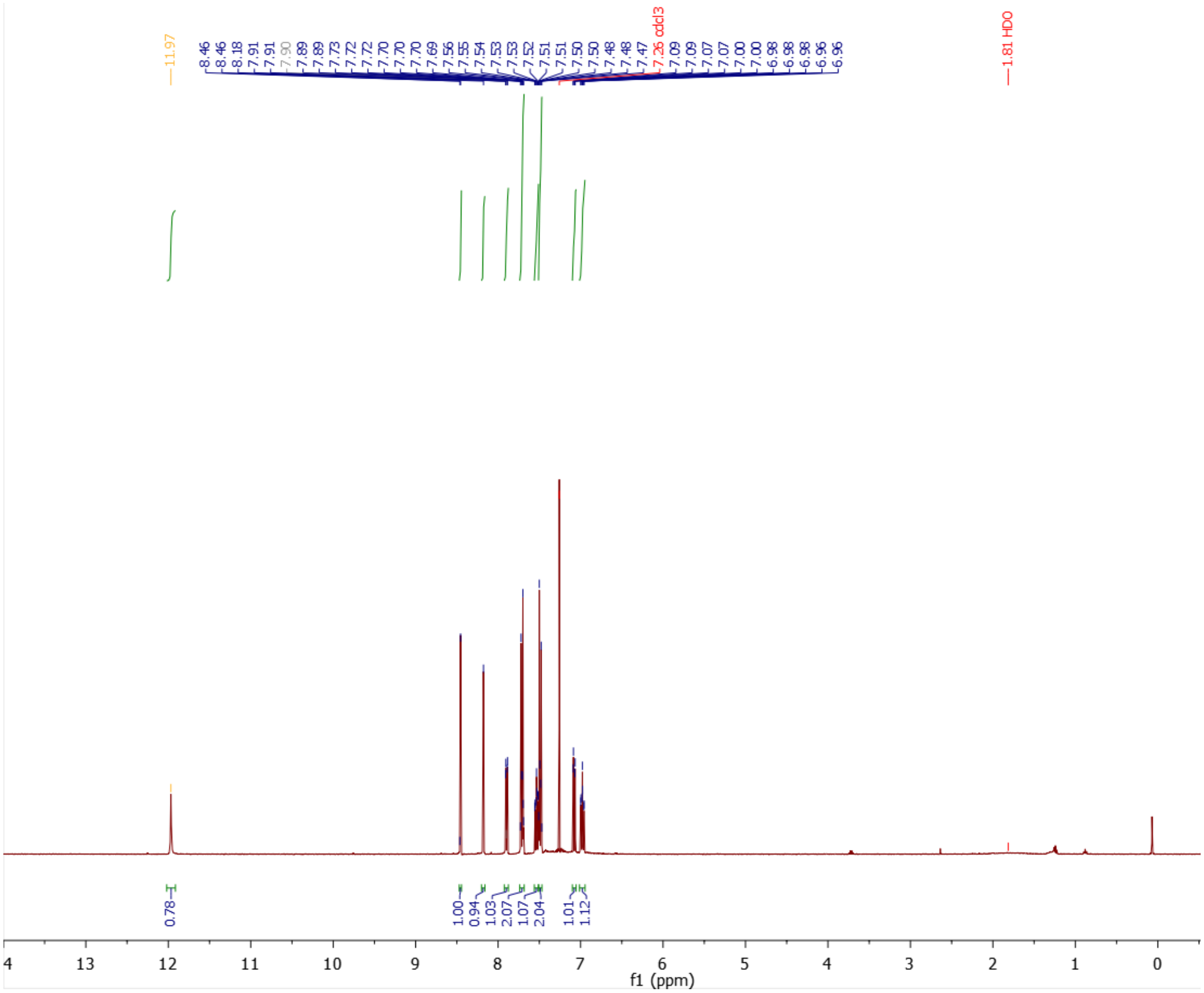

[1]**H NMR** of compound **CK-27** (400 MHz, $CDCl_3$).

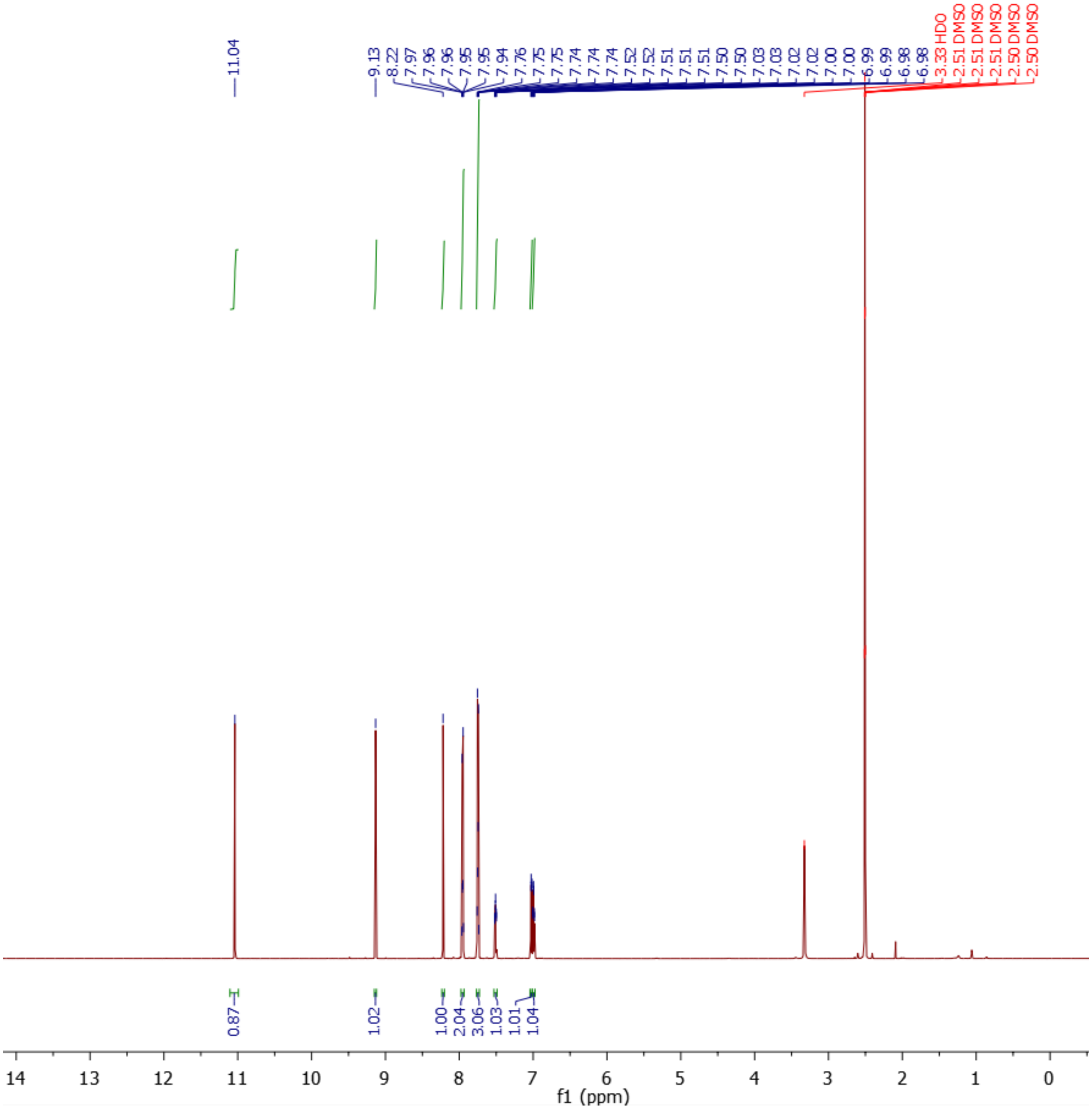

^{1}H NMR of compound **CK-28** (700 MHz, DMSO-d_6).

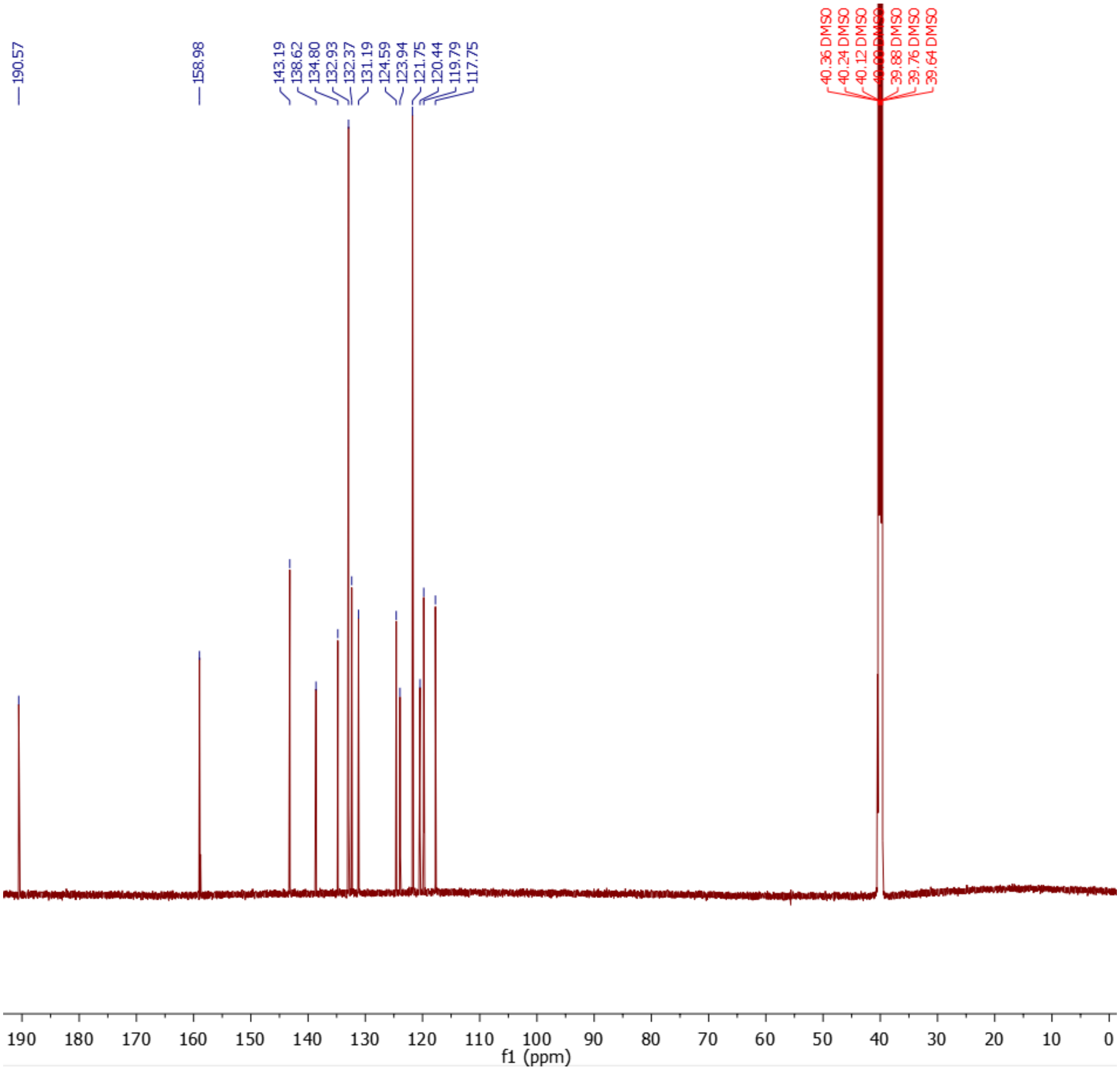

^{13}C NMR of compound **CK-28** (176 MHz, DMSO-d_6).

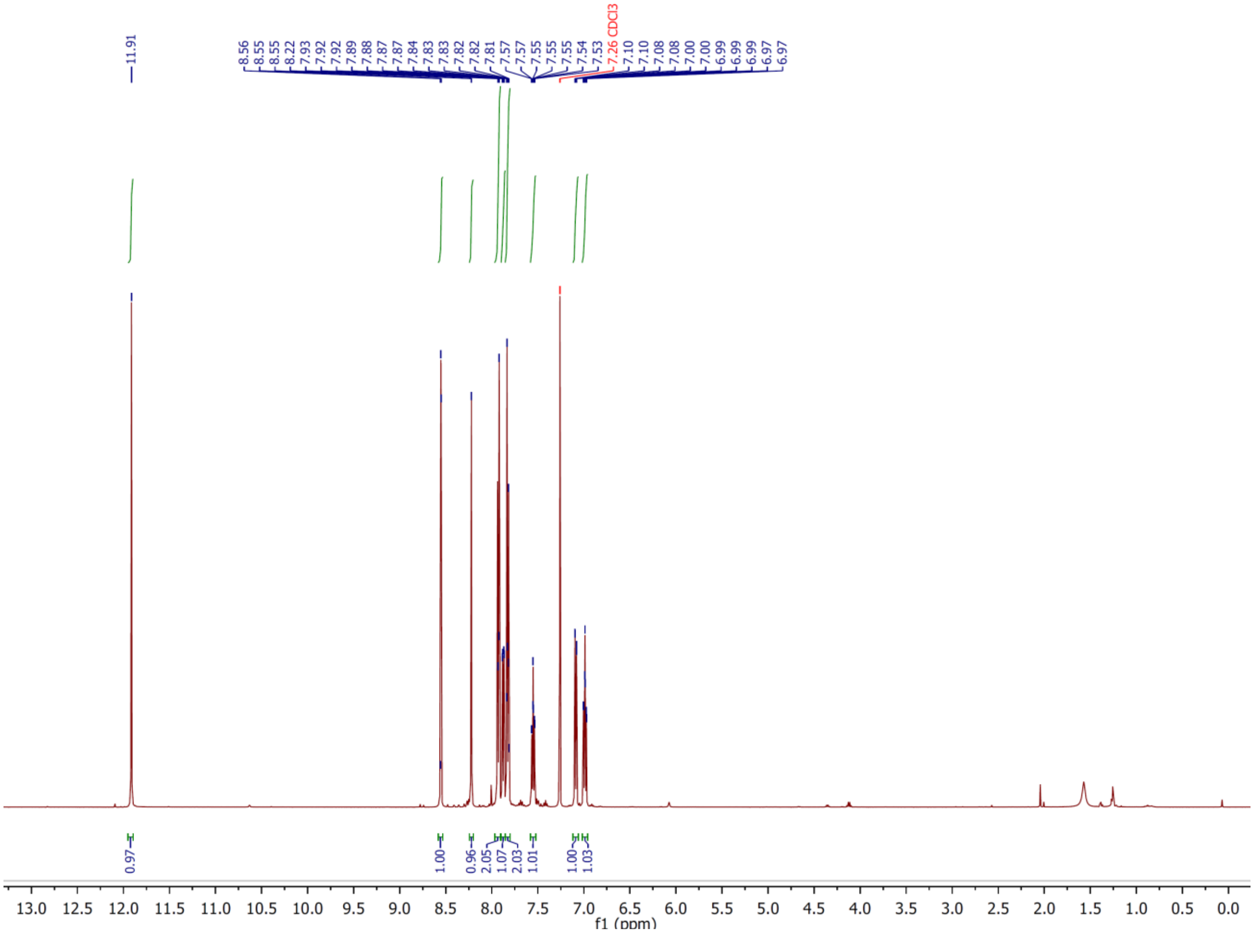

[1]**H NMR** of compound **CK-38** (500 MHz, $CDCl_3$).

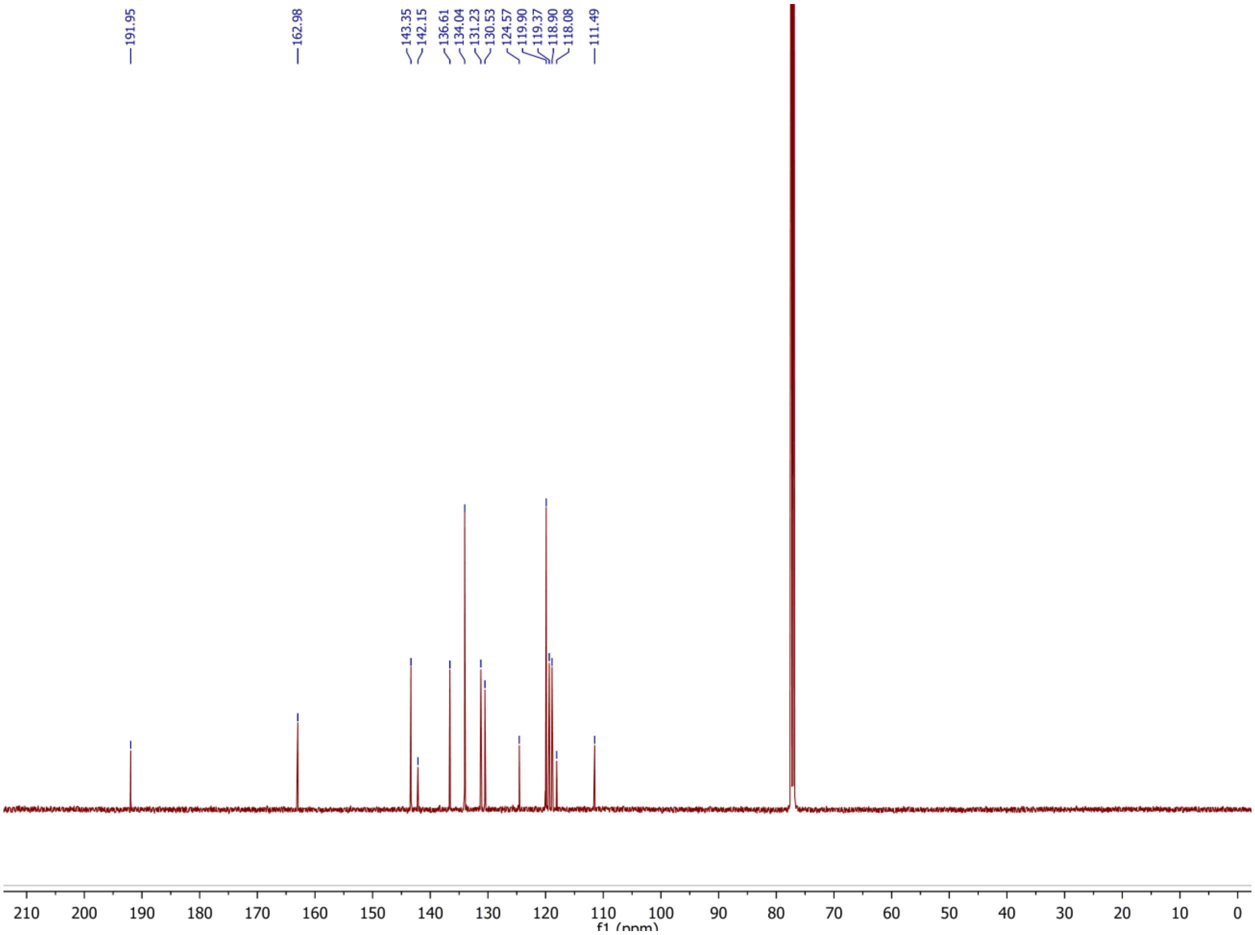

^{13}C NMR of compound **CK-38** (126 MHz, $CDCl_3$).

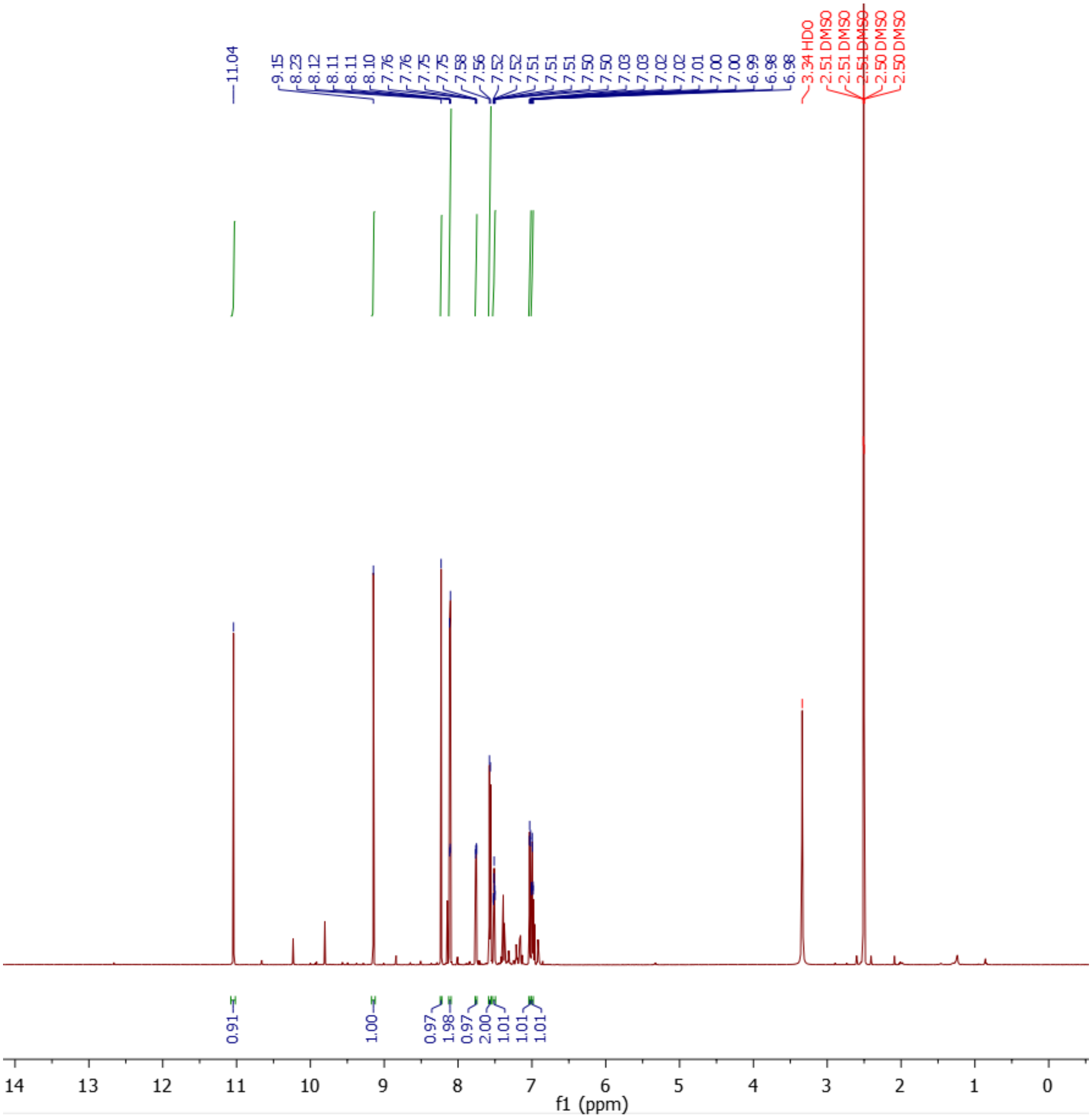

^{1}H NMR of compound **CK-39** (700 MHz, DMSO-d_6).

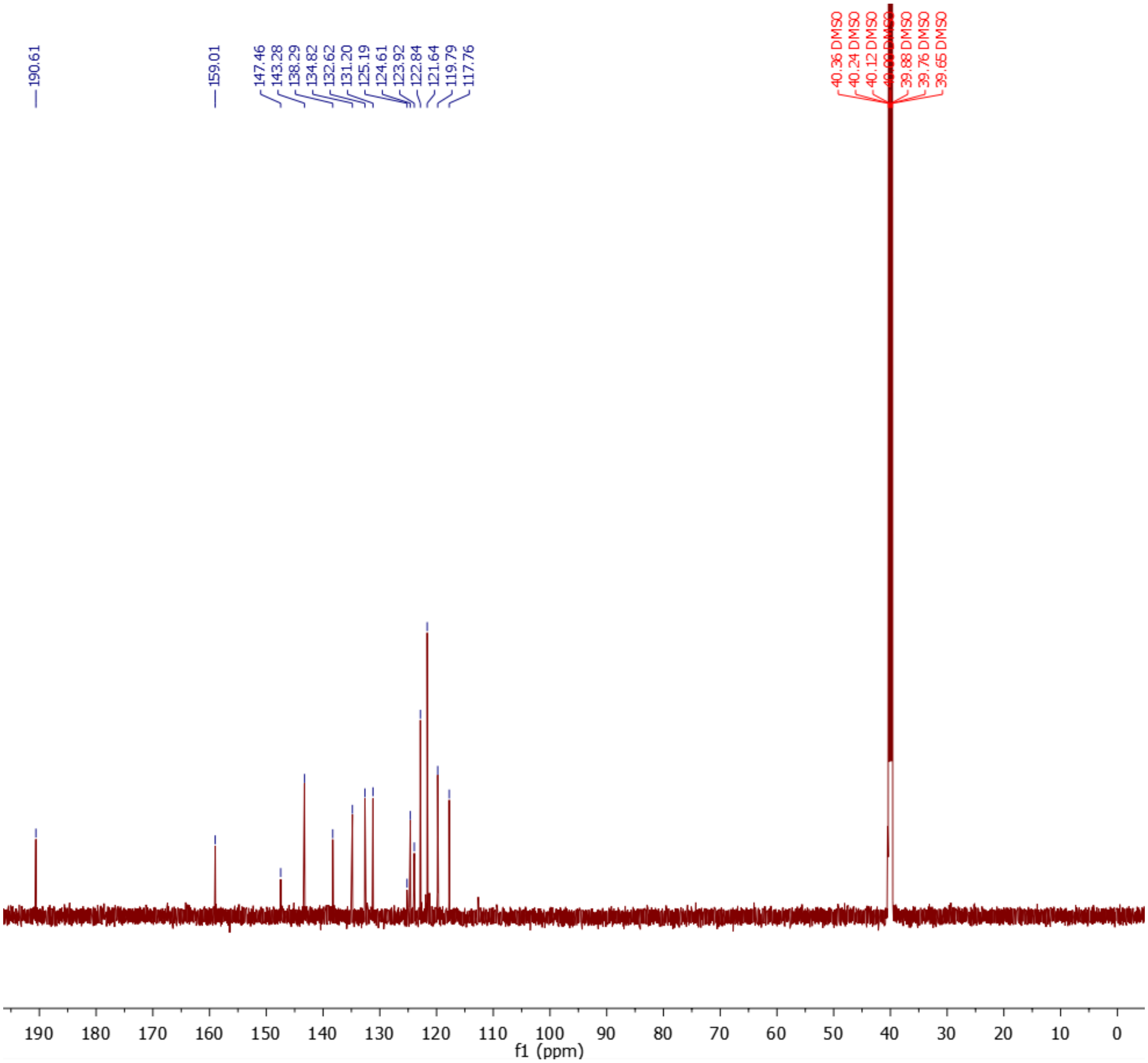

^{13}C NMR of compound **CK-39** (176 MHz, DMSO-d_6).

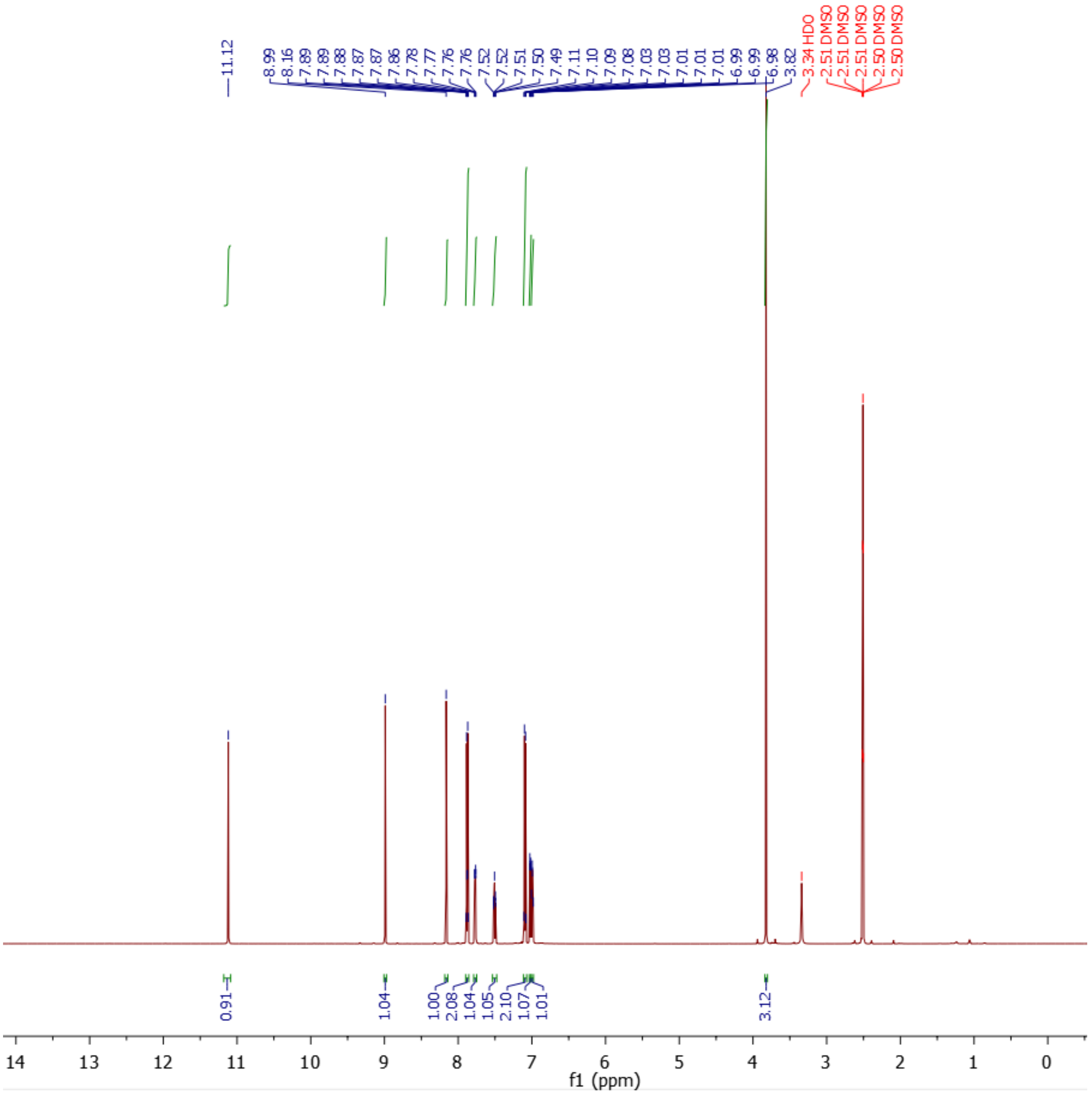

^{1}H NMR of compound **CK-40** (600 MHz, DMSO-d_6).

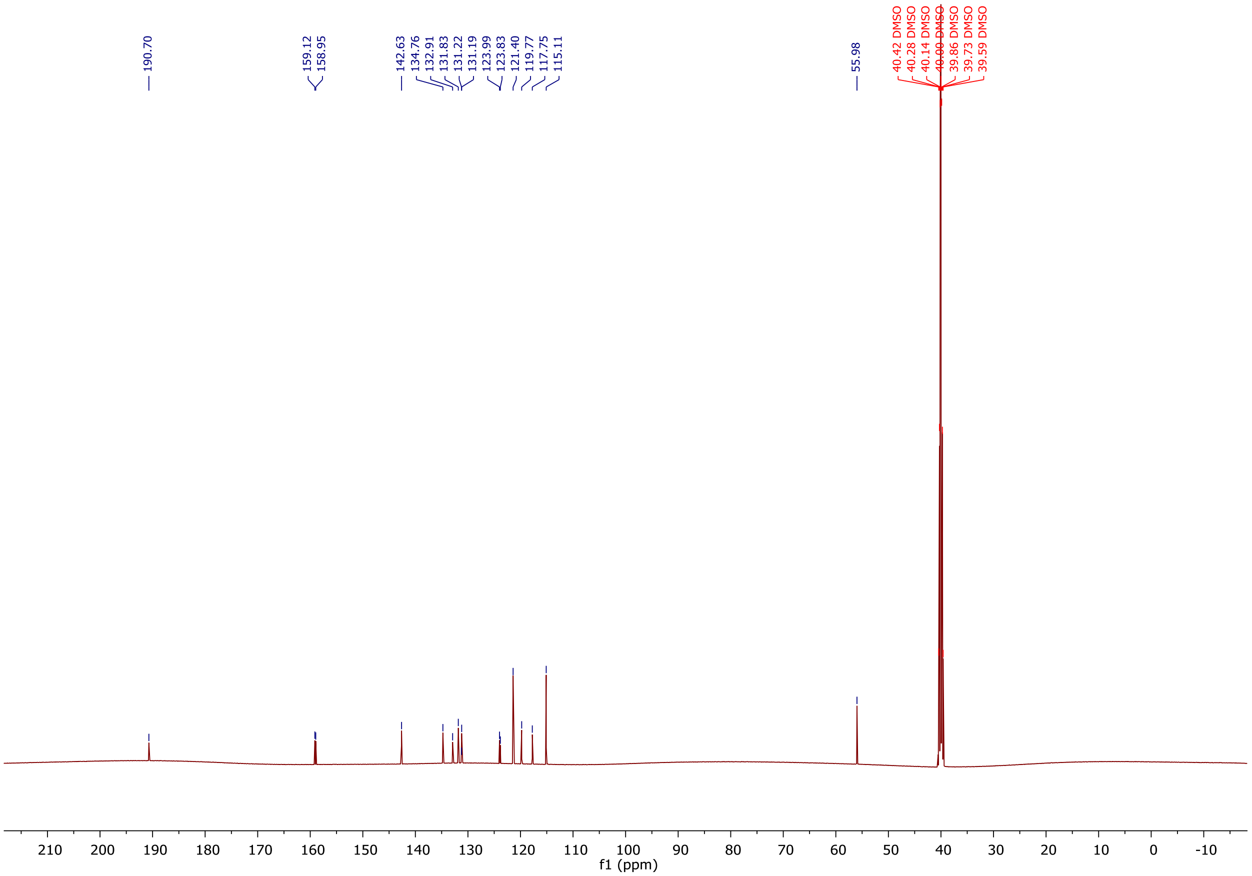

^{13}C NMR of compound **CK-40** (151 MHz, DMSO-d_6).

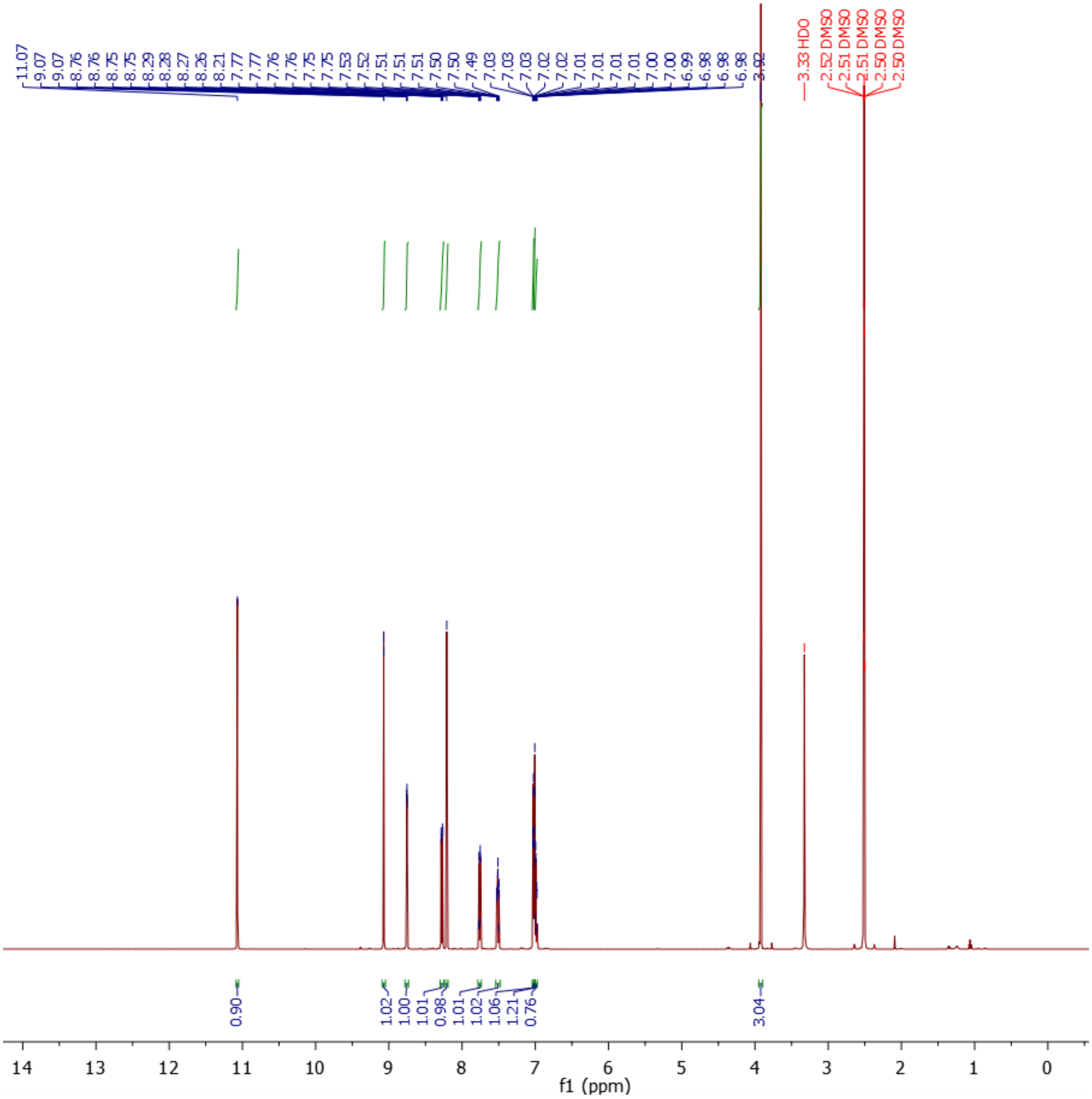

^{1}H NMR of compound **CK-47** (500 MHz, DMSO-d_6).

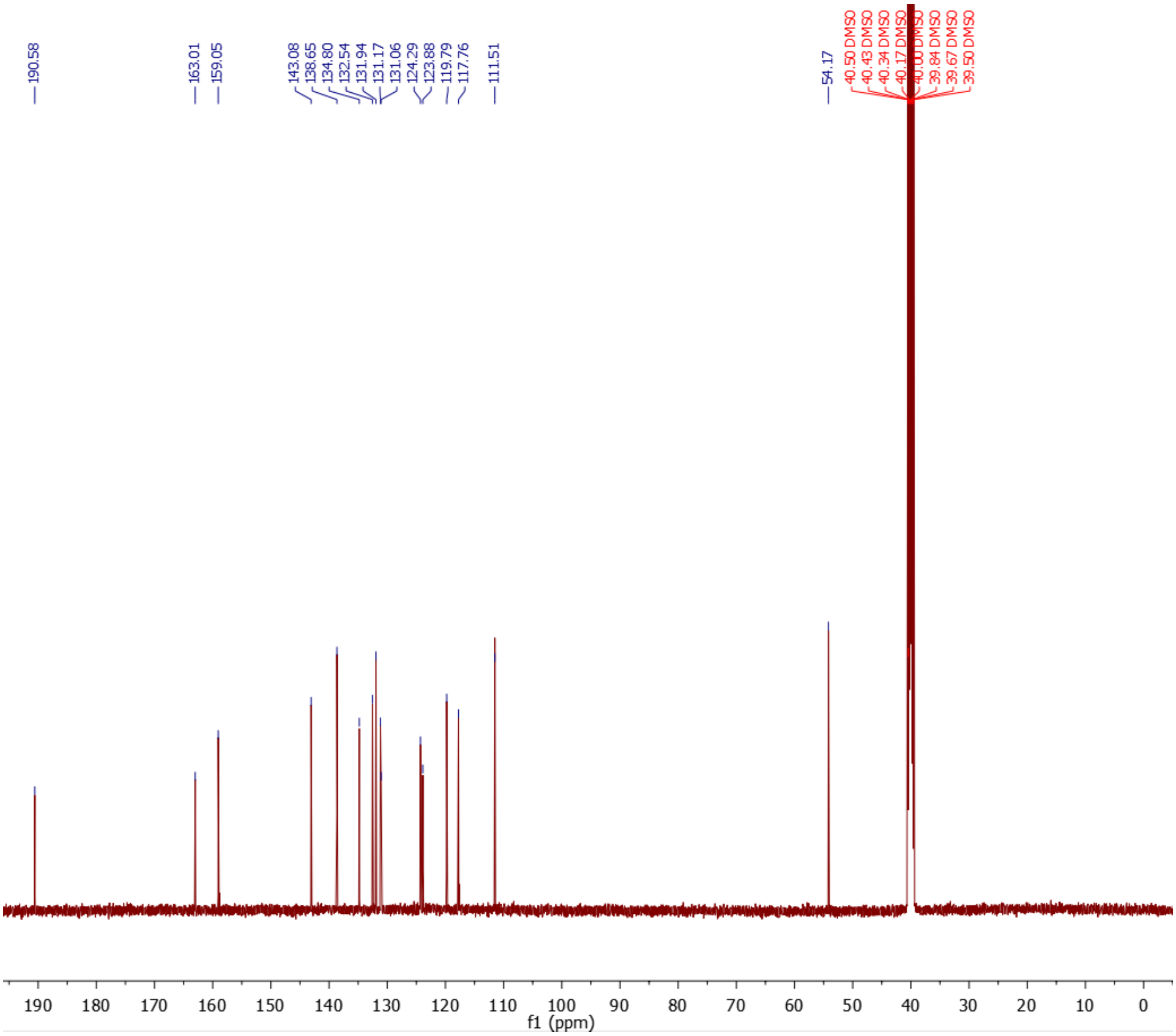

^{13}C NMR of compound **CK-47** (126 MHz, DMSO-d_6).

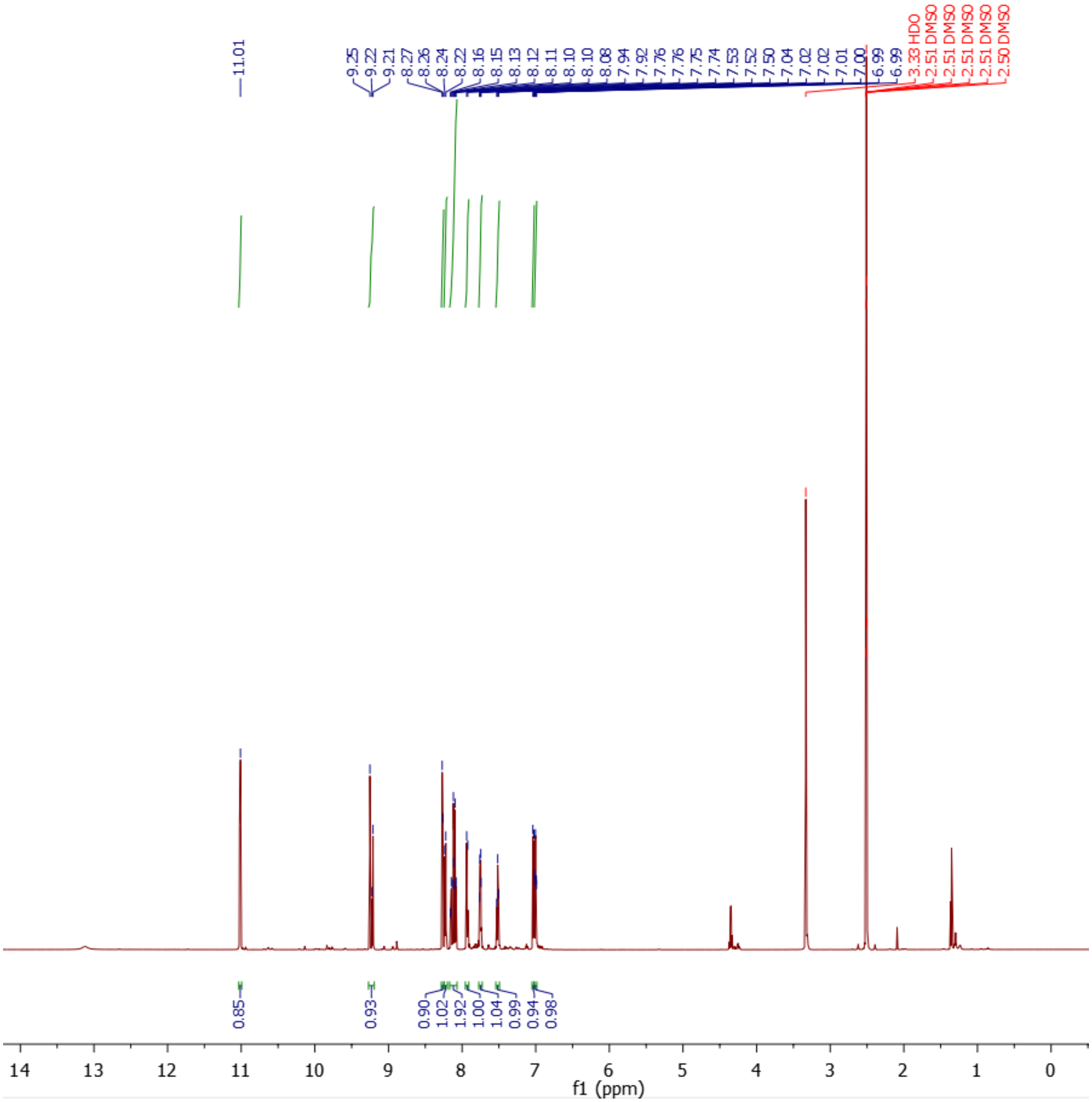

[1]**H NMR** of compound **CK-48** (600 MHz, DMSO-d_6).

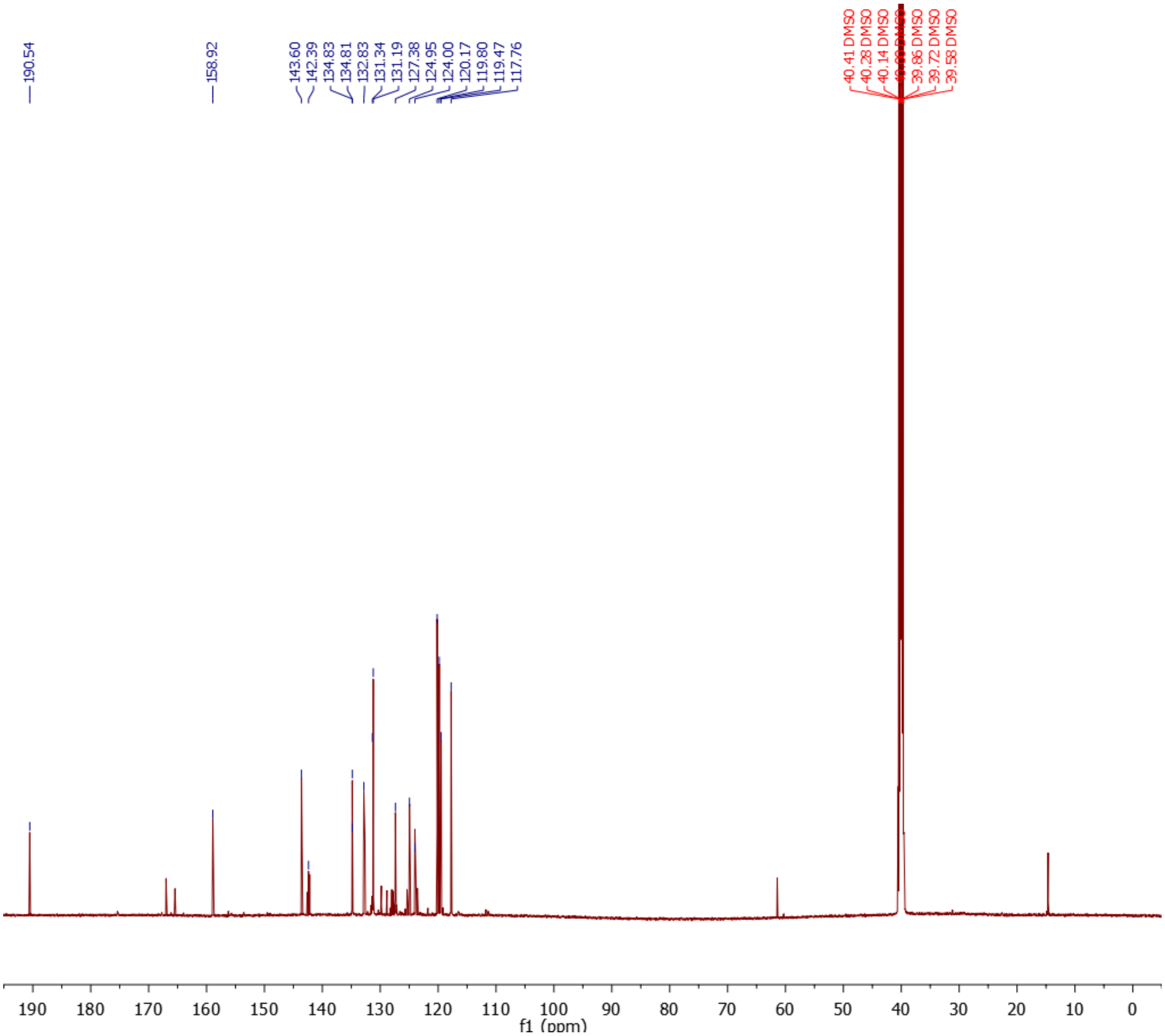

^{13}C NMR of compound **CK-48** (151 MHz, DMSO-d_6).

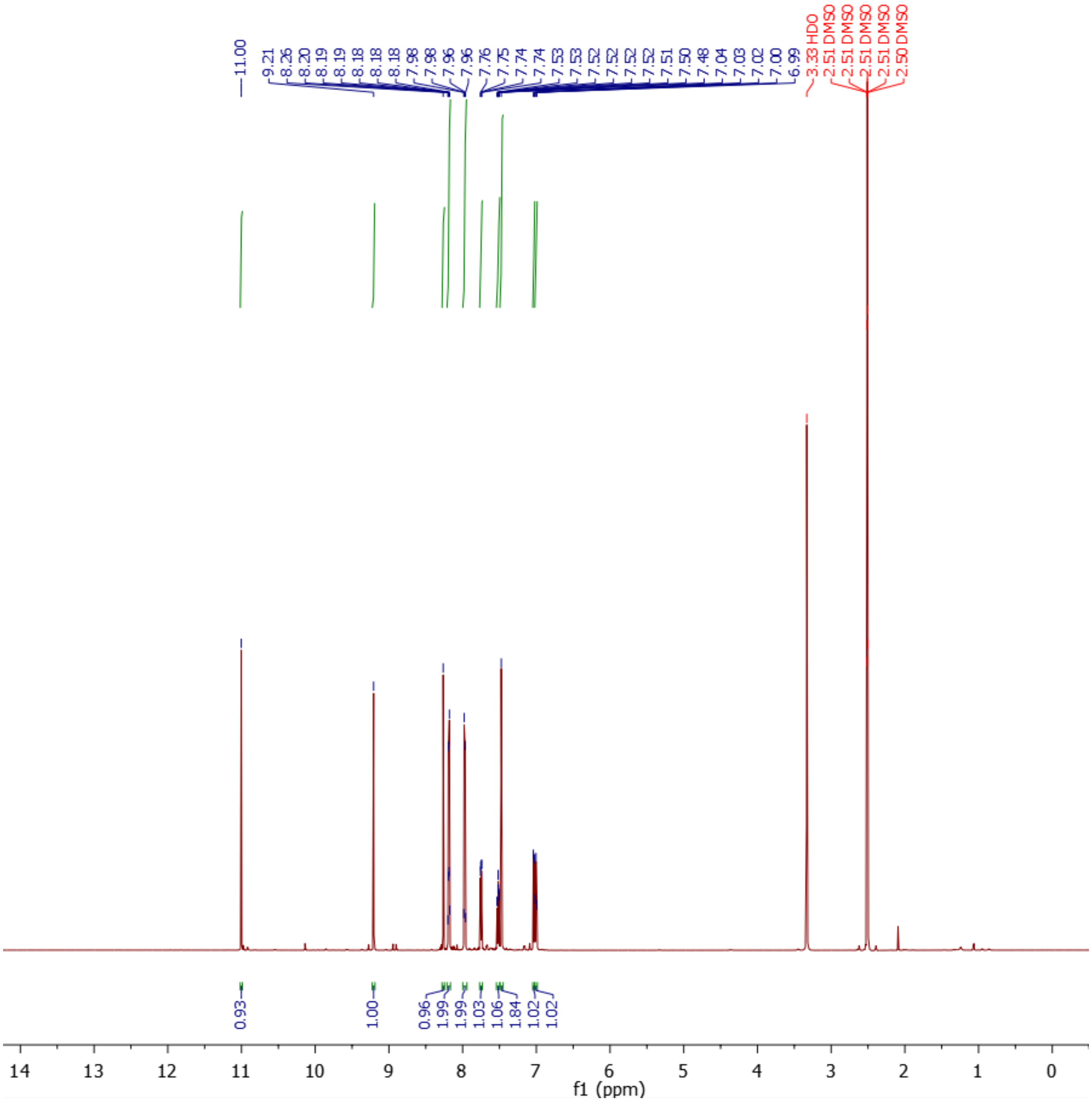

^{1}H NMR of compound **CK-49** (600 MHz, DMSO-d_6).

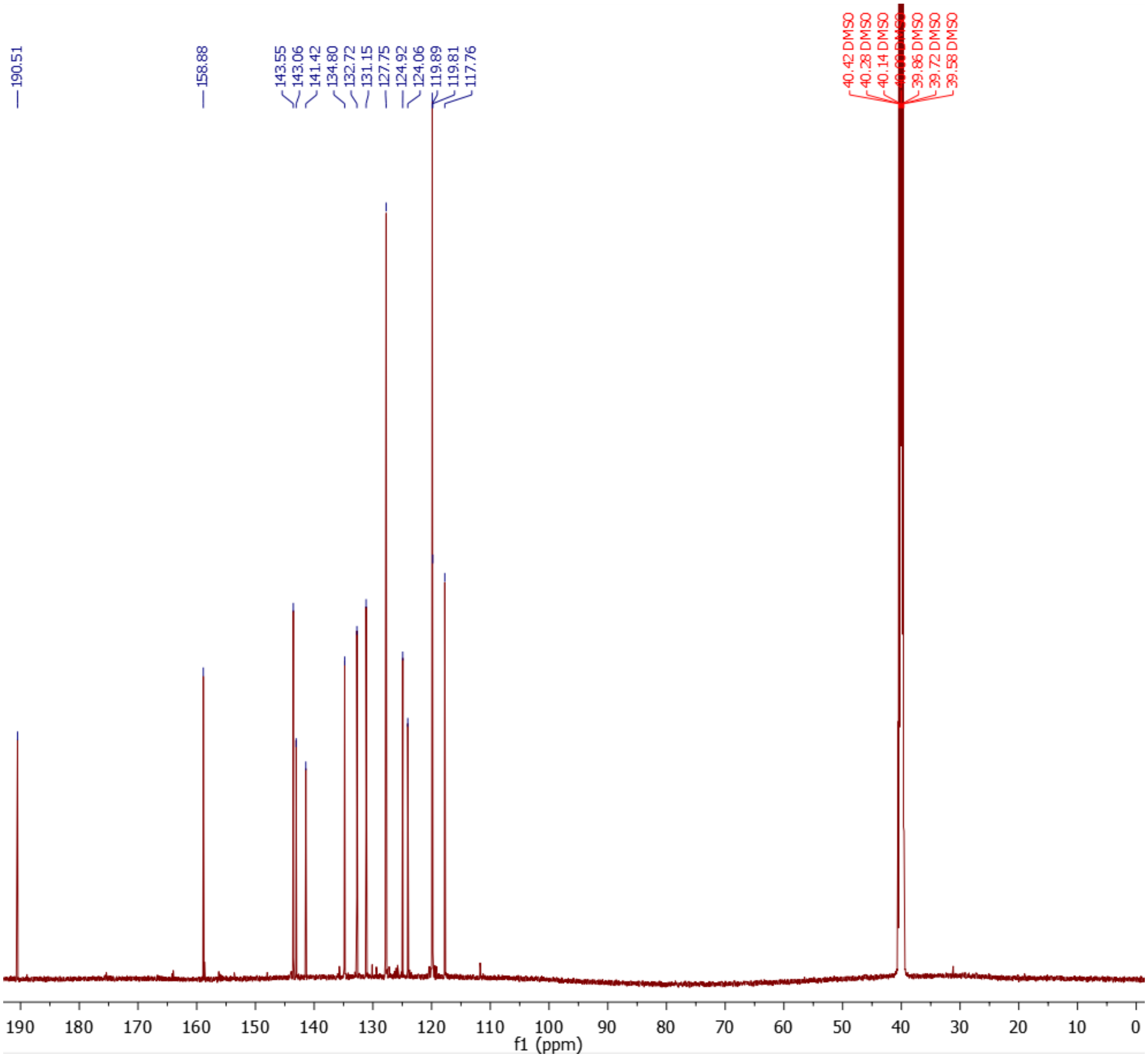

^{13}**C NMR** of compound **CK-49** (151 MHz, DMSO-d_6).

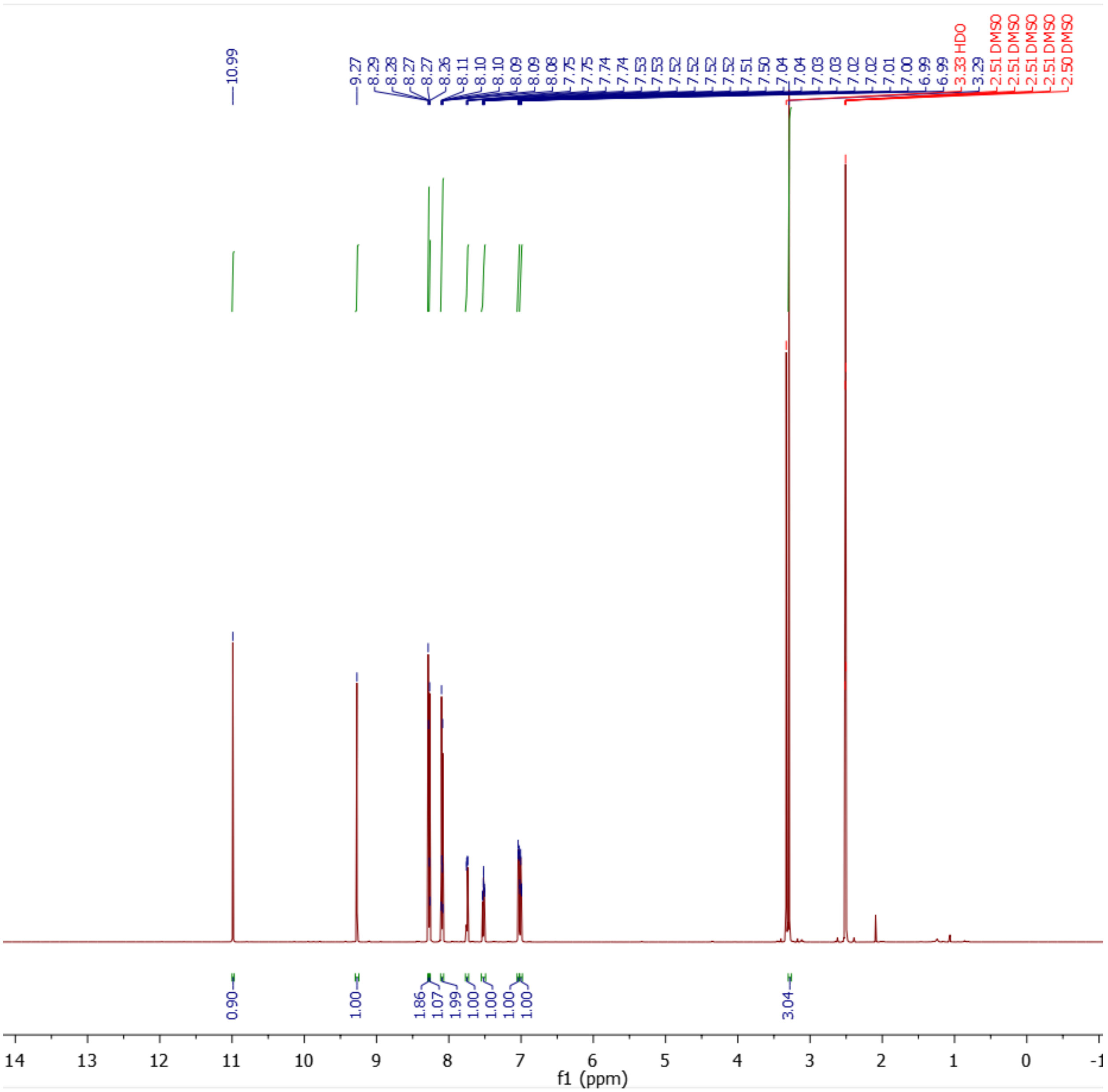

^{1}H NMR of compound **CK-50** (600 MHz, DMSO-d_6).

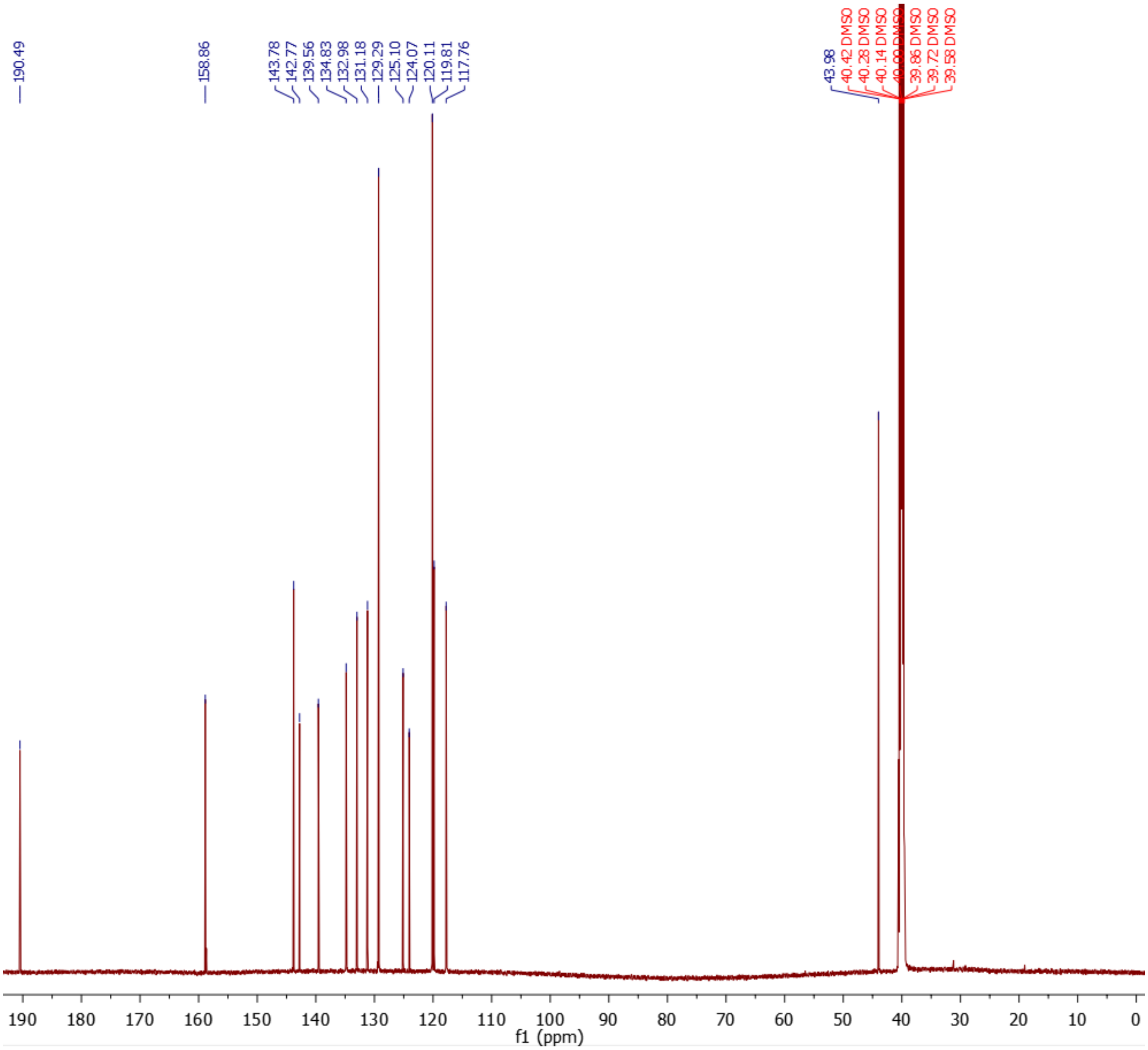

^{13}**C NMR** of compound **CK-50** (151 MHz, DMSO-d_6).

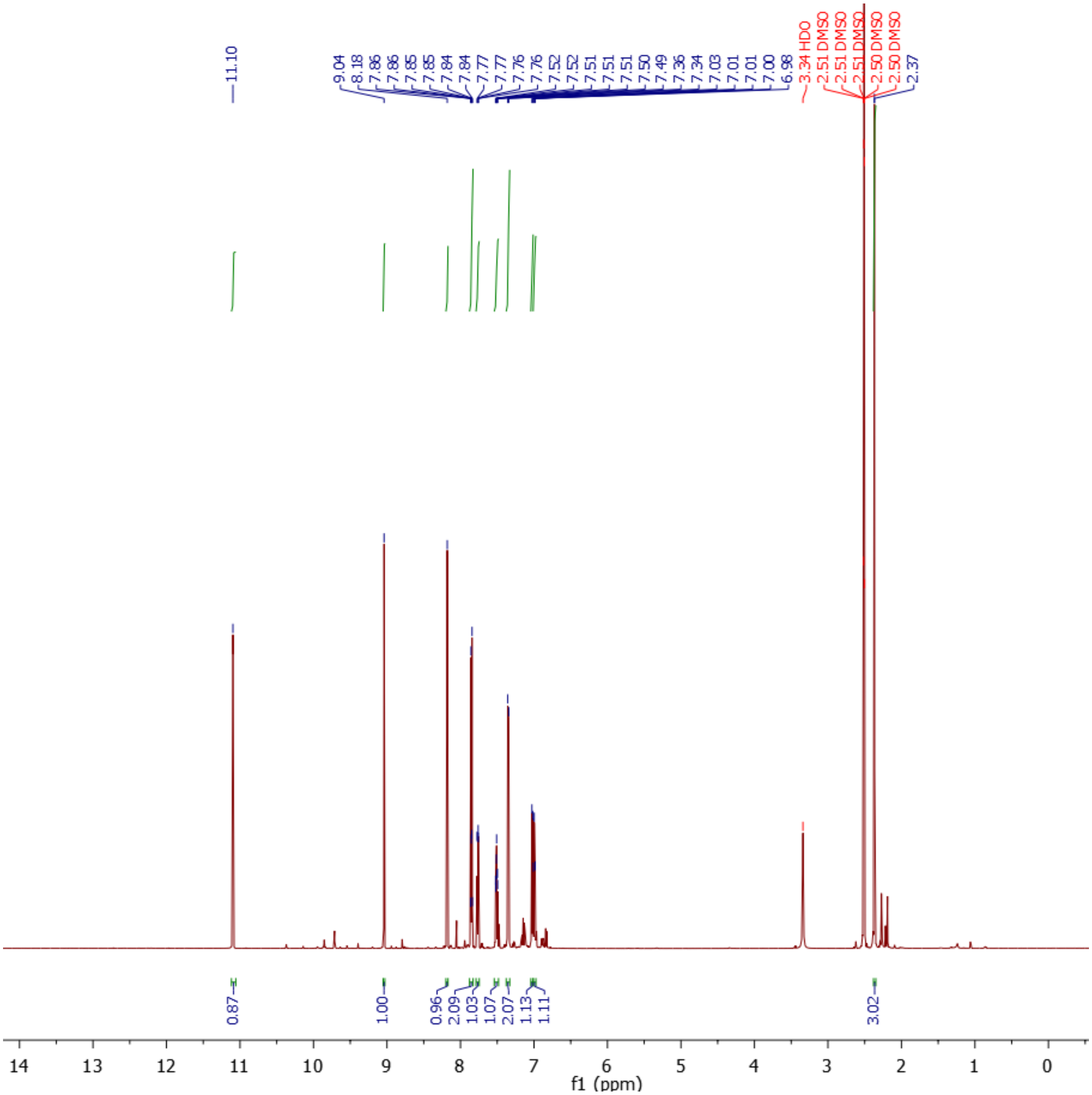

[1]**H NMR** of compound **CK-51** (600 MHz, DMSO-d_6).

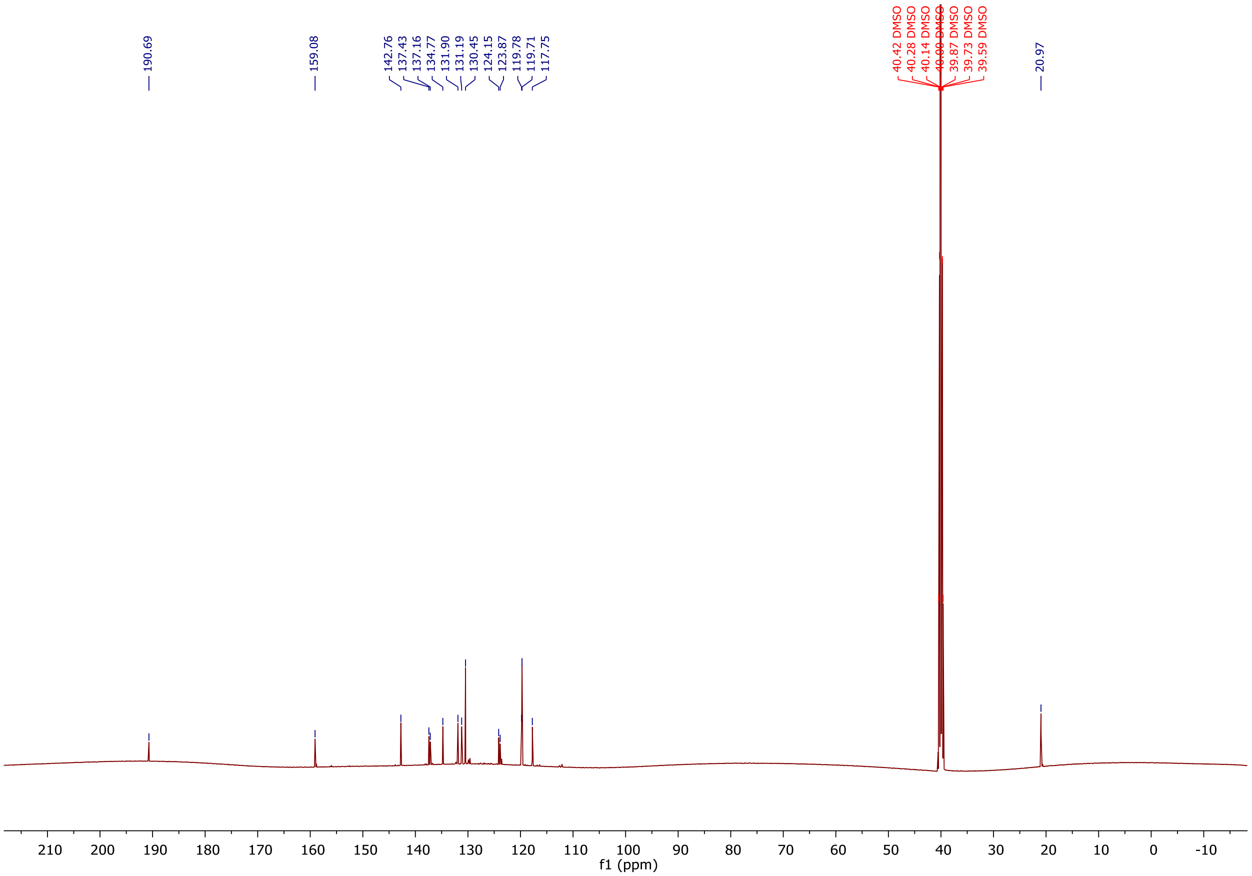

^{13}C NMR of compound **CK-51** (151 MHz, DMSO-d_6).

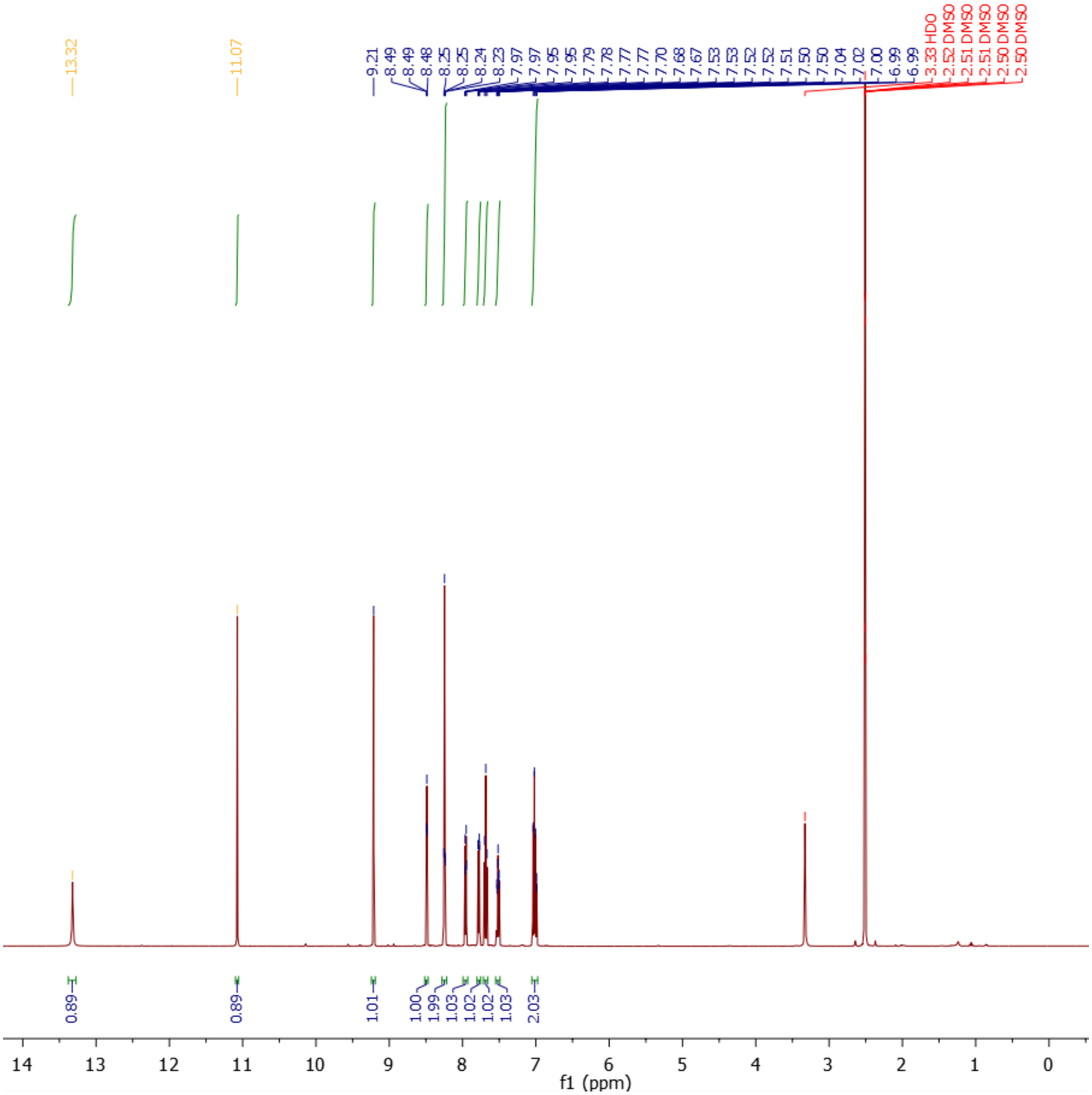

[1]**H NMR** of compound **CK-23** (500 MHz, DMSO-d_6).

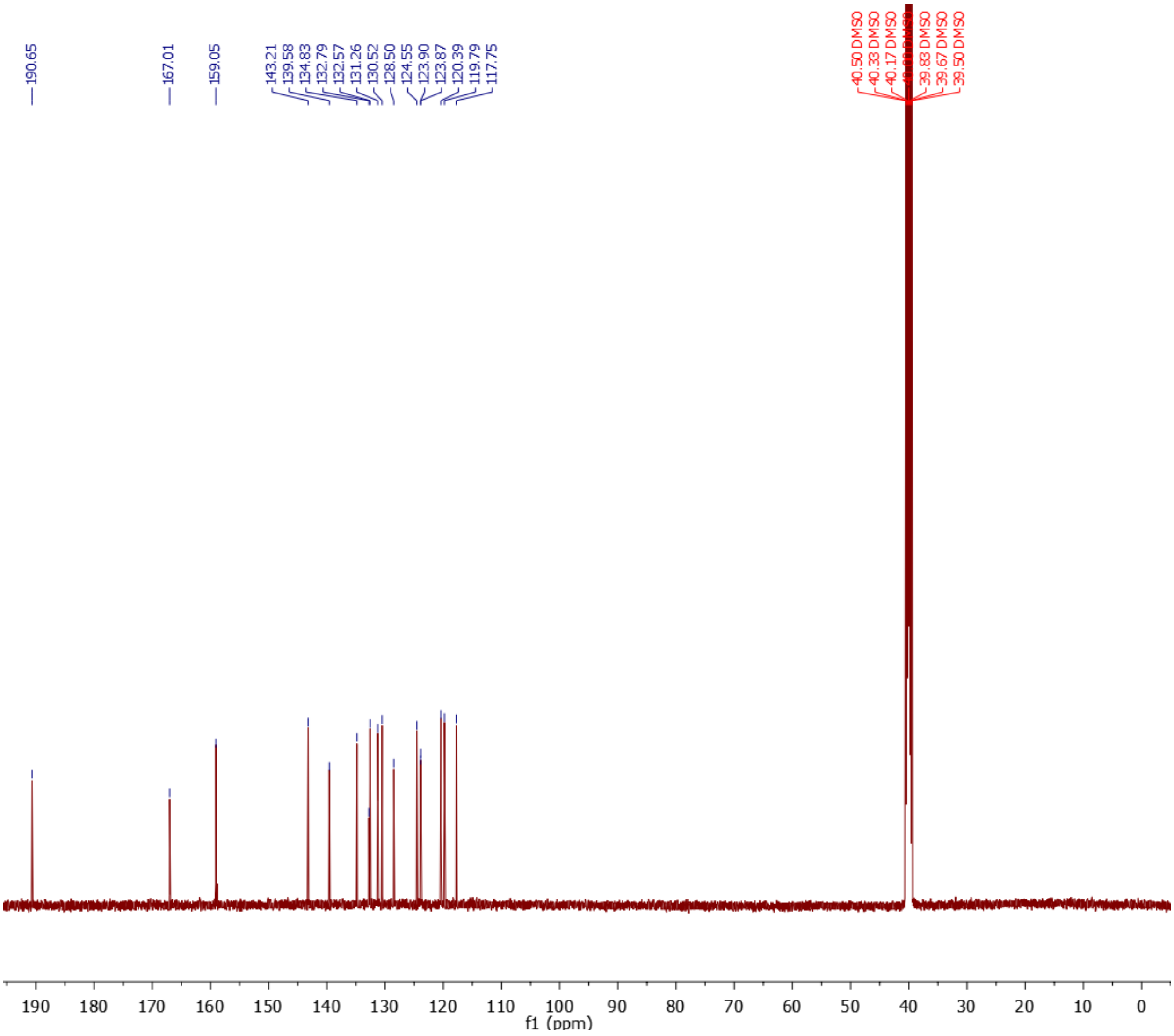

^{13}C NMR of compound **CK-23** (126 MHz, DMSO-*d*$_6$).

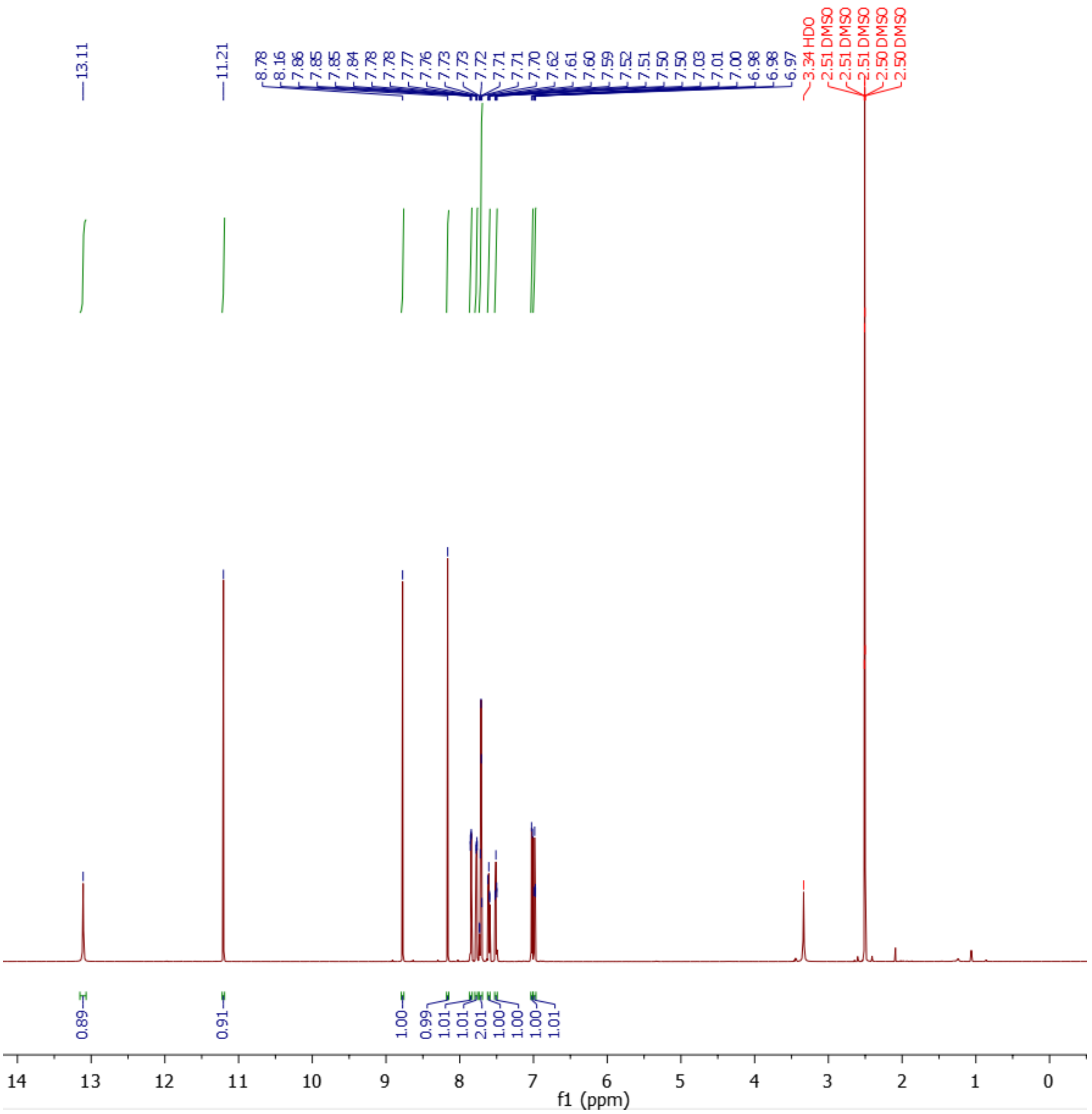

^{1}H NMR of compound **CK-25** (700 MHz, DMSO-d_6).

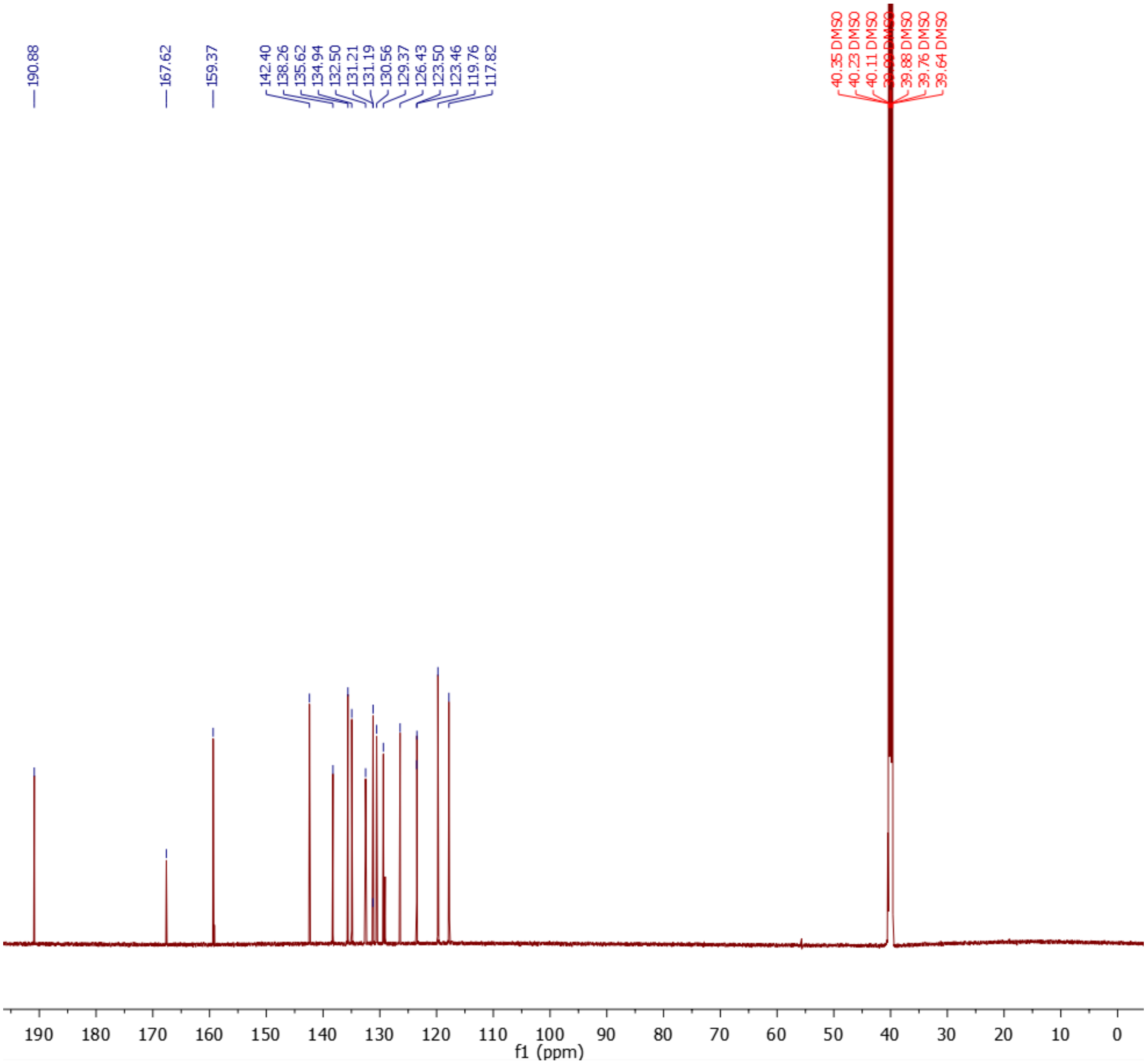

^{13}**C NMR** of compound **CK-25** (176 MHz, DMSO-d_6).

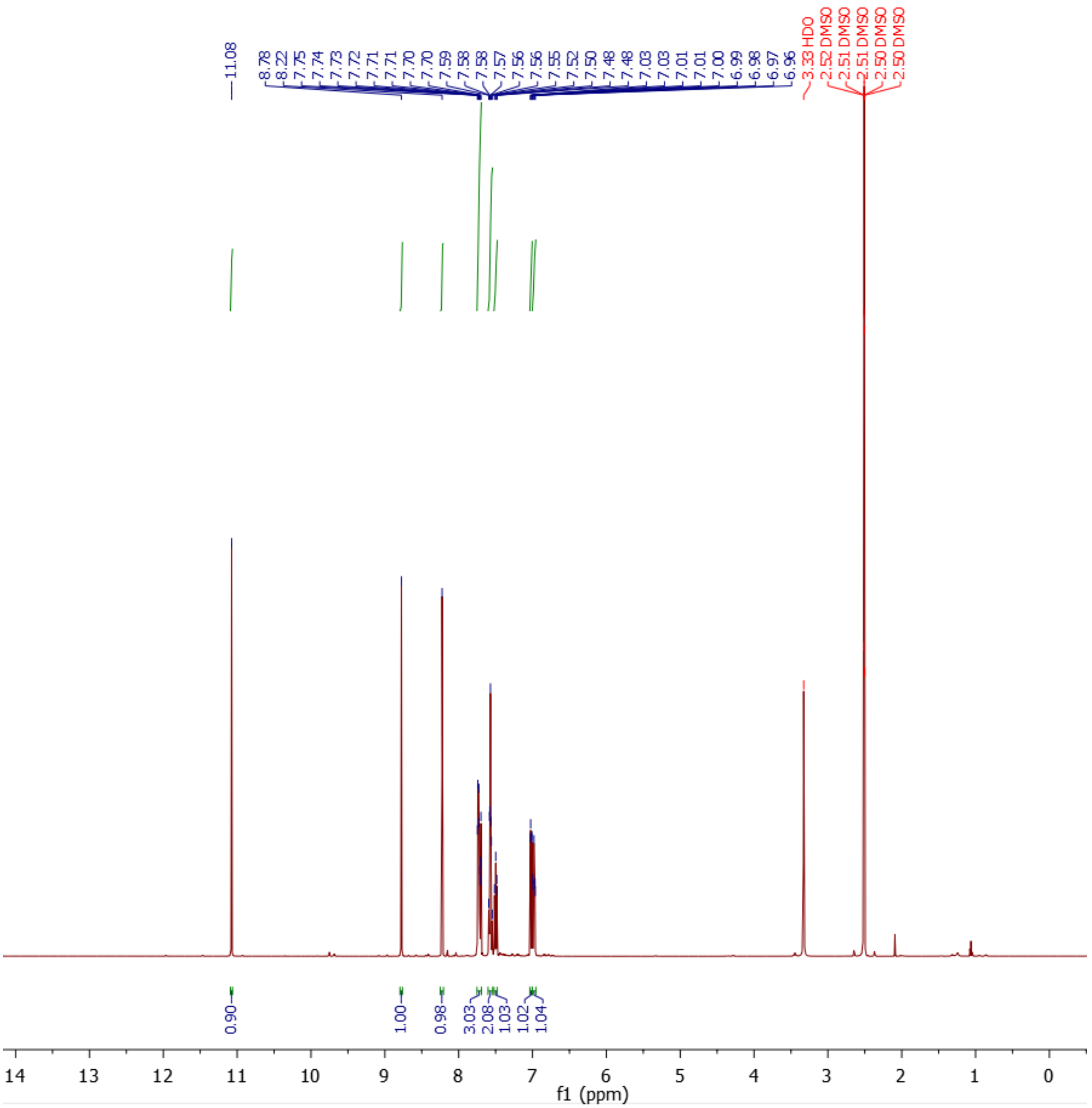

^{1}H NMR of compound **CK-44** (500 MHz, DMSO-*d*6).

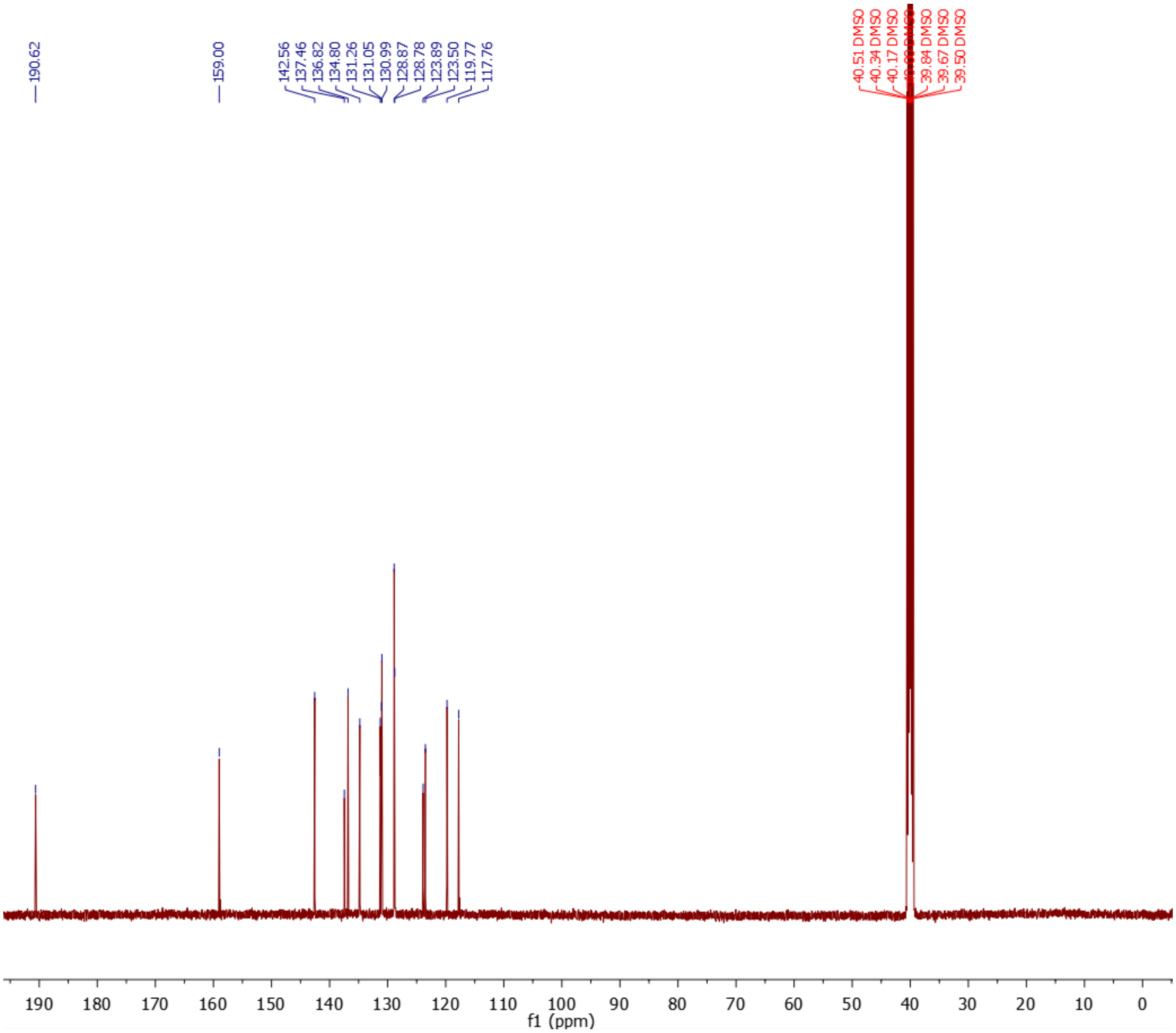

^{13}C NMR of compound **CK-44** (126 MHz, DMSO-d_6).

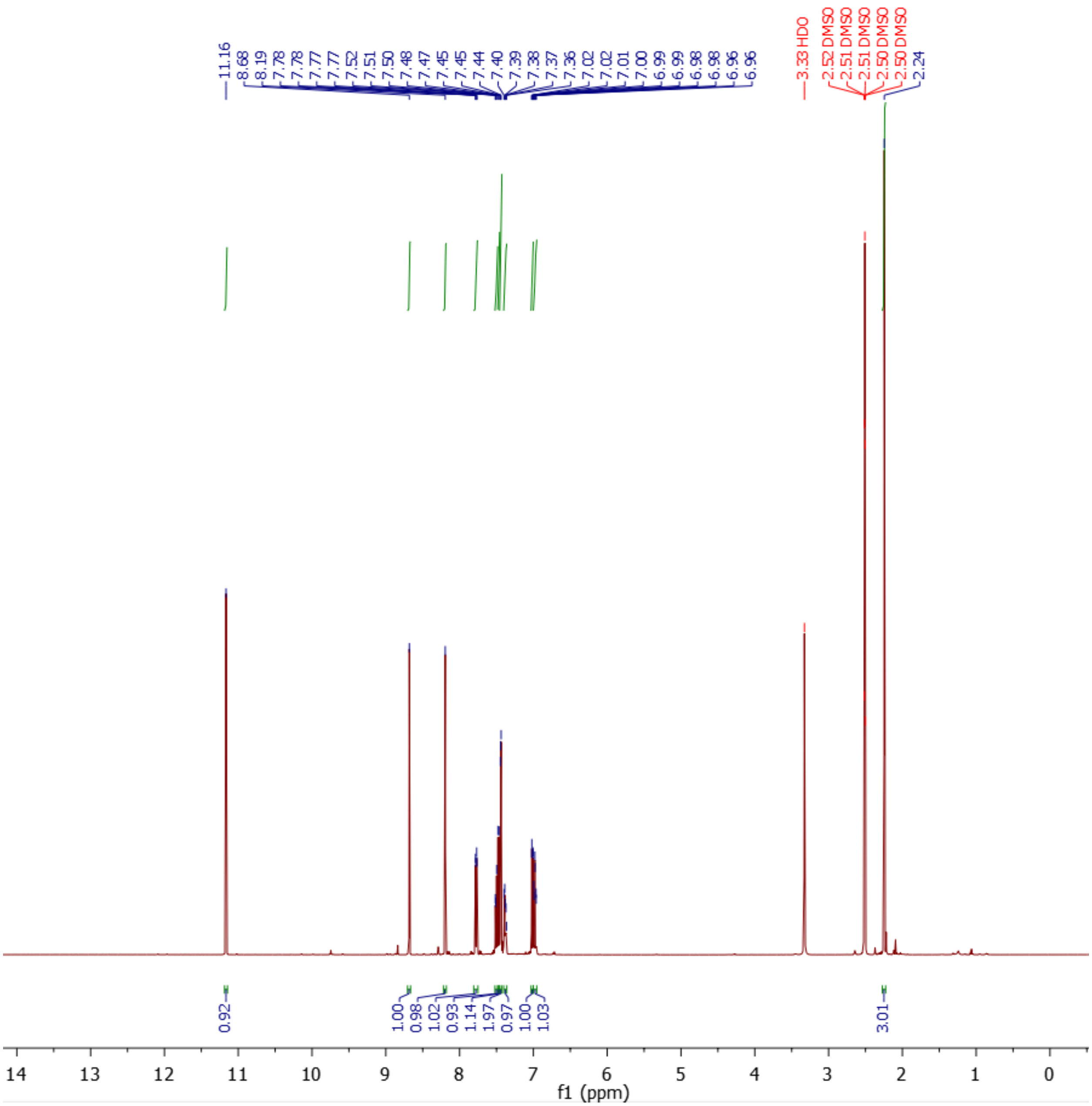

^{1}H NMR of compound **CK-45** (500 MHz, DMSO-d_6).

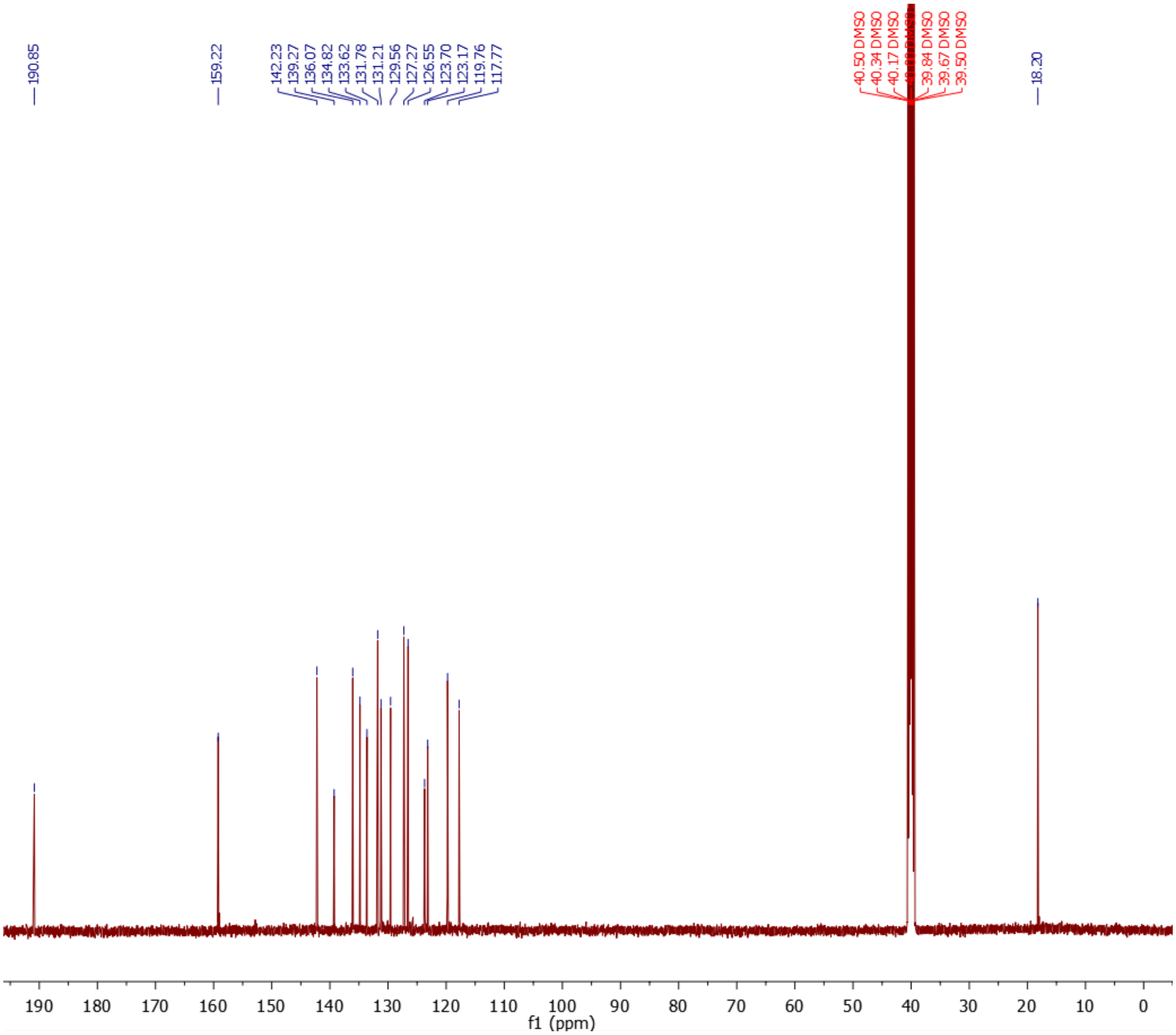

^{13}C NMR of compound **CK-45** (126 MHz, DMSO-d_6).

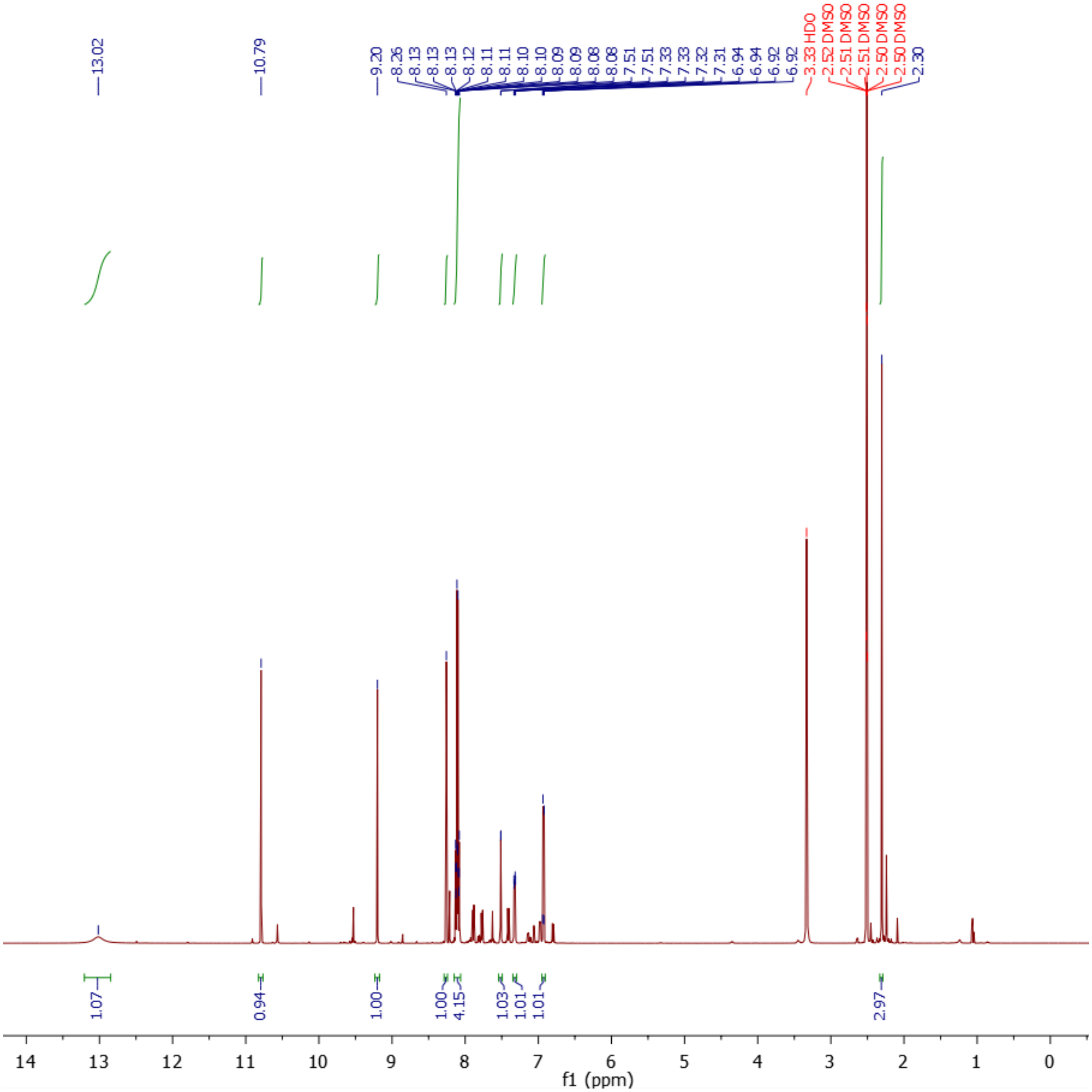

^{1}H NMR of compound **CK-54** (500 MHz, DMSO-d_6).

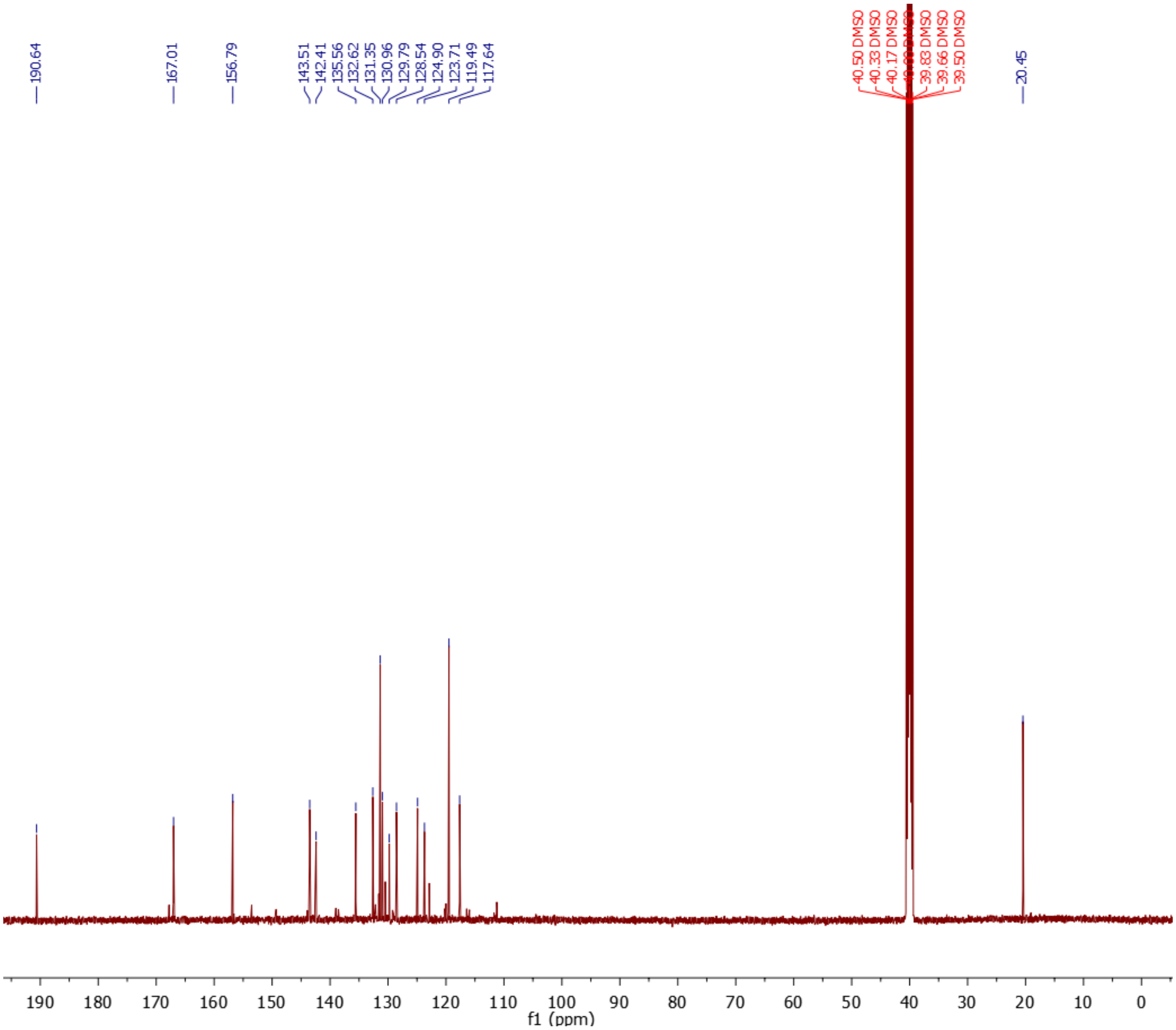

^{13}C NMR of compound **CK-54** (126 MHz, DMSO-*d*6).

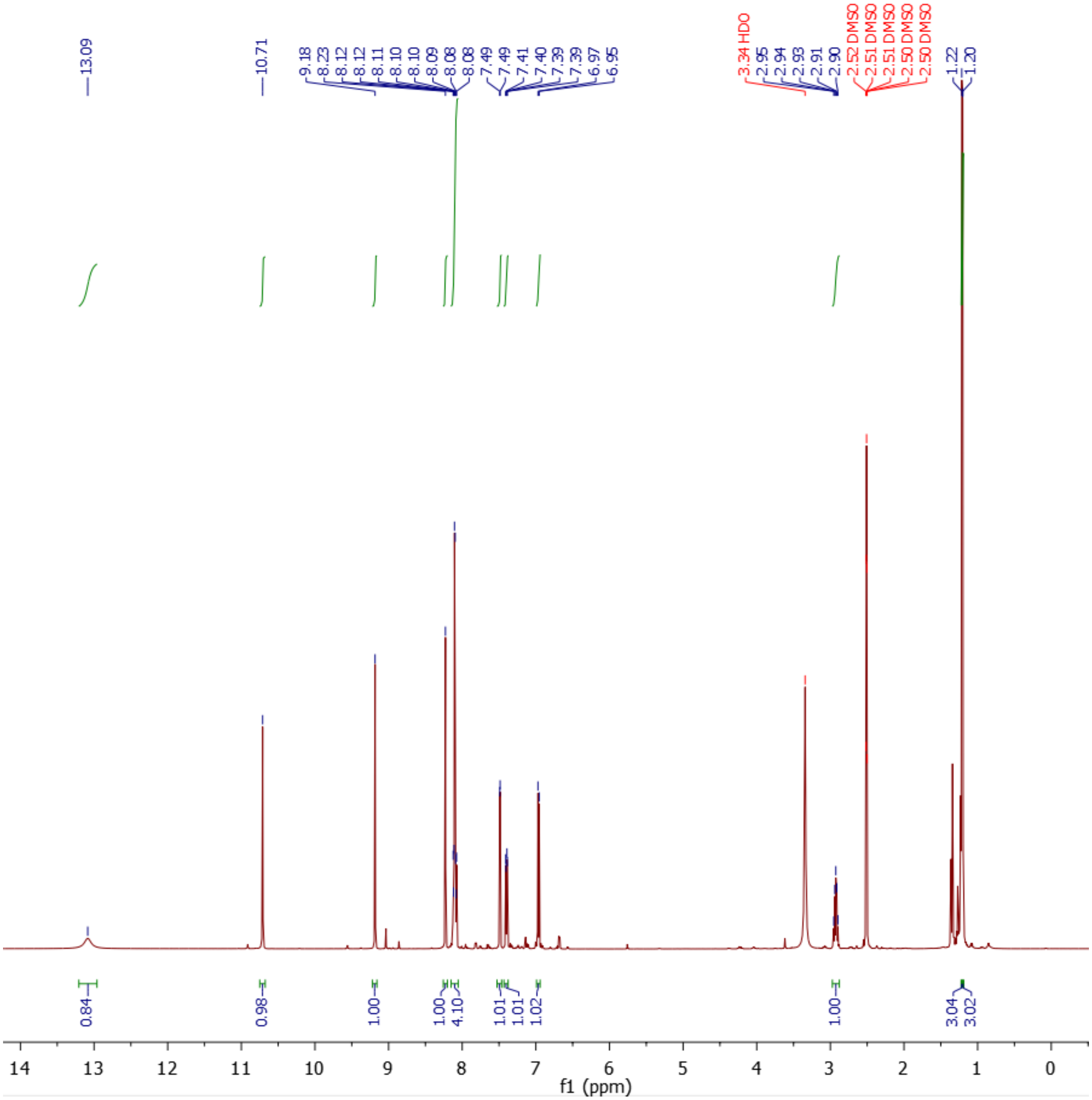

^{1}H NMR of compound **CK-55** (500 MHz, DMSO-*d*$_6$).

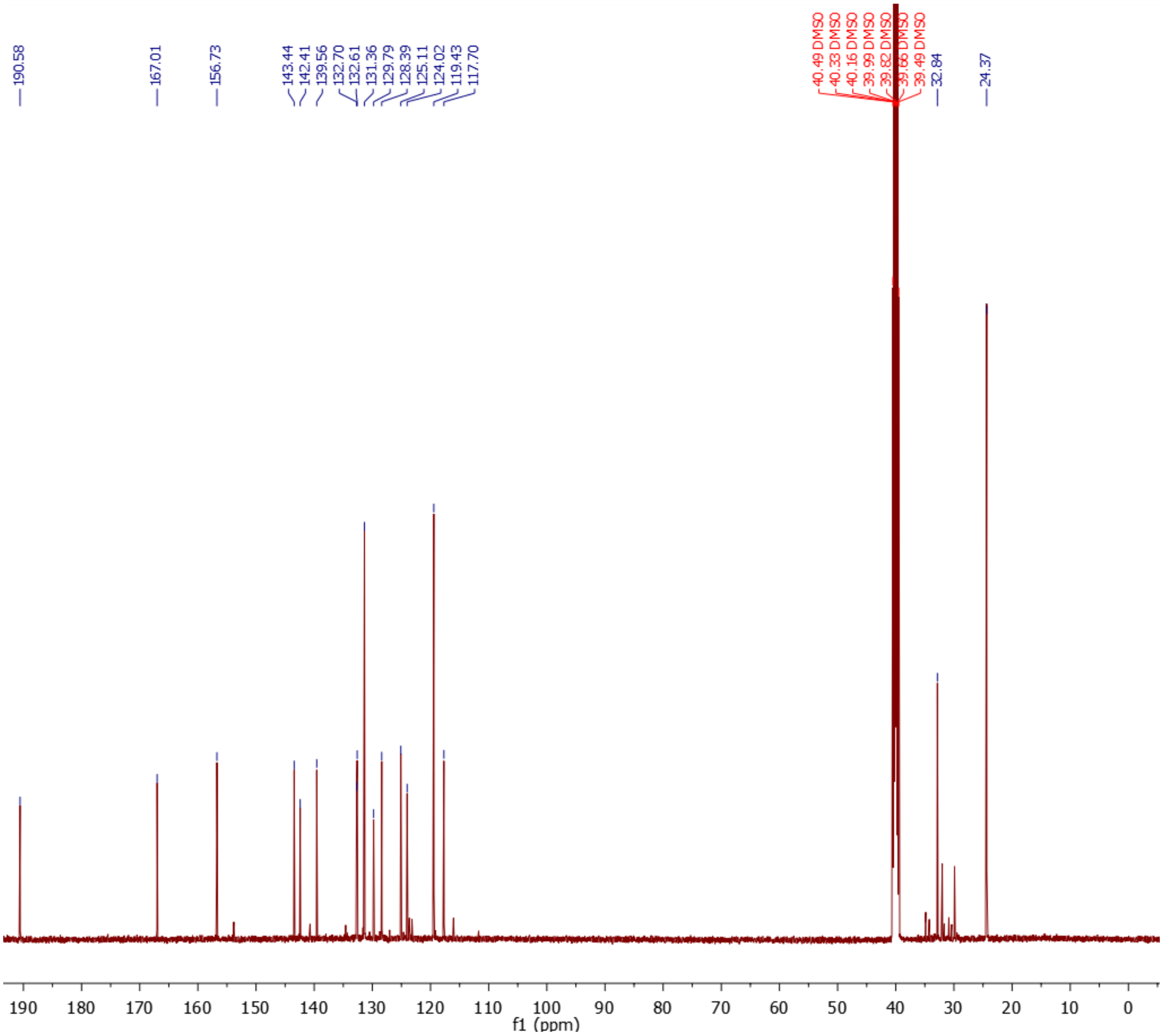

^{13}C NMR of compound **CK-55** (126 MHz, DMSO-d_6).

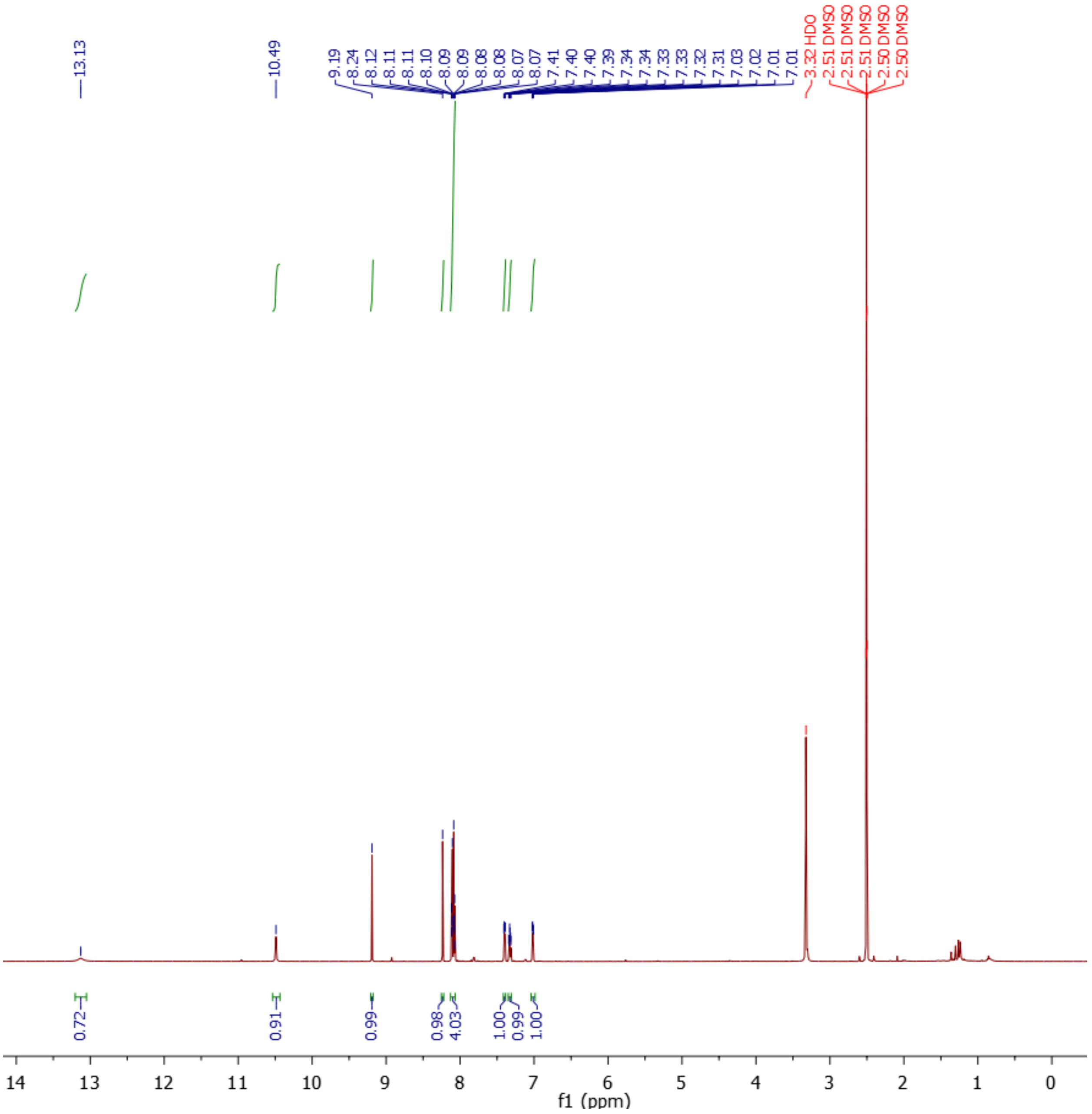

^{1}H NMR of compound **CK-56** (700 MHz, DMSO-d_6).

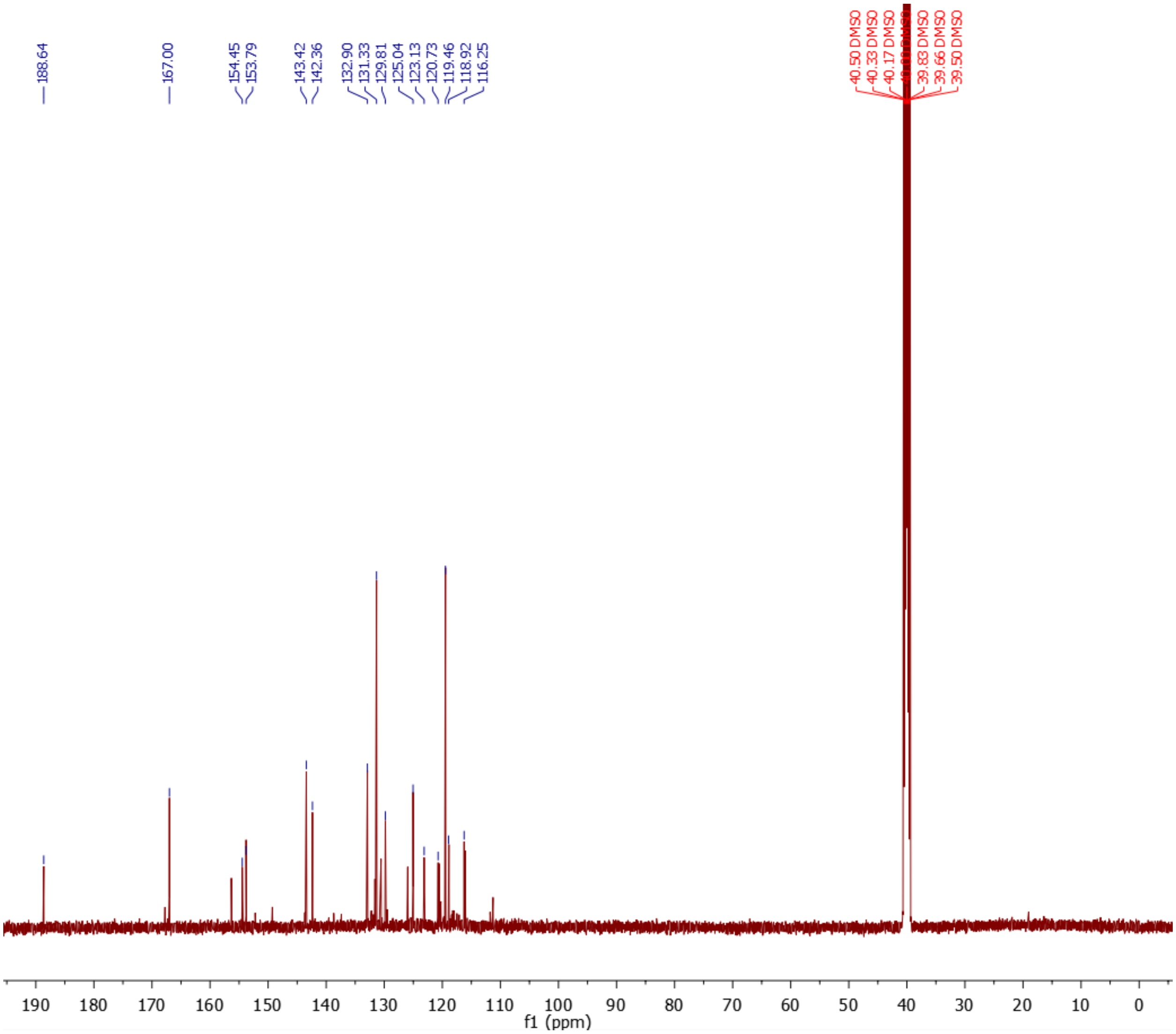

^{13}C NMR of compound **CK-56** (176 MHz, DMSO-d_6).

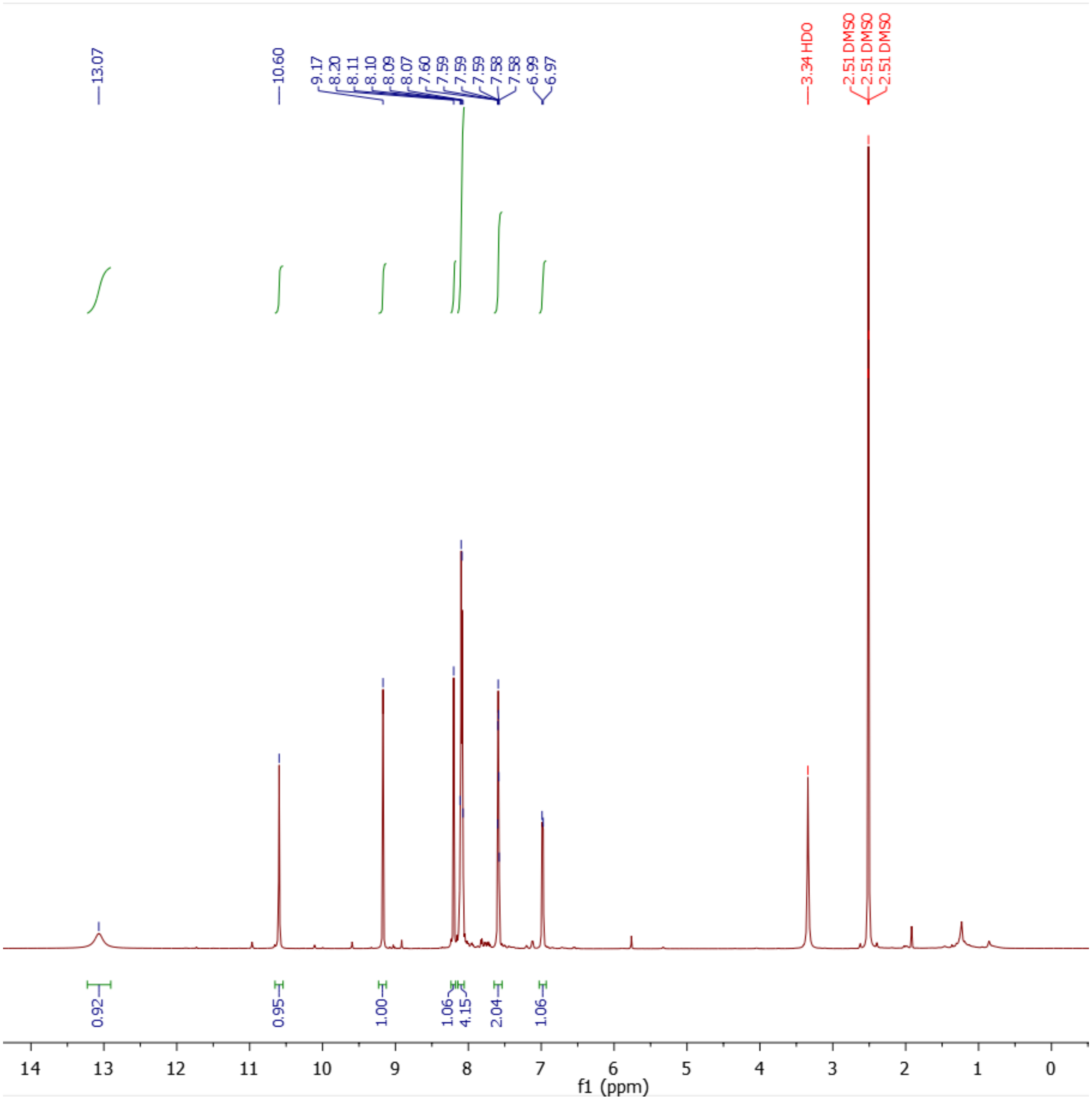

^{1}H NMR of compound **CK-60** (600 MHz, DMSO-*d*$_6$).

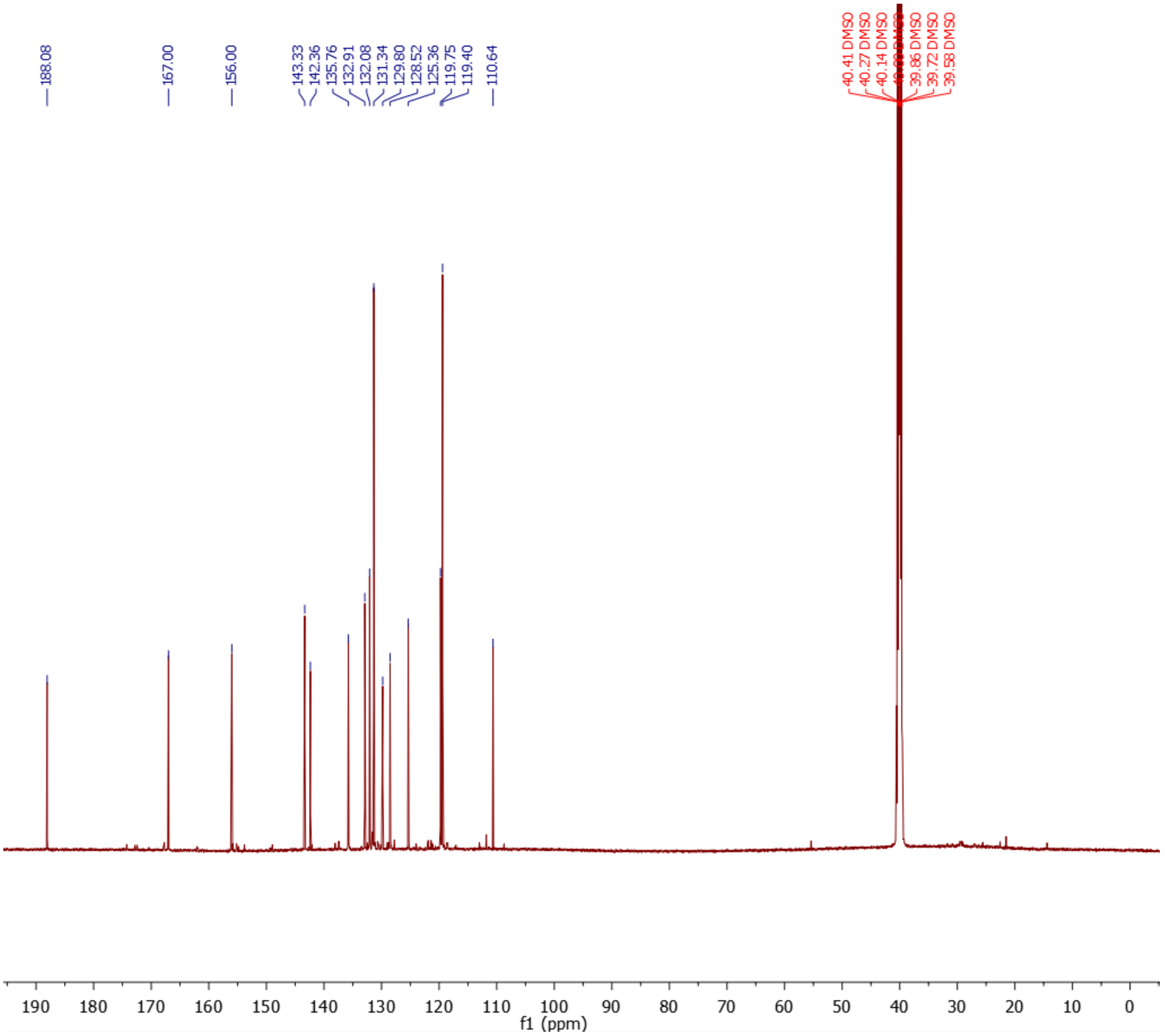

^{13}C NMR of compound **CK-60** (151 MHz, DMSO-*d*$_6$).

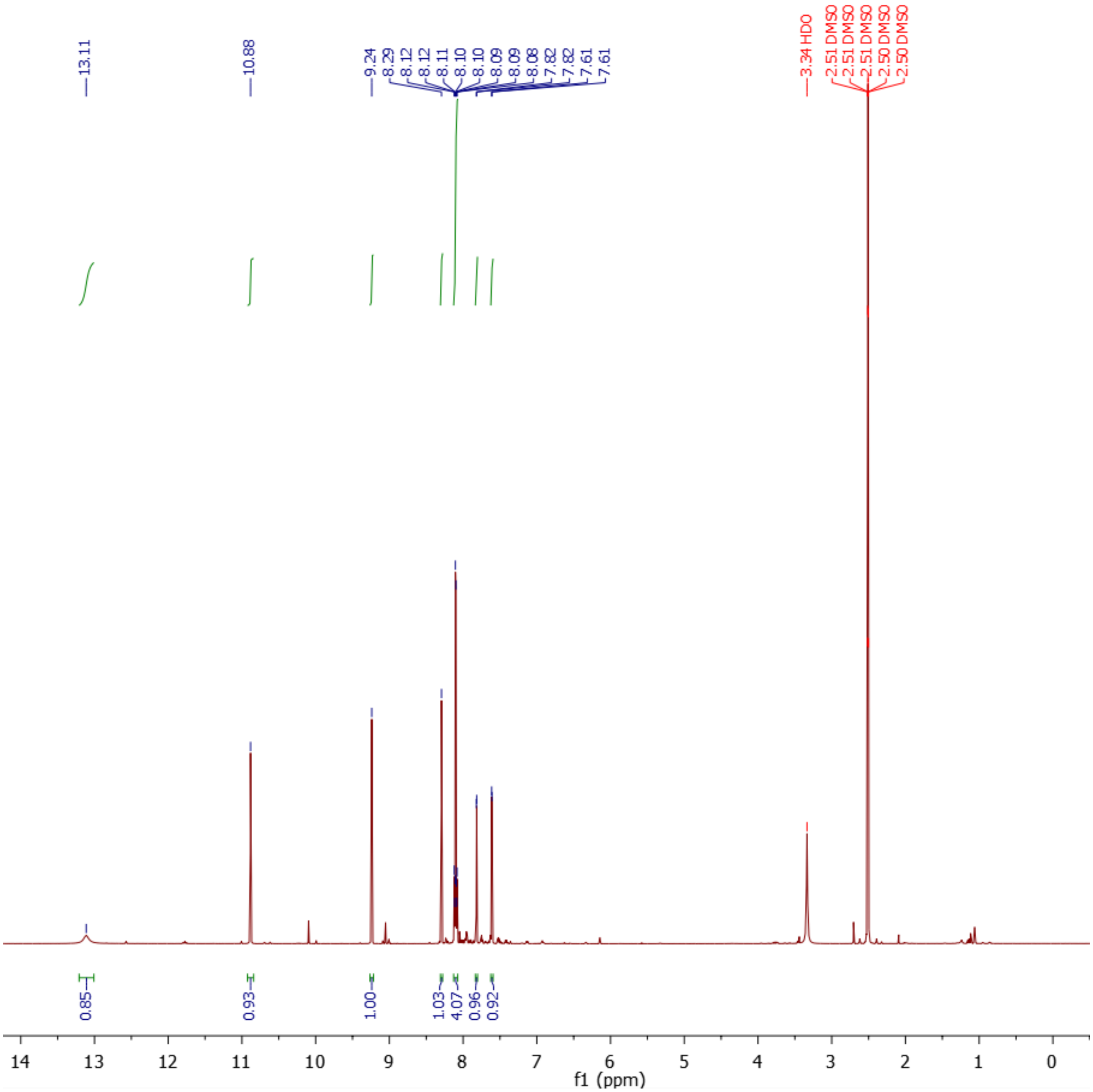

^{1}H NMR of compound **CK-61** (600 MHz, DMSO-*d*$_6$).

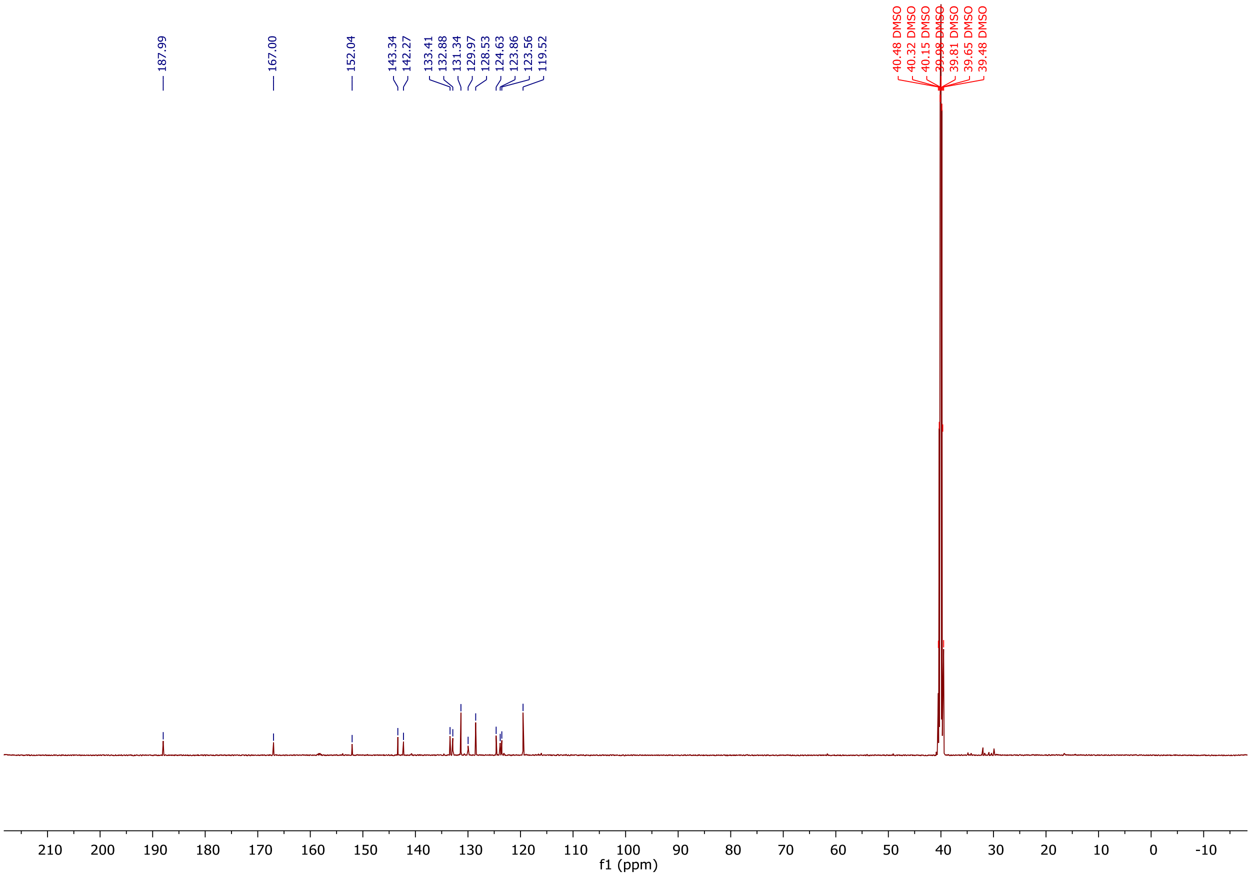

^{13}C NMR of compound **CK-61** (151 MHz, DMSO-d_6).

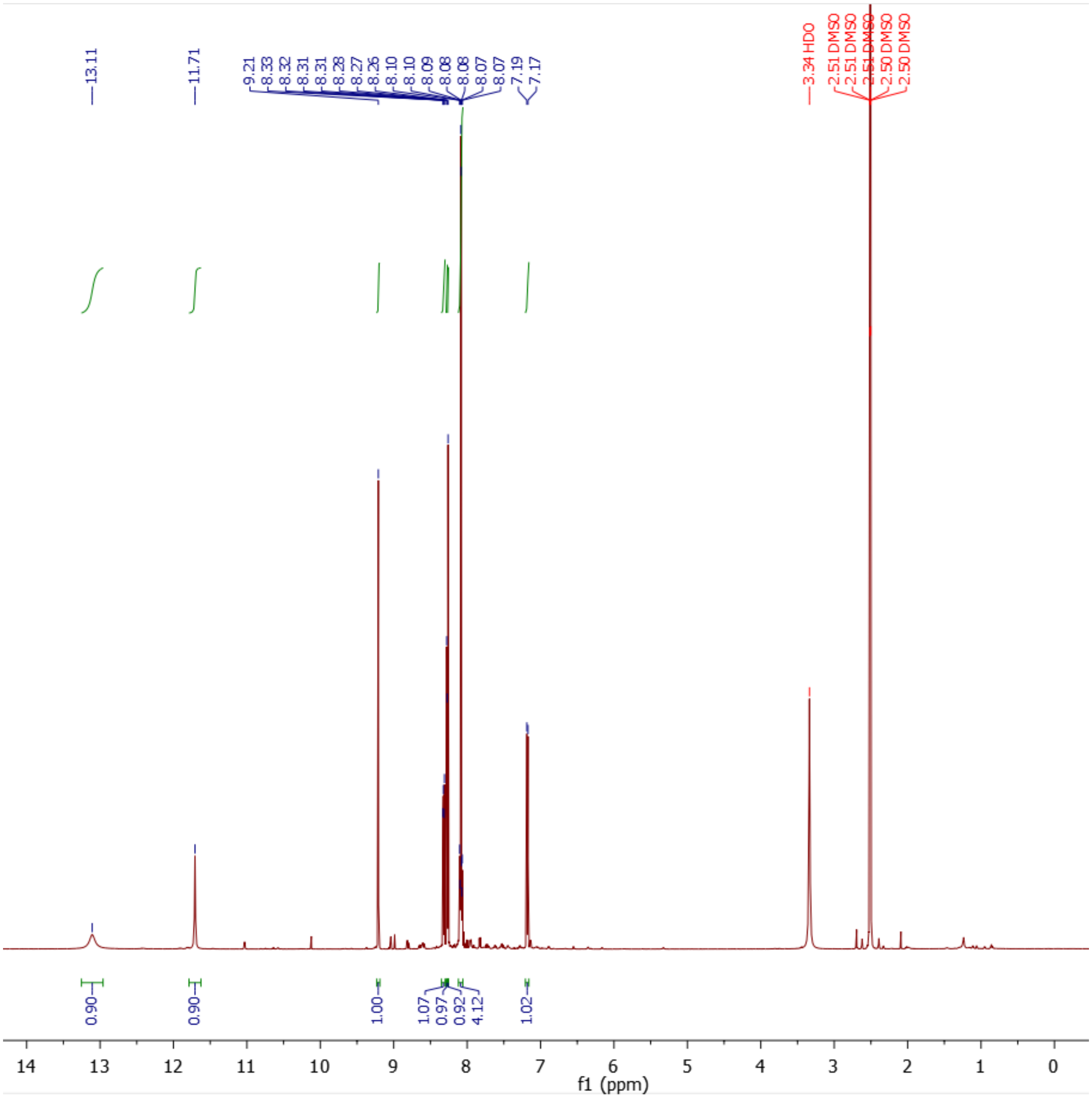

^{1}H NMR of compound **CK-62** (600 MHz, DMSO-d_6).

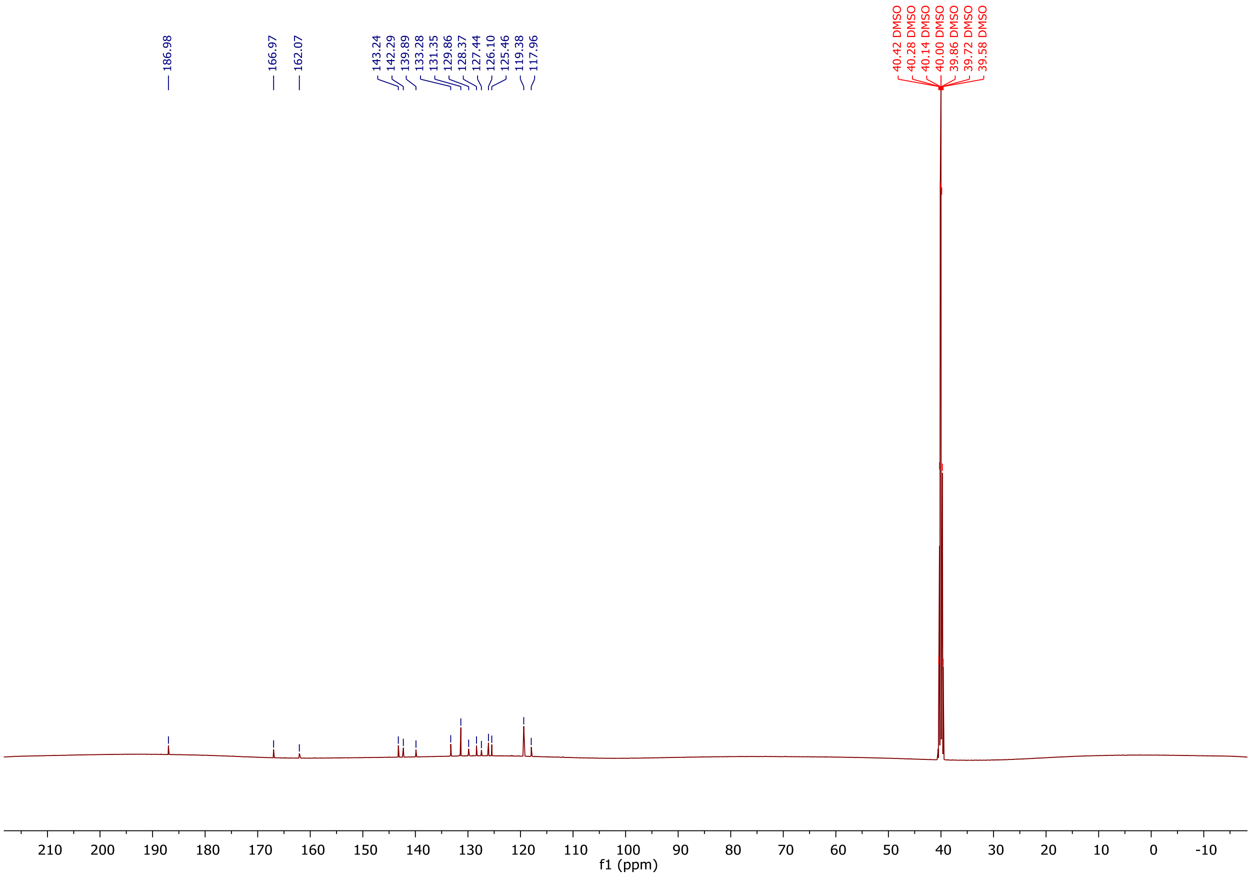

^{13}C NMR of compound **CK-62** (151 MHz, DMSO-d_6).

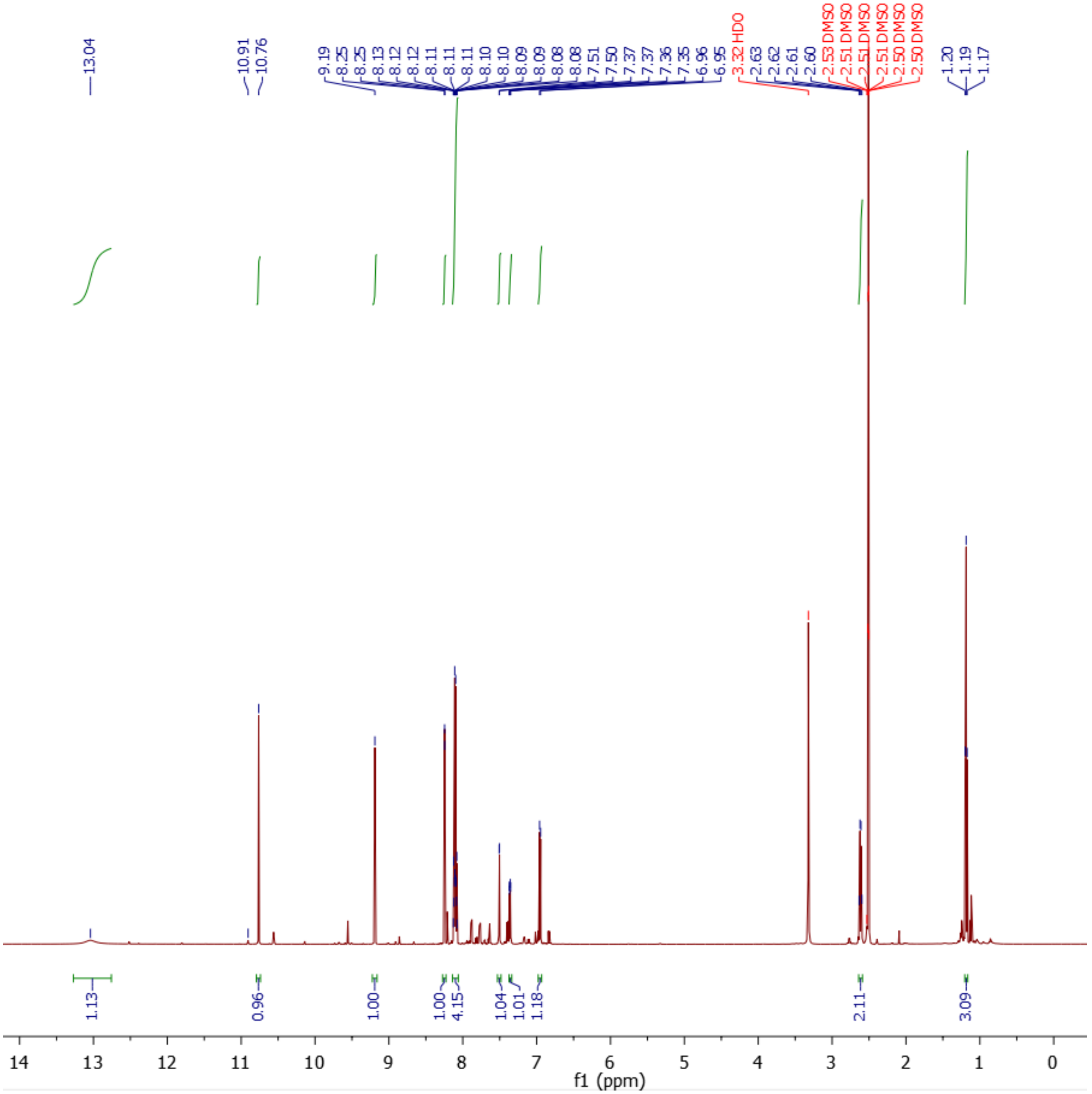

^{1}H NMR of compound **CK-65** (600 MHz, DMSO-*d*$_6$).

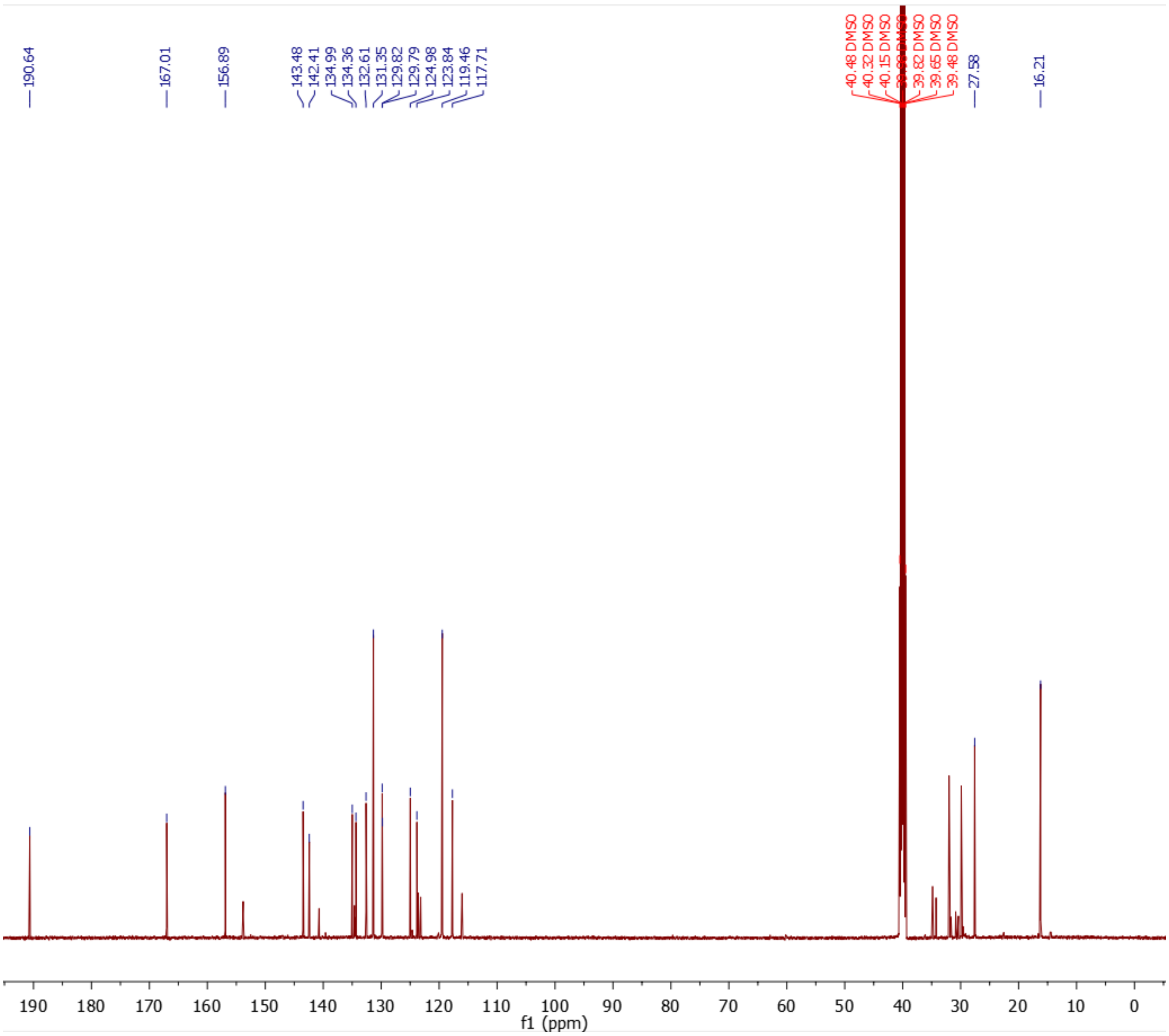

^{13}C NMR of compound **CK-65** (151 MHz, DMSO-d_6).

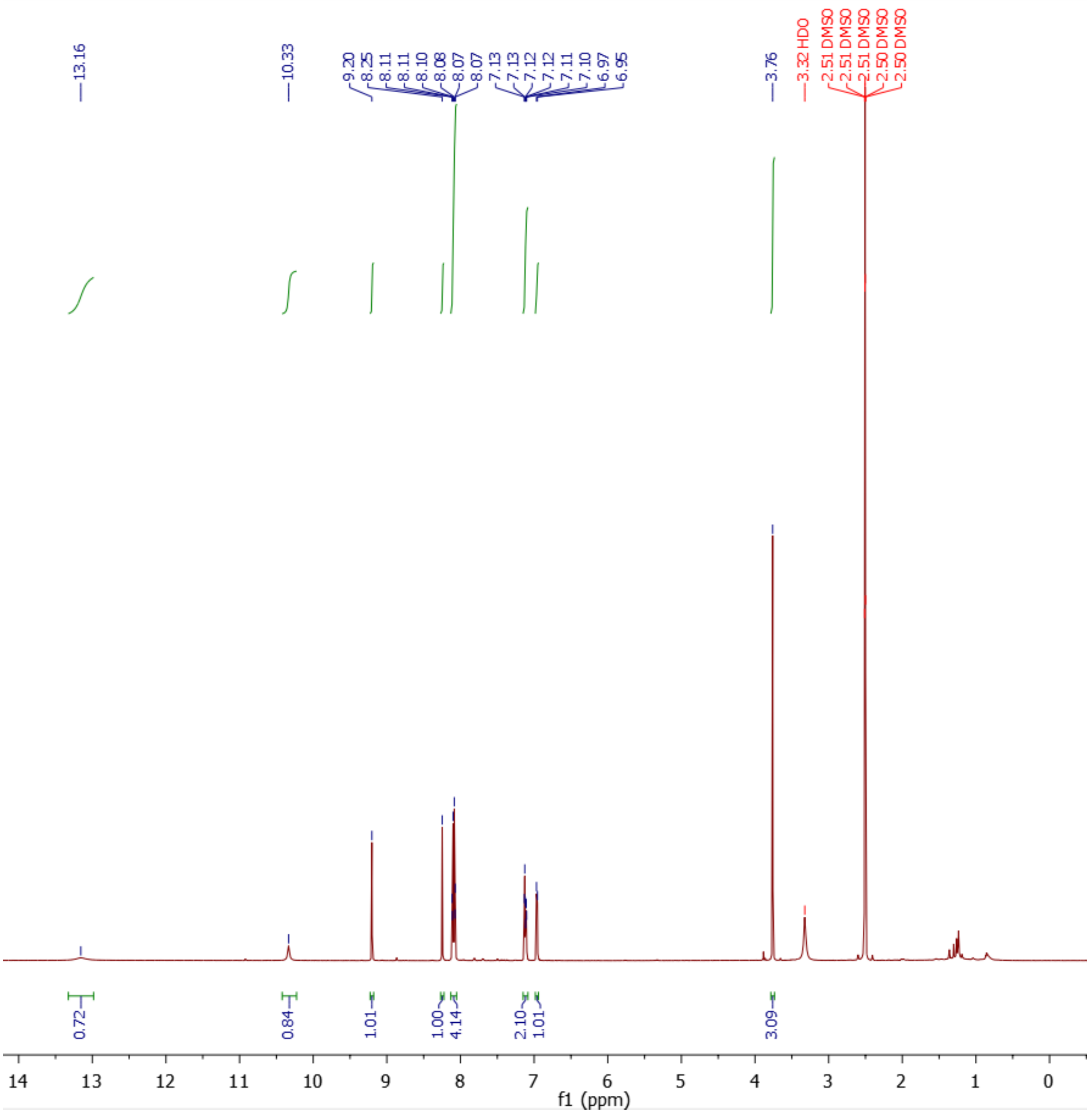

^{1}H NMR of compound **CK-66** (700 MHz, DMSO-*d*$_6$).

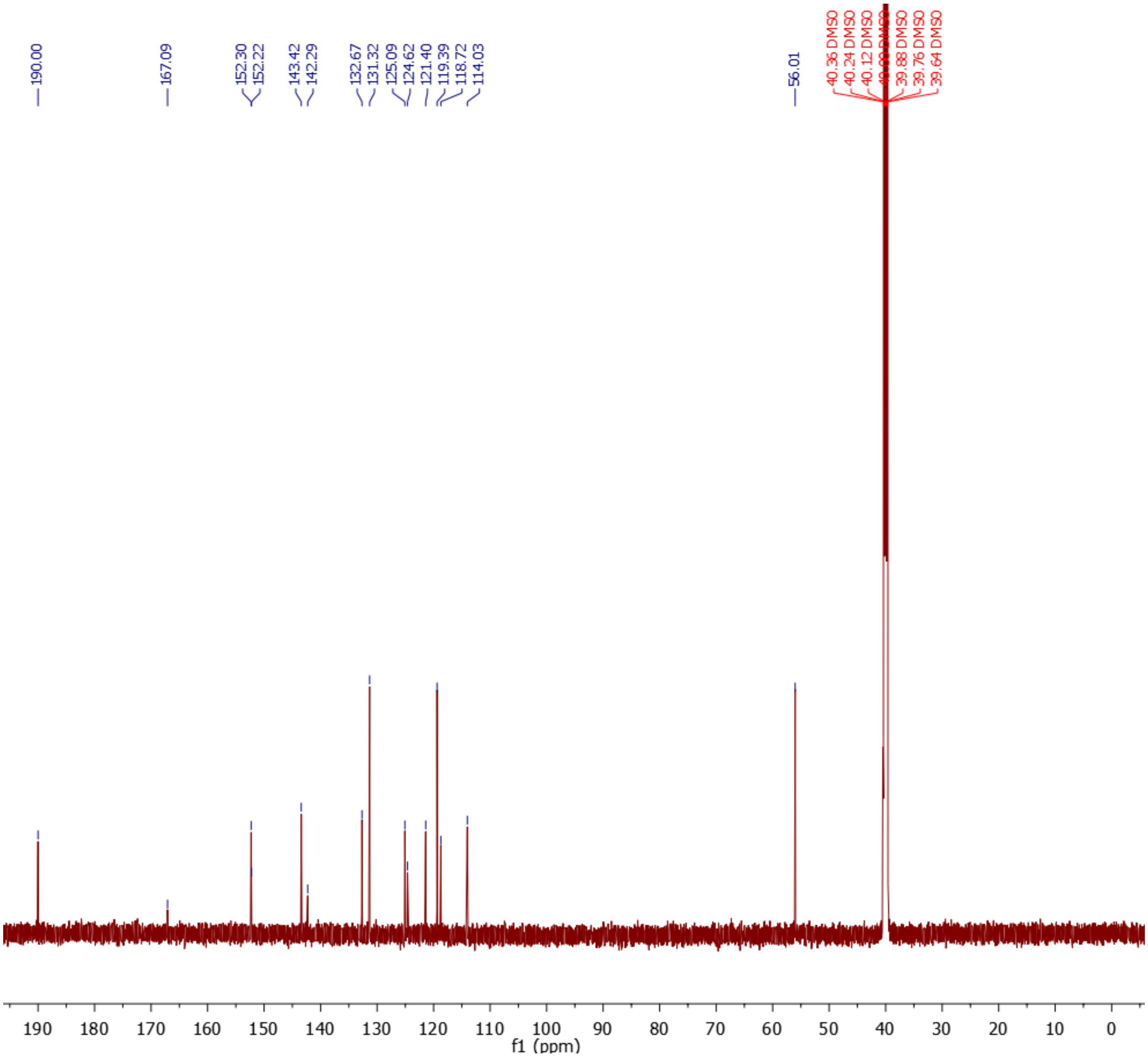

^{13}C NMR of compound **CK-66** (176 MHz, DMSO-*d*$_6$).

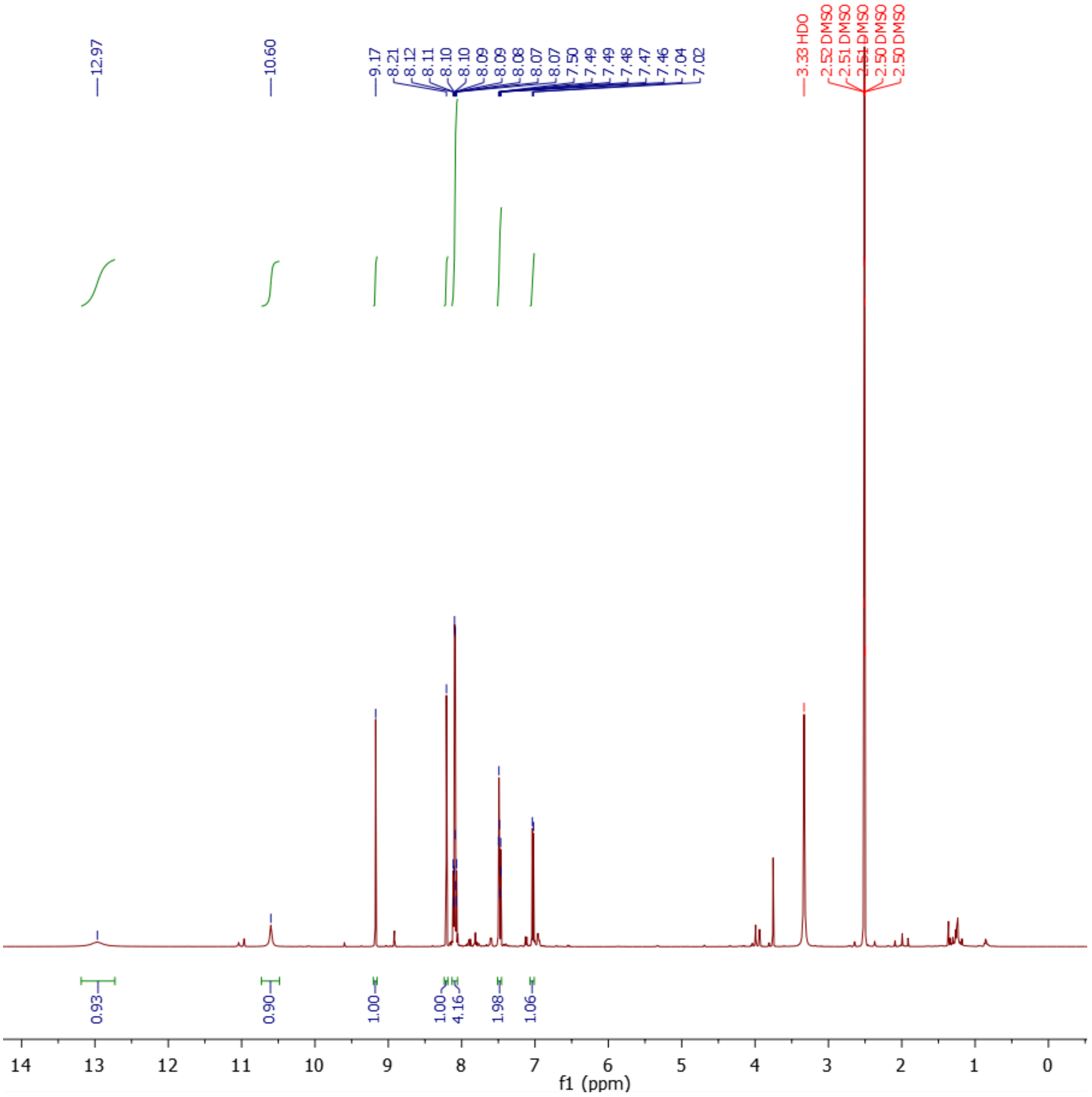

^{1}H NMR of compound **CK-67** (600 MHz, DMSO-d_6).

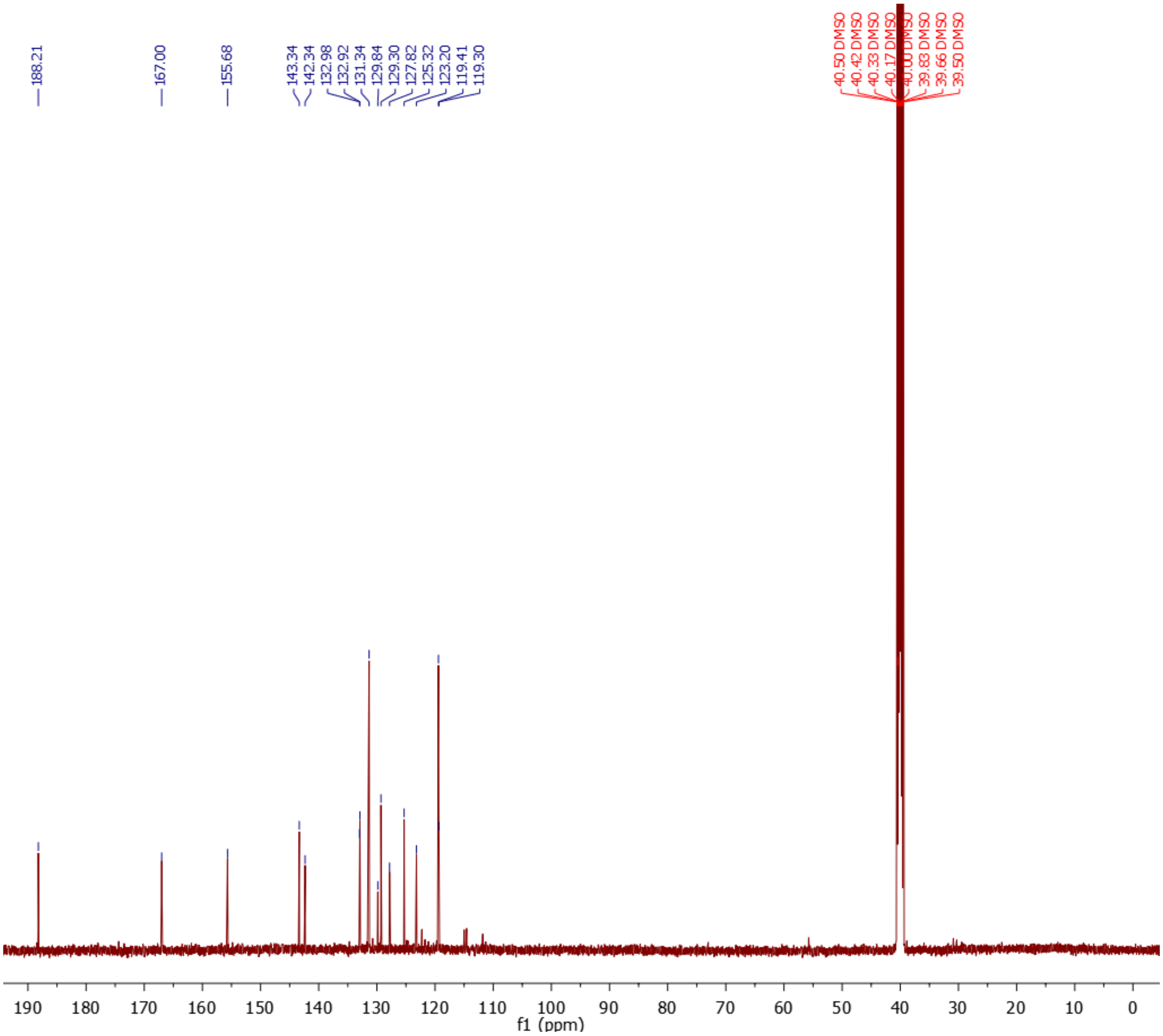

^{13}C NMR of compound **CK-67** (151 MHz, DMSO-*d*$_6$).

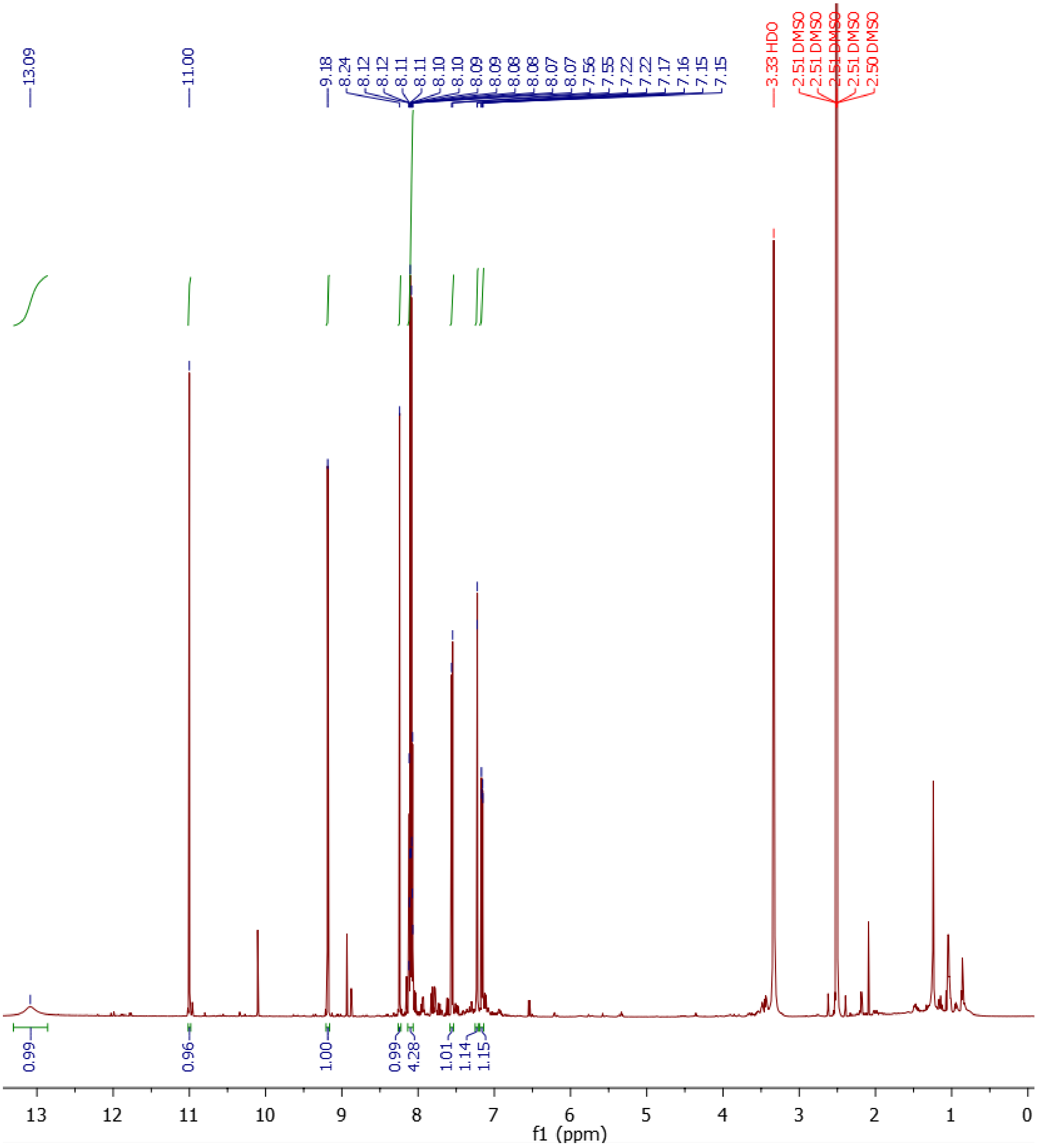

^{1}H NMR of compound **CK-70** (600 MHz, DMSO-d_6).

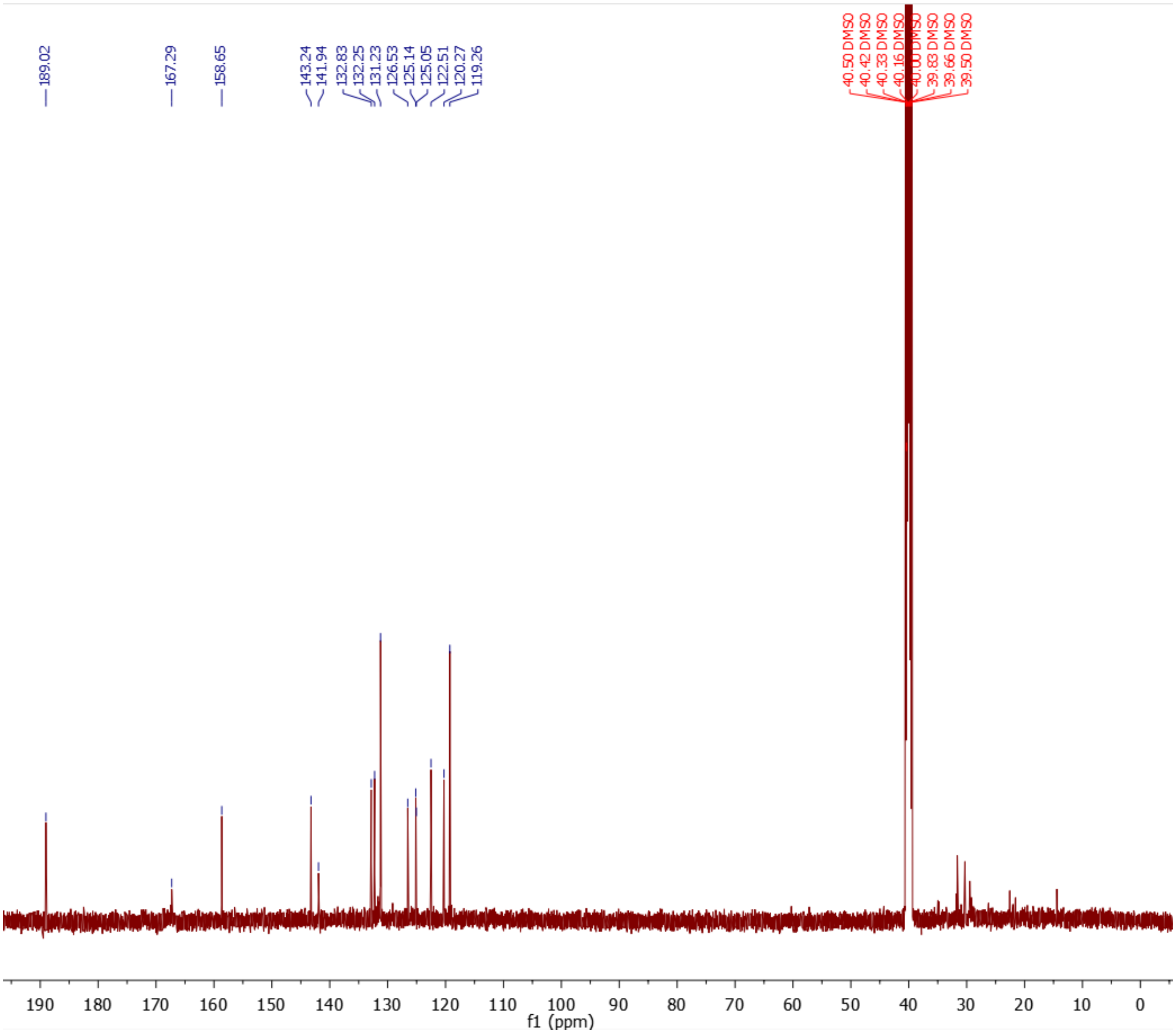

^{13}**C NMR** of compound **CK-70** (151 MHz, DMSO-d_6).

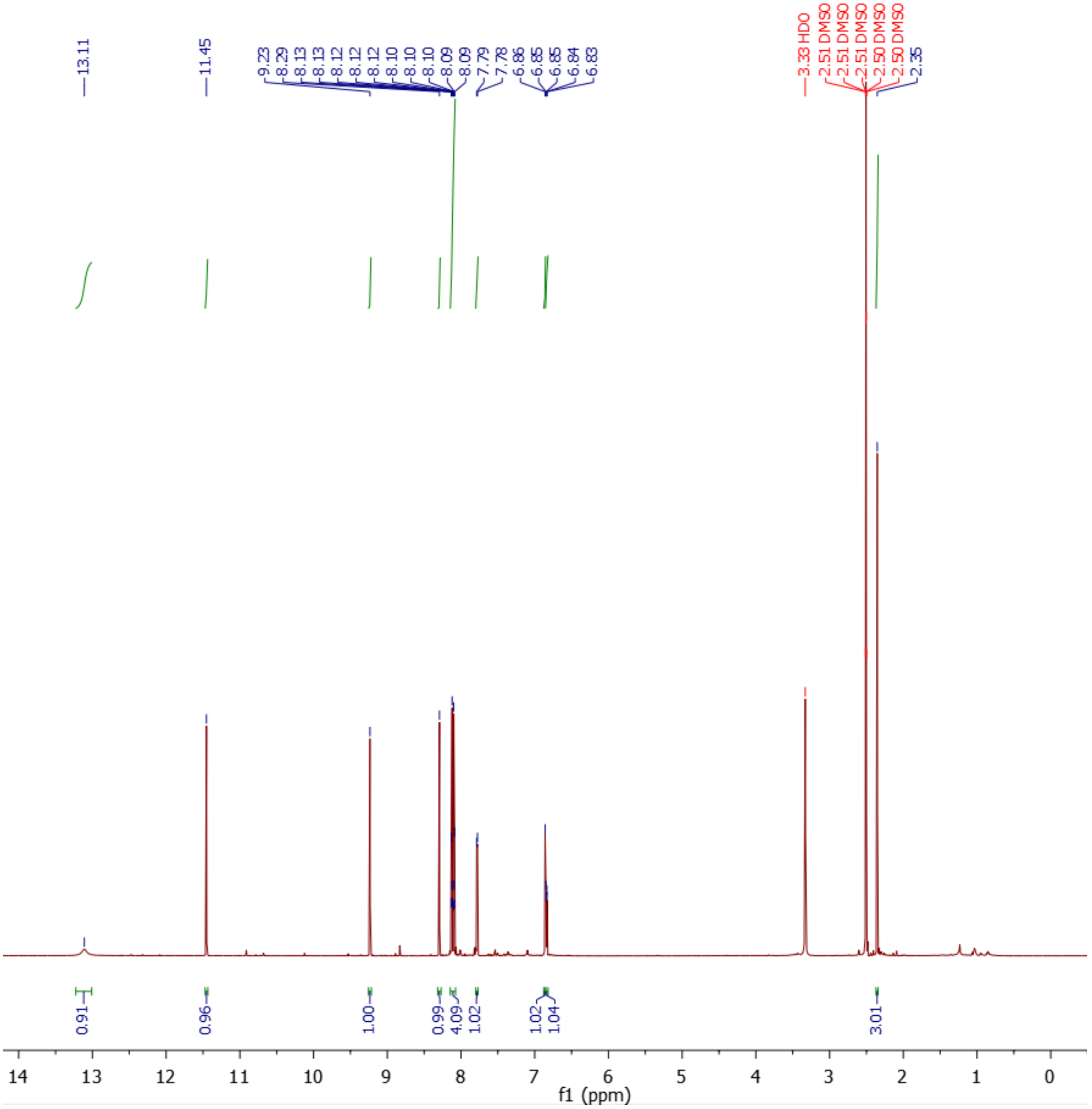

^{1}H NMR of compound **CK-73** (700 MHz, DMSO-*d*$_6$).

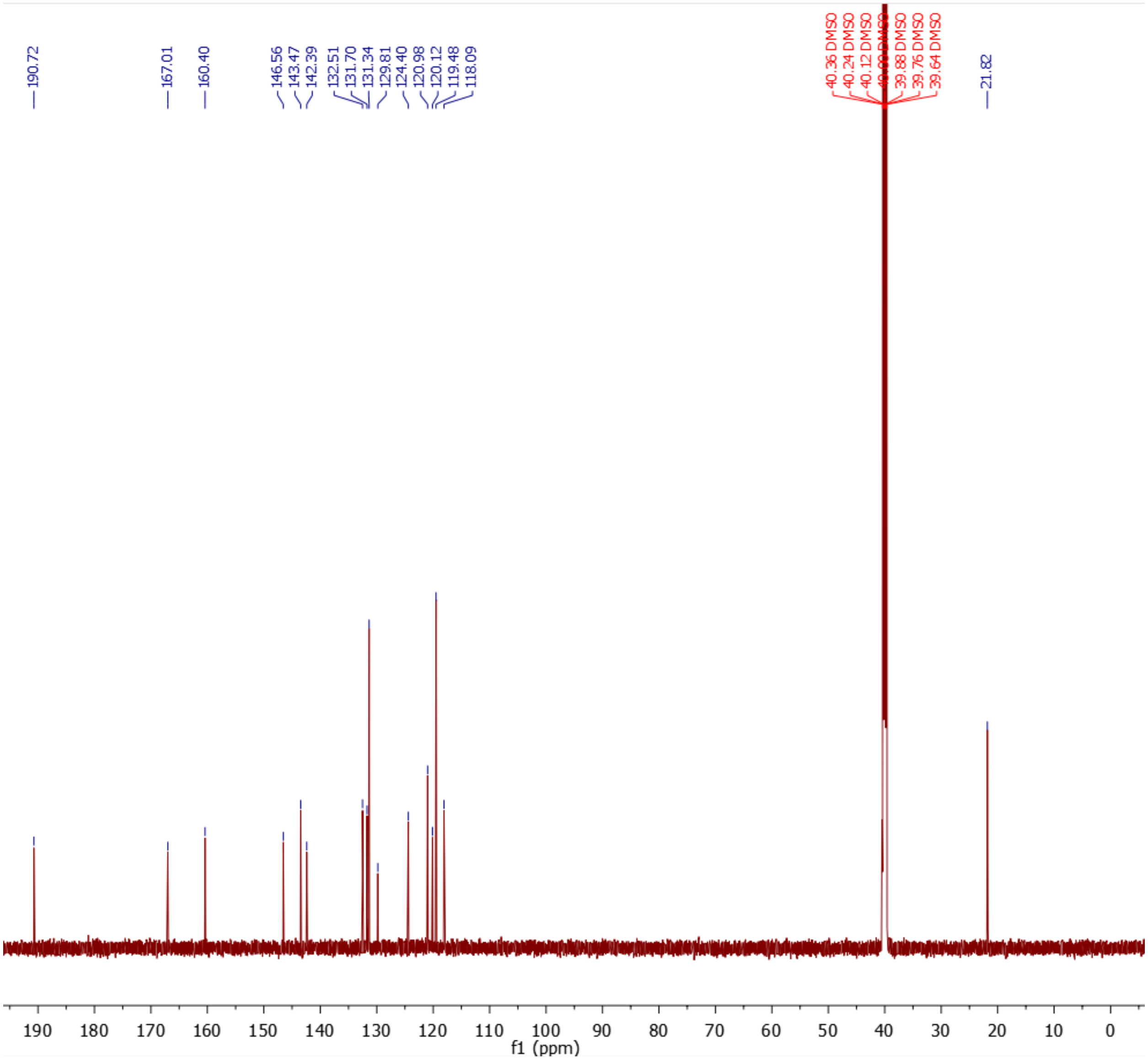

^{13}C NMR of compound **CK-73** (176 MHz, DMSO-d_6).

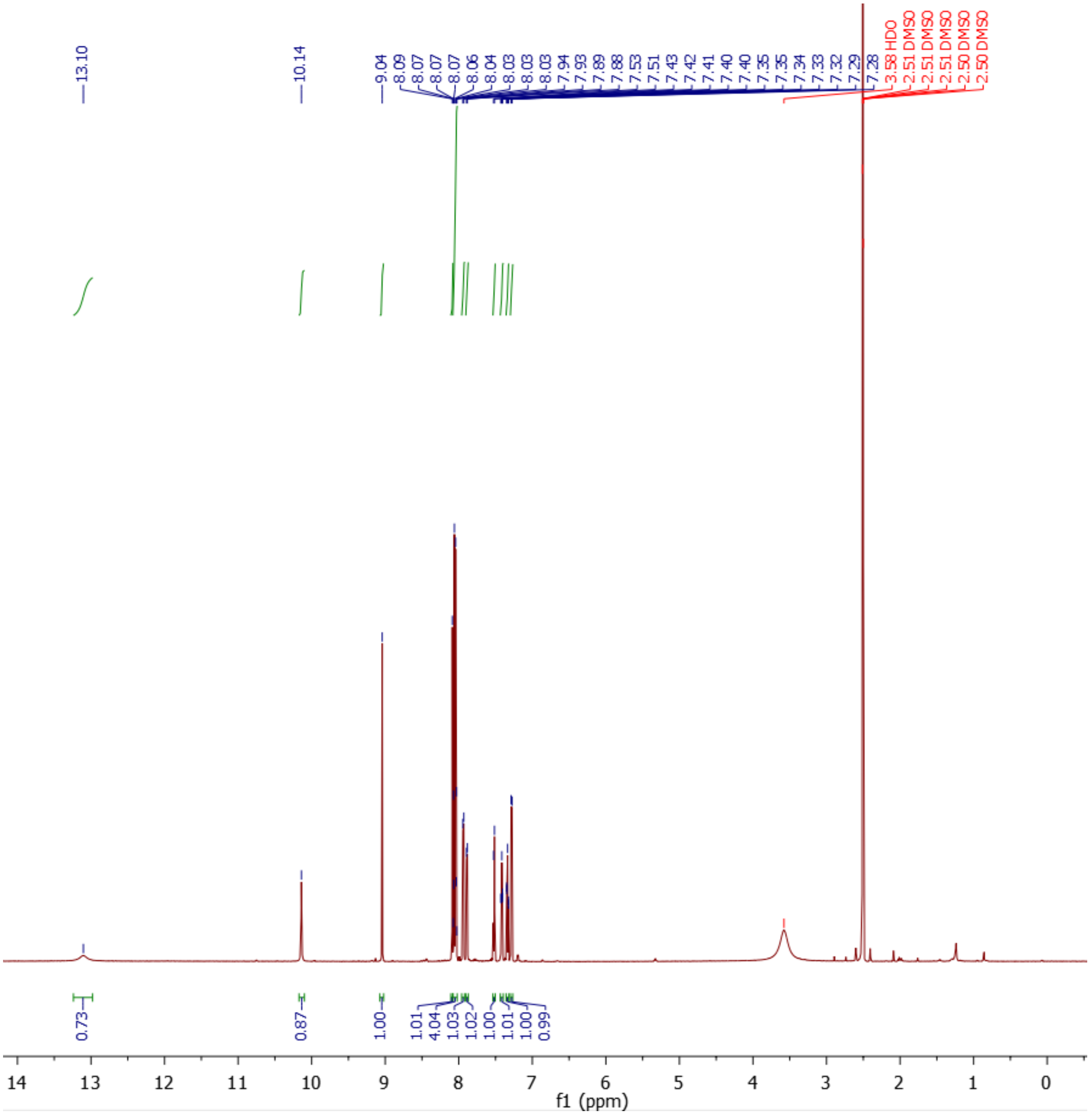

[1]**H NMR** of compound **CK-75** (700 MHz, DMSO-d_6).

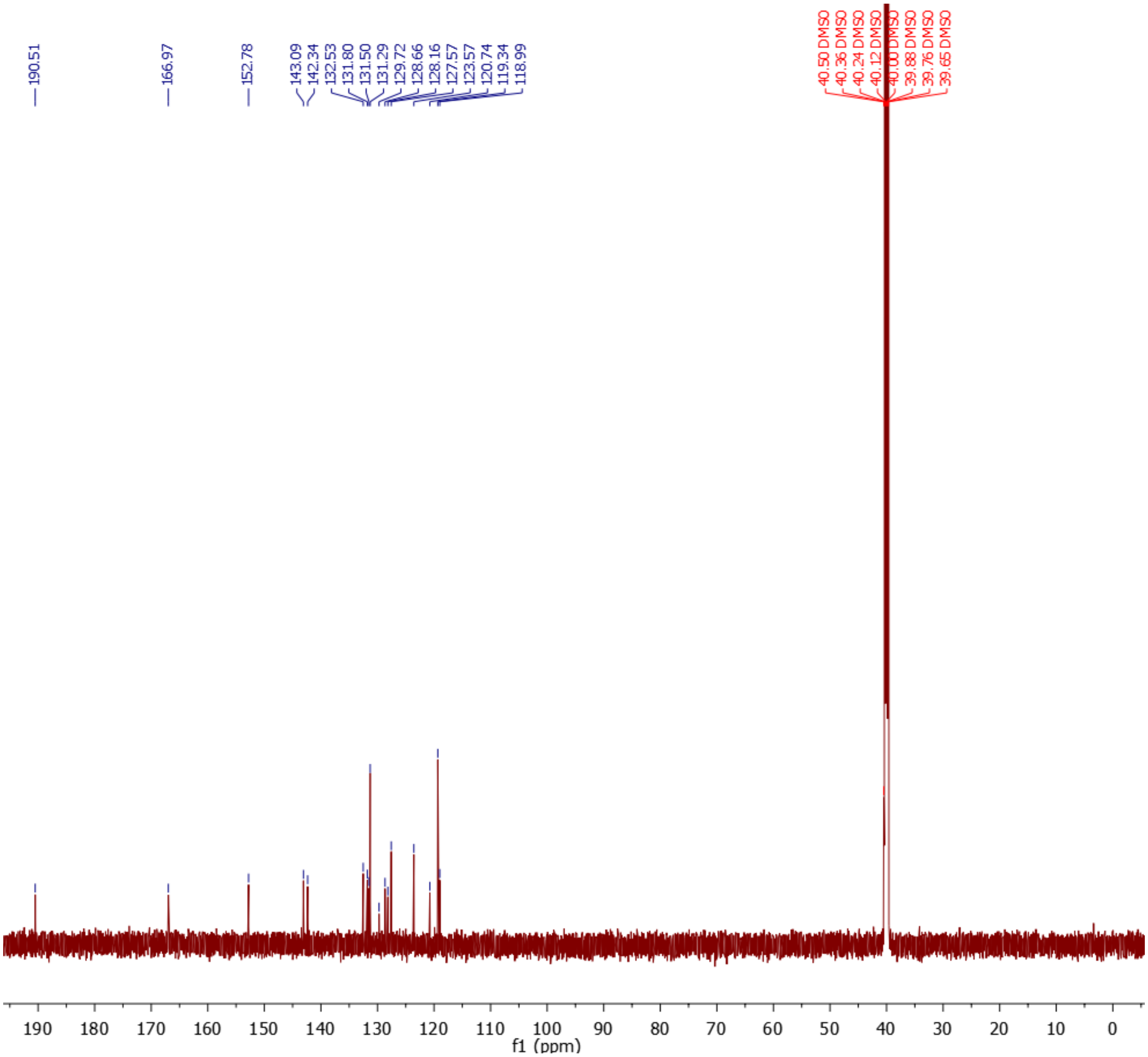

^{13}C NMR of compound **CK-75** (176 MHz, DMSO-d_6).

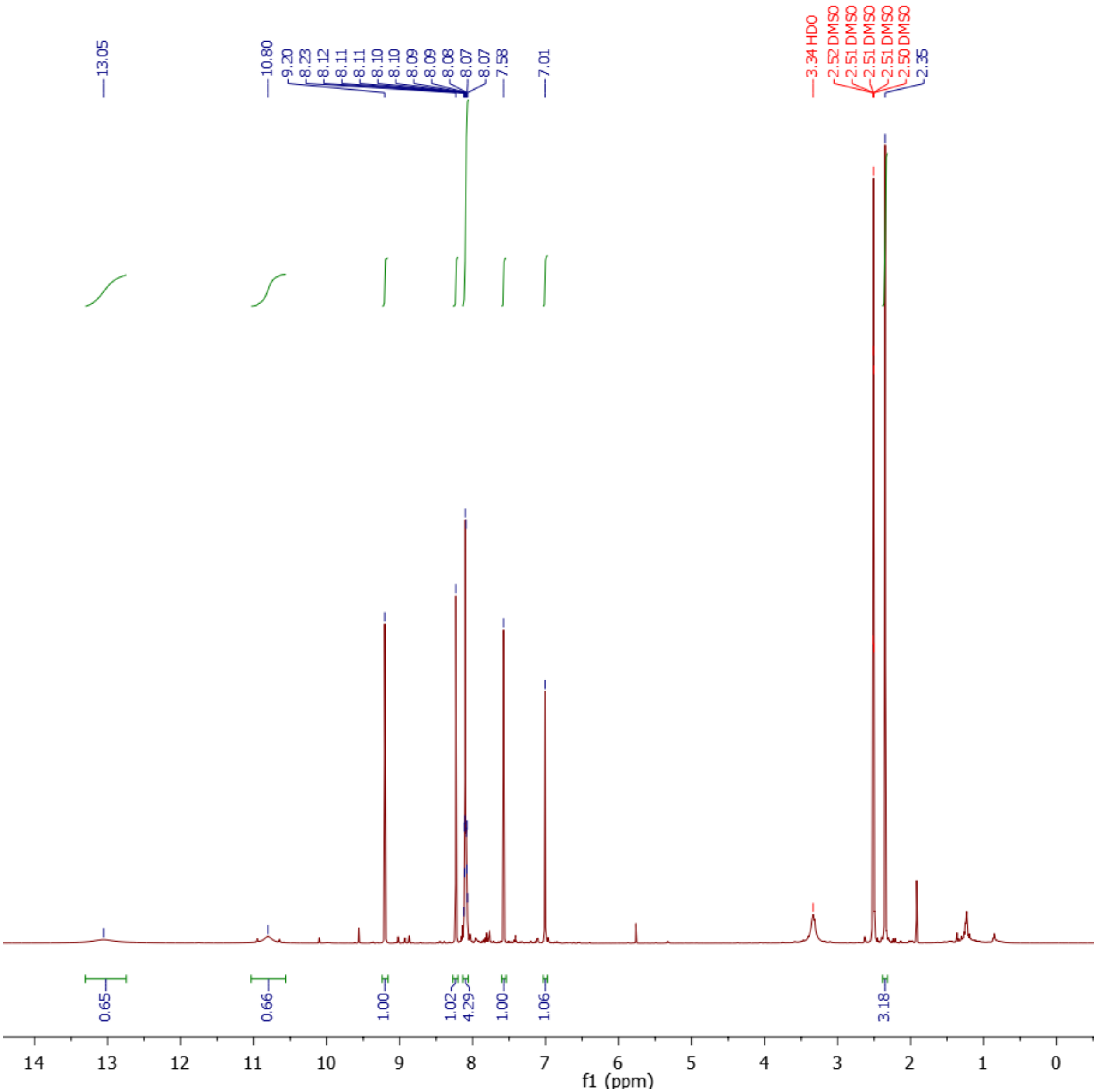

^{1}H NMR of compound **CK-79** (600 MHz, DMSO-d_6).

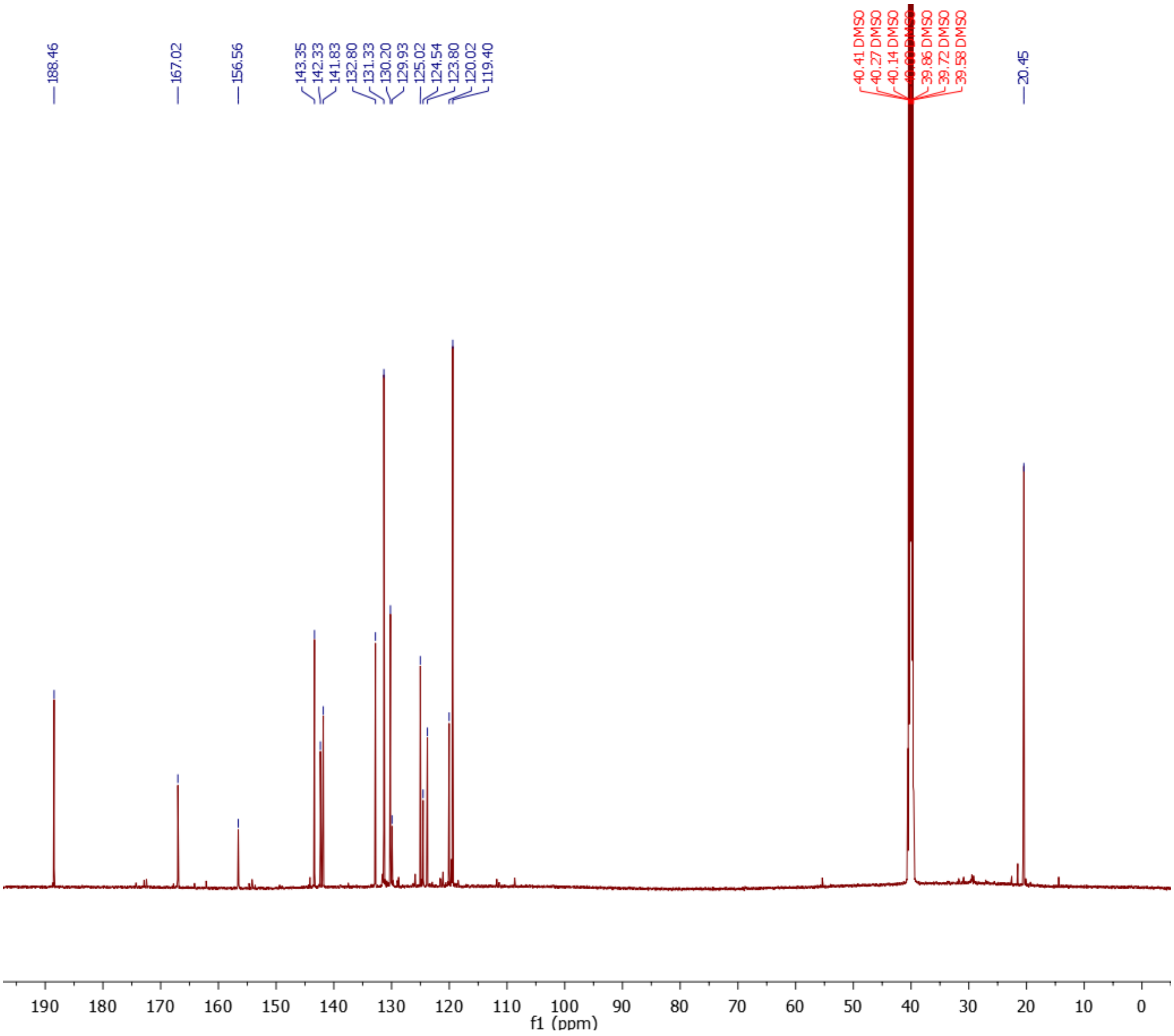

^{13}C NMR of compound **CK-79** (151 MHz, DMSO-d_6).

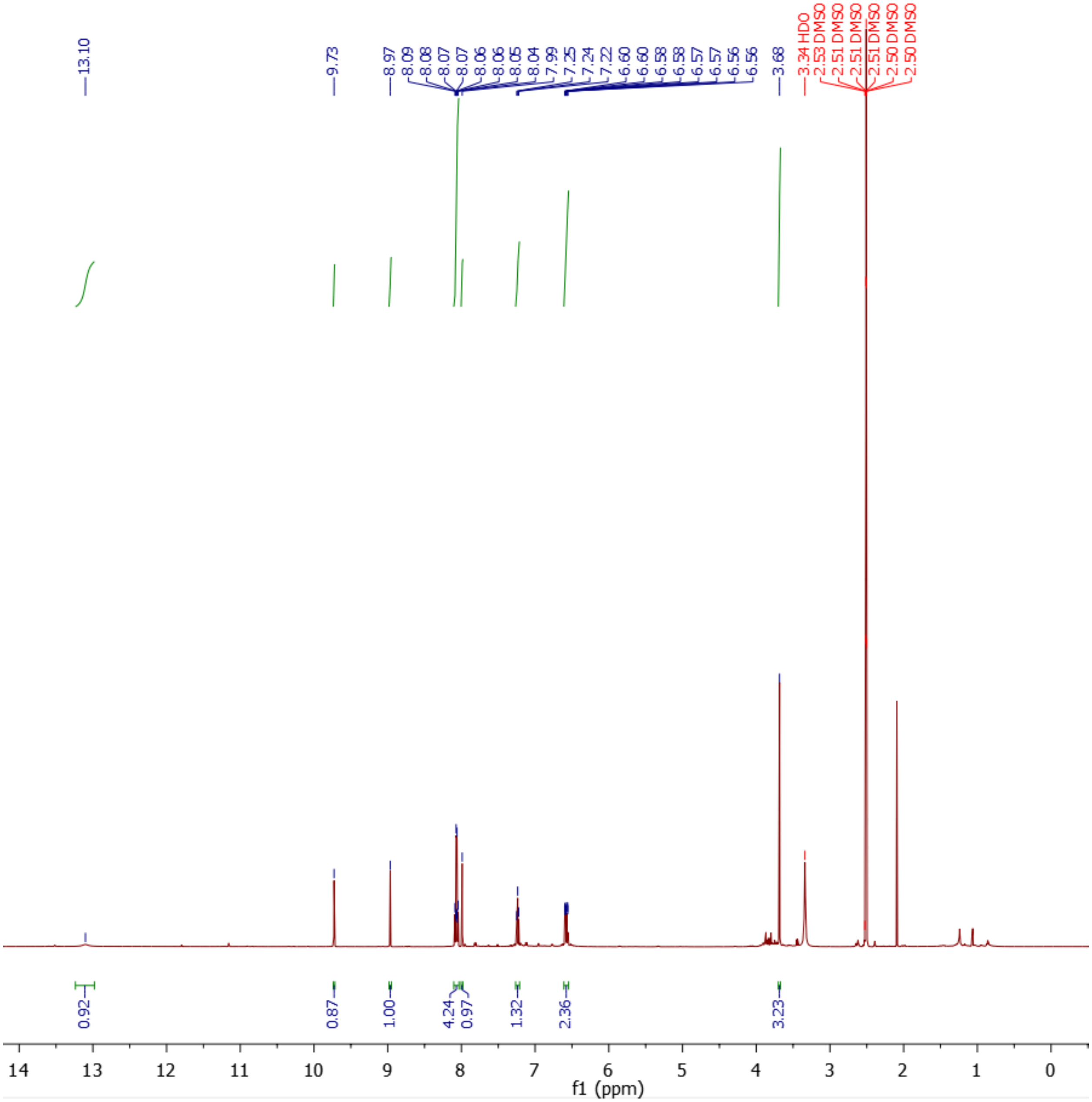

^{1}H NMR of compound **CK-85** (600 MHz, DMSO-d_6).

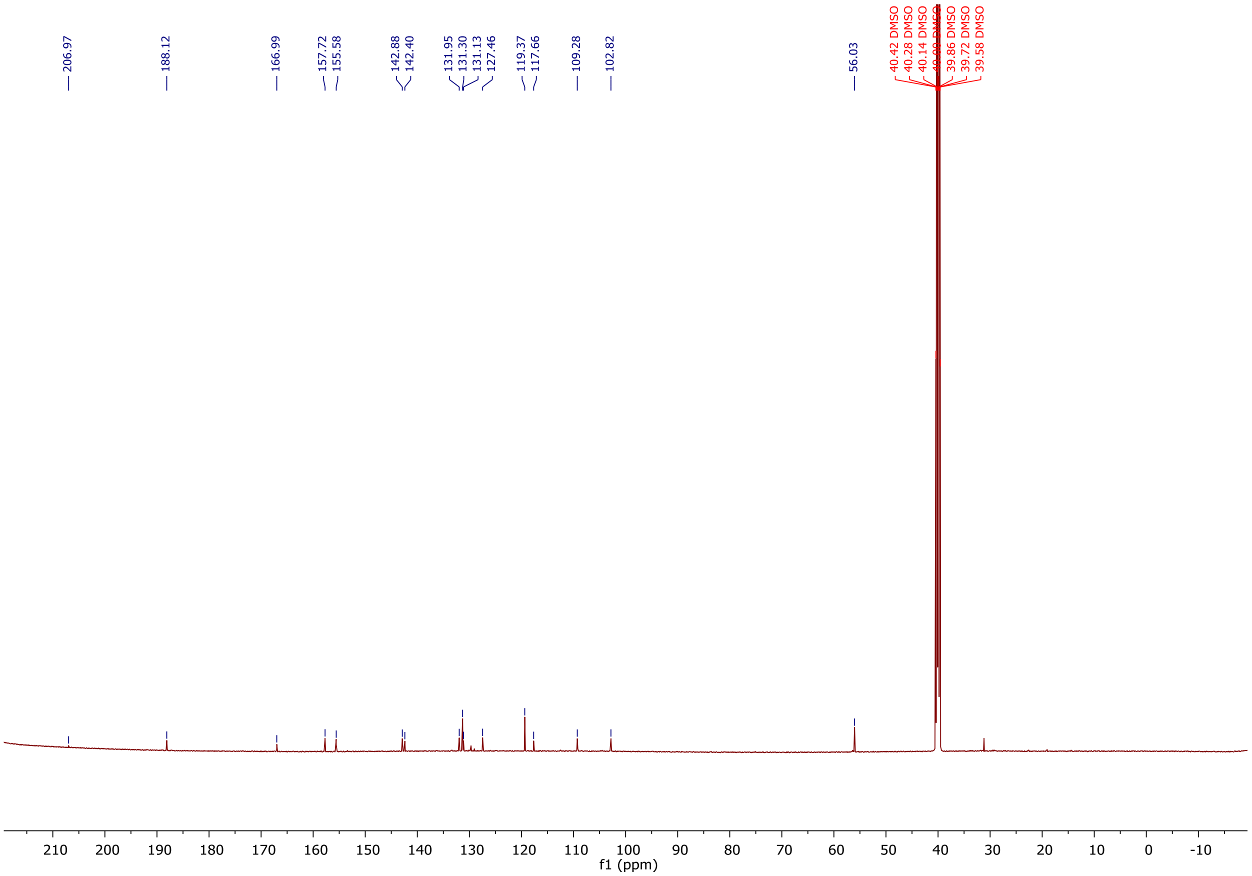

^{13}**C NMR** of compound **CK-85** (151 MHz, DMSO-*d*$_6$).

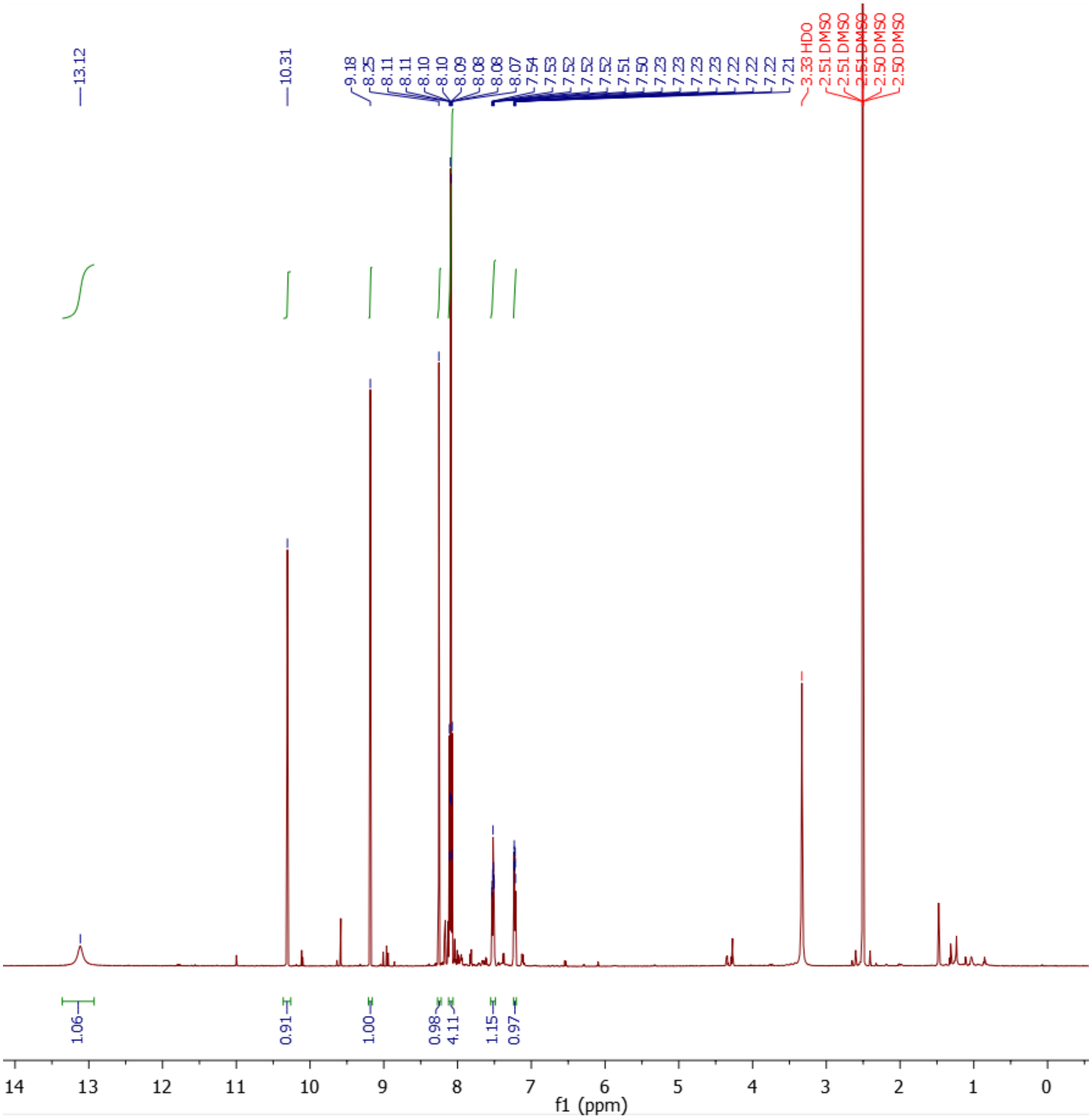

^{1}H NMR of compound **CK-86** (700 MHz, DMSO-d_6).

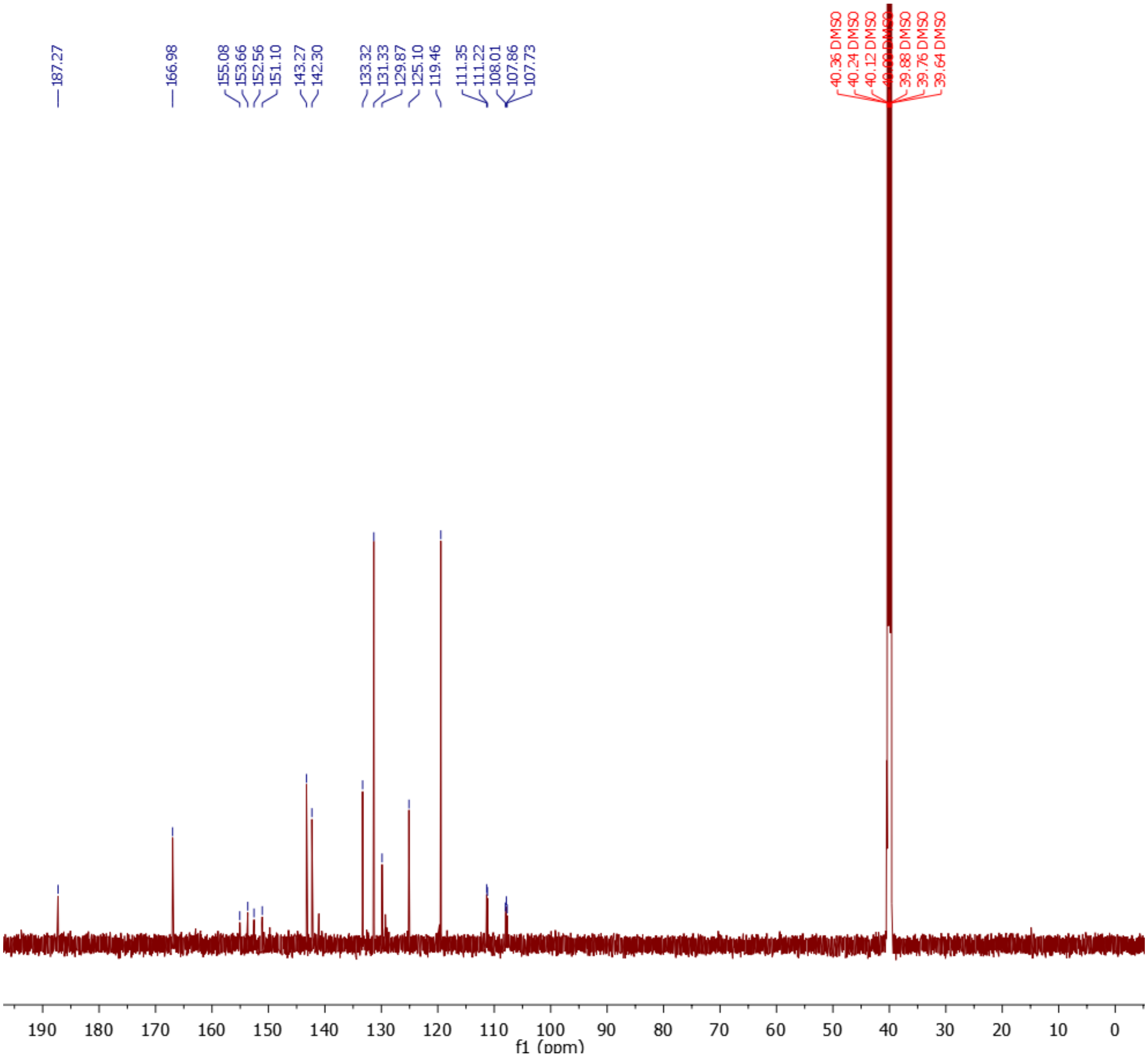

^{13}**C NMR** of compound **CK-86** (176 MHz, DMSO-d_6).

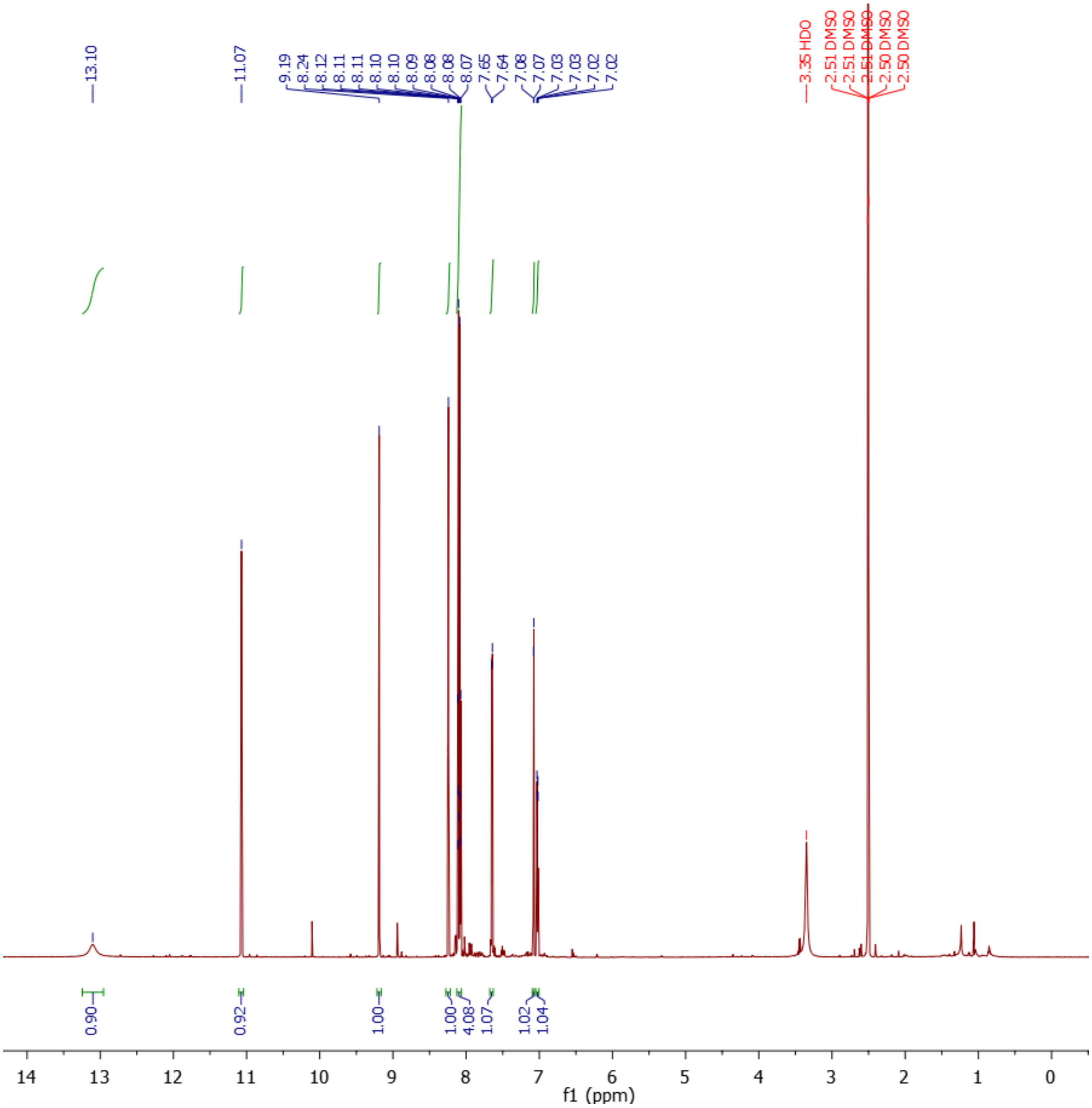

^{1}H NMR of compound **CK-88** (700 MHz, DMSO-*d*$_6$).

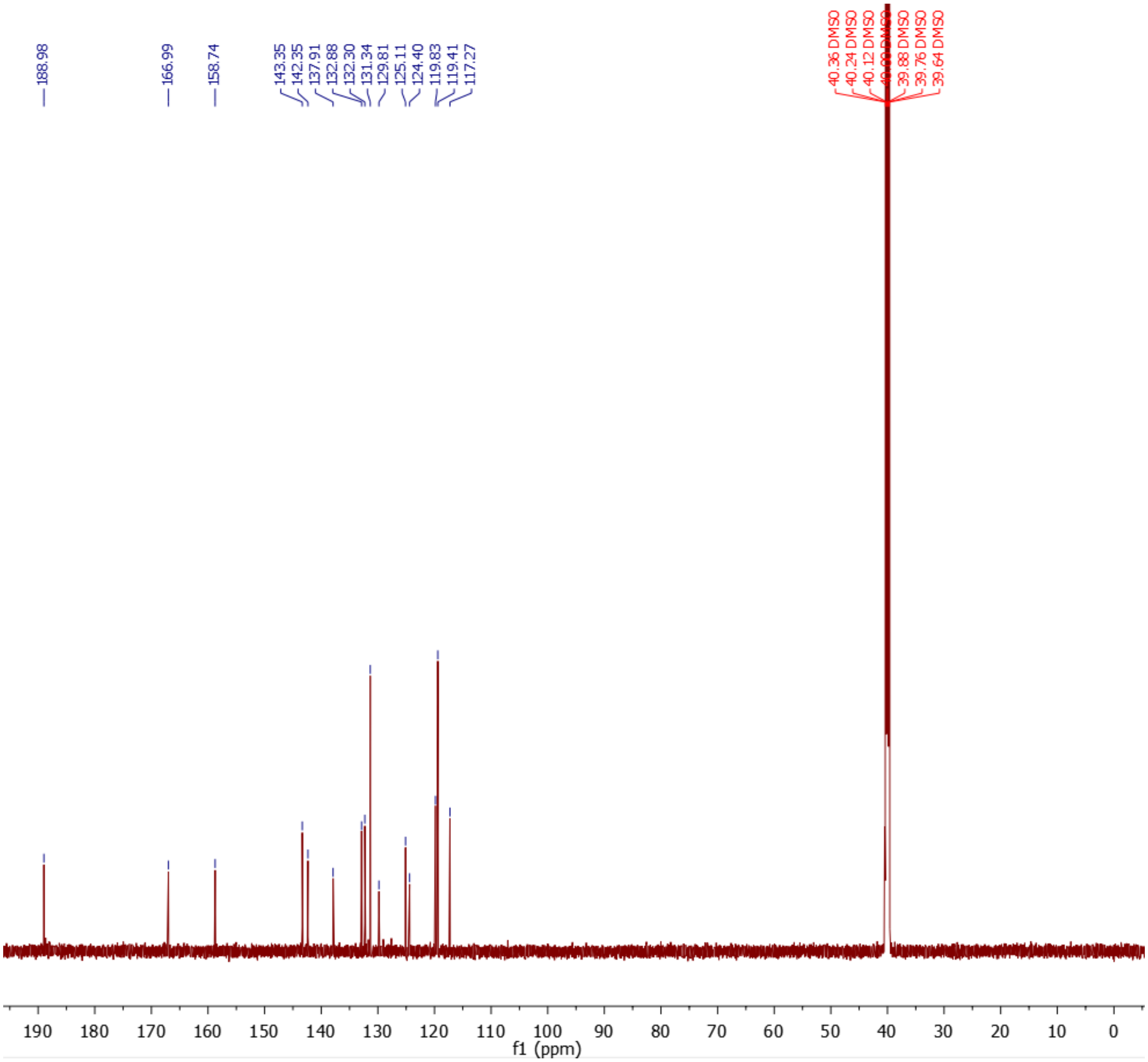

^{13}C NMR of compound **CK-88** (176 MHz, DMSO-d_6).

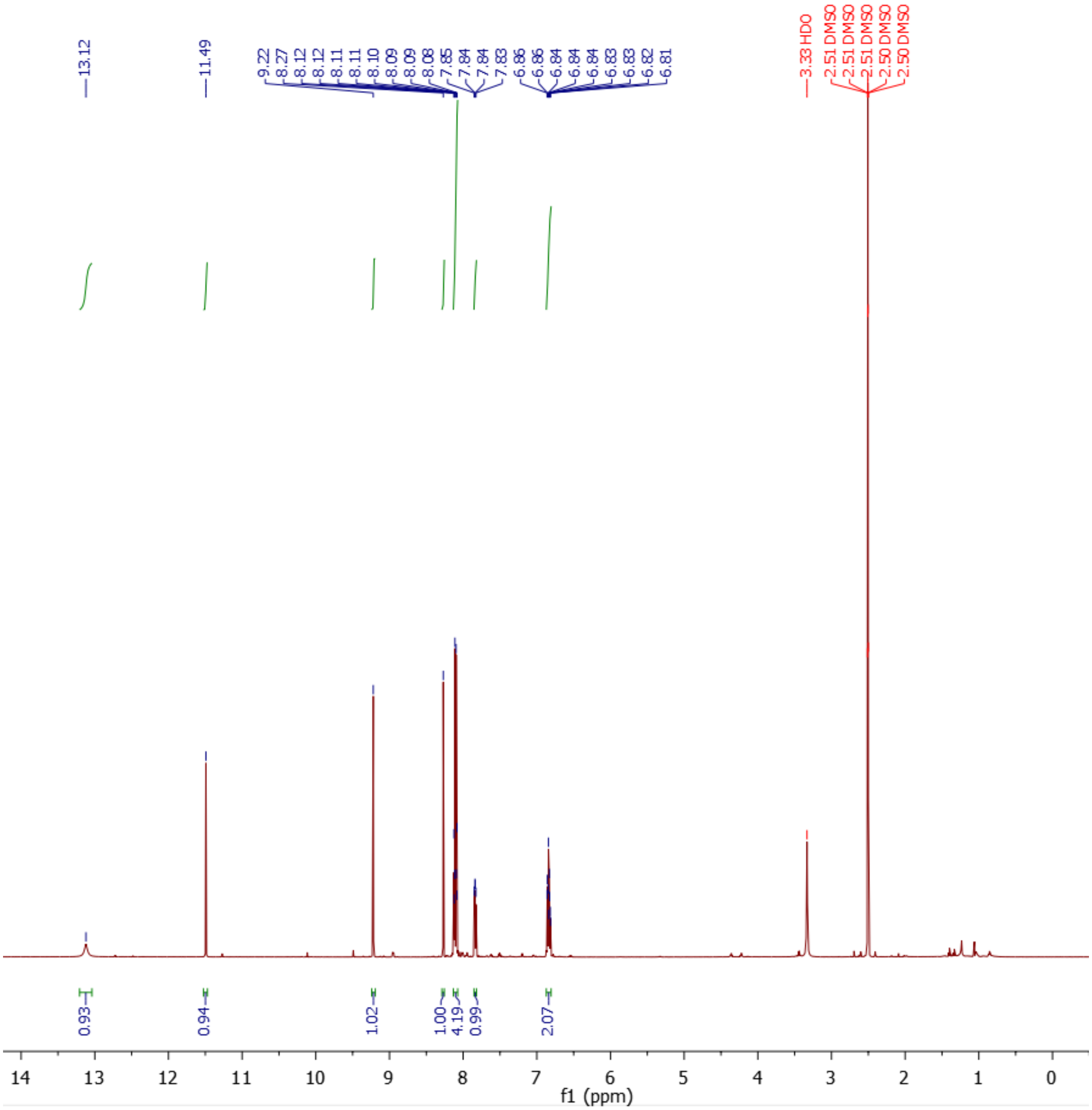

^{1}H NMR of compound **CK-89** (700 MHz, DMSO-*d*6).

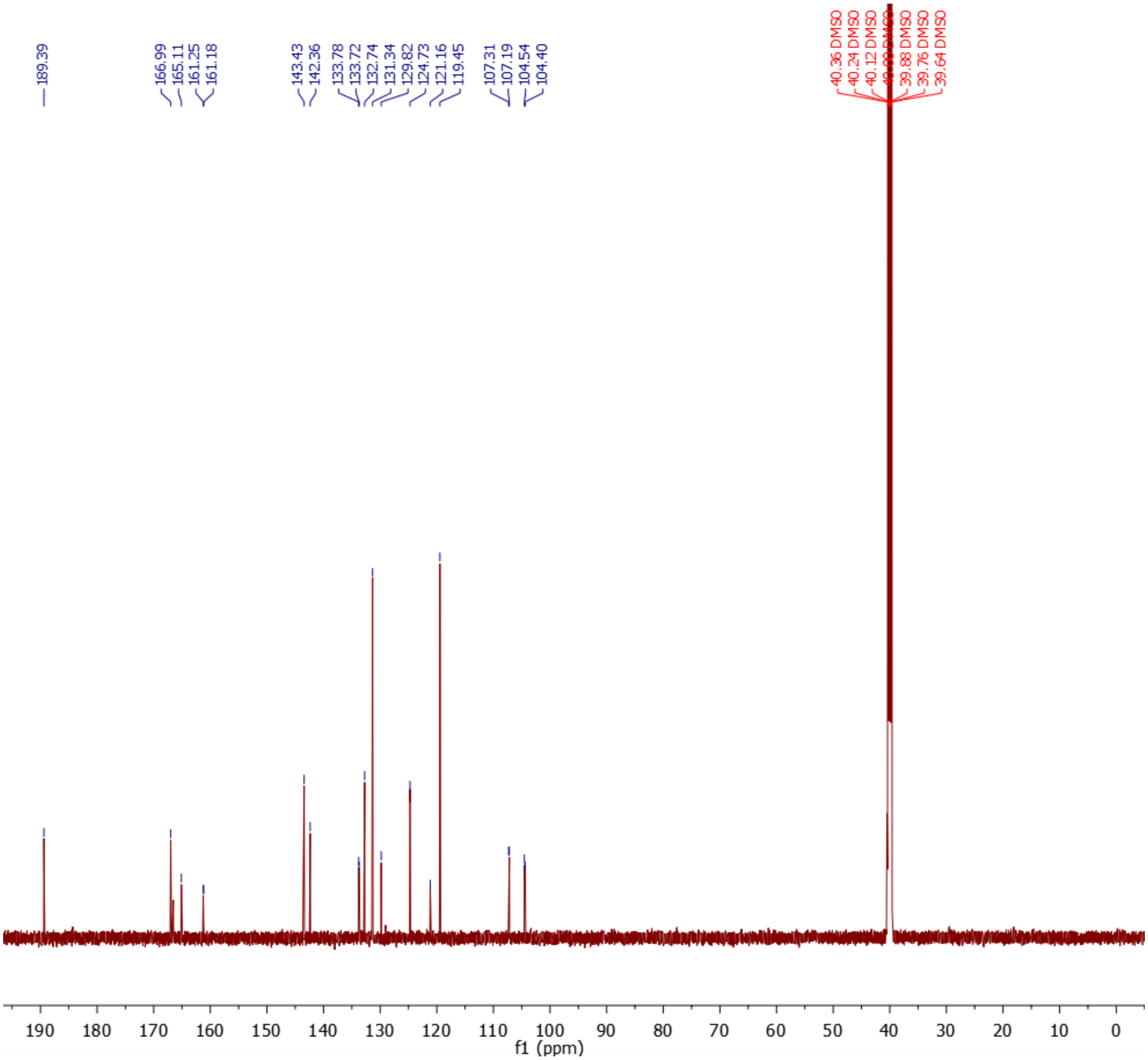

^{13}C NMR of compound **CK-89** (176 MHz, DMSO-d_6).

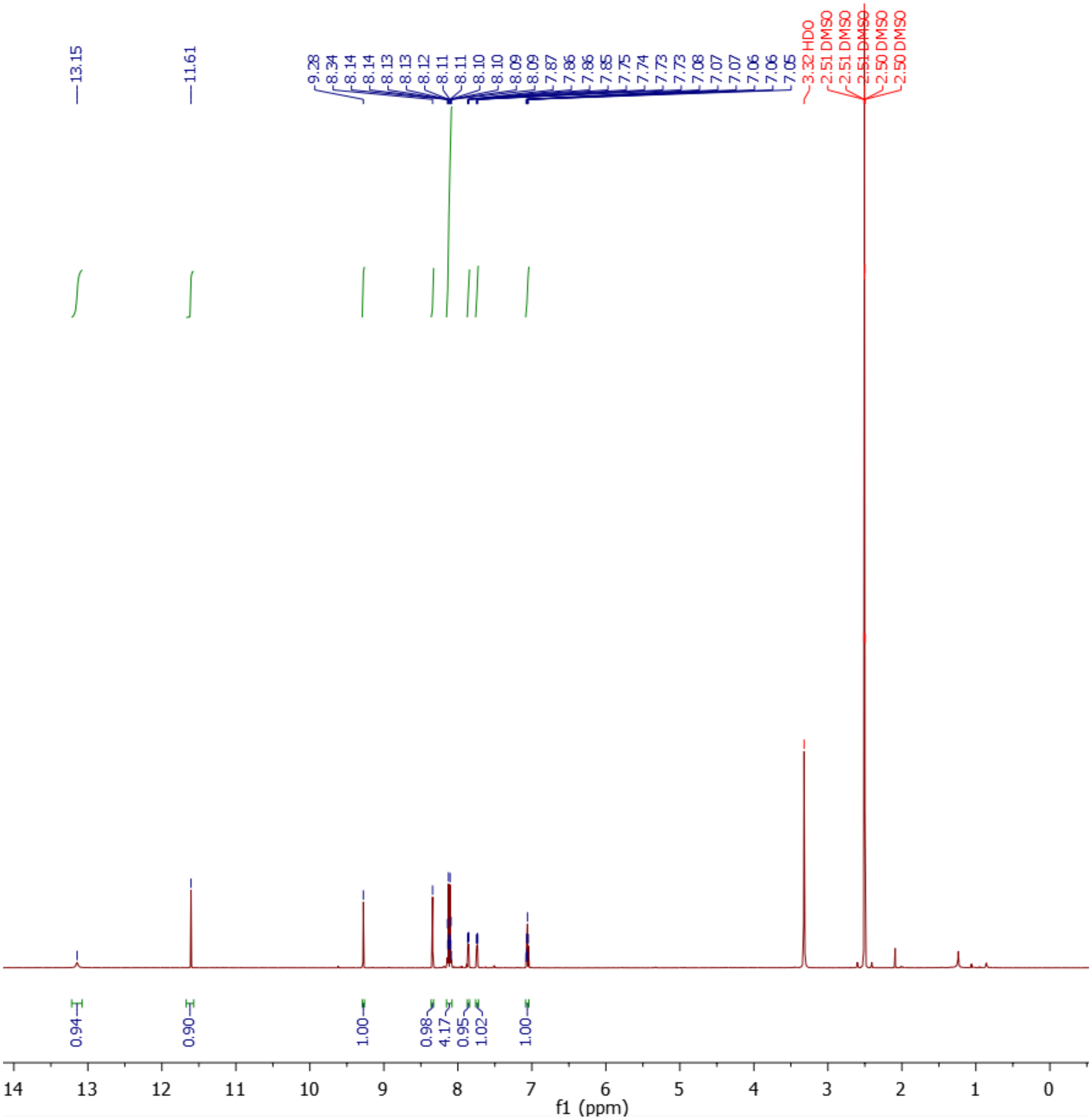

^{1}H NMR of compound **CK-96** (700 MHz, DMSO-d_6).

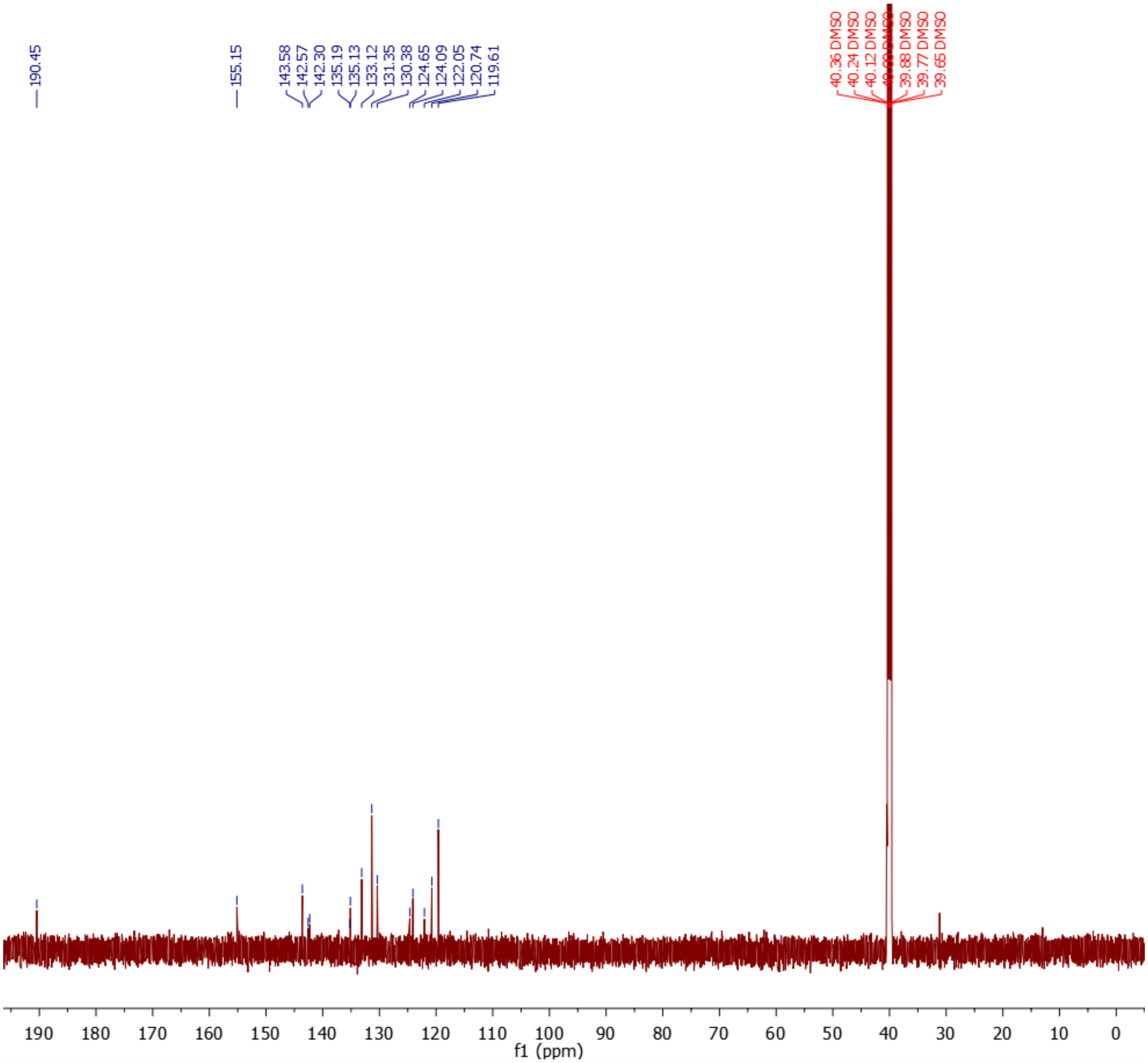

^{13}C NMR of compound **CK-96** (176 MHz, DMSO-d_6).

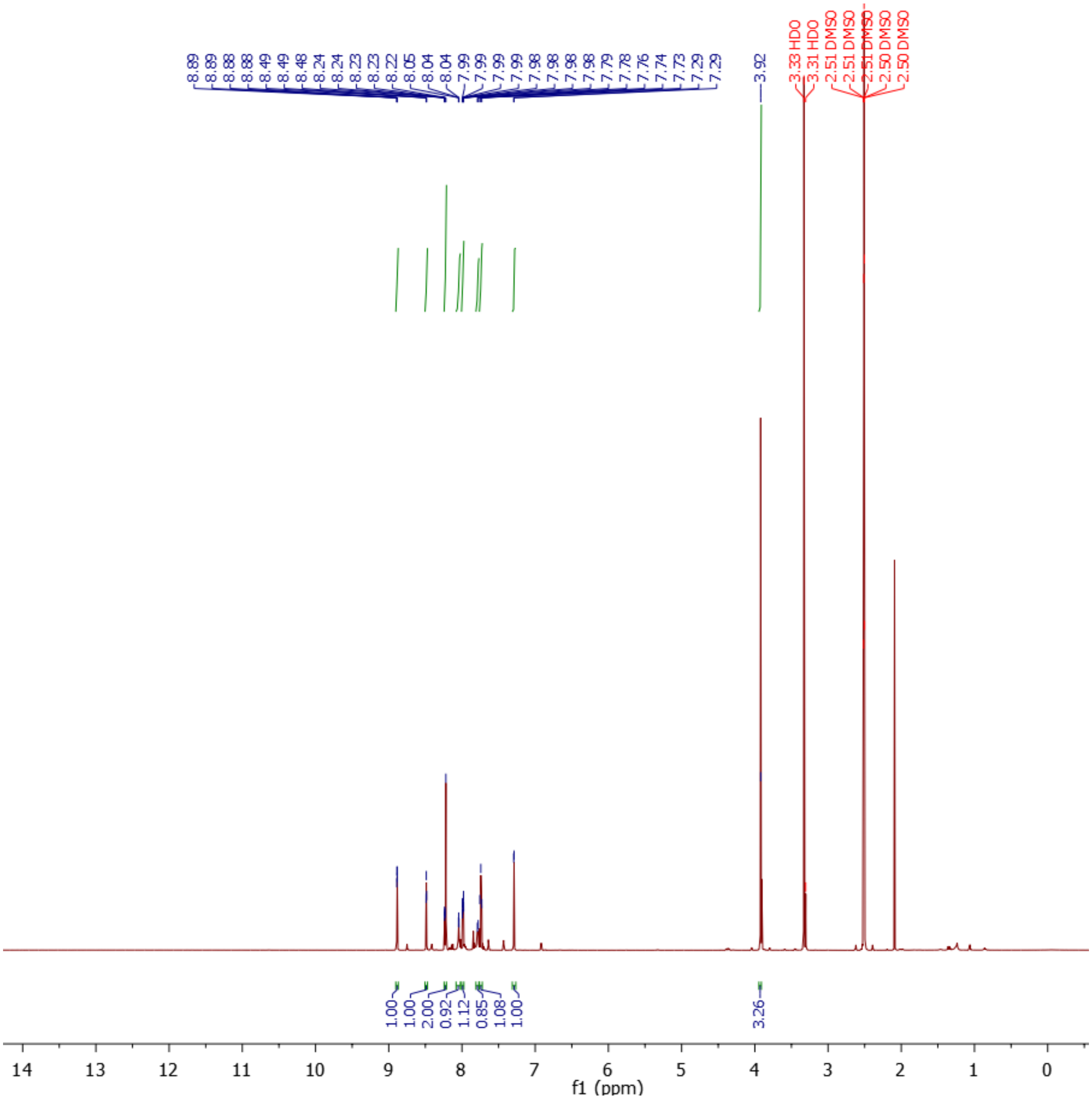

^{1}H NMR of compound **CK-94** (600 MHz, DMSO-d_6).

LC-MS Spectra

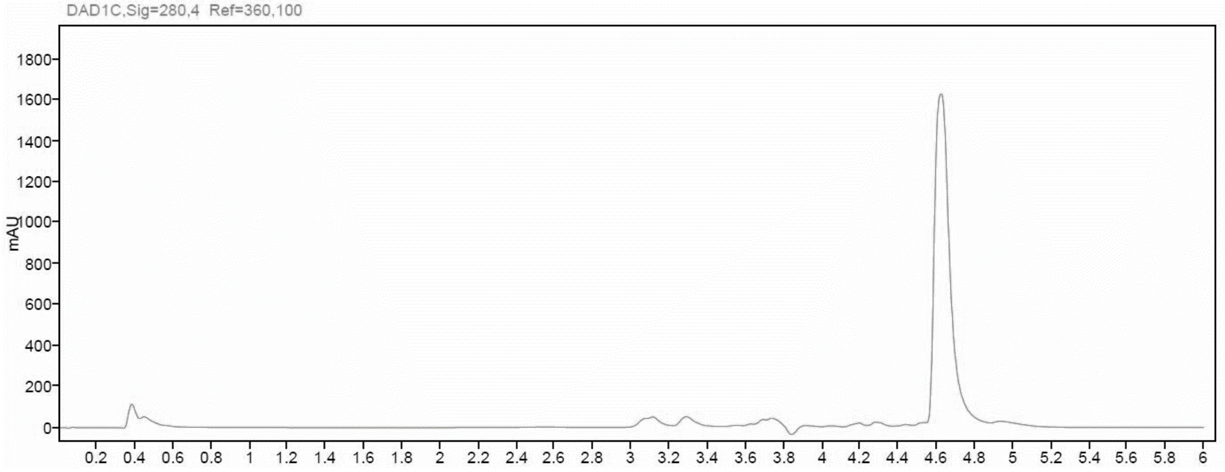

Analytical UHPLC of compound **CK-55**.

Peak RT 4.671

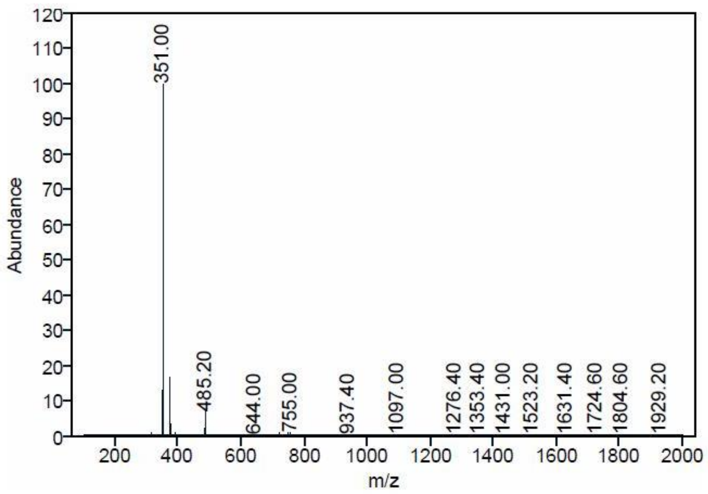

Mass spectrum of compound **CK-55**.

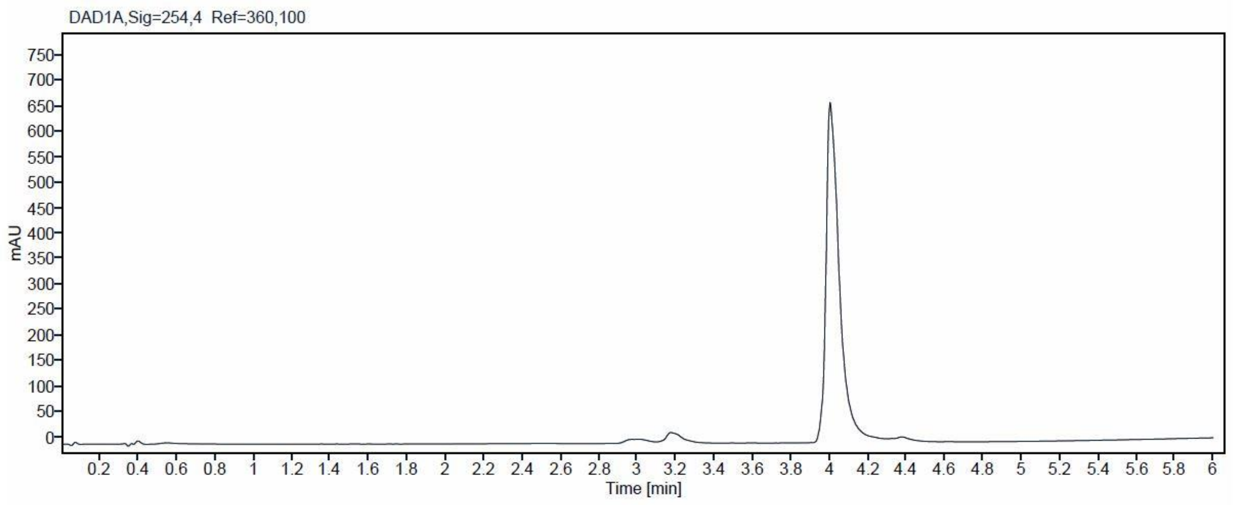

Analytical UHPLC of compound **CK-56**.

Peak RT 4.061

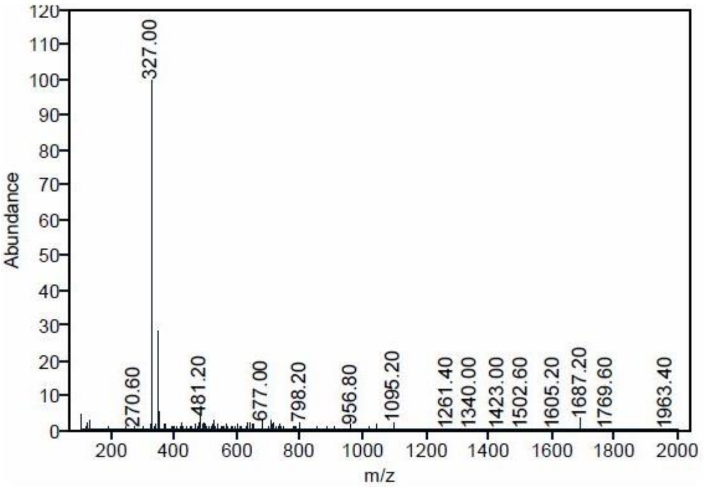

Mass spectrum of compound **CK-56**.

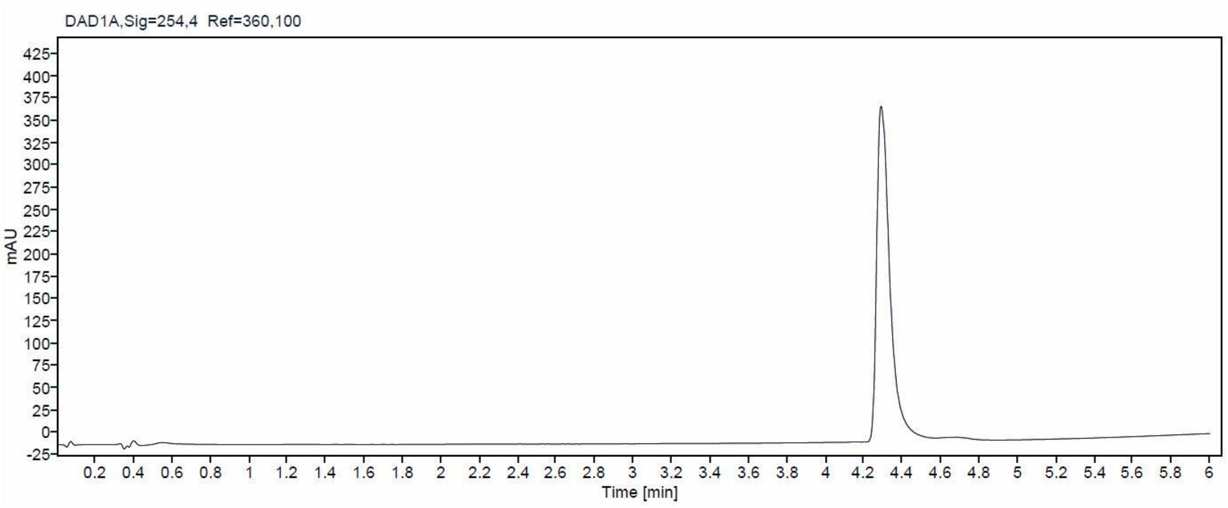

Analytical UHPLC of compound **CK-60**.

Peak RT 4.380

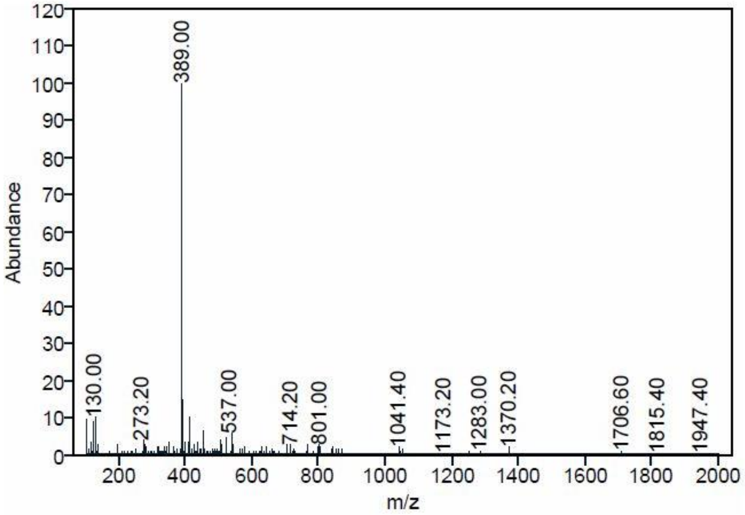

Mass spectrum of compound **CK-60**.

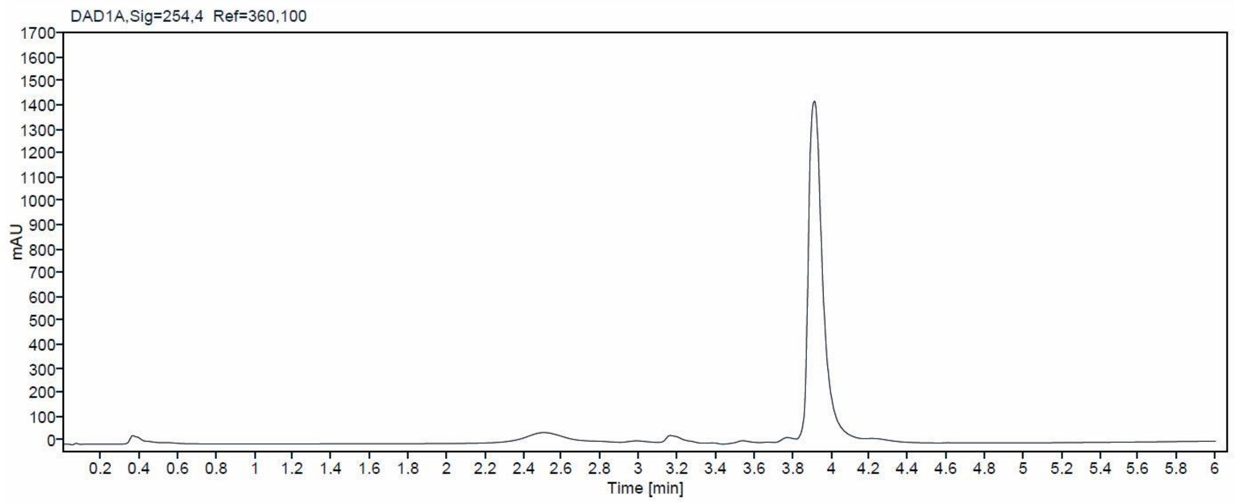

Analytical UHPLC of compound **CK-62**.

Peak RT 3.959

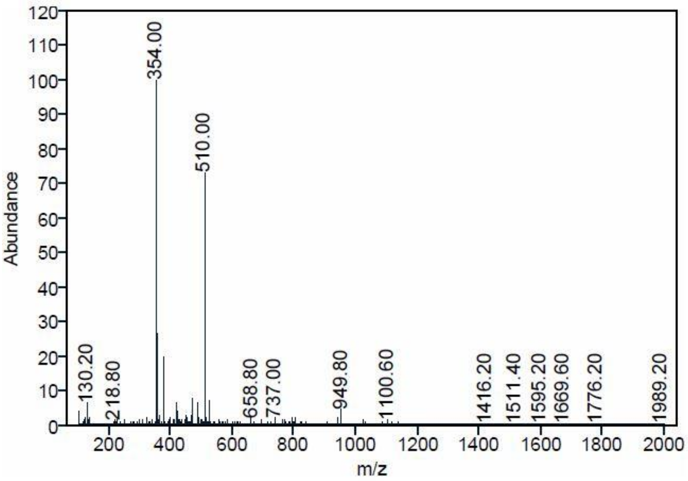

Mass spectrum of compound **CK-62**.

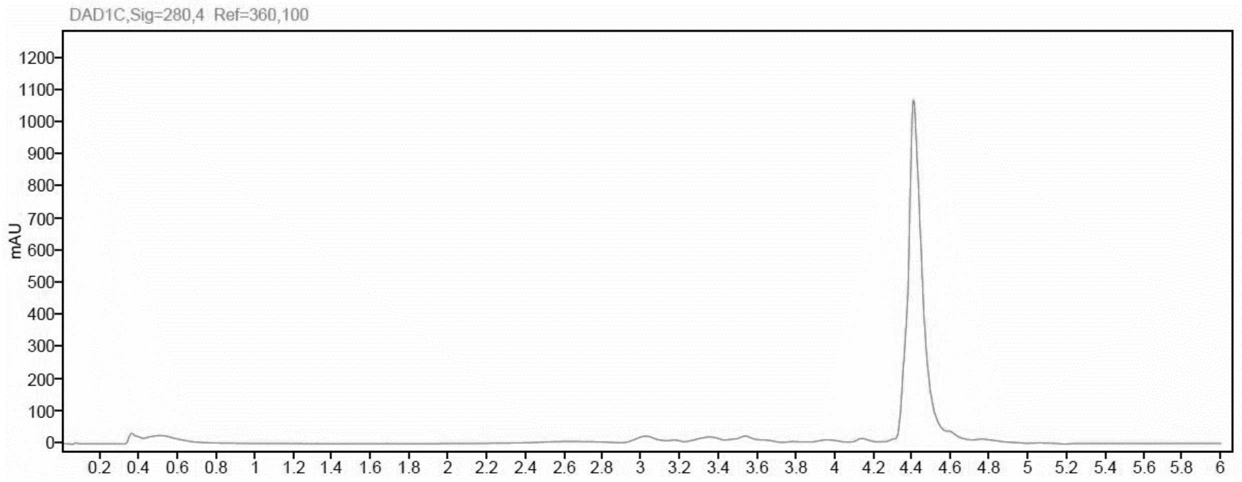

Analytical UHPLC of compound **CK-65**.

Peak RT 4.468

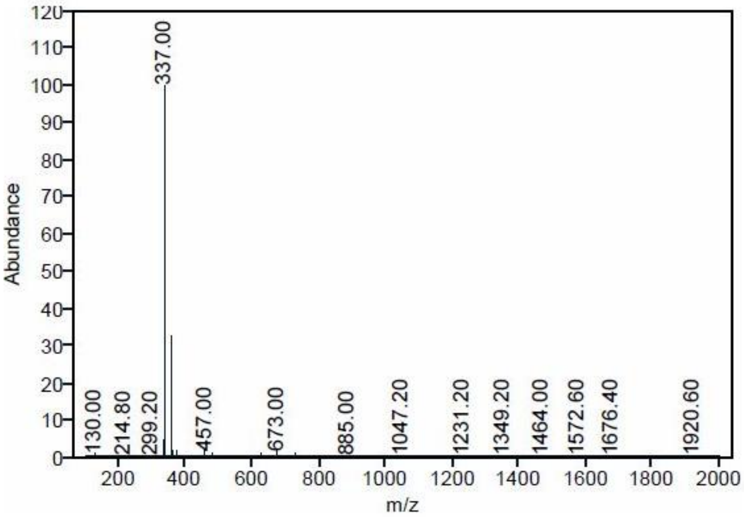

Mass spectrum of compound **CK-65**.

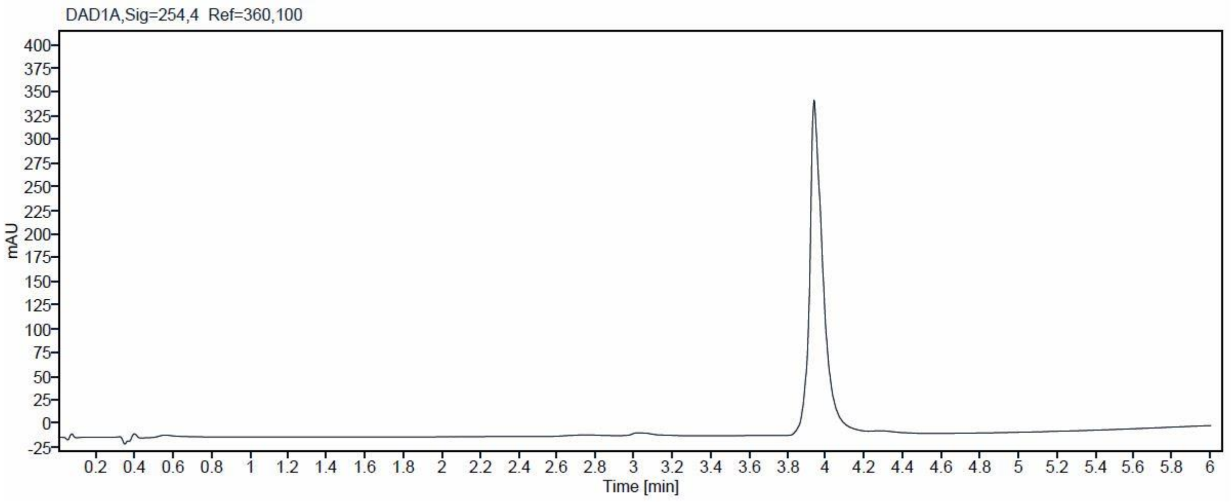

Analytical UHPLC of compound **CK-66**.

Peak RT 4.016

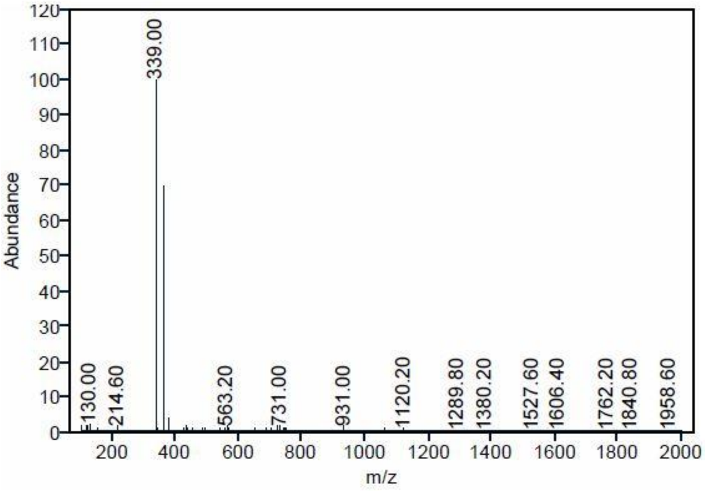

Mass spectrum of compound **CK-66**.

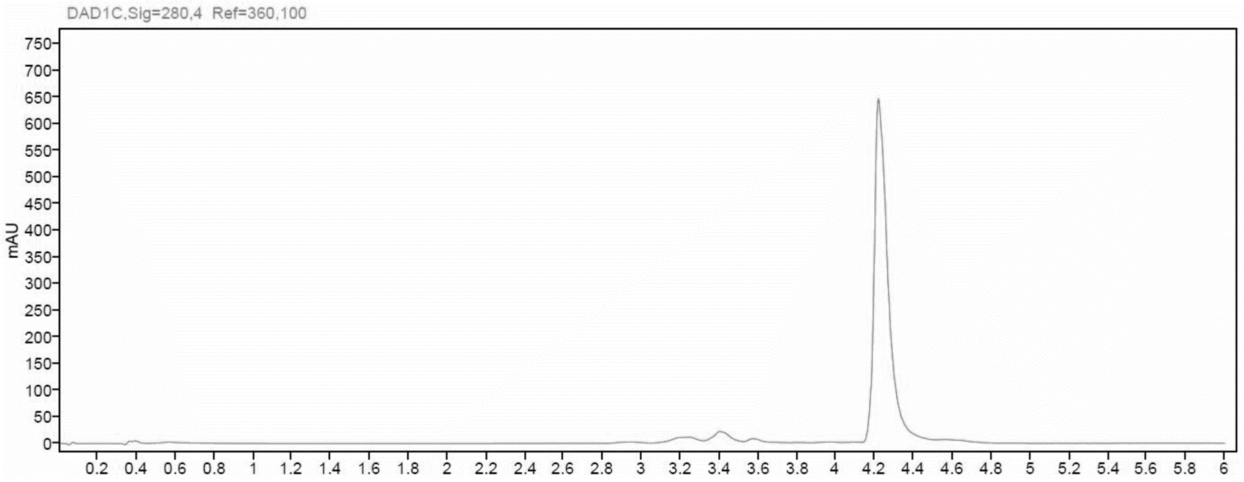

Analytical UHPLC of compound **CK-67**.

Peak RT 4.273

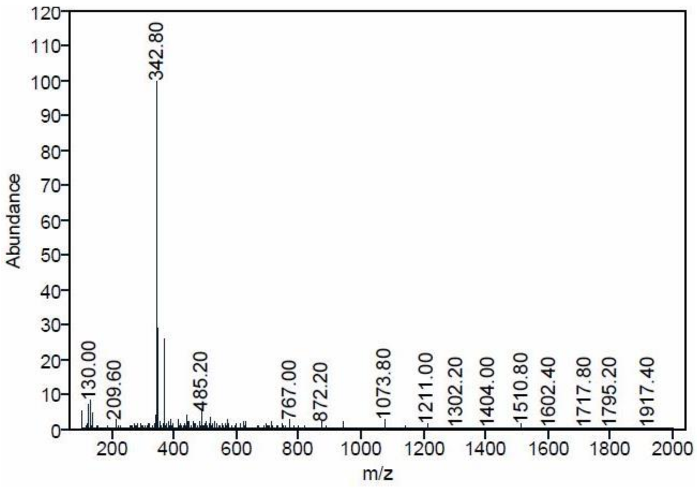

Mass spectrum of compound **CK-67**.

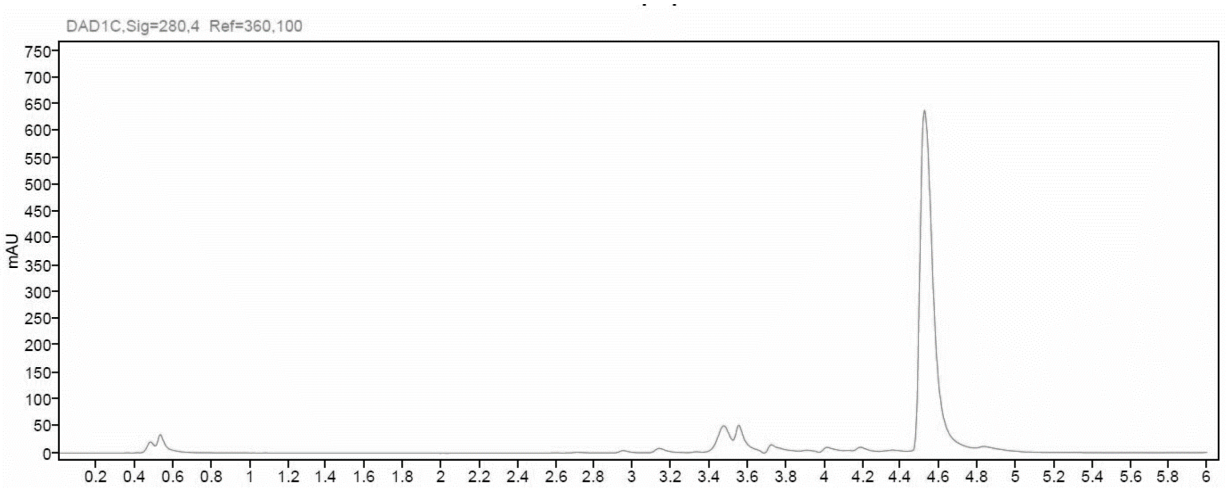

Analytical UHPLC of compound **CK-70**.

Peak RT 4.602

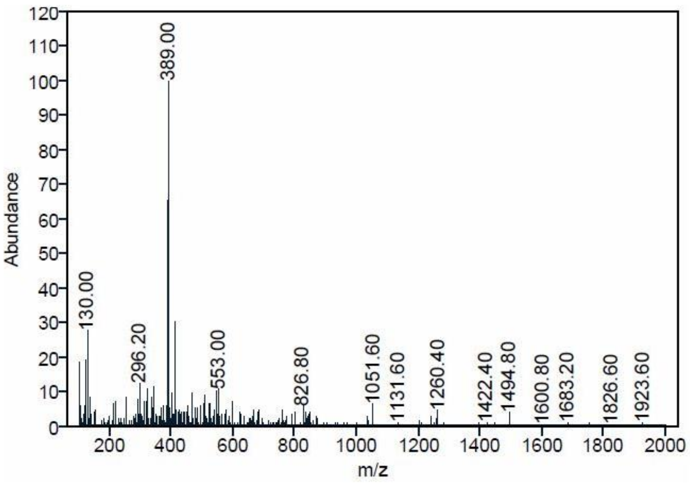

Mass spectrum of compound **CK-70**.

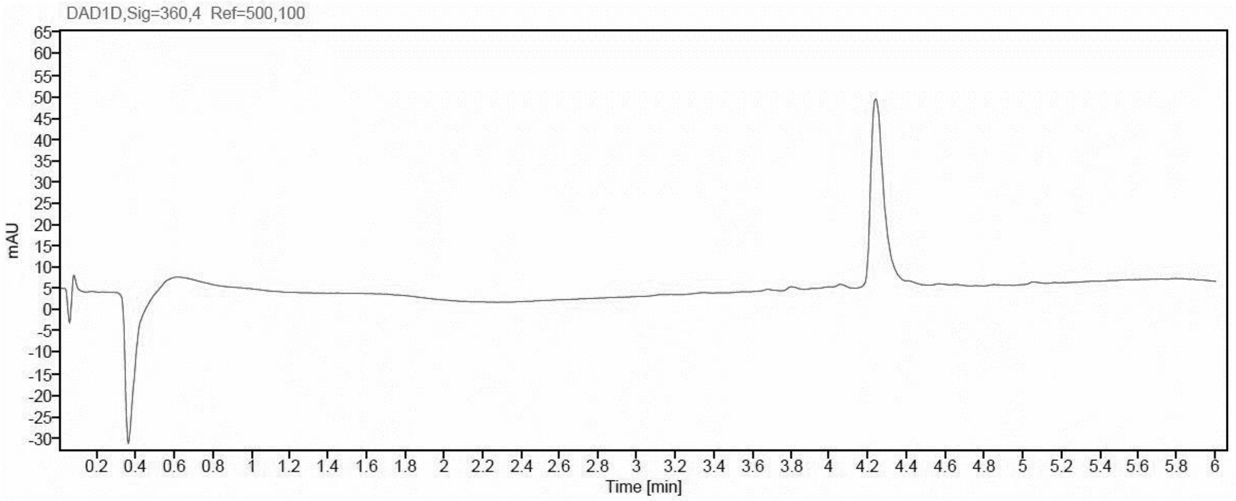

Analytical UHPLC of compound **CK-73**.

Peak RT 4.306

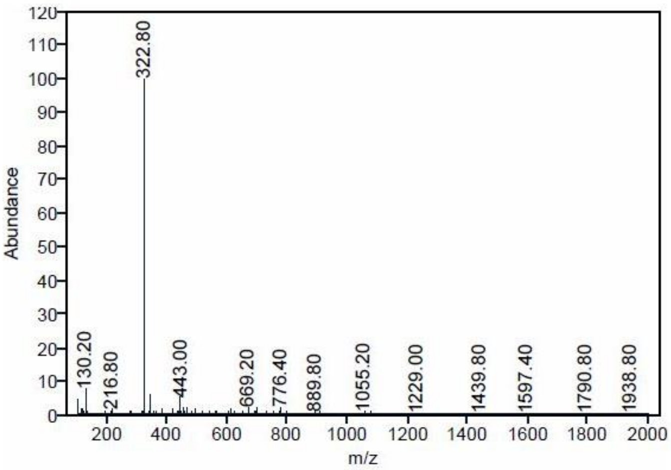

Mass spectrum of compound **CK-73**.

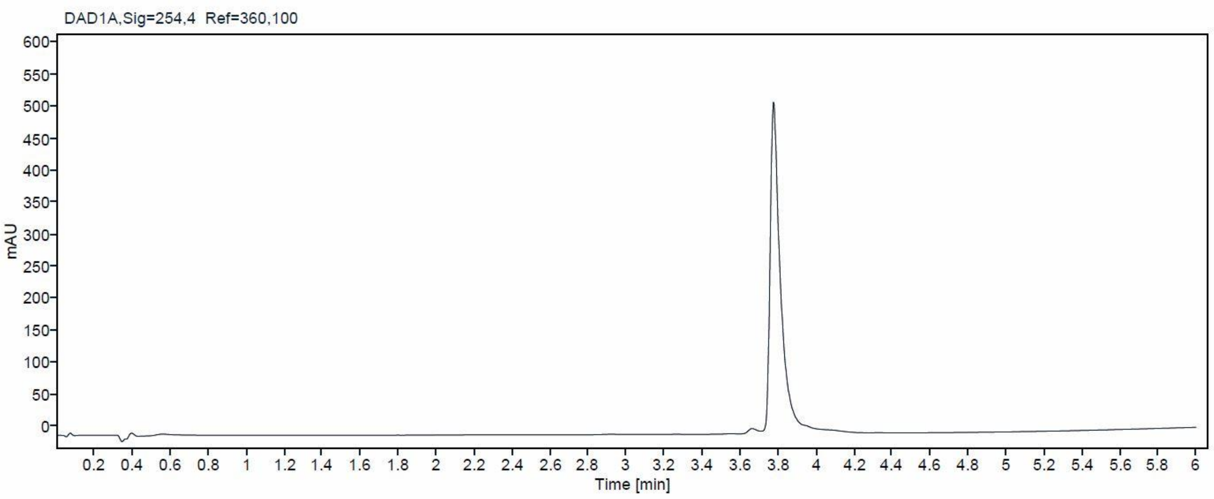

Analytical UHPLC of compound **CK-75**.

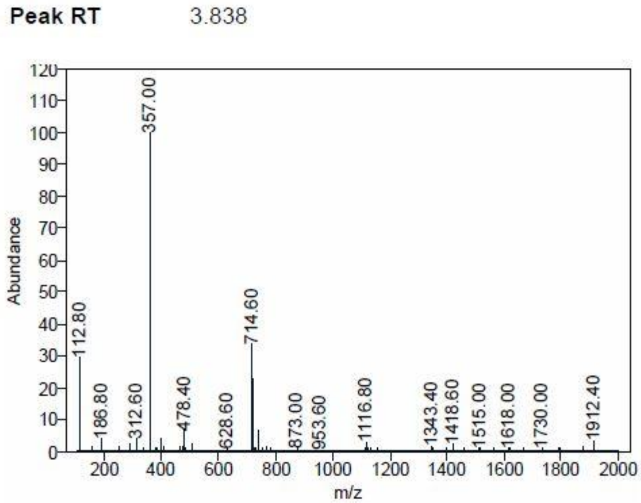

Mass spectrum of compound **CK-75**.

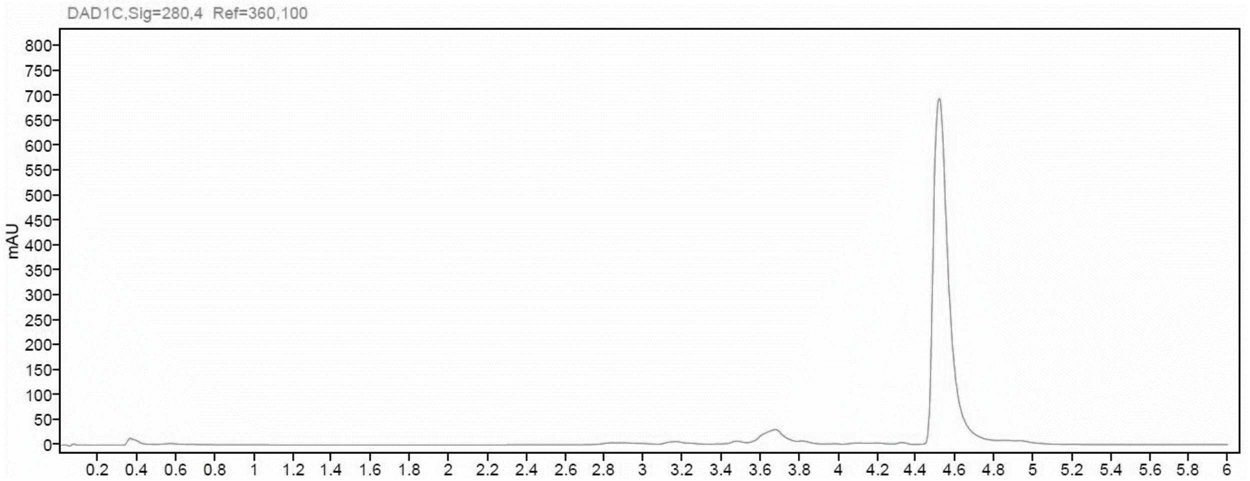

Analytical UHPLC of compound **CK-79**.

Peak RT 4.583

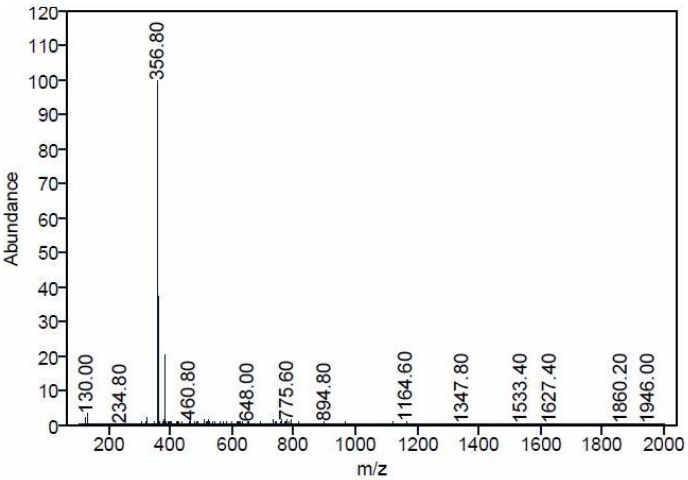

Mass spectrum of compound **CK-79**.

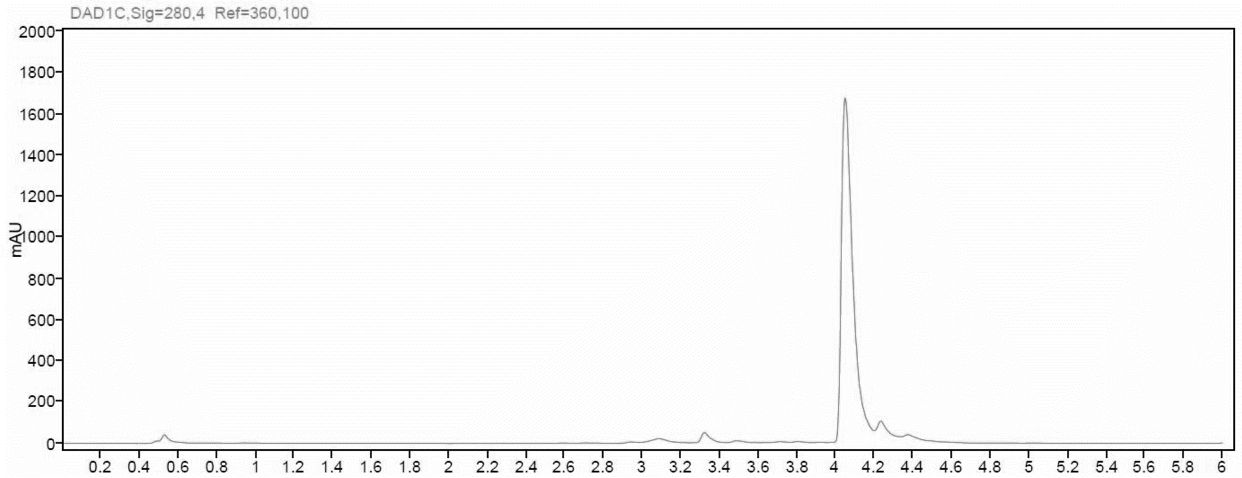

Analytical UHPLC of compound **CK-86**.

Peak RT 4.110

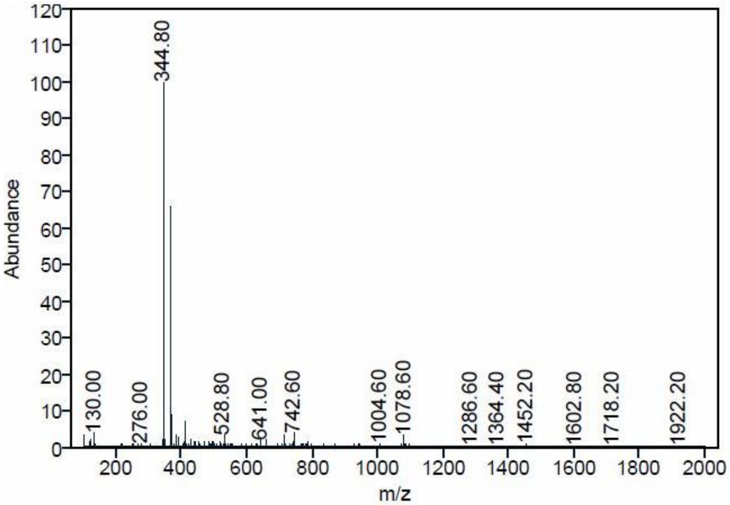

Mass spectrum of compound **CK-86**.

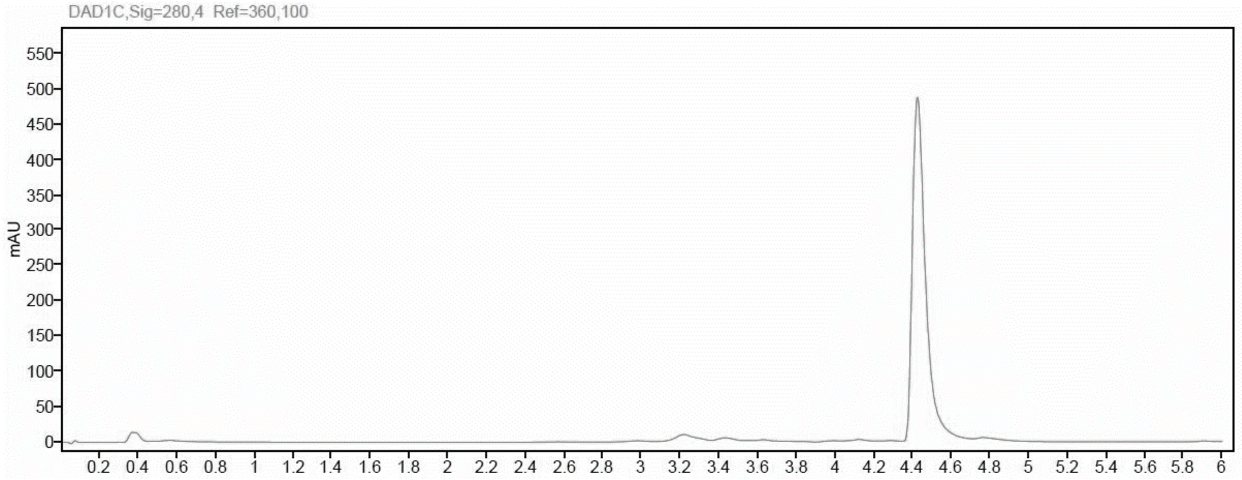

Analytical UHPLC of compound **CK-88**.

Peak RT 4.486

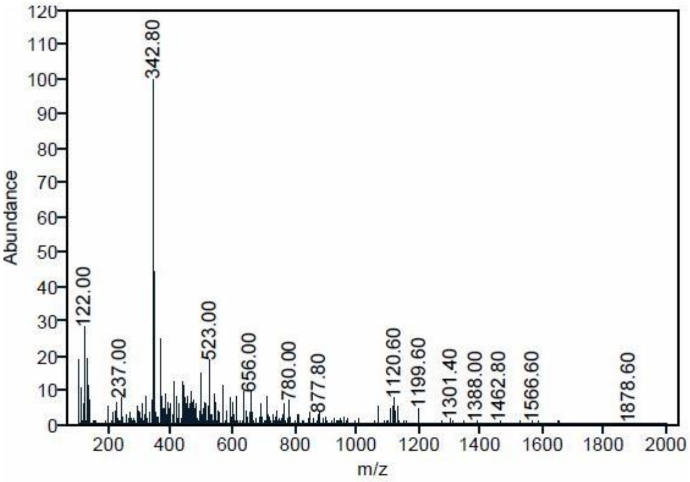

Mass spectrum of compound **CK-88**.

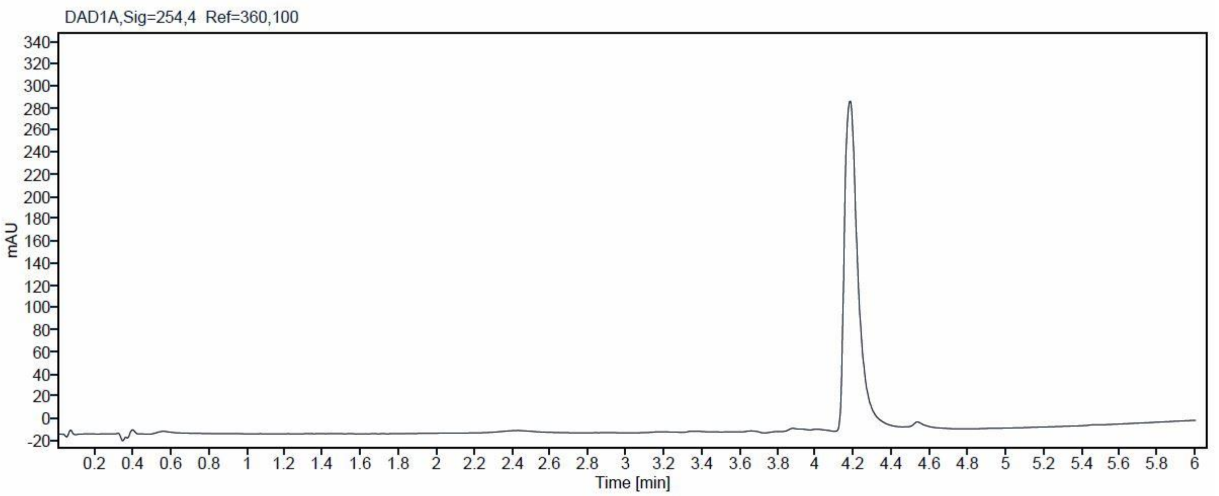

Analytical UHPLC of compound **CK-89**.

Peak RT 4.245

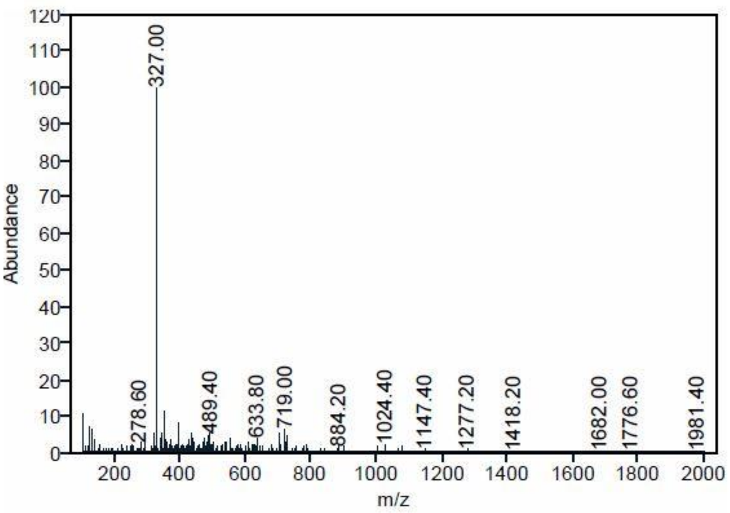

Mass spectrum of compound **CK-89**.

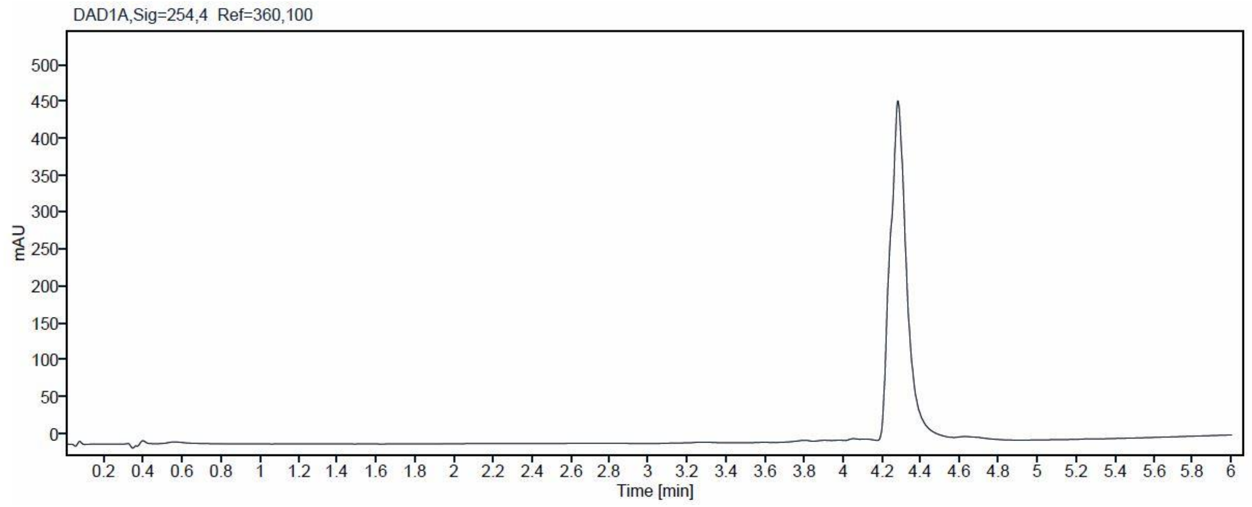

Analytical UHPLC of compound **CK-96**.

Peak RT 4.351

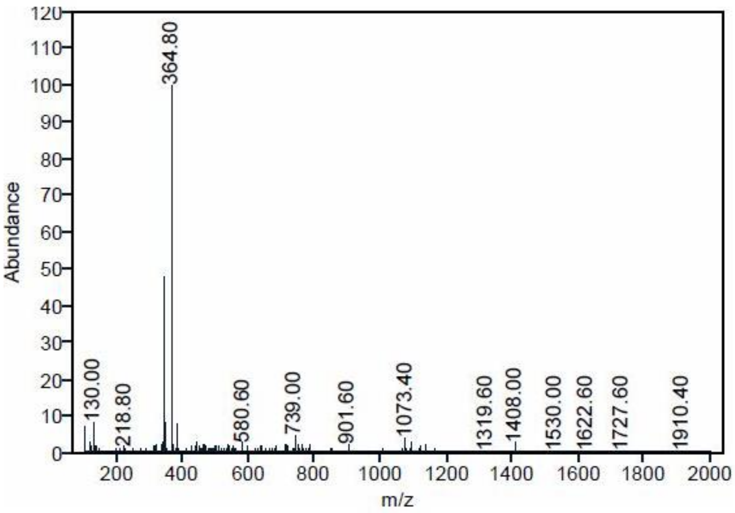

Mass spectrum of compound **CK-96**.

References

1. Khan, K. M. *et al.* 3-Formylchromones: Potential antiinflammatory agents. *Eur. J. Med. Chem.* **45,** 4058–4064 (2010).

2. Ulven, T. *et al.* Novel Selective Orally Active CRTH2 Antagonists for Allergic Inflammation Developed from in Silico Derived Hits. *J. Med. Chem.* **49,** 6638–6641 (2006).

3. Figarella, K. *et al.* Antileishmanial and Antitrypanosomal Activity of Synthesized Hydrazones, Pyrazoles, Pyrazolo[1,5-a]-Pyrimidines and Pyrazolo[3,4-b]-Pyridine. *Curr. Bioact. Compd.* **14,** 234–239 (2018).

4. Bhimapaka, C. R. *et al.* Synthesis and antiproliferative activity of 4H-chromonephenylhydrazones, 1H-pyrazolecarboxylates and pyrazolylmethanones. *Acta Chim. Slov.* **65,** 34–49 (2018).

5. Gellis, A., Yves, N., Crozet, M. P. & Vanelle, P. One Pot Synthesis of 4-(2-Hydroxybenzoyl)-pyrazoles from 3-Formylchromones Under Microwave Irradiation in Solvent Free Conditions. *Synth. Commun.* **31,** 1257–1262 (2001).
